# Unmasking Activation of the Zygotic Genome Using Chromosomal Deletions in the *Drosophila* Embryo 

**DOI:** 10.1371/journal.pbio.0050117

**Published:** 2007-04-24

**Authors:** Stefano De Renzis, Olivier Elemento, Saeed Tavazoie, Eric F Wieschaus

**Affiliations:** 1 Howard Hughes Medical Institute, Department of Molecular Biology, Princeton University, New Jersey, United States of America; 2 Lewis-Sigler Institute for Integrative Genomics, Princeton University, Princeton, New Jersey, United States of America; University of California San Francisco, United States of America

## Abstract

During the maternal-to-zygotic transition, a developing embryo integrates post-transcriptional regulation of maternal mRNAs with transcriptional activation of its own genome. By combining chromosomal ablation in *Drosophila* with microarray analysis, we characterized the basis of this integration. We show that the expression profile for at least one third of zygotically active genes is coupled to the concomitant degradation of the corresponding maternal mRNAs. The embryo uses transcription and degradation to generate localized patterns of expression, and zygotic transcription to degrade distinct classes of maternal transcripts. Although degradation does not appear to involve a simple regulatory code, the activation of the zygotic genome starts from intronless genes sharing a common *cis*-element. This *cis*-element interacts with a single protein, the Bicoid stability factor, and acts as a potent enhancer capable of timing the activity of an exogenous transactivator. We propose that this regulatory mode links morphogen gradients with temporal regulation during the maternal-to-zygotic transition.

## Author Summary

Embryonic development is controlled by a complex interaction between maternal and zygotic activities. Maternal messenger RNAs and proteins are deposited in the unfertilized egg during oogenesis; after fertilization, the activation of the zygotic genome is accompanied by the degradation of a fraction of maternally supplied transcripts. This switch from maternal to zygotic control of development is characterized by a dramatic remodeling of gene expression, and represents a universal regulatory point during animal development. Because it is not usually possible to identify which genomes are contributing to these transcriptional changes, we have used chromosomal ablation to determine maternal versus zygotic contribution for each mRNA detectable on microarray in the *Drosophila* blastoderm. This has allowed us to distinguish transcriptional and post-transcriptional modes of regulation and to identify common *cis*-regulatory elements associated with different classes of transcripts. Our analysis revealed that although mRNA degradation does not involve a simple regulatory code, the activation of the zygotic genome is based on a simple mechanism, which links morphogen gradients with temporal regulation. It will be interesting to address whether similar mechanisms also operate in other animals.

## Abstract 3

As the egg develops into the embryo, maternal mRNAs are degraded and new genes activated. By using chromosomal ablation in*Drosophila,* the authors characterized the basis of this integration.

## Introduction

Embryonic development is controlled by a complex interaction between maternal and zygotic activities. Although maternal transcripts and proteins are deposited in the egg during oogenesis, the activation of the zygotic genome starts at different stages in different animals and is concomitant with the degradation of a fraction of maternally supplied transcripts [1–3]. Thus, during the maternal-to-zygotic transition (MZT), the embryo undergoes an extensive remodeling of gene expression and must integrate post-transcriptional regulatory mechanisms, which are the only ones operating during the previous maternal stages, with transcriptional regulation of its own genome. How this is achieved is poorly understood.

The concomitant degradation of maternal transcripts and activation of zygotic transcription has made it difficult in any animal to interpret changes in gene expression [4–6]. Whereas an increase in gene expression levels can be interpreted as a sign of zygotic transcription, a decrease or absence of change is also consistent with zygotic gene activation if it is accompanied by maternal mRNA degradation. One way to test whether a particular RNA is supplied maternally or zygotically is to compare its levels in embryos that have or do not have the corresponding DNA template. Under these conditions, differences in expression level indicate the relative maternal and zygotic contribution. Drosophila melanogaster offers the unique opportunity to perform such an experiment for the entire genome, as it is possible to use chromosomal rearrangements to produce embryos that lack specific arms or even entire chromosomes [7,8]. Such embryos develop normally until cycle 14 and then show defects characteristic of the chromosomal region deleted. The results of such experiments suggest that the *Drosophila* embryo develops under the control of maternally provided proteins until nuclear division 13. This stage, usually referred to as the mid-blastula transition (MBT), defines the point from which development comes to be controlled by the zygote's own genome [1]. The first morphological signs of the zygotic genome appear with the cellularization of the cortically migrating nuclei and the beginning of gastrulation. From a transcriptional point of view, the zygotic genome is silent until nuclear cycle 9–10 [9]. In the germline, this quiescence is maintained until later stages of development, arguing for specific regulation between the soma and the germline [10].

The molecular mechanisms linking the nuclear cycles to the activation of transcription are unknown and may involve the chromosomal squelching of negative regulators of transcription, as has been proposed for the *Xenopus* embryo [3]. Chromatin-based mechanisms may also play a role. In the mouse embryo, for example, at least one cycle of DNA replication is required to change the methylation state of the chromatin to a transcriptionally competent conformation [11]. However, in none of these organisms have the molecular players actually regulating activation of the zygotic genome been identified. Because such regulators must be maternally provided, they are not easily identifiable in genetic screens. On the other hand, the recent technological advances in genomics and bioinformatics may offer alternative strategies for elucidating this mechanism, especially if the identification of *cis*-regulatory elements can be coupled to a biochemical characterization of the factors that bind to them.

Here we took advantage of the phenotype generated by the removal of specific genes acting during cellularization to identify embryos lacking defined chromosomal arms, and analyzed their expression profiles using microarrays. Because this strategy allows discrimination between transcriptional and post-transcriptional regulation of gene expression, we describe here the first complete analysis of the MZT during animal development.

## Results

### Time-Course Analysis

Earlier attempts to identify zygotically active genes in *Drosophila* relied on comparing mRNA levels at cycle 14 with those from unfertilized eggs or early 0–1-h-old embryos [12]. Although zygotic transcription begins already at earlier nuclear cycles (9–10), we also started our analysis by focusing on cycle 14 because this stage represents the earliest time point at which the mutant phenotypes associated with the deletion of each specific chromosome can be recognized. The time-course characterization of earlier time points will be presented in the section describing the activation of the zygotic genome. The temporal resolution of our measurements is at 1-h intervals covering the first 3 h of embryogenesis: (1) unfertilized eggs, (2) 0–1 h (cycles 1 to 10), (3) 1–2 h (cycles 10 to 13), and (4) 2–3 h (cycle 14).

**Figure 1:**
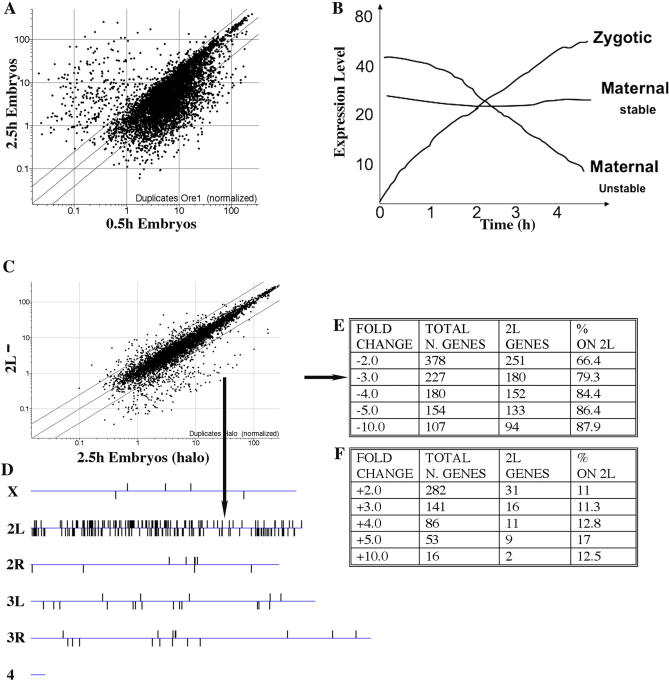
Time-Course Analysis of the MZT and Ablation of the Left Arm of the Second Chromosome (A) Scatter plot representation of freshly fertilized eggs (0–1 h = 0.5 h) and mid-cycle 14 embryos (2–3 h = 2.5 h) microarray measurements. This result represents the average of two biological replicates. Note the extensive degradation of maternal mRNAs appearing as a large number of dots below the 3-fold diagonal lines. Each dot corresponds to an individual probe. The labeling “Duplicates OreR” in the *x*-axis of the scatter plot refer to the fly strain (Oregon R) used to collect the WT embryos. (B) Cartoon indicating the three possible outcomes of time-course microarray measurements during the MZT. (C) Scatter plot analysis of embryos missing the left arm of the second chromosomes (2L−) compared to similarly staged cycle 14 embryos mutant for the *halo* gene. Both population of embryos were identified based on the halo phenotype, see also Figure 2E. Results represent the average of four biological replicates. (D) Genes whose expression was down-regulated 3-fold in the 2L− collection map predominately to 2L. (E and F) Chromosomal distribution of down-regulated (E) and up-regulated (F) genes at different cut-off (*p* < 0.001). Only down-regulated genes were enriched on 2L.

Figure 1A plots the levels of mRNAs from visually staged 0–1-h eggs with those that have developed to cycle 14 (2–3 h). In principle, this type of measurement allows identification of the following categories of transcripts: (1) purely zygotic (transcripts that are not expressed at 0–1 h and are detected as present at 2–3 h), (2) maternal+zygotic (transcripts that are present at 0–1 h and whose level increases at 2–3 h), and (3) maternal or maternal+zygotic (transcripts that are present at 0–1 h and whose level either does not change or decreases in level at 2–3 h).

**Figure 2:**
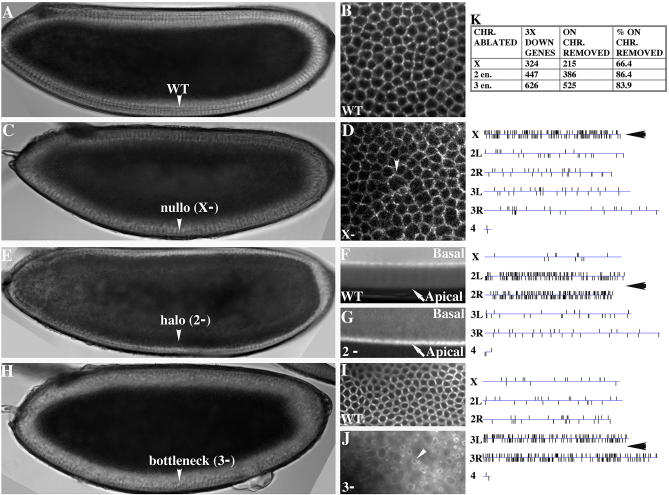
Ablation of the X, 2-Entire, 3-Entire Chromosomes and Identification of Mutant Embryos (A, C, E, and H) Live embryos were imaged using an up-right microscope. Arrowhead indicates the phenotypes associated with each chromosomal ablation. (A) shows a WT embryo. In (C), an X− embryo shows the irregular cellularization front (nullo phenotype) due to the failure to form furrow canals around some nuclei. (E) The 2− embryo developed the characteristic halo phenotype: a dark cytoplasmic halo below the nuclei. (H) The 3− embryo developed the bottleneck phenotype. Nuclei failed to be incorporated in the cellularization front because of the early and uncontrolled contractility of the actin-myosin network. (B, D, F, G, I, and J) Immunostaining using anti-Armadillo (B and D) and anti Myosin-2 antibodies (F, G, I, and J). (B) and (D) show the apical surface, top view, of WT and X− embryos respectively. Arrowhead indicates the irregular conformation of the apical membrane in the X− embryo. (F and G) Optical cross section of WT (F) and 2− (G) embryos stained with anti-myosin-2 antibodies. Note the lack of cell membranes in 2− embryos and failure to localize myosin-2 to the basal side of the cellularization front. (I and J) Top view of WT (I) and 3− (J) embryos stained with anti-myosyn-2 antibodies showing the typical bottleneck phenotype, arrowhead in (J). (K) Table summarizing the results of each chromosomal manipulation. The chromosomal location of down-regulated genes (3-fold, *p* < 0.001) is plotted next to the corresponding embryo. Data were obtained using four biological replicates (ablation of chromosome X and 2) and two biological replicates (ablation of Chromosome 3 entire).

Transcripts expressed at the same level in both collections lie on the diagonal (Figure 1). A large fraction of transcripts deviates from the diagonal and are present at increased or decreased levels in cycle 14. Although mRNAs that increase can be most simply explained by new transcription, the existence of mRNAs whose levels go down suggests that post-transcriptional regulation may be too complex to make judgments about the maternal or zygotic source of a transcript based on measured mRNA levels alone. The decrease or stability in the level of mRNAs may reflect a complex balance between activation and degradation. Even the identification of purely zygotic transcripts can be problematic if the designation is based only on measurements at 2–3 h being above the background at 0–1 h. To address this problem, we undertook a genetic approach based on chromosomal deletions (in embryos that had developed exactly to the same stage) coupled to microarray analysis. We sought to evaluate the traditional interpretation of gene expression measurements, which considers up-regulated transcripts as zygotic, stable transcripts as maternal, and down-regulated transcripts as maternal-degraded (Figure 1B, model).

### Identification of 2L Zygotic Genes

The left arm of the second chromosome represents approximately 20% of the entire genome and is predicted to contain approximately 2,500 open reading frames (BDGP4 annotation; Berkeley *Drosophila* Genome Project, http://www.fruitfly.org/). We compared mRNAs from embryos that lack the left arm of the second chromosome with similarly staged wild-type embryos. Such 2L− embryos can be recognized by their distinctive halo of lipid-rich cortical cytoplasm during cellularization [13], at the precise moment when major zygotic transcription begins. Figure 1C and 1D plot the result of this experiment.

Most mRNAs have similar levels in both collections, and lie on a diagonal (Figure 1). Deviations tend to be located towards the lower left of the diagonal, indicating that certain mRNAs are less abundant in the 2L− collection. There is a small number of mRNAs whose level increases when the 2L arm is removed. Altogether these changes can represent direct and/or indirect responses to the ablation of the 2L arm. Although primary responses must involve genes located on 2L, secondary responses are expected to be randomly distributed on the three major chromosomes. We plotted the chromosomal location of down-regulated and up-regulated genes at different cut-offs (Figure 1E and 1F). At a stringent fold-change cut-off value of ten, the number of deviant mRNAs is small, and all of them represent mRNAs that are less abundant than in wild type. Approximately 90% of these mRNAs are encoded by genes located on 2L, indicating that they are normally supplied by zygotic transcription: removal of 2L eliminates the DNA templates for such transcripts, and the transcripts are not made.

As we decrease the fold-change cut-off, the number of genes that deviate from the diagonal increases. A 2-fold cut-off identifies 378 genes on 2L whose levels depend on the presence of that chromosomal arm in the embryos. A 2-fold difference signifies that at least 50% of the total number of transcripts for each of these genes, present at cycle 14, are derived from zygotic rather than maternal transcription. The observation that even at this cut-off, approximately 60% of down-regulated genes are located on 2L strongly validates this procedure. Indeed, if the observed changes were due to random fluctuations of mRNA levels, such changes would be distributed over the entire genome, and 20% of them would be located on 2L. It should be noted that, in principle, the down-regulated transcripts might also include maternal mRNAs whose stability is regulated by zygotic transcription. However, the enrichment on 2L suggests that this applies to a very small fraction of genes. We therefore classify all down-regulated transcripts (on the deleted arm) as zygotic. The remaining 631 genes that are located on 2L and detected in cycle 14 embryos are not dependent on the presence of the left arm of the second chromosome in the embryo, and must therefore be supplied by maternal transcription. At the 2-fold cut-off, a second class of affected mRNAs appears. These mRNAs are expressed at a higher or lower level than the wild-type controls, and they mapped to other regions of the genome. We interpret these mRNAs as gene products whose levels depend indirectly on the left arm of the second chromosome. We therefore name these genes “secondary targets” of 2L removal. They may be targets of transcription factors encoded on 2L whose expression at cycle 14 depends on the presence of that arm. Alternatively, they might be post-transcriptionally regulated maternal transcripts whose stability or degradation depends on zygotic transcription. In order to discriminate between these mechanisms, we screened the entire genome and determined the maternal and zygotic contribution for each individual gene.

### Whole-Genome Identification of Zygotic Genes and Secondary Targets

Using additional chromosomal rearrangements, we extended the analysis described in detail above for 2L to the rest of the genome, analyzing mRNA populations present in embryos deficient for the X chromosome, the entire second chromosome, or the entire third chromosome. In most cases, hybridizations were performed in quadruplicate using different batches of embryos. Mutant embryos were recognized under a compound microscope based on their specific abnormalities associated with defects in nuclear morphology, in actin-myosin dynamics, and organelle transport: nullo (chromosome X) [14], halo (Chromosome 2) [13], and bottleneck (Chromosome 3) [15]. These three phenotypes appear synchronously as the embryo enters cycle 14, thus allowing a precise staging protocol (Figure 2A–2J). In each case, we were able to identify mRNAs encoded on the deleted chromosome and whose levels depend directly on the presence of that chromosome. These mRNAs thus appear to be predominantly supplied by zygotic transcription (Figure 2K). For all subsequent analyses, we defined the class of down-regulated genes to be those genes with a fold-change of at least three and a *p*-value less than 0.001. In this range, between 60% to 80% of down-regulated genes map to the chromosomes removed. A more stringent cut-off would have increased specificity at the expense of secondary targets. In addition, we have built a simple online database, which provides access to the entire dataset at any (user-specified) fold-change and/or *p*-value cut-off (http://rd.plos.org/pbio.0050117).

A 3-fold cut-off identifies all mRNAs that are at least 67% supplied by zygotic transcription at cycle 14. Combining the data from all four manipulations, we estimate that such zygotically active genes represent about 18% of the genes detectable at cycle 14, i.e., 1,158 genes distributed on all four chromosomes (Table S1). The remaining mRNA species appear to be supplied predominantly by maternal transcription. When looking at the entire dataset, zygotically active genes appear to be uniformly distributed throughout the genome.

Each chromosomal manipulation also identified apparent secondary targets that mapped to other chromosomes. Similar to the results obtained from the 2L− experiments, levels of such mRNAs deviated at most 2- to 3-fold in either the positive or the negative direction from wild-type (WT) mRNAs. To test whether these genes were in fact transcriptional targets of genes on the removed chromosome, we asked whether third chromosomal or X chromosomal genes identified in the 2L chromosomal screen as secondary targets behaved as primary targets when the third chromosome or X was removed. This was true for 62% of down-regulated and 29% of up-regulated genes. Our four experiments identified a total number of 778 secondary targets of which only 28% are zygotic (Table S2). The remaining 72% (563) are mostly maternally supplied. We conclude from these observations that the expression level of most zygotically active genes was not influenced by other loci, and changed significantly only when the chromosome encoding them was removed.

### Maternal Transcripts and Degradation

The identification of 563 non-zygotic mRNAs (Table S3) whose level changed in response to the removal of a specific chromosome must represent post-transcriptional regulation of maternal transcripts. The stability or degradation of these transcripts may be regulated by transcription of certain factors (coding for RNA-binding proteins or regulatory RNAs) on the chromosomes that are removed. In agreement with this interpretation is the observation that ablation of each chromosome or chromosomal arm results in the misregulation of distinct targets. Thus, transcription at multiple loci regulates the stability of distinct maternal transcripts. For example, the degradation of String and Twine, two cell cycle regulators involved in timing the MZT [16], is regulated by zygotic transcription on the X and second chromosomes, respectively (Table S2).

**Figure 3:**
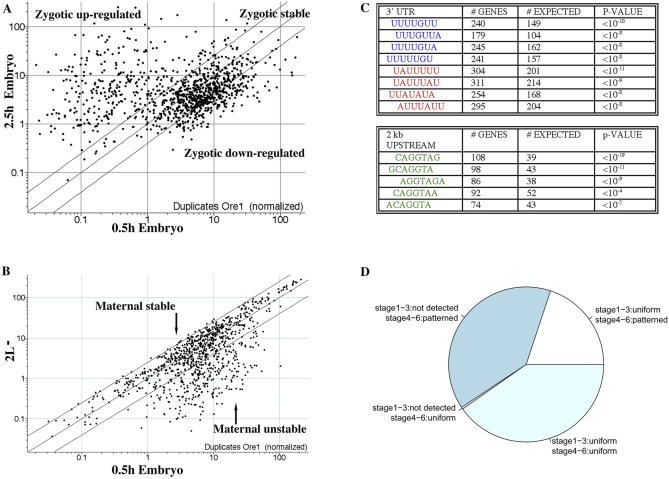
Motif Analysis of Maternal and Zygotic Genes (A) The complete sets of zygotic genes identified in the chromosomal deletion experiments (using the 3-fold cut-off and *p* < 0.001) were plotted in the time-course experiments (two biological replicates for each time point. The scatter plot was generated using the average of the two measurements). This graph demonstrates that zygotic genes can be up-regulated, down-regulated, or remain unchanged during the MZT. Increase in gene expression over time can only identify one third of the zygotic genes. (B) Stability of maternal transcripts: the expression value of 2L genes in freshly fertilized eggs (0.5 h, the average of two biological replicates is plotted) was compared to the expression value of these genes in embryos missing 2L at (2.5 h, average of four biological replicates is plotted). Under this condition, the stability of a specific transcript is solely dependent on its decay kinetics. (C) Motif discovery in the 3′ UTR of maternal unstable transcripts (upper table, the first motif is in red and the second is in blue) and in the 2-kb upstream regions of purely zygotic genes (lower table, in green). The UUGUU sequence resembles the target site for the PUF family of RNA-binding proteins, and the UAUUUAU motif resembles the AU-rich element. (D) BDGP in situ database analysis showing the expression pattern of the different categories of zygotic genes. Stage 1–3 corresponds to maternal stages (0–2 h), and 4–6 to zygotic stages (2–4 h).

Next, we characterized the relative contribution of maternal transcripts to the total cycle 14 expression level of zygotically active genes. We compared the mRNA levels of 1,158 zygotic genes at 0–1 h with that observed at 2–3 h (Figure 3A). In one third of the cases, transcripts could not be detected in 0–1-h embryos, and increased over the 2-h period that follows fertilization. Expression of these genes is therefore purely zygotic: all transcripts detected at cycle 14 are produced by transcription in the embryo itself (Table S4). Almost all of these transcripts (~90%, 300 out of 334) would have been detected as purely zygotic using the simple criterion “absent at 0–1 h–present at 2–3 h” (Table S5). On the other hand, if only this latter criterion had been used, an additional 268 genes would be scored as purely zygotic, even though the level of these transcripts does not change significantly when the chromosome harboring them is removed. When used on its own, the “absent-present” filter may be unreliable because it identifies zygotically active genes by comparing expression measurements at one stage with background levels at another. Our double-filter approach (change in response to the deletion of the DNA template + a “present-absent” value) yields a more stringent and accurate estimate of purely zygotic transcripts.

The remaining two thirds of the 1,158 zygotic genes were present in unfertilized eggs (Table S6). Because the overall levels of theses mRNAs either did not change significantly or decreased between 0 h and 3 h, the dependence of cycle 14 levels on zygotic transcription implies the specific degradation of maternal transcripts before that time. Thus, we conclude that an increase in gene expression over time is not a sufficient criterion to identify zygotic genes.

To follow the stability of maternal transcripts (which is obscured by the presence of newly supplied zygotic transcripts in WT embryos), we compared mRNA levels from early 0–1-h embryos (WT) with mRNA levels from embryos missing each chromosome, hand-selected from the same stock during cycle 14. The initial analysis was restricted to genes on the left arm of the second chromosome (Figure 3B); therefore, all 2L mRNAs detected at either stage must be supplied maternally. The relative change in expression levels between 0–1 h and 2–3 h provides a measure of their stability during that period. Consistent with their strictly maternal source, none of the 1,009 transcripts from 2L increased significantly between 0 h and 3 h. Approximately 65% remained constant, and 35% dropped more than 3-fold. We extended this analysis to the rest of the genome and estimated that of 6,485 total maternally supplied genes, 2,110 (33%) go down significantly by cycle 14. In 646 cases, the maternal degradation was at least in part compensated by zygotic transcription (Table S7). A representative list of maternal and zygotic transcripts known to be degraded or induced during the MZT and detected by our analysis is shown in Table 1.

**Table 1:**
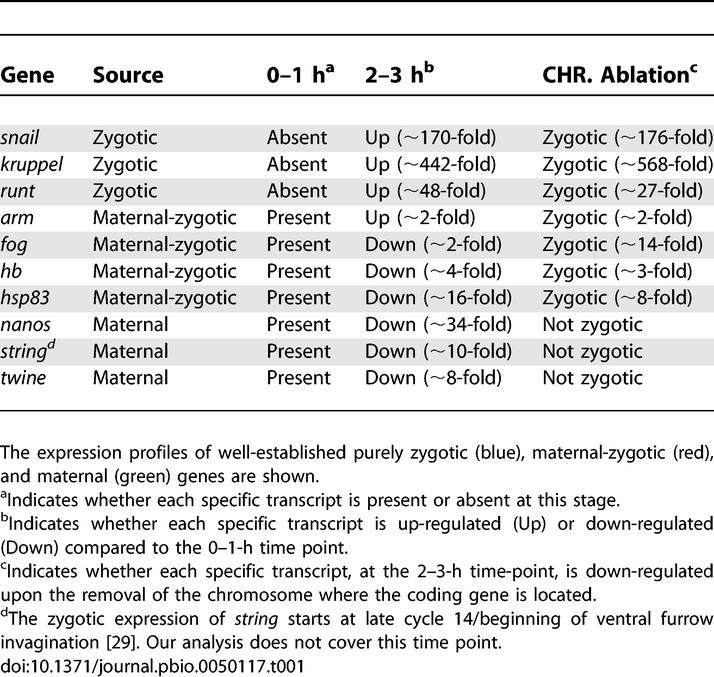
Expression Profile of Known Maternal and Zygotic Genes

### Zygotic Transcription Generates Restricted Pattern of Gene Expression

One third of the zygotic transcripts we have identified are not expressed maternally and can be considered purely zygotic genes. These genes are enriched for transcription factors (“transcription factor activity” Gene Ontology (GO) category, *p* < 10^−9^). This may reflect the necessity of timing the activity of genes regulating the establishment of cell identity during differentiation. The remaining two thirds, those with maternal contribution, are not significantly enriched in any specific functional class. This raises the question as to why the embryo transcribes genes when the corresponding maternal transcripts are present. Two possible scenarios can be envisaged: (1) maternal transcripts must also be supplied by zygotic transcription, because they are degraded very quickly (i.e., they have short half-lives), and (2) zygotic transcription offers some advantages, such as precise spatial patterning, differential processing (e.g., splice variants), or intracellular localization. In the latter scenario, maternal mRNAs would be specifically degraded to ensure that zygotic transcripts are the only source of these genes at cycle 14.

Using data downloaded from the BDGP in situ database, we asked whether the zygotic genes we have identified are expressed in specific patterns at cycle 14 (Figure 3D). A total of 241 of the genes we found to be zygotic are annotated in the database. Of those, 59% were expressed in discrete patterns at cycle 14. Among the total number of genes in the database (1,227), only 27% were patterned at cycle 14. Thus, the zygotic genes we identified are enriched more than 2-fold (*p* < 10^−32^) in patterned expression compared to what would be expected by chance. Even among the 143 zygotic genes that initially had uniform maternal component, 29% evolved to patterned expression by cycle 14, a situation occurring for only 11% of the genes in the entire in situ database (*p* < 10^−8^). Because the expression level of these genes was either stable or decreased during the MZT, we conclude that coupling maternal degradation with zygotic transcription is part of the patterning mechanism. Indeed, one third of the genes expressed in patterns at cycle 14 required both zygotic transcription and degradation of uniform maternal mRNAs (Figure 3D).

### Down-Regulated Maternal Genes and Zygotic Genes Share Distinct Common Motifs

We then asked whether the different categories defined above share common genomic regulatory elements, which could explain the behavior of an individual gene during the MZT.

We first investigated whether down-regulated maternal genes have over-represented motifs in their 3′ UTRs. A total of 1,095 maternal genes with annotated 3′ UTRs decreased in levels significantly between 0–1 h and 2–3 h. As shown in Figure 3C, we found several short sequences that are significantly enriched within these 3′ UTRs, compared to the entire set of annotated 3′ UTRs. None of them matched the 5′ extremity of any of the 78 known microRNAs (miRNAs) in D. melanogaster. A similar conclusion was drawn also by studying transcript stability in unfertilized eggs [17]. The sequences we found can be divided into two families, based on sequence similarity. The first family contains a UUGUU core, which resembles the target site for the PUF family of RNA-binding proteins (whose unique representative in the D. melanogaster genome is Pumilio). To further investigate the role of Pumilio in maternal mRNA degradation, we compared our down-regulated maternal genes to the list of 135 targets of Pumilio in fly embryos [18]. Although these targets do not all contain exactly the same sequence, 118 of the 135 target genes were identified as maternal in our experiments, and 63 of these (53%) were also down-regulated between 0–1 h and 2–3 h. On the other hand, only 23% of maternal genes decreased globally. Therefore, Pumilio targets are very significantly over-represented in maternal down-regulated genes (*p* < 10^−12^). Sequences from the second family match the AU-rich element (canonically defined as UAUUUAU), a known mediator of mRNA degradation [19]. Interestingly, an RNA interference (RNAi)-based screen performed in *Drosophila* S2 cells has suggested that several components of the miRNA processing pathway are required for degradation of AU-rich element–containing mRNAs [20].

We then investigated whether the zygotic transcripts share common DNA regulatory motifs in their upstream regions. We identified a highly over-represented 7-nucleotide–long sequence (CAGGTAG, which from now on we will refer to as the 7mer) and several of its variants within the 2 kilobase (kb) upstream regions of purely zygotic genes (Figure 3C). This motif has been previously identified in the upstream region of *sisterless* A and B, and *Sex-lethal,* three genes involved in sex determination that are expressed early during embryogenesis [21]. A more recent study identified this motif upstream of other genes expressed prior to cycle 14, thus suggesting a more general regulatory function [22].

### CAGGTAG and the Activation of the Zygotic Genome

The results described above are intriguing because the 7mer we found is present upstream of only a fraction of the zygotic genes at cycle 14. Although the major activation of the zygotic genome occurs at cycle 14, earlier reports indicated signs of zygotic transcription as early as cycle 10 when the embryonic DNA is still engaged in fast cycles of S-phases and mitoses without interphases [23]. We therefore asked whether the 7mer represents a general feature of genes expressed prior to cycle 14 and, in general, whether the zygotic genes we have identified are transcribed altogether during cycle 10 or whether different classes of transcripts respond differently to the embryonic cycles and DNA content.

We compared the expression profile of unfertilized eggs, 0–1-h freshly fertilized eggs (pre-pole cell formation, cycles 1–9) and 1–2-h embryos (post-pole cell formation and pre-cellularization, cycles 10–13). No significant change in expression levels was observed between unfertilized eggs and the 0–1-h eggs, indicating that neither transcription nor degradation has occurred (Figure S2). Importantly, in these experiments, we analyzed unfertilized eggs that had been aged for 1 h at most. Therefore, our results do not contradict previous reports describing the degradation of a subset of maternal transcripts in unfertilized eggs [17,24] since, in those studies, unfertilized eggs were aged for longer periods of time, and degradation was observed after 2 h, peaking between 2 and 4 h.

**Figure 4:**
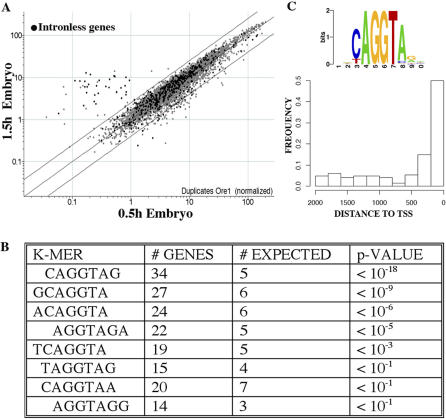
The Activation of the Zygotic Genome Starts from Intronless Genes Sharing the CAGGTAG Motif (A) Expression profile of freshly fertilized eggs (0.5 h, average of two biological replicates) compared to 1.5-h embryos (cycle 10–12, post-pole cell formation; average of two biological replicates). A group of 59 genes is significantly up-regulated at 1.5 h. Intronless genes are shown in black. About 70% of the up-regulated genes do not contain introns. (B and C) Identification of the CAGGTAG motif within the 2-kb upstream regions of the 59 genes up-regulated in (A). (C) Distribution of distances between CAGGTAG occurrences and the transcription start site of the early zygotic genes.

Between the 0–1-h to 1–2-h collections, a single group of 59 genes was significantly up-regulated (Figure 4A). These genes (including *Snail, Zen,* and *Nullo,* see Table S8 for a complete list) are expressed even prior to the gap and pair-rule genes, which in our measurements do not yet show significant increased levels at this time point. Expression of gap and pair-rule transcripts was detected at 2–3 h (Table S1), arguing that their transcripts accumulate with a slower kinetic. When searching for over-represented motifs in the 2-kb upstream regions of these genes, we found the same motif as for the pure zygotic genes, along with other overlapping or slightly distinct variants (Figure 4B). We found 91.5% of the 59 genes have at least one copy of any of these variants, whereas the expectation based on all genes in the genome is 40% (*p* < 10^−15^). Moreover, 28.8% of the 59 genes have four or more non-overlapping copies of these sequences, a situation occurring for only 1.6% of the *Drosophila* genes (*p* < 10^−16^). Thus we conclude that the activation of the zygotic genome starts from genes containing this motif. Interestingly, the occurrences of the 7mer within the 2-kb upstream regions tend to be much closer to the transcription start site than expected by chance (Figure 4C). Finally, we asked whether these genes share some additional features that increase the overall fitness of gene expression prior to cycle 14. We found that 70% of these genes do not contain introns (Figure 4A). Since intronless genes represent only 20% of the *Drosophila* genome, this result suggests an important selective advantage for the transcription of intronless genes in concomitance with fast-cycling nuclei.

### Biochemical Purification of Bicoid Stability Factor as the 7mer Binding Protein

**Figure 5:**
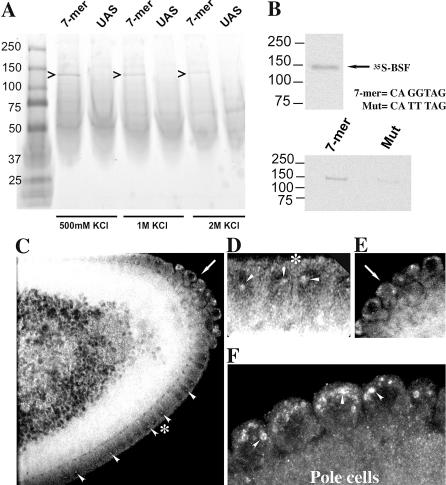
Purification of BSF as the 7mer Interacting Protein (A) Coomassie staining of 7mer interacting protein. Forty grams of embryo extract was prepared as described in Materials and Methods, and subjected to two sequential purification steps. In the first step, proteins were loaded onto an agarose column coupled to the UAS. Unbound proteins were then split into two equal fractions, and each fraction was loaded onto the 7mer or UAS columns. Bound proteins were eluted with a step concentration of KCl and resolved on SDS-Page. Gel shows the results of this last purification step. One major interacting protein bound specifically to the 7mer oligo. Mass spectroscopy sequencing identified this protein as the BSF. (B) BSF binds directly and specifically to the 7mer. BSF was in vitro transcribed and translated in the presence of ^35^S-methionine (^35^S-BSF; upper panel), and the binding to the 7mer or to a mutated sequence (MUT; the two GG at position 3 and 4 were mutated to TT) was tested (lower panel). (C–E) Confocal microscopy analysis of BSF localization in the early embryo. A BSF polyclonal antibody was used to detect the endogenous localization of BSF. BSF was localized to both the cytoplasm and nuclei (arrowheads in [C] and [D]) of the blastoderm epithelium. In pole cells, BSF was localized to cytoplasmic structures, which appeared as dots, arrowhead (F). The arrow in (C) indicates the region which is enlarged in (E). The asterisk (*) in (C) indicate the region which is enlarged in (D).

The identification of a single highly over-represented *cis*-element in the 5′ region of the early zygotic genes suggests the existence of a single *trans*-acting factor involved in timing the activation of the zygotic genome. If such a factor exists, it is most likely maternally provided and loaded into the egg during oogenesis. To identify this factor, we undertook a biochemical approach. We performed sequential DNA affinity chromatography (see Materials and Methods for details) using the 7mer or, as negative control, the upstream activation sequence (UAS) (the consensus binding site of the yeast *trans*-activator GAL4). The result of this experiment is shown in Figure 5A. Only one band was detected in the 7mer elute, and no specific band was detected in the UAS control elute. Mass spectrometry sequencing identified this protein as the Bicoid stability factor (BSF), and Western blotting analysis confirmed this result (unpublished data).

BSF has been previously identified as a Bicoid mRNA binding protein involved in regulating the stability of Bicoid transcripts during oogenesis [25]. Our data suggest an additional transcriptional function for BSF in the embryo, and indeed, the human homolog of BSF has been shown to function as a transcriptional regulator [26].

In order to address the specificity of the 7mer/BSF interaction, BSF was expressed in rabbit reticulocyte in the presence of ^35^S methionine, and the binding to the 7mer or to a mutated oligo (in which the two GG at position 3 and 4 were mutated to TT) was tested. In vitro–synthesized BSF bound directly and specifically to the 7mer, and only background signal was retained on the beads coupled to the mutated oligo (Figure 5B).

Next, we analyzed the subcellular distribution of BSF in the embryo using immunostaining and confocal microscopy imaging. BSF was localized to both the cytoplasm as well as the nuclei of the blastoderm epithelium (Figure 5C and 5D). In the germ cells (pole cells), which at this stage are transcriptionally silent, BSF was retained in cytoplasmic puncta (Figure 5E and 5F). Thus, BSF is differentially compartmentalized between the soma and the germ line, and this compartmentalization may be important to maintain the transcriptional quiescence in the germline.

To test the function of BSF in the early embryo, it is necessary to remove the maternal contribution. (BSF transcripts are maternally provided and the protein is expressed during oogenesis [25].) To perform this experiment, we produced germline clones using a P element insertion that maps in the BSF open reading frame and is homozygous lethal. Flies containing such clones failed to lay eggs, and the ovaries were arrested at a very early stage of development, indicating that BSF is required also during oogenesis. This made it impossible to test the function of BSF in the early embryo. Therefore, we took an alternative approach with the aim to functionally characterize the activity of the 7mer. We considered two possible scenarios. One possibility is that the 7mer may have enhancer activity, sufficient to drive transcription on its own. Alternatively, it may play a permissive role by functioning in a combinatorial fashion with additional factors. To discriminate between these two possibilities, we set up conditions to measure gene expression using an assay based on the UAS/GAL4 system [27].

### CAGGTAG Activates Transcription Prior to Cycle 14

**Figure 6:**
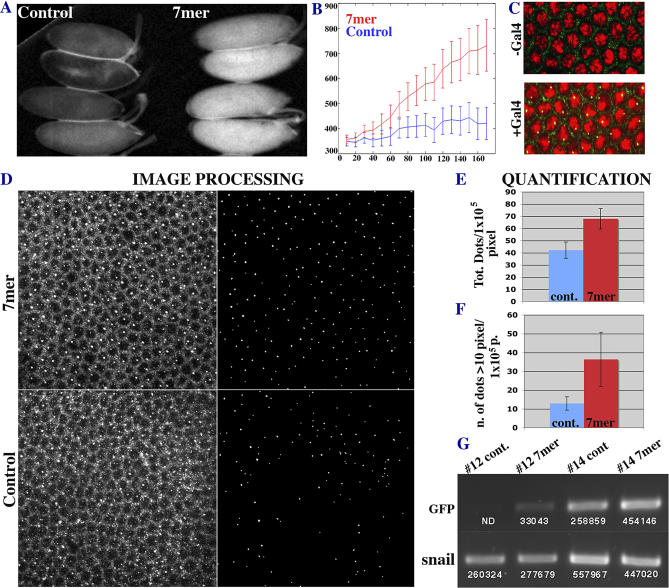
The CAGGTAG Motif Activates Transcription prior to Cycle 14 (A) Transgenic embryos over-expressing GFP with (7mer) or without (control) the 7mer. Five copies of the 7mer were obtained by crossing males carrying the specific transgene to females providing Gal4. GFP expression was monitored using video microscopy. In (A), a single frame is shown. (B) Fluorescent GFP quantification at 20-min intervals. (C) FISH showing the GAL4 dependence of the 7mer-mediated GFP transcription. Only one major dot is observed per nucleus because males carrying the transgene were crossed to females supplying Gal4. (D) FISH analysis of embryos over-expressing GFP with (7mer) or without (control) the 7mer. Images were processed and quantified as described in Materials and Methods. (E and F) Quantification of the number and size of nuclear dots. Results were expressed as number of dots per 1 × 10^5^ pixels. In (F), only dots larger than ten pixels were quantified. (G) RT-PCR quantification. Control and 7mer embryos were harvested at the indicated cycles (12 and 14), and the amount of GFP transcripts quantified using the FluorChem gel documentation system equipped with a CCD camera. The numbers below the bands are the result of this measurement. ND, non-detected.

We generated embryos expressing green fluorescent protein (GFP) under the control of the UAS–heat shock minimal promoter either with or without five copies of the 7mer, and followed GFP expression using video microscopy. GFP was not detected in embryos unless GAL4 was also provided. Strikingly, the presence of the 7mer led to a more than 4-fold increase in the expression of GFP compared to controls (transgene without the 7mer), as shown in Figure 6A and 6B.

Next, we asked how early this stimulatory activity could be detected. We analyzed GFP transcripts using fluorescent in situ hybridization (FISH). This technology allows the visualization of nascent transcripts as they arise from the site of transcription [28]. Because we crossed males carrying the GFP transgene to females providing GAL4, only one chromosome in the embryo is expected to transcribe GFP. In agreement with this prediction, we detected only one major transcription focus, appearing as an individual dot, per nucleus (Figure 6C and 6D). We observed an increase in the number and size of dots at each nuclear division when the 7mer was present (Figure 6D). This difference could be detected as early as cycle 11 (Figure S1). By cycle 14, images are characterized by a high signal-to-noise ratio and showed an approximately 1.7-fold increase in the number of dots per embryo (Figure 6E). Thus, the presence of CAGGTAG increases the number of nuclei that are actually engaged in transcription. Because the size of each dot is also larger (Figure 6F), each dot most likely contains more transcripts. If this interpretation is correct, then it should be possible to quantify this difference by measuring the total amount of GFP transcripts.

**Figure 7:**
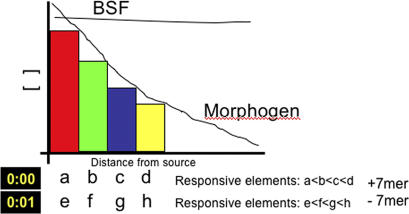
Proposed Mode of Action for the 7mer/BSF Interaction in Timing the Response to a Morphogen Gradient A maternally provided morphogen, for example, Dorsal, activates target gene expression in a concentration-dependent manner, defining distinct developmental units (colored rectangles). Gene *a,* whose enhancer has the lower affinity for this particular morphogen, is expressed only at the higher concentration of the gradient. Gene *d,* whose enhancer has the higher affinity, is expressed also at a lower concentration. The 7mer sequence can time the response to this gradient while maintaining the spatial information. Because the 7-mer alone is not able to activate transcription (Figure 6), it can function as a timer linking the spatial gradient with the temporal regulation. For example, the 7mer sequence contained in the 5′ regulatory region of gene *a* would allow gene *a* to be expressed before gene *e* within the same spatial unit (red). This regulatory mode can operate along both the dorsal-ventral (D-V) and anterior-posterior (A-P) axes because it would not interfere with the positional information encoded in the gradient itself. Indeed the 7mer and its variants are particularly enriched in enhancers bound by Dorsal (D-V) and Bicoid (A-P).

Embryos were harvested either at the stage when the earliest GFP transcripts were expressed (cycle 10 to 13) or at cycle 14. Total RNA was extracted and subjected to reverse-transcription PCR (RT-PCR) (Figure 6G). As a staging control, we followed the expression of *Snail,* a known zygotic gene. We detected 7mer-driven transcription as early as cycle 12. In the absence of this motif, no GFP expression was detected. By cycle 14, we observed a 2-fold increase in GFP expression, which is in agreement with the FISH quantification. As a control, we also followed *Snail* mRNA, which was expressed at similar levels in both conditions, and its expression increased from cycle 12 to 14. Altogether, these results show that the CAGGTAG motif functions as an enhancer that cannot drive transcription on its own (Figure 6C), but can activate expression prior to cycle 14, in combination with a transcriptional activator. In agreement with this result is the finding that CAGGTAG and its variants are particularly abundant in enhancer sequences bound by Dorsal and Bicoid (Table S9), thus suggesting a combinatorial regulatory mode (see model in Figure 7 and Discussion).

## Discussion

The switch from maternal to zygotic control of early embryonic development is characterized by a dramatic remodeling of the transcriptional complexity present in the oocyte. We have genetically identified the relative maternal and zygotic contribution for the expression of each individual gene during the D. melanogaster mid-blastula transition. The criterion we used to identify zygotically expressed genes is strictly based on the direct relationship between the DNA template and the corresponding transcript. The specific phenotype generated upon removal of each chromosomal arm allowed us to collect a synchronous population of embryos just at the stage when the first morphological signs of the zygotic genome become visible. The location of the majority of down-regulated genes to the chromosomal arm that was ablated provided an excellent control for the entire experimental procedure we have undertaken.

In summary, our results indicate that zygotic transcription contributes to approximately 20% of the genes expressed at cycle 14, and as much as 30% of maternal transcripts become unstable during the mid-blastula transition. However, about a third of these transcripts are also supplied by zygotic transcription and, therefore, their expression levels at cycle 14 remain constant. Purely zygotic transcripts represent only a third of the total set of zygotically expressed genes. The remaining two thirds also had a maternal contribution and were present in unfertilized or 0–1-h eggs. The zygotic transcription of such maternally provided genes does not always result in an increase in the total amount of transcript, indicating specific degradation of the maternal counterpart. Thus, a change in gene expression over time is not a sufficient criterion to identify zygotically active genes, nor to measure the stability of maternal transcripts. These results have important implications for the definition of maternal and zygotic genes, and provide a genome-wide analysis, which will be instrumental for a molecular characterization of the MZT.

Our analysis shows that purely zygotic transcripts are enriched in transcription factors. Providing these genes through zygotic transcription, which in turn is related to the number of nuclei, ensures that correct number of cells is assigned to a specific fate and, ultimately, the establishment of the correct body proportion. The execution of a specific differentiation program represents a more complex problem, in that it involves the adjustment of the expression of genes involved in basic cell function. Therefore, these genes must be expressed during oogenesis, to support oocyte development, and their activity modulated through zygotic transcription.

Our data argue that zygotic transcription allows a large fraction of ubiquitously expressed maternal mRNAs to be expressed again in localized patterns at the blastoderm stage. The generation of these patterns involves the degradation of the maternal transcript and the corresponding activation of zygotic transcription. This result is in agreement with previous studies on individual genes (e.g., the maternally provided Cdc25 phosphatase string and the maternal-zygotic transcription factor hunchback), which were reported to undergo a similar MZT [29,30]. Altogether our analysis is consistent with the proposal that zygotic transcription provides the spatial precision at which important regulatory genes must be expressed during the differentiation of the developing embryo. Therefore, our results can be used in combination with chromosomal deficiency screening to quickly identify gene function at the mid-blastula transition by reducing the number of candidate genes contained in each deficiency to zygotic-dependent expressed genes. We have used this approach to identify the bearded genes as the zygotic genes regulating Notch signaling during mesoectoderm specification [31].

In addition, our results show that zygotic transcription is required for the degradation of a distinct subset of maternal transcripts. Because these transcripts do not share any statistically enriched common regulatory sequence and because each chromosomal manipulation targeted distinct transcripts, we propose that multiple zygotic activities must be involved in this regulation. One possibility is that the zygotic expression of miRNAs might be part of this mechanism. For example, in zebrafish, mir-430 was shown to control the degradation of a pool of maternal transcripts [32]. Although we have not detected any significant over-representation of known miRNA target sites in our data, the involvement of miRNAs in specific pathways can be tested once zygotic control regions have been more closely defined. We also identified maternal mRNAs that are degraded and not replenished by zygotic transcription. A fraction of these genes share sequences within their 3′ UTR, which resemble the known target site for the Pumilio RNA-binding protein. We showed that a very large fraction of the Pumilio targets in the embryo are indeed degraded during the transition from maternal to zygotic stages. Pumilio was first identified as an inhibitor of translation controlling posterior fate by promoting deadenylation of hunchback mRNA [33,34]. Our results suggest that Pumilio might also promote degradation of mRNA targets as shown for the yeast homolog Puf3 [35].

The transition from a silent to a transcriptionally active genome is one of the most dramatic events in a developing embryo and is subject to regulation at multiple steps. We have identified the CAGGTAG motif (and its variants) as an important player in this transition. We identified the BSF as the factor binding to this motif in the early embryo.

BSF has been previously identified as a protein binding to the 3′ UTR of Bicoid mRNA and involved in regulating Bicoid transcript stability during oogenesis [25]. However, the precise biochemical function of BSF is unknown. Mutation of this gene causes lethality, and induction of homozygous germline clones arrests oogenesis (see Results). Thus, BSF must have additional function other than the regulation of Bicoid transcripts because Bicoid itself is not required for oogenesis, and zygotic mutants are viable.

Interestingly, the human ortholog of BSF, the leucine-rich protein LRP130, has been shown to bind to a *cis*-regulatory sequence in the 5′ proximal region of the MDR1 gene and to act as a transcriptional regulator [26,36]. Although we could not genetically test the function of BSF, our analysis suggests that BSF is not able to drive transcription on its own, but must act in a combinatorial fashion. Indeed, we found that CAGGTAG and its variants are particularly abundant in enhancer sequences bound by Dorsal and Bicoid (Table S9). These transcription factors activate the expression of their target genes in a concentration-dependent manner and define distinct developmental units along the dorsal-ventral (D-V) and anterior-posterior (A-P) axes [37,38]. Interestingly, only a subset of the known targets for these transcription factors have this element, even within the same spatial unit. For example, in the D-V patterning system controlled by the Dorsal gradient, CAGGTAG is found in *Snail* and *Tom,* but not *Neuralized*. Both *Snail* and *Tom* are expressed prior to *Neuralized* (*Snail* and *Tom* are among the 59 genes induced during cycle 10–11) and must act before *Neuralized* to precisely position Notch signaling at the mesoderm–mesoectoderm boundary [31]. The inability of CAGGTAG to drive transcription on its own makes it an ideal timer, which links spatial gradient with temporal regulation (see model, Figure 7).

In conclusion, the experiments described in this work will be instrumental for studying the activation of the zygotic genome in other animals and for guiding embryonic stem cell differentiation. In the mouse, zygotic transcription begins by the two-cell stage, and a large number of maternal mRNAs persist beyond this stage [4]. The contribution of zygotic transcription to the expression of these mRNAs is still unknown. Further, the activation of the mouse genome is characterized by discrete wave-like patterns of gene expression. Similarly, the activation of the *Drosophila* genome starts with a battery of 59 transcripts induced from cycles 10–11 to cycle 14. Interestingly, 70% of these genes do not contain introns, and they all encode small proteins. This result is in agreement with two previous studies showing that genes transcribed early in development tend to be unusually short [39] and that the presence of a long intron (19 kb) limits the expression of the *knirps*-related gene *(knrl)* early during *Drosophila* development [40]. The use of intronless genes might reflect the necessity of expressing regulatory genes requiring a minimal response time. Intriguingly, many short genes in the human genome have been implicated in anti-sense–mediated gene regulation [41]. In the *Drosophila* embryo, the expression of intronless genes occurs when the nuclei are still engaged in rapid phases of DNA duplication and mitoses. Because the nuclear membrane is required for the assembly of the splicing machinery, the selection of intronless genes might ensure the production of functional transcripts in concomitance with nuclear divisions.

## Materials and Methods

### Genetics and flies stocks.

WT flies were Oregon-R; all stocks were maintained by standard methods at 18 °C, unless otherwise specified. Transgenic embryos over-expressing GFP were generated using P element–mediated germline transformation using w^1118^ as the recipient host. GFP was ectopically expressed using the 7merUAS-GFP line 4 (III) or UAS-GFP line 10 (II) and the matαTub-Gal4VP16 67C;15 driver. Embryos were collected at room temperature. The halo deficiency is Df(2L)dpp[s7-dp35] 21F1–3;22F1–2 and was balanced over CyO [13]. Compound chromosomes: embryos with no X chromosome were obtained by crossing attached-X/Y females to X/Y males. The stock used was C(1) *DX, y f* [7]. The compound II chromosomes RM(2L); RM(2R) = C(2)*v,* in which the two left arms or the two right arms segregate together, were used to generate 2L− and 2R− embryos [8]. The compound II C(2) *EN* and compound III C(3) *EN* st1, cu1, es, stocks (Bloomington 2974 and 1117) were used to generate embryos deficient for the entire second and third chromosome, respectively. The BSF P element insertion used for the germline clone experiments was obtained from the Szged stock center: FRT-l(2)SH1181. This P element insertion has been mapped to the BSF cDNA and is homozygous lethal. Moreover, it failed to complement a deficiency covering the BSF genomic locus *Df*(2L)M36F-S5.

### Microarray.

Embryos were collected on apple juice-agar plates, visually staged under a compound microscope, dechorionated for 2 min in 5.25% sodium hypochlorite (Austin's bleach), washed in water, and then frozen in 1 ml of heptane (Sigma, http://www.sigmaaldrich.com) using a dry ice/ethanol chamber. Total RNA was extracted with TRIzol (Invitrogen, http://www.invitrogen.com), and 10 mg of RNA (approximately 100 embryos) was used to synthesize complementary RNA (cRNA) according to the Affymetrix protocol. Each array (standard format: *Drosophila* genome 1, Affymetrix) was probed with 15 mg of biotinilated cRNA for 16 h in a 45 °C oven. Arrays were washed and stained using the GeneChip Fluidics Station (Affymetrix, http://www.affymetrix.com) according to the EukGE-WS2 protocol. Subsequently the arrays were scanned with an Argon-ion laser scanner (Affymetrix). Graphs were generated using the GeneSpring software (Silicon Genetics/Agilent, http://www.chem.agilent.com). CEL files were loaded in R (http://www.r-project.org), and analyzed using the Bioconductor package [42]. The analysis followed the “Golden Spike” methodology described in [43]. Briefly, Present, Marginal, or Absent flags were computed using the *MAS* approach, with default parameters. Within a group of replicates of the same condition, a probe set was identified as present if it had a Present flag (not Marginal) in more than 50% of the replicates. Intensity values were corrected using the *MAS* approach, and arrays were normalized with respect to each other at the probe level using a *loess* function. PM probe intensities were corrected for unspecific hybridization using the MM probes and the *MAS* approach. Expression summaries were generated using the *MedianPolish* method. A second *loess* correction was subsequently applied to the expression summaries. Two-tailed statistical tests were performed on the replicates using the regularized *t*-test approach implemented in CyberT [44], with normalization constant set to five times the minimum number of replicates among the two populations analyzed, and the window size set to 101. Further set operations (unions and intersections) were performed at the probe set level, using custom R functions.

### Motif finding.

Probe sets from the DrosGenome1 Affymetrix platform were matched to the latest version of the D. melanogaster cDNAs downloaded from ENSEMBL [45], as of August 2005 (BDGP4). Briefly, each probe was matched to the cDNA set using BLAST, with seed length 11, and e-value threshold 1e-5. Only probes with 100% identity to their match were considered. A probe set was considered to match a transcript if at least ten (out of 14) of its probes matched the transcript. Transcript identifiers were collapsed into their corresponding gene identifier. Two-kilobase upstream regions (up to the TSS when available, or to the ATG codon otherwise) and 3′ UTRs were downloaded from ENSEMBL, also as of August 2005. Only the longest 3′ UTR for a same gene was retained. Motif finding within a set of genes of interest was performed using an exhaustive *k*-mer enumeration and over-representation approach. Briefly, each 7mer was considered in turn (6-, 8-, 9- and gapped *k*-mers were also examined, but yielded negative or similar results). The set of genes of interest (e.g., early zygotic genes) that have at least one copy of the 7mer was determined, with size denoted as *s_1_*. The set of all *Drosophila* genes that have at least one copy of the same 7mer, in their upstream (or 3′ UTR) region was then determined, with size denoted as *s_2_*. The size of the overlap between the two sets was then calculated, and denoted as *i*. A *p*-value of the size of overlap being greater than *i* (representing the over-representation of the *k*-mer within *s_1_*) was calculated using the cumulative hypergeometric distribution. All 7mers were sorted based on their *p*-value, corrected for multiple testing using the Bonferroni correction. Only 7mers with corrected *p*-values lower than 0.05 were considered significant and used for further analysis. Motifs derived from 3′ UTRs were systematically compared to the seed regions within the sequences of the 78 known *Drosophila* miRNAs, downloaded in August 2005 from the miRNA registry [46].

### Purification of BSF as the 7mer interacting protein.

The 0–3-h. embryos were harvested, dechorionated for 2 min, washed in phosphate buffer saline (PBS) 0.1% Triton X-100, and frozen at −80 °C. Forty grams of packed embryos were diluted in 50 ml of Lysis buffer (25 mM Hepes [pH7.6]; 100 mM KCl; 12.5 mM MgCl2; 1 mM DTT; 10% glycerol; 500 μg of Poly DI-DC (Sigma), 1X Protease Inhibitor cocktail (P8340; Sigma) and homogenized in a Dounce tissue grinder at 4 °C. Total lysate was spun down for 5 min at 1,000 rpm in order to pellet the nuclei. Total membranes were spun down by ultracentrifugation in a 70 Ti rotor (Beckman, http://www.bioscience.com) at 40,000 rpm for 1 h at 4 °C. The supernatant from this centrifugation step was incubated for 5 h at 4 °C with 500 μl of immobilized streptavidin agarose beads (Pierce 20347; http://www.piercenet.com) coupled to 1 mg of double-stranded UAS oligo (51 bases long, three tandem copies of the GAL4 binding site) biotinylated at the 5′ end of the upper strand with a BioTEG (5′-BioTEGCGGAGTACTGTCCTCCGCGGAGTACTGTCCTCCGCGGAGTACTGTCCTCCG) and equilibrated in 100 mM KCl; 25 mM Hepes (pH 7.4). The flow true from this purification step was then loaded onto either 500 μl of beads containing the 7mer sequence (49 bases long, seven copies of the CAGGTAG repeat; (CAGGTAGCAGGTAGCAGGTAGCAGGTAG CAGGTAGCAGGTAGCAGGTAG) or onto 500 μl of UAS oligo beads prepared as described above and incubated for an additional 4 h at 4 °C. Columns were washed with 20 ml of wash buffer (100 mM KCl, 25 mM Hepes [pH 7.4]). Bound proteins were eluted in three fractions containing 5 mM EDTA, 50 mM Hepes (pH 7.4), 1 mM DTT and increasing concentration of KCl: first, 500 mM KCl; second, 1 M KCl, and third, 2 M KCl. Each elution step was performed by incubating the beads for 10 min in a total volume of 500 μl at room temperature. Each eluted fraction was concentrated and desalted on a centrifugal filter 3-kDa cut-off (Millipore CAT NO: 42403; http://www.millipore.com) and loaded on SDS-Page. The bound protein was cut and sequenced according to standard mass spectroscopy procedure.

### In vitro binding of BSF to the 7mer sequence.

BSF cDNA was in vitro transcribed and translated in the presence of ^35^S methionine using the T7-TnT Quick-coupled transcription/translation system (Promega CAT NO: TM045; http://www.promega.com). We incubalted 60 μl of radio-labeled BSF for 3 h at 4 °C with 50 μl of beads containing the 7mer sequence or a mutated sequence (CATTTAG) prepared as described above, in a total volume of 500-μl buffer containing 25 mM Hepes (pH 7.4), 100 mM KCl, 12.5 mM MgCl; 1mM DTT 1-mg/ml BSA, and 2.5-mg poly dI-dC. After four washes in 1 ml of buffer containing 25 mM Hepes (pH 7.4), 100 mM KCl, and 1 mM DTT, bound proteins were eluted in 80 μl of 1.5 M NaCl, 50 mM Hepes (pH7.4), 5 mM EDTA, and 1 mM DTT. Then 30 μl of the eluted proteins were loaded on SDS-page and processed for autoradiography.

### CAGGTAG analysis.

All variants of the CAGGTAG motif with a significant *p*-value were retained, and aligned manually. All occurrences of these variants within the 59 early genes were determined, along with their position with respect to the TSS; overlapping occurrences were removed. The remaining occurrences were used to form a weight matrix, whose motif logo was drawn using WebLogo [47]. All *Drosophila* enhancers in the Redfly database [48] were downloaded, as of March 2006. All enhancers were searched for the above variants of CAGGTAG, but only those that had at least two copies were retained. The density of CAGGTAG variants per kilobase was calculated for all retained enhancers, and enhancers were sorted according to this density.

### In situ database analysis.

All available in situ data and corresponding annotation keywords were downloaded from the BDGP in situ database [49], as of August 2005. Only genes with detectable expression at stages 4–6 were retained for further analysis, resulting in a set of 1,227 genes. Based on the annotated keywords, gene expression at each stage was classified as uniformly expressed, patterned, or not detected.

### Cloning and RT-PCR quantification.

Two complementary oligos each containing five copies of the CAGGTAG repeats flanked by a SphI restriction site at both the 5′ and 3′ extremities were in vitro synthesized, annealed, cut with SphI, and cloned into the SphI site of the pUasT-EGFP (1075) destination vector (Gateway; Invitrogen). Recombinant plasmids were identified using PCR and sequencing for correct orientation. For the quantification of GFP transcripts, 80 embryos were visually staged under a regular dissecting microscope, hand selected, and total RNA extracted using Trizol (Sigma). For each time point, 80 ng of total RNA was used in a one-step RT-PCR reaction (Invitrogen Superscript One-step RT-PCR). Twenty-five cycles of amplification at TM 55 °C ensured a linear range of amplification. The primers used were: GFP-F: ACGTAAACGGCCACAAGTTC; GFP-R: TGCTCAGGTAGTGGTTGTCG; Snail-F: CGGAACCGAAACGTGACTAT; Snail-R: GCGGTAGTTTTTGGCATGAT. Amplified reactions were loaded on a 1.2% agarose gel, stained with ethidium bromide, and quantified using a gel documentation system equipped with a CCD camera (FluorChem; Alpha Innotech, http://www.alphainnotech.com).

### Immunostaining.

Embryos were dechorionated for 2 min in bleach and fixed in 4% paraformaldehyde (Electron Microscopy Science, http://www.emsdiasum.com/microscopy/)-heptane for 20 min. Embryos were blocked in 10% bovine serum albumin (BSA) in PBS, 0.1% Triton-X-100 (Sigma) for 1 h. Primary antibodies were incubated in PBS containing 5% BSA and 0.1% Tween-20 for 12 h at 4 °C. Embryos were washed five times in PBS, 0.1% Triton-X-100, and incubated with secondary antibodies for 2 h at room temperature in PBS, 5% BSA, 0.1% Tween-20. After five washes in PBS, stained embryos were mounted in Aquapolymount (Polyscience, http://www.polyscience.com). Antibodies: mouse anti-Armadillo (1:50); rabbit anti-myosin-2 (1:500). Secondary antibodies were Alexa-488 conjugated (1:500; Molecular Probes). For the detection of BSF, embryos were heat fixed and the goat anti-BSF antibody (P.M. Macdonald) was used at 1:500 dilution as described in [25].

### FISH and image analysis.

FITC-UTP–labeled RNA antisense probes against GFP were generated by cutting the pBlue script II EGFP(N) plasmids with KpnI and transcribing with the T7 polymerase. Embryos were dechorionated for 2 min in bleach and fixed in 4% paraformaldehyde (Electron Microscopy Science)-heptane for 20 min. Hybridization was performed in 50% formamide (Roche, http://www.roche.com), 5× SSC (Sigma), 1× Denharts (Sigma), 1% (w/v) blocking agent (Roche), 10-mg/ml yeast tRNA (Sigma), 0.1% Triton X-100 (Sigma), 0.1% CHAPS (Sigma) for 16 h at 56 °C. Thereafter, embryos were blocked in 2× Western blocking reagent (Roche), PBS, 0.1% Triton-X-100 (Sigma) for 1 h at room temperature and incubated with mouse anti-FITC (Roche) 1:400 incubated in blocking buffer for 3 h. Embryos were washed five times in PBS, 0.1% Triton-X-100, and incubated with anti-mouse Alexa488-conjugated secondary antibodies (Molecular Probes) used at 1:500 dilution for 1.5 h at room temperature in blocking buffer. After five washes, nuclei were stained using Toto-3 dye (Molecular Probes), incubated in 70% glycerol for 30 min, and mounted in Aquapolymount (Polyscience). Images were acquired with a PerkinElmer spinning disk confocal microscope equipped with a 40× (numerical aperture [na] 1.3) oil immersion objective (Nikon, http://www.nikon.com) using the Ultra VIEW imaging system (PerkinElmer, http://www.perkinelmer.com). Serial sections were collected, and the number and size of nuclear dots was quantified as follows. Image analysis was performed using MATLAB v7 and the Image Processing Toolkit (http://www.mathworks.com). Pixel intensities were first linearly stretched using the *imadjust* and *stretchlim* functions. Whole-embryo boundaries were located within the images using a thresholding of 0.20, a morphological opening with disk of radius 1 (pixel) for removal of small artifacts, a filling of type “hole” with radius 4, a morphological opening with disk of radius 20 for removing larger artifacts, followed by a morphological closing with disk of radius 1 for obtaining sharper boundaries. The resulting single object (representing the embryo) was used as a mask for further analysis. Intensities within the masks were then thresholded using a 0.99 cut-off. A morphological opening operation was used to remove small artifacts, using a disk of radius 1. The remaining objects (spots) were extracted from the image using the *bwlabel* function, and their number and area calculated using the *regionprops* function. For the GFP quantification, masks for each of the ten WT and nine 7mer embryos within the time course were drawn using Photoshop (Adobe Systems, http://www.adobe.com), from the first frame in the movie. These masks were subsequently used to study only the regions of interests (single embryos) in later frames. For each frame in the time course, the median pixel intensity with each embryo boundary was calculated. Average median intensities and corresponding standard deviations across the ten WT and nine 7mer embryos were finally calculated.

## Supporting Information

Figure S1 FISH of GFP Transcripts during Cycle 11 to 14 Embryos were fixed and processed for FISH analysis. Stained embryos were imaged using confocal microscopy. Each panel corresponds to a stack of ten sections (0.5 μm/section). (A) to (D) correspond to embryos in cycles 11, 12, 13, and 14, respectively, expressing GFP under the 7mer sequence. (E) to (H) correspond to embryos in cycles 11, 12, 13, and 14, respectively, expressing GFP without the 7mer sequence.
(2.1 MB JPG)

Figure S2 Microarray Measurements Comparing Unfertilized Eggs and 0–1-h Embryos Unfertilized eggs and 0–1-h embryos (prior to pole cell formation) were collected and their expression profile analyzed using microarrays. The majority of transcripts were present at similar levels in both collections. None of the changes were statistically significant, indicating the lack of both transcription and degradation.
(621 KB JPG)

Table S1 List of Zygotic Genes: Primary List of mRNAs that were down-regulated at least 3-fold compared to similarly staged WT embryos in cycle 14, upon ablation of each chromosome, and that were located on the chromosome that was removed. These are the primary zygotic genes and include both purely zygotic as well as maternal+zygotic transcripts. The difference in expression level indicates the relative maternal and zygotic contribution to the expression level at cycle 14. Table key: “affy_probe_set_id” is the Affimetrix probe identifier; “fbgn for the probe set” corresponds to the fbgn number provided by Affymetrix; the “CGid” was determined using the mapping to the *Drosophila* genome I; “Chr. Location” indicates the chromosomal location where the down-regulated genes were located; fold-change refers to the fold change of down-regulated genes in mutant embryos compared to WT embryos in cycle14; the *p*-value for all measurements was less than 0.001.
(86 KB PDF)

Table S2 List of Secondary Targets: Zygotic List of mRNAs that were down-regulated or up-regulated at least 3-fold, compared to similarly staged WT embryos in cycle 14, upon ablation of each chromosomes, and that, if down-regulated, were not located on the chromosome that was removed. However, these transcripts were identified as primary zygotic transcripts when the chromosome harboring them was removed. These are zygotic transcripts whose expression is controlled by other zygotic genes. Table key: “affy_probe_set_id” is the Affymetrix probe identifier; “fbgn for the probe set” corresponds to the fbgn number provided by Affymetrix; the “CGid” was determined using the mapping to the *Drosophila* genome I; “Chr. Location” indicates the chromosomal location where the down-regulated genes were located; fold-change refers to the fold change of down-regulated genes in mutant embryos compared to WT embryos in cycle14; the *p*-value for all measurements was less than 0.001.
(31 KB PDF)

Table S3 List of Secondary Targets: Non-zygotic. List of mRNAs that were down-regulated or up-regulated at least 3-fold, compared to similarly staged WT embryos in cycle 14, upon ablation of each chromosome, and that were not identified as primary zygotic transcripts when the chromosome harboring them was removed. Therefore, these are maternal transcripts whose expression or stability is controlled by other zygotic genes. Table key: “affy_probe_set_id” is the Affymetrix probe identifier; “fbgn for the probe set” corresponds to the fbgn number provided by Affymetrix; the “CGid” was determined using the mapping to the *Drosophila* genome I; “Chr. Location” indicates the chromosomal location where the down-regulated genes were located; fold-change refers to the fold change of down-regulated or up-regulated genes in mutant embryos compared to WT embryos in cycle 14; the *p*-value for all measurements was less than 0.001.
(31 KB PDF)

Table S4 List of Purely Zygotic Genes (Identified Using Chromosome Deletion + Time Course) List of mRNAs that were not expressed at 0–1 h, were down-regulated at least 3-fold compared to similarly staged WT embryos in cycle 14, upon ablation of each chromosome, and that were located on the chromosome that was removed. Therefore, these are purely zygotic transcripts and represent a subgroup of Table S1. This last table includes both purely zygotic as well as maternal+zygotic. Table key: “affy_probe_set_id” is the Affymetrix probe identifier; “fbgn for the probe set” corresponds to the fbgn number provided by Affymetrix; the “CGid” was determined using the mapping to the *Drosophila* genome I; “Chr. Location” indicates the chromosomal location where the down-regulated genes were located; fold-change refers to the fold change of down-regulated genes in mutant embryos compared to WT embryos in cycle 14; the *p*-value for all measurements was less than 0.001.
(41 KB PDF)

Table S5 List of Purely Zygotic Genes (Identified Using Only Time Course) List of mRNAs which were not expressed at 0–1 h, and were expressed at 2–3 h. These are purely zygotic transcripts identified using solely the time course measurement. Note that this list contains 300 out of the 334 transcripts identified in Table S4 (see text for more details). Table key: “affy_probe_set_id” is the Affymetrix probe identifier; “fbgn for the probe set” corresponds to the fbgn number provided by Affymetrix; the “CGid” was determined using the mapping to the *Drosophila* genome I; “Chr. Location” indicates the chromosomal location where the up-regulated genes were located; fold-change refers to the fold change of up-regulated genes in 2–3-h embryos compared to 0–1-h embryos; the *p*-value for all measurements was less than 0.001.
(60 KB PDF)

Table S6 List of Maternal-Zygotic Transcripts List of mRNAs that were down-regulated at least 3-fold, compared to similarly staged WT embryos in cycle 14, upon ablation of each chromosome, and that were expressed also during maternal stages (0–1-h collection). These are zygotic transcripts, which also had maternal contribution. Table key: “affy_probe_set_id” is the Affymetrix probe identifier; “fbgn for the probe set” corresponds to the fbgn number provided by Affymetrix; the “CGid” was determined using the mapping to the *Drosophila* genome I; “Chr. Location” indicates the chromosomal location where the down-regulated genes were located; fold-change refers to the fold change of down-regulated genes compared to WT embryos in cycle 14; the *p*-value for all measurements was less than 0.001.
(70 KB DOC)

Table S7 List of Zygotic Transcripts Down-Regulated from 0–1 to 2–3 h List of mRNAs that were down-regulated at least 3-fold during the maternal-to-zygotic transition (from 0–1 h to 2–3 h) and that were down-regulated at least 3-fold upon ablation of the chromosomes on which they mapped compared to similarly staged cycle 14 WT embryos. Table key: “affy_probe_set_id” is the Affymetrix probe identifier; “fbgn for the probe set” corresponds to the fbgn number provided by Affymetrix; the “CGid” was determined using the mapping to the *Drosophila* genome I; “Chr. Location” indicates the chromosomal location where the down-regulated genes were located; fold-change refers to the fold change of down-regulated genes compared to WT embryos in cycle 14; the *p*-value for all measurements was less than 0.001.
(59 KB PDF)

Table S8 List of Early Zygotic Genes (1–2 h) List of mRNAs that were not expressed at 0–1 h, and were up-regulated at 1–2 h. These are the earliest 59 zygotic expressed genes. Table key: “affy_probe_set_id” is the Affymetrix probe identifier; “fbgn for the probe set” corresponds to the fbgn number provided by Affymetrix; the “CGid” was determined using the mapping to the *Drosophila* genome I; “Chr. Location” indicates the chromosomal location where the up-regulated genes were located; fold-change refers to the fold change of up-regulated genes in 1–2-h embryos compared to 0–1 h embryos; the *p*-value for all measurements was less than 0.001.
(22 KB PDF)

Table S9 CAGGTAG (and Variants) Occurrences within Experimentally Verified *cis*-Regulatory Modules Densities of CAGGTAG (and variants) occurrences within experimentally verified *cis*-regulatory modules (CRMs), from the RedFly database. CRMs with the highest density of CAGGTAG/variants are bound by Bcd or Dl. Table key: “# of CAGGTAG/kb” indicates the density of CAGGTAG(+variants)/kb, “CRM length (bp)” is the length of *cis*-regulatory modules in base pairs; “# of CAGGTAG” is the counted number of CAGGTAG(+variants) in the CRM; “CRM name (RedFly)” indicates the nomenclature of each specific CRM according to the Regulatory Element Database for *Drosophila* (REDfly)
(32 KB PDF)

## Abbreviations

2L−: lacking the left arm of the second chromosome
BDGP: Berkeley *Drosophila* Genome Project
BSF: Bicoid stability factor
FISH: fluorescent in situ hybridization
GFP: green fluorescent protein
kb: kilobase
miRNA: microRNA
MZT: maternal-to-zygotic transition
RT-PCR: reverse-transcription PCR
UAS: upstream activation sequence
WT: wild-type
