# Correction: Unmasking Activation of the Zygotic Genome Using Chromosomal Deletions in the Drosophila Embryo

**DOI:** 10.1371/journal.pbio.0050195

**Published:** 2007-07-17

**Authors:** Stefano De Renzis, Olivier Elemento, Saeed Tavazoie, Eric Wieschaus

In *PLoS Biology*, volume 5, issue 5: doi: 10.1371/journal.pbio.0050117

We would like to acknowledge that the 5‚ cis-regulatory element referred to in our paper as 7-mer had been previously named TAGteam (John R. ten Bosch, Joseph A. Benavides, Thomas W. Cline, [see reference number 22 in our paper]). The authors provide functional evidence that this element functions as an enhancer during the maternal-to-zygotic transition, a conclusion that is supported by our results but that we did not discuss in the text. We also apologize to the Cline lab for not having cited the 2003 *Genetics* paper where the authors first demonstrated functional activity of this cis-element for the transcriptional activation of *scute/sisB*.

Wrischnik LA, Timmer JR, Megna LA, Cline TW (2003) Recruitment of the proneural gene scute to the Drosophila sex-determination pathway. Genetics 165: 2007–2027.

