# Correction: Unmasking Activation of the Zygotic Genome Using Chromosomal Deletions in the Drosophila Embryo

**DOI:** 10.1371/journal.pbio.0050213

**Published:** 2007-08-14

**Authors:** Stefano De Renzis, Olivier Elemento, Saeed Tavazoie, Eric Wieschaus

In *PLoS Biology,* volume 5, issue 5: doi:10.1371/journal.pbio.0050117

An incorrect version of Table S3 was published. The correct Table S3 is given here; the legend remains the same.

Table S3 List of Secondary Targets: Non-zygotic. (51 KB PDF)

