# Supplementary material for: Unmasking Activation of the Zygotic Genome Using Chromosomal Deletions in the Drosophila Embryo
Source: PLoS Biol. 2007 Apr 24;5(5):e117. doi: 10.1371/journal.pbio.0050117 (PMC1854917; doi:10.1371/journal.pbio.0050117)
Supplement: Supplementary file 2 [file pbio.0050117.st002.pdf]

Table S2: Zygotic Secondary Targets

affy_probe_set_id	fbgn for the probe set	CG id	Chr.Location	fold-changes	p-value
142230_at	FBgn0004583	CG4114	chr2L	-21.86488017	3.58E-08
142879_at	FBgn0010583	CG3727	chr2L	-7.70958031	1.45E-12
143064_at	FBgn0000058	CG1374	chr2L	-68.21710309	1.78E-11
143285_at	FBgn0002970	CG6246	chr2L	-66.51985661	4.44E-12
143356_at	FBgn0003448	CG3956	chr2L	-176.796814	0
143414_at	FBgn0004009	CG4889	chr2L	-101.5154803	3.68E-12
143555_at	FBgn0005616	CG3241	chr2L	-23.50063859	2.01E-07
143743_at	FBgn0014015	CG33529	chr2L	-19.87204014	1.24E-09
143754_at	FBgn0014179	CG12245	chr2L	-20.09897655	1.26E-07
143774_at	FBgn0015000	CG3469	chr2L	-9.863871721	1.53E-05
143820_at	FBgn0015567	CG4260	chr2L	-5.117495744	3.12E-05
144171_at	FBgn0027104	CG10393	chr2L	-11.62780678	7.03E-10
144234_at	FBgn0028538	CG7578	chr2L	-38.2410798	2.92E-11
145509_at	FBgn0031215	CG11450	chr2L	-194.4009665	2.92E-13
145753_at	FBgn0031595	CG15427	chr2L	-27.21476857	1.15E-14
145830_at	FBgn0031718	CG14014	chr2L	-92.99444277	1.11E-16
145894_at	FBgn0031806	CG9506	chr2L	-35.85515304	0
146171_at	FBgn0032270	CG31721	chr2L	-3.844919239	3.08E-06
146224_at	FBgn0032335	CG14915	chr2L	-280.5706315	0
146263_at	FBgn0032390	CG16969	chr2L	-21.25715629	2.45E-08
146397_at	FBgn0032629	CG15138	chr2L	-27.16551712	1.71E-09
146436_at	FBgn0032682	CG10176	chr2L	-26.70650372	7.57E-13
151713_at	LD44245.3prime-hit	CG18024	chr2L	-137.4555654	5.21E-13
152162_at	FBgn0028523	CG5888	chr2L	-119.311262	1.11E-16
152360_at	FBgn0000675	CG5686	chr2L	-5.62519481	5.61E-09
152508_at	FBgn0031897	CG13784	chr2L	-14.75847568	0
152549_at	FBgn0017551	CG10800	chr2L	-10.67303809	5.55E-09
152899_at	FBgn0016930	CG4551	chr2L	-3.053617416	2.21E-05
153299_at	FBgn0032451	CG12292	chr2L	-28.0218796	5.88E-09
153624_at	FBgn0032694	CG15162	chr2L	-71.53164055	2.33E-07
153717_at	FBgn0031435	CG9883	chr2L	-11.78651761	0
153728_at	FBgn0012037	CG8827	chr2L	-24.57894558	1.10E-08
153835_at	FBgn0004893	CG10021	chr2L	-70.76091155	1.29E-09
153866_at	FBgn0031303	CG4710	chr2L	-70.67737187	2.48E-11
153943_at	FBgn0031250	CG11907	chr2L	-11.55528308	4.43E-06
154673_at	FBgn0020616	CG3423	chr2L	-25.75732516	2.32E-10
154983_at	FBgn0031621	CG15634	chr2L	-1111.115953	0
141307_at	FBgn0034225	CG4827	chr2R	-7.627187151	2.44E-11
141373_at	FBgn0032993	CG18140	chr2h	-8.314321757	7.74E-06
141565_at	FBgn0003701	CG5785	chr2R	-4.047116361	6.47E-10
142143_at	FBgn0016078	CG8804	chr2R	-6.861927625	1.39E-05
142212_at	FBgn0034393	CG15095	chr2R	-140.9026915	2.19E-13
142479_at	FBgn0034342	CG17533	chr2R	-6.175429195	1.56E-05
142966_at	FBgn0003731	CG10079	chr2R	-7.275055966	1.05E-10
143153_at	FBgn0000606	CG2328	chr2R	-19.51925474	0
143209_at	FBgn0001325	CG3340	chr2R	-568.8701576	1.11E-16
143299_at	FBgn0003067	CG17725	chr2R	-99.63089187	0
143303_at	FBgn0003082	CG11205	chr2R	-4.423103843	1.11E-05
143584_at	FBgn0010109	CG8704	chr2R	-9.238923672	2.24E-13
143631_at	FBgn0010638	CG10130	chr2R	-9.78635983	4.44E-16
143665_at	FBgn0011592	CG8581	chr2R	-7.690035282	1.11E-16
143907_at	FBgn0019968	CG8183	chr2R	-34.11820734	5.46E-09

145681_at	FBgn0031477	CG31694	chr2L	-3.47574739	4.64E-08
146921_at	FBgn0033483	CG12919	chr2R	-149.9073743	7.57E-14
147023_at	FBgn0033638	CG9005	chr2R	-4.339045242	2.82E-05
147037_at	FBgn0033652	CG12443	chr2R	-56.34372714	1.74E-10
147079_at	FBgn0033714	CG8487	chr2R	-13.96701084	8.66E-07
147342_at	FBgn0034170	CG33456	chr2R	-14.34332525	3.75E-09
147434_at	FBgn0034334	CG17522	chr2R	-64.06891216	1.03E-11
147701_at	FBgn0034764	CG9952	chr2R	-63.0642467	4.55E-15
151573_at	GM02568.3prime-hit	CG13344	chr2R	-5.854669202	6.30E-07
151623_at	LD09801.3prime-hit	CG12424	chr2R	-26.15549925	6.02E-09
151656_at	LD19406.3prime-hit	CG8166	chr2R	-22.94749362	2.53E-08
151902_at	FBgn0034772	CG30092	chr2R	-25.89218206	1.07E-09
152444_at	FBgn0011236	CG5575	chr2R	-15.69390656	2.42E-08
152515_at	FBgn0003074	CG8251	chr2R	-4.721422994	2.19E-05
152598_at	FBgn0034335	CG5164	chr2R	-48.50174532	0
152832_at	FBgn0033632	CG30023	chr2R	-26.29022732	1.34E-14
153087_at	FBgn0034479	CG8654	chr2R	-19.91179235	5.33E-09
153146_at	FBgn0034220	CG6530	chr2R	-7.670900287	7.15E-05
153473_at	FBgn0033181	CG1624	chr2R	-4.597114461	1.24E-12
153848_at	FBgn0033855	CG13333	chr2R	-2891.247622	0
153867_at	FBgn0000577	CG9015	chr2R	-29.16784949	7.08E-09
154213_at	FBgn0033992	CG11798	chr2R	-12.80328376	1.36E-13
154254_at	FBgn0034650	CG10318	chr2R	-18.43770033	2.44E-08
154837_at	FBgn0034275	CG5002	chr2R	-26.24882987	9.94E-08
141268_at	FBgn0035394	CG32486	chr3L	-11.73736104	6.94E-11
141276_at	FBgn0022359	CG4649	chr3R	-3.32115313	1.11E-06
141301_at	FBgn0004885	CG6863	chr3R	-16.49258895	4.50E-05
141377_at	FBgn0011653	CG15002	chr3L	-15.56644063	3.21E-09
141518_at	FBgn0003117	CG3978	chr3R	-23.38285374	5.83E-05
141685_at	FBgn0037515	CG3066	chr3R	-3.380538267	0.00012286
141687_at	FBgn0003507	CG3992	chr3R	-239.2030269	1.12E-08
142179_s_at	FBgn0028495	CG18616	chr3R	-3.093283088	0.00013091
142672_at	FBgn0039099	CG10157	chr3R	-7.546758784	1.22E-11
142718_at	FBgn0027500	CG17286	chr3L	-4.21122399	7.46E-08
142813_at	FBgn0020299	CG31317	chr3R	-27.9350415	2.13E-05
142830_at	FBgn0001316	CG17046	chr3L	-43.88160514	8.12E-06
143122_at	FBgn0000439	CG2189	chr3R	-11.88134256	0.00013494
143147_at	FBgn0000565	CG7266	chr3L	-5.630391093	8.02E-06
143164_at	FBgn0000723	CG8874	chr3R	-102.4288205	5.94E-07
143169_at	FBgn0001077	CG2047	chr3R	-36.17992251	2.13E-13
143176_at	FBgn0001099	CG33088	chr3L	-8.215797454	1.35E-09
143180_at	FBgn0001138	CG9656	chr3R	-64.16065542	2.77E-06
143198_at	FBgn0001233	CG1242	chr3L	-8.193411573	6.14E-13
143199_at	FBgn0001235	CG17117	chr3R	-69.29834537	2.07E-06
143208_at	FBgn0001320	CG4717	chr3L	-75.52773852	3.23E-07
143251_at	FBgn0002629	CG6099	chr3R	-74.55269175	1.82E-10
143254_at	FBgn0002633	CG8361	chr3R	-8.472839892	1.24E-09
143293_at	FBgn0003041	CG8114	chr3L	-71.79245411	5.11E-15
143472_at	FBgn0004449	CG5723	chr3L	-20.21509276	7.01E-05
143496_at	FBgn0004595	CG17228	chr3R	-13.4958963	3.22E-06
143500_at	FBgn0004635	CG1004	chr3L	-99.22404082	5.95E-07
143718_at	FBgn0013755	CG7960	chr3L	-47.53495444	2.42E-13
143756_at	FBgn0014343	CG10601	chr3L	-21.12092178	7.18E-05
144063_at	FBgn0025456	CG5413	chr3R	-7.144856462	2.23E-08
148012_at	FBgn0035287	CG13937	chr3L	-17.93583074	3.40E-08

148164_at	FBgn0035525	CG11582	chr3L	-33.86816382	4.88E-07
148194_at	FBgn0035569	CG15876	chr3L	-153.8288826	4.81E-12
148195_at	FBgn0035570	CG13712	chr3L	-97.13298727	1.90E-07
148197_at	FBgn0035572	CG13711	chr3L	-258.0545234	7.42E-11
148369_at	FBgn0035830	CG8209	chr3L	-3.43717647	7.09E-07
148440_at	FBgn0035954	CG5093	chr3L	-146.5864319	1.10E-07
148493_at	FBgn0036048	CG6736	chr3L	-546.2940716	9.29E-11
148557_at	FBgn0036135	CG7636	chr3L	-20.43201644	3.28E-08
148703_at	FBgn0036350	CG14111	chr3L	-18.76276765	5.43E-11
148822_at	FBgn0036530	CG7554	chr3L	-12.94944266	4.50E-11
148988_at	FBgn0036779	CG13695	chr3L	-183.5974947	2.17E-09
148995_at	FBgn0036787	CG4306	chr3L	-61.23248837	4.22E-06
149011_at	FBgn0036811	CG6884	chr3L	-3.084806092	4.40E-06
149132_at	FBgn0037000	CG5130	chr3L	-4.031341633	4.88E-07
149180_at	FBgn0037085	CG7204	chr3L	-93.30677677	1.93E-07
149474_at	FBgn0037548	CG7900	chr3R	-12.2538246	9.02E-05
149620_at	FBgn0037797	CG12420	chr3R	-68.3599417	1.05E-14
149633_at	FBgn0037819	CG14688	chr3R	-6.074089249	4.16E-09
149706_at	FBgn0037947	CG6930	chr3R	-60.54731375	2.12E-06
149906_i_at	FBgn0038243	CG8066	chr3R	-20.63428482	7.74E-05
149907_r_at	FBgn0038243	CG8066	chr3R	-17.20630164	0.00012374
149913_at	FBgn0038255	CG7552	chr3R	-30.72095616	6.92E-07
149940_at	FBgn0038302	CG4210	chr3R	-15.62019155	3.56E-05
149974_at	FBgn0038363	CG18505	chr3R	-3.323550805	2.14E-05
150016_at	FBgn0038430	CG14895	chr3R	-7.077932511	1.11E-09
150269_at	FBgn0038813	CG5206	chr3R	-41.1006593	9.19E-06
150331_at	FBgn0038908	CG6637	chr3R	-4.815931963	1.22E-07
150558_at	FBgn0039288	CG13653	chr3R	-70.79593765	1.20E-06
151087_at	FBgn0040602	CG14545	chr3R	-6.520780077	2.21E-05
151178_at	FBgn0040701	NA	chr3L	-30.30358234	4.63E-13
151179_at	FBgn0040702	CG13713	chr3L	-129.8324067	5.48E-09
151287_at	FBgn0040813	CG11051	chr3L	-6.672907061	1.45E-06
151599_at	HL01913.3prime-hit	CG1225	chr3L	-494.5261049	8.61E-10
151681_at	LD29569.3prime-hit	CG32306	chr3L	-13.68170051	2.16E-12
151695_at	LD33458.3prime-hit	NA	chr3R	-12.53753657	1.30E-05
151758_at	SD09884.3prime-hit	CG11100	chr3L	-13.35591637	8.29E-05
151806_at	FBgn0036732	CG7571	chr3L	-74.37033148	5.96E-07
151807_at	FBgn0039537	CG5590	chr3R	-3.488746293	6.33E-06
151904_at	FBgn0027598	CG31012	chr3R	-22.02426964	7.74E-09
151918_s_at	FBgn0027594	CG2086	chr3L	-13.15174488	7.63E-09
152037_at	FBgn0035889	CG33057/CG7	chr3L	-50.01951083	2.36E-06
152200_at	FBgn0003308	CG7642	chr3R	-127.3359556	8.73E-08
152253_at	FBgn0037216	CG12582	chr3R	-47.40099279	3.61E-07
152563_at	FBgn0038803	CG5191	chr3R	-5.614024679	2.92E-08
152735_at	FBgn0036136	CG6233	chr3L	-25.34204813	5.63E-07
152914_at	FBgn0036684	CG3764	chr3L	-22.75693077	5.60E-09
153349_at	FBgn0038608	CG7670	chr3R	-17.31819777	0.00017446
153576_at	FBgn0036182	CG6084	chr3L	-3.390947312	6.47E-07
153731_at	FBgn0001225	CG4183	chr3L	-6.870225113	4.27E-08
153758_at	FBgn0000338	CG17894	chr3R	-6.318289404	5.36E-06
153779_at	FBgn0000368	CG6383	chr3R	-43.60121676	3.77E-14
154146_at	FBgn0001323	CG4761	chr3L	-9.77012675	0.00012517
154388_at	FBgn0010340	CG9852	chr3R	-13.21585042	0.00029383
154482_at	FBgn0037556	CG9636	chr3R	-3.555287975	0.00081625
154602_at	FBgn0010389	CG7223	chr3R	-88.48448839	1.72E-06

154821_at	FBgn0037699	CG8147	chr3R	-229.9680718	2.83E-09
154880_at	FBgn0000071	CG2198	chr3R	-494.7113856	2.66E-14
154929_at	FBgn0039286	CG11849	chr3R	-2961.521527	3.38E-11
154936_at	FBgn0038184	CG9285	chr3R	-4.193070081	4.81E-08
155012_at	FBgn0038538	CG7660	chr3R	-11.78415037	3.99E-11
155072_at	FBgn0035745	CG32386	chr3L	-48.46981845	3.58E-09
155107_at	FBgn0022349	CG1910	chr3R	-3.659083699	1.18E-07
141713_at	FBgn0004643	CG9900	chrX	-144.0344433	8.33E-08
142777_at	FBgn0004655	CG3707	chrX	-10.39996027	4.74E-05
142824_at	FBgn0022768	CG2984	chrX	-3.349488096	0.00058077
143133_at	FBgn0000499	CG18361	chrX	-7.04322649	5.99E-09
143135_at	FBgn0000524	CG3929	chrX	-14.96306892	2.68E-05
143175_at	FBgn0001092	CG8893	chrX	-5.885673966	8.18E-07
143185_at	FBgn0001150	CG7952	chrX	-37.61589914	8.08E-09
143237_at	FBgn0002561	CG3839	chrX	-36.9976416	8.15E-06
143359_at	FBgn0003463	CG9224	chrX	-10.82949421	0.00020881
143373_at	FBgn0003659	CG33070	chrX	-11.1950905	0.00013737
143386_at	FBgn0003865	CG1502	chrX	-6.992435599	4.64E-08
143471_at	FBgn0004446	CG32659	chrX	-3.437929002	0.00024739
143841_at	FBgn0015773	CG18657	chrX	-254.6798885	1.55E-08
144094_at	FBgn0025800	CG2262	chrX	-67.07769398	1.18E-06
144430_at	FBgn0029650	CG3939	chrX	-3.374287903	1.47E-05
144619_at	FBgn0029907	CG4547	chrX	-74.86507578	9.50E-07
144630_s_at	FBgn0029920	CG4575/CG79	chrX	-32.49527683	8.40E-08
144635_at	FBgn0029927	CG14430	chrX	-46.18386319	3.30E-11
144639_at	FBgn0029931	CG14427	chrX	-52.79865677	7.74E-11
144730_at	FBgn0030090	CG12664	chrX	-4.516312778	0.00014123
144784_at	FBgn0030180	CG12102	chrX	-3.950749049	5.58E-05
145025_at	FBgn0030510	CG12177	chrX	-5.683622221	0.00015319
145095_at	FBgn0030602	CG32594	chrX	-29.49693294	1.36E-05
145222_at	FBgn0030807	CG13000	chrX	-492.3378039	9.89E-10
145379_at	FBgn0031036	CG14220	chrX	-18.46751952	3.27E-05
151326_at	FBgn0040853	CG32666	chrX	-18.84576169	8.78E-05
151389_at	FBgn0040920	CG32736	chrX	-6.019656659	0.00069322
151495_at	GH06717.3prime-hit	CG2930	chrX	-25.2528433	1.03E-06
151504_i_at	GH10316.complete-hit	CG3620	chrX	-4.372962594	5.68E-05
151603_at	HL05860.3prime-hit	CG7053	chrX	-43.42567078	1.46E-10
151610_at	LD03548.3prime-hit	CG3026	chrX	-3.045337843	0.00012442
151660_at	LD21771.3prime-hit	CG3429	chrX	-5.248301022	0.00013059
151863_at	FBgn0003300	CG1849	chrX	-27.31367272	2.21E-05
152338_at	FBgn0003053	CG12212	chrX	-107.8236687	1.24E-07
153044_at	FBgn0015565	CG2913	chrX	-9.588917164	1.11E-08
153222_at	FBgn0030274	CG1908	chrX	-65.10946786	9.62E-07
153230_at	FBgn0024251	CG32527	chrX	-46.48266827	1.02E-05
153332_at	FBgn0029958	CG12151	chrX	-28.54075047	2.14E-05
153647_at	FBgn0014133	CG1822	chrX	-3.677547286	4.41E-05
153751_at	FBgn0030003	CG2116	chrX	-5.241721318	3.53E-06
154137_at	FBgn0010315	CG9096	chrX	-3.590181537	3.54E-05
154221_at	FBgn0011761	CG4193	chrX	-31.18374974	3.90E-11
154261_at	FBgn0030973	CG7332	chrX	-5.37043855	2.30E-06
154424_at	FBgn0028469	CG8062	chrX	-3.503806404	0.00050928
154558_at	FBgn0026080	CG6121	chrX	-6.766100342	6.52E-08
154638_at	FBgn0031062	CG14230	chrX	-4.052445284	2.95E-05
