# Supplementary material for: Unmasking Activation of the Zygotic Genome Using Chromosomal Deletions in the Drosophila Embryo
Source: PLoS Biol. 2007 Apr 24;5(5):e117. doi: 10.1371/journal.pbio.0050117 (PMC1854917; doi:10.1371/journal.pbio.0050117)
Supplement: Supplementary file 3 [file pbio.0050117.st003.pdf]

# Table S2: Zygotic Secondary Targets

| affy_probe_set_id | fbgn for the probe set | CG id | Chr.Location | fold-changes | p-value |
|---|---|---|---|---|---|
| 142230_at | FBgn0004583 | CG4114 | chr2L | -21.86488017 | 3.58E-08 |
| 142879_at | FBgn0010583 | CG3727 | chr2L | -7.70958031 | 1.45E-12 |
| 143064_at | FBgn0000058 | CG1374 | chr2L | -68.21710309 | 1.78E-11 |
| 143285_at | FBgn0002970 | CG6246 | chr2L | -66.51985661 | 4.44E-12 |
| 143356_at | FBgn0003448 | CG3956 | chr2L | -176.796814 | 0 |
| 143414_at | FBgn0004009 | CG4889 | chr2L | -101.5154803 | 3.68E-12 |
| 143555_at | FBgn0005616 | CG3241 | chr2L | -23.50063859 | 2.01E-07 |
| 143743_at | FBgn0014015 | CG33529 | chr2L | -19.87204014 | 1.24E-09 |
| 143754_at | FBgn0014179 | CG12245 | chr2L | -20.09897655 | 1.26E-07 |
| 143774_at | FBgn0015000 | CG3469 | chr2L | -9.863871721 | 1.53E-05 |
| 143820_at | FBgn0015567 | CG4260 | chr2L | -5.117495744 | 3.12E-05 |
| 144171_at | FBgn0027104 | CG10393 | chr2L | -11.62780678 | 7.03E-10 |
| 144234_at | FBgn0028538 | CG7578 | chr2L | -38.2410798 | 2.92E-11 |
| 145509_at | FBgn0031215 | CG11450 | chr2L | -194.4009665 | 2.92E-13 |
| 145753_at | FBgn0031595 | CG15427 | chr2L | -27.21476857 | 1.15E-14 |
| 145830_at | FBgn0031718 | CG14014 | chr2L | -92.99444277 | 1.11E-16 |
| 145894_at | FBgn0031806 | CG9506 | chr2L | -35.85515304 | 0 |
| 146171_at | FBgn0032270 | CG31721 | chr2L | -3.844919239 | 3.08E-06 |
| 146224_at | FBgn0032335 | CG14915 | chr2L | -280.5706315 | 0 |
| 146263_at | FBgn0032390 | CG16969 | chr2L | -21.25715629 | 2.45E-08 |
| 146397_at | FBgn0032629 | CG15138 | chr2L | -27.16551712 | 1.71E-09 |
| 146436_at | FBgn0032682 | CG10176 | chr2L | -26.70650372 | 7.57E-13 |
| 151713_at | LD44245.3prime-hit | CG18024 | chr2L | -137.4555654 | 5.21E-13 |
| 152162_at | FBgn0028523 | CG5888 | chr2L | -119.311262 | 1.11E-16 |
| 152360_at | FBgn0000675 | CG5686 | chr2L | -5.62519481 | 5.61E-09 |
| 152508_at | FBgn0031897 | CG13784 | chr2L | -14.75847568 | 0 |
| 152549_at | FBgn0017551 | CG10800 | chr2L | -10.67303809 | 5.55E-09 |
| 152899_at | FBgn0016930 | CG4551 | chr2L | -3.053617416 | 2.21E-05 |
| 153299_at | FBgn0032451 | CG12292 | chr2L | -28.0218796 | 5.88E-09 |
| 153624_at | FBgn0032694 | CG15162 | chr2L | -71.53164055 | 2.33E-07 |
| 153717_at | FBgn0031435 | CG9883 | chr2L | -11.78651761 | 0 |
| 153728_at | FBgn0012037 | CG8827 | chr2L | -24.57894558 | 1.10E-08 |
| 153835_at | FBgn0004893 | CG10021 | chr2L | -70.76091155 | 1.29E-09 |
| 153866_at | FBgn0031303 | CG4710 | chr2L | -70.67737187 | 2.48E-11 |
| 153943_at | FBgn0031250 | CG11907 | chr2L | -11.55528308 | 4.43E-06 |
| 154673_at | FBgn0020616 | CG3423 | chr2L | -25.75732516 | 2.32E-10 |
| 154983_at | FBgn0031621 | CG15634 | chr2L | -1111.115953 | 0 |
| 141307_at | FBgn0034225 | CG4827 | chr2R | -7.627187151 | 2.44E-11 |
| 141373_at | FBgn0032993 | CG18140 | chr2h | -8.314321757 | 7.74E-06 |
| 141565_at | FBgn0003701 | CG5785 | chr2R | -4.047116361 | 6.47E-10 |
| 142143_at | FBgn0016078 | CG8804 | chr2R | -6.861927625 | 1.39E-05 |
| 142212_at | FBgn0034393 | CG15095 | chr2R | -140.9026915 | 2.19E-13 |
| 142479_at | FBgn0034342 | CG17533 | chr2R | -6.175429195 | 1.56E-05 |
| 142966_at | FBgn0003731 | CG10079 | chr2R | -7.275055966 | 1.05E-10 |
| 143153_at | FBgn0000606 | CG2328 | chr2R | -19.51925474 | 0 |
| 143209_at | FBgn0001325 | CG3340 | chr2R | -568.8701576 | 1.11E-16 |
| 143299_at | FBgn0003067 | CG17725 | chr2R | -99.63089187 | 0 |
| 143303_at | FBgn0003082 | CG11205 | chr2R | -4.423103843 | 1.11E-05 |
| 143584_at | FBgn0010109 | CG8704 | chr2R | -9.238923672 | 2.24E-13 |
| 143631_at | FBgn0010638 | CG10130 | chr2R | -9.78635983 | 4.44E-16 |
| 143665_at | FBgn0011592 | CG8581 | chr2R | -7.690035282 | 1.11E-16 |
| 143907_at | FBgn0019968 | CG8183 | chr2R | -34.11820734 | 5.46E-09 |

| | | | | | |
|---|---|---|---|---|---|
| 145681_at | FBgn0031477 | CG31694 | chr2L | -3.47574739 | 4.64E-08 |
| 146921_at | FBgn0033483 | CG12919 | chr2R | -149.9073743 | 7.57E-14 |
| 147023_at | FBgn0033638 | CG9005 | chr2R | -4.339045242 | 2.82E-05 |
| 147037_at | FBgn0033652 | CG12443 | chr2R | -56.34372714 | 1.74E-10 |
| 147079_at | FBgn0033714 | CG8487 | chr2R | -13.96701084 | 8.66E-07 |
| 147342_at | FBgn0034170 | CG33456 | chr2R | -14.34332525 | 3.75E-09 |
| 147434_at | FBgn0034334 | CG17522 | chr2R | -64.06891216 | 1.03E-11 |
| 147701_at | FBgn0034764 | CG9952 | chr2R | -63.0642467 | 4.55E-15 |
| 151573_at | GM02568.3prime-hit | CG13344 | chr2R | -5.854669202 | 6.30E-07 |
| 151623_at | LD09801.3prime-hit | CG12424 | chr2R | -26.15549925 | 6.02E-09 |
| 151656_at | LD19406.3prime-hit | CG8166 | chr2R | -22.94749362 | 2.53E-08 |
| 151902_at | FBgn0034772 | CG30092 | chr2R | -25.89218206 | 1.07E-09 |
| 152444_at | FBgn0011236 | CG5575 | chr2R | -15.69390656 | 2.42E-08 |
| 152515_at | FBgn0003074 | CG8251 | chr2R | -4.721422994 | 2.19E-05 |
| 152598_at | FBgn0034335 | CG5164 | chr2R | -48.50174532 | 0 |
| 152832_at | FBgn0033632 | CG30023 | chr2R | -26.29022732 | 1.34E-14 |
| 153087_at | FBgn0034479 | CG8654 | chr2R | -19.91179235 | 5.33E-09 |
| 153146_at | FBgn0034220 | CG6530 | chr2R | -7.670900287 | 7.15E-05 |
| 153473_at | FBgn0033181 | CG1624 | chr2R | -4.597114461 | 1.24E-12 |
| 153848_at | FBgn0033855 | CG13333 | chr2R | -2891.247622 | 0 |
| 153867_at | FBgn0000577 | CG9015 | chr2R | -29.16784949 | 7.08E-09 |
| 154213_at | FBgn0033992 | CG11798 | chr2R | -12.80328376 | 1.36E-13 |
| 154254_at | FBgn0034650 | CG10318 | chr2R | -18.43770033 | 2.44E-08 |
| 154837_at | FBgn0034275 | CG5002 | chr2R | -26.24882987 | 9.94E-08 |
| 141268_at | FBgn0035394 | CG32486 | chr3L | -11.73736104 | 6.94E-11 |
| 141276_at | FBgn0022359 | CG4649 | chr3R | -3.32115313 | 1.11E-06 |
| 141301_at | FBgn0004885 | CG6863 | chr3R | -16.49258895 | 4.50E-05 |
| 141377_at | FBgn0011653 | CG15002 | chr3L | -15.56644063 | 3.21E-09 |
| 141518_at | FBgn0003117 | CG3978 | chr3R | -23.38285374 | 5.83E-05 |
| 141685_at | FBgn0037515 | CG3066 | chr3R | -3.380538267 | 0.00012286 |
| 141687_at | FBgn0003507 | CG3992 | chr3R | -239.2030269 | 1.12E-08 |
| 142179_s_at | FBgn0028495 | CG18616 | chr3R | -3.093283088 | 0.00013091 |
| 142672_at | FBgn0039099 | CG10157 | chr3R | -7.546758784 | 1.22E-11 |
| 142718_at | FBgn0027500 | CG17286 | chr3L | -4.21122399 | 7.46E-08 |
| 142813_at | FBgn0020299 | CG31317 | chr3R | -27.9350415 | 2.13E-05 |
| 142830_at | FBgn0001316 | CG17046 | chr3L | -43.88160514 | 8.12E-06 |
| 143122_at | FBgn0000439 | CG2189 | chr3R | -11.88134256 | 0.00013494 |
| 143147_at | FBgn0000565 | CG7266 | chr3L | -5.630391093 | 8.02E-06 |
| 143164_at | FBgn0000723 | CG8874 | chr3R | -102.4288205 | 5.94E-07 |
| 143169_at | FBgn0001077 | CG2047 | chr3R | -36.17992251 | 2.13E-13 |
| 143176_at | FBgn0001099 | CG33088 | chr3L | -8.215797454 | 1.35E-09 |
| 143180_at | FBgn0001138 | CG9656 | chr3R | -64.16065542 | 2.77E-06 |
| 143198_at | FBgn0001233 | CG1242 | chr3L | -8.193411573 | 6.14E-13 |
| 143199_at | FBgn0001235 | CG17117 | chr3R | -69.29834537 | 2.07E-06 |
| 143208_at | FBgn0001320 | CG4717 | chr3L | -75.52773852 | 3.23E-07 |
| 143251_at | FBgn0002629 | CG6099 | chr3R | -74.55269175 | 1.82E-10 |
| 143254_at | FBgn0002633 | CG8361 | chr3R | -8.472839892 | 1.24E-09 |
| 143293_at | FBgn0003041 | CG8114 | chr3L | -71.79245411 | 5.11E-15 |
| 143472_at | FBgn0004449 | CG5723 | chr3L | -20.21509276 | 7.01E-05 |
| 143496_at | FBgn0004595 | CG17228 | chr3R | -13.4958963 | 3.22E-06 |
| 143500_at | FBgn0004635 | CG1004 | chr3L | -99.22404082 | 5.95E-07 |
| 143718_at | FBgn0013755 | CG7960 | chr3L | -47.53495444 | 2.42E-13 |
| 143756_at | FBgn0014343 | CG10601 | chr3L | -21.12092178 | 7.18E-05 |
| 144063_at | FBgn0025456 | CG5413 | chr3R | -7.144856462 | 2.23E-08 |
| 148012_at | FBgn0035287 | CG13937 | chr3L | -17.93583074 | 3.40E-08 |

| | | | | | |
|---|---|---|---|---|---|
| 148164_at | FBgn0035525 | CG11582 | chr3L | -33.86816382 | 4.88E-07 |
| 148194_at | FBgn0035569 | CG15876 | chr3L | -153.8288826 | 4.81E-12 |
| 148195_at | FBgn0035570 | CG13712 | chr3L | -97.13298727 | 1.90E-07 |
| 148197_at | FBgn0035572 | CG13711 | chr3L | -258.0545234 | 7.42E-11 |
| 148369_at | FBgn0035830 | CG8209 | chr3L | -3.43717647 | 7.09E-07 |
| 148440_at | FBgn0035954 | CG5093 | chr3L | -146.5864319 | 1.10E-07 |
| 148493_at | FBgn0036048 | CG6736 | chr3L | -546.2940716 | 9.29E-11 |
| 148557_at | FBgn0036135 | CG7636 | chr3L | -20.43201644 | 3.28E-08 |
| 148703_at | FBgn0036350 | CG14111 | chr3L | -18.76276765 | 5.43E-11 |
| 148822_at | FBgn0036530 | CG7554 | chr3L | -12.94944266 | 4.50E-11 |
| 148988_at | FBgn0036779 | CG13695 | chr3L | -183.5974947 | 2.17E-09 |
| 148995_at | FBgn0036787 | CG4306 | chr3L | -61.23248837 | 4.22E-06 |
| 149011_at | FBgn0036811 | CG6884 | chr3L | -3.084806092 | 4.40E-06 |
| 149132_at | FBgn0037000 | CG5130 | chr3L | -4.031341633 | 4.88E-07 |
| 149180_at | FBgn0037085 | CG7204 | chr3L | -93.30677677 | 1.93E-07 |
| 149474_at | FBgn0037548 | CG7900 | chr3R | -12.2538246 | 9.02E-05 |
| 149620_at | FBgn0037797 | CG12420 | chr3R | -68.3599417 | 1.05E-14 |
| 149633_at | FBgn0037819 | CG14688 | chr3R | -6.074089249 | 4.16E-09 |
| 149706_at | FBgn0037947 | CG6930 | chr3R | -60.54731375 | 2.12E-06 |
| 149906_i_at | FBgn0038243 | CG8066 | chr3R | -20.63428482 | 7.74E-05 |
| 149907_r_at | FBgn0038243 | CG8066 | chr3R | -17.20630164 | 0.00012374 |
| 149913_at | FBgn0038255 | CG7552 | chr3R | -30.72095616 | 6.92E-07 |
| 149940_at | FBgn0038302 | CG4210 | chr3R | -15.62019155 | 3.56E-05 |
| 149974_at | FBgn0038363 | CG18505 | chr3R | -3.323550805 | 2.14E-05 |
| 150016_at | FBgn0038430 | CG14895 | chr3R | -7.077932511 | 1.11E-09 |
| 150269_at | FBgn0038813 | CG5206 | chr3R | -41.1006593 | 9.19E-06 |
| 150331_at | FBgn0038908 | CG6637 | chr3R | -4.815931963 | 1.22E-07 |
| 150558_at | FBgn0039288 | CG13653 | chr3R | -70.79593765 | 1.20E-06 |
| 151087_at | FBgn0040602 | CG14545 | chr3R | -6.520780077 | 2.21E-05 |
| 151178_at | FBgn0040701 | NA | chr3L | -30.30358234 | 4.63E-13 |
| 151179_at | FBgn0040702 | CG13713 | chr3L | -129.8324067 | 5.48E-09 |
| 151287_at | FBgn0040813 | CG11051 | chr3L | -6.672907061 | 1.45E-06 |
| 151599_at | HL01913.3prime-hit | CG1225 | chr3L | -494.5261049 | 8.61E-10 |
| 151681_at | LD29569.3prime-hit | CG32306 | chr3L | -13.68170051 | 2.16E-12 |
| 151695_at | LD33458.3prime-hit | NA | chr3R | -12.53753657 | 1.30E-05 |
| 151758_at | SD09884.3prime-hit | CG11100 | chr3L | -13.35591637 | 8.29E-05 |
| 151806_at | FBgn0036732 | CG7571 | chr3L | -74.37033148 | 5.96E-07 |
| 151807_at | FBgn0039537 | CG5590 | chr3R | -3.488746293 | 6.33E-06 |
| 151904_at | FBgn0027598 | CG31012 | chr3R | -22.02426964 | 7.74E-09 |
| 151918_s_at | FBgn0027594 | CG2086 | chr3L | -13.15174488 | 7.63E-09 |
| 152037_at | FBgn0035889 | CG33057/CG7 | chr3L | -50.01951083 | 2.36E-06 |
| 152200_at | FBgn0003308 | CG7642 | chr3R | -127.3359556 | 8.73E-08 |
| 152253_at | FBgn0037216 | CG12582 | chr3R | -47.40099279 | 3.61E-07 |
| 152563_at | FBgn0038803 | CG5191 | chr3R | -5.614024679 | 2.92E-08 |
| 152735_at | FBgn0036136 | CG6233 | chr3L | -25.34204813 | 5.63E-07 |
| 152914_at | FBgn0036684 | CG3764 | chr3L | -22.75693077 | 5.60E-09 |
| 153349_at | FBgn0038608 | CG7670 | chr3R | -17.31819777 | 0.00017446 |
| 153576_at | FBgn0036182 | CG6084 | chr3L | -3.390947312 | 6.47E-07 |
| 153731_at | FBgn0001225 | CG4183 | chr3L | -6.870225113 | 4.27E-08 |
| 153758_at | FBgn0000338 | CG17894 | chr3R | -6.318289404 | 5.36E-06 |
| 153779_at | FBgn0000368 | CG6383 | chr3R | -43.60121676 | 3.77E-14 |
| 154146_at | FBgn0001323 | CG4761 | chr3L | -9.77012675 | 0.00012517 |
| 154388_at | FBgn0010340 | CG9852 | chr3R | -13.21585042 | 0.00029383 |
| 154482_at | FBgn0037556 | CG9636 | chr3R | -3.555287975 | 0.00081625 |
| 154602_at | FBgn0010389 | CG7223 | chr3R | -88.48448839 | 1.72E-06 |

| | | | | | |
|---|---|---|---|---|---|
| 154821_at | FBgn0037699 | CG8147 | chr3R | -229.9680718 | 2.83E-09 |
| 154880_at | FBgn0000071 | CG2198 | chr3R | -494.7113856 | 2.66E-14 |
| 154929_at | FBgn0039286 | CG11849 | chr3R | -2961.521527 | 3.38E-11 |
| 154936_at | FBgn0038184 | CG9285 | chr3R | -4.193070081 | 4.81E-08 |
| 155012_at | FBgn0038538 | CG7660 | chr3R | -11.78415037 | 3.99E-11 |
| 155072_at | FBgn0035745 | CG32386 | chr3L | -48.46981845 | 3.58E-09 |
| 155107_at | FBgn0022349 | CG1910 | chr3R | -3.659083699 | 1.18E-07 |
| 141713_at | FBgn0004643 | CG9900 | chrX | -144.0344433 | 8.33E-08 |
| 142777_at | FBgn0004655 | CG3707 | chrX | -10.39996027 | 4.74E-05 |
| 142824_at | FBgn0022768 | CG2984 | chrX | -3.349488096 | 0.00058077 |
| 143133_at | FBgn0000499 | CG18361 | chrX | -7.04322649 | 5.99E-09 |
| 143135_at | FBgn0000524 | CG3929 | chrX | -14.96306892 | 2.68E-05 |
| 143175_at | FBgn0001092 | CG8893 | chrX | -5.885673966 | 8.18E-07 |
| 143185_at | FBgn0001150 | CG7952 | chrX | -37.61589914 | 8.08E-09 |
| 143237_at | FBgn0002561 | CG3839 | chrX | -36.9976416 | 8.15E-06 |
| 143359_at | FBgn0003463 | CG9224 | chrX | -10.82949421 | 0.00020881 |
| 143373_at | FBgn0003659 | CG33070 | chrX | -11.1950905 | 0.00013737 |
| 143386_at | FBgn0003865 | CG1502 | chrX | -6.992435599 | 4.64E-08 |
| 143471_at | FBgn0004446 | CG32659 | chrX | -3.437929002 | 0.00024739 |
| 143841_at | FBgn0015773 | CG18657 | chrX | -254.6798885 | 1.55E-08 |
| 144094_at | FBgn0025800 | CG2262 | chrX | -67.07769398 | 1.18E-06 |
| 144430_at | FBgn0029650 | CG3939 | chrX | -3.374287903 | 1.47E-05 |
| 144619_at | FBgn0029907 | CG4547 | chrX | -74.86507578 | 9.50E-07 |
| 144630_s_at | FBgn0029920 | CG4575/CG79 | chrX | -32.49527683 | 8.40E-08 |
| 144635_at | FBgn0029927 | CG14430 | chrX | -46.18386319 | 3.30E-11 |
| 144639_at | FBgn0029931 | CG14427 | chrX | -52.79865677 | 7.74E-11 |
| 144730_at | FBgn0030090 | CG12664 | chrX | -4.516312778 | 0.00014123 |
| 144784_at | FBgn0030180 | CG12102 | chrX | -3.950749049 | 5.58E-05 |
| 145025_at | FBgn0030510 | CG12177 | chrX | -5.683622221 | 0.00015319 |
| 145095_at | FBgn0030602 | CG32594 | chrX | -29.49693294 | 1.36E-05 |
| 145222_at | FBgn0030807 | CG13000 | chrX | -492.3378039 | 9.89E-10 |
| 145379_at | FBgn0031036 | CG14220 | chrX | -18.46751952 | 3.27E-05 |
| 151326_at | FBgn0040853 | CG32666 | chrX | -18.84576169 | 8.78E-05 |
| 151389_at | FBgn0040920 | CG32736 | chrX | -6.019656659 | 0.00069322 |
| 151495_at | GH06717.3prime-hit | CG2930 | chrX | -25.2528433 | 1.03E-06 |
| 151504_i_at | GH10316.complete-hit | CG3620 | chrX | -4.372962594 | 5.68E-05 |
| 151603_at | HL05860.3prime-hit | CG7053 | chrX | -43.42567078 | 1.46E-10 |
| 151610_at | LD03548.3prime-hit | CG3026 | chrX | -3.045337843 | 0.00012442 |
| 151660_at | LD21771.3prime-hit | CG3429 | chrX | -5.248301022 | 0.00013059 |
| 151863_at | FBgn0003300 | CG1849 | chrX | -27.31367272 | 2.21E-05 |
| 152338_at | FBgn0003053 | CG12212 | chrX | -107.8236687 | 1.24E-07 |
| 153044_at | FBgn0015565 | CG2913 | chrX | -9.588917164 | 1.11E-08 |
| 153222_at | FBgn0030274 | CG1908 | chrX | -65.10946786 | 9.62E-07 |
| 153230_at | FBgn0024251 | CG32527 | chrX | -46.48266827 | 1.02E-05 |
| 153332_at | FBgn0029958 | CG12151 | chrX | -28.54075047 | 2.14E-05 |
| 153647_at | FBgn0014133 | CG1822 | chrX | -3.677547286 | 4.41E-05 |
| 153751_at | FBgn0030003 | CG2116 | chrX | -5.241721318 | 3.53E-06 |
| 154137_at | FBgn0010315 | CG9096 | chrX | -3.590181537 | 3.54E-05 |
| 154221_at | FBgn0011761 | CG4193 | chrX | -31.18374974 | 3.90E-11 |
| 154261_at | FBgn0030973 | CG7332 | chrX | -5.37043855 | 2.30E-06 |
| 154424_at | FBgn0028469 | CG8062 | chrX | -3.503806404 | 0.00050928 |
| 154558_at | FBgn0026080 | CG6121 | chrX | -6.766100342 | 6.52E-08 |
| 154638_at | FBgn0031062 | CG14230 | chrX | -4.052445284 | 2.95E-05 |
