# Supplementary material for: Unmasking Activation of the Zygotic Genome Using Chromosomal Deletions in the Drosophila Embryo
Source: PLoS Biol. 2007 Apr 24;5(5):e117. doi: 10.1371/journal.pbio.0050117 (PMC1854917; doi:10.1371/journal.pbio.0050117)
Supplement: Supplementary file 4 [file pbio.0050117.st004.pdf]

Pure Zygotic Transcripts (Chromosome Deletion)

141238_at	FBgn0032046	CG10595	chr2L	-15.089311	6.16E-12
141280_at	FBgn0032681	CG10283	chr2L	-90.362179	0
141443_at	FBgn0031717	CG6957	chr2L	-27.1666098	6.75E-10
142165_at	FBgn0031245	CG3625	chr2L	-8.00747861	5.55E-16
142230_at	FBgn0004583	CG4114	chr2L	-21.8648802	3.58E-08
142432_at	FBgn0032377	CG14937	chr2L	-147.711621	4.53E-13
142749_at	FBgn0023416	CG1506	chr2L	-5.52430654	4.54E-05
142879_at	FBgn0010583	CG3727	chr2L	-7.70958031	1.45E-12
143064_at	FBgn0000058	CG1374	chr2L	-68.2171031	1.78E-11
143089_at	FBgn0000227	CG12205	chr2L	-206.462941	0
143131_at	FBgn0000490	CG9885	chr2L	-35.7536957	4.36E-09
143219_at	FBgn0001981	CG3758	chr2L	-37.8913369	0
143221_at	FBgn0001987	CG3903	chr2L	-25.2224025	0
143285_at	FBgn0002970	CG6246	chr2L	-66.5198566	4.44E-12
143286_at	FBgn0002985	CG3851	chr2L	-87.6474971	0
143331_at	FBgn0003313	CG4922	chr2L	-179.765021	0
143356_at	FBgn0003448	CG3956	chr2L	-176.796814	0
143414_at	FBgn0004009	CG4889	chr2L	-101.51548	3.68E-12
143457_at	FBgn0004394	CG12287	chr2L	-289.82846	0
143481_at	FBgn0004567	CG2939	chr2L	-26.0267944	6.68E-07
143487_at	FBgn0004579	CG6464	chr2L	-100.082394	6.32E-13
143529_at	FBgn0004892	CG3242	chr2L	-10.5161113	1.22E-15
143555_at	FBgn0005616	CG3241	chr2L	-23.5006386	2.01E-07
143754_at	FBgn0014179	CG12245	chr2L	-20.0989766	1.26E-07

144171_at	FBgn0027104	CG10393	chr2L	-11.6278068	7.03E-10
144283_at	FBgn0028876	CG16876	chr2L	-67.9415472	3.38E-10
145509_at	FBgn0031215	CG11450	chr2L	-194.400967	2.92E-13
145753_at	FBgn0031595	CG15427	chr2L	-27.2147686	1.15E-14
145780_at	FBgn0031632	CG15628	chr2L	-40.4152988	1.11E-16
145830_at	FBgn0031718	CG14014	chr2L	-92.9944428	1.11E-16
145936_at	FBgn0031879	CG9138	chr2L	-5.46058729	9.39E-09
146014_at	FBgn0032002	CG8353	chr2L	-31.0240327	4.88E-09
146070_at	FBgn0032097	CG32982	chr2L	-5.52977366	5.73E-10
146071_at	FBgn0032098	CG32982	chr2L	-7.29374748	8.69E-10
146087_at	FBgn0032117	CG4435	chr2L	-3.00377407	0.00064424
146141_at	FBgn0032221	CG5375	chr2L	-29.6136634	2.38E-09
146147_at	FBgn0032230	CG13139	chr2L	-27.0381608	1.11E-09
146171_at	FBgn0032270	CG31721	chr2L	-3.84491924	3.08E-06
146224_at	FBgn0032335	CG14915	chr2L	-280.570632	0
146318_at	FBgn0032489	CG15480	chr2L	-26.9599495	0
146320_at	FBgn0032491	CG16815	chr2L	-13.2606125	4.44E-16
146322_at	FBgn0032493	CG15479	chr2L	-18.2025405	0
146397_at	FBgn0032629	CG15138	chr2L	-27.1655171	1.71E-09
146436_at	FBgn0032682	CG10176	chr2L	-26.7065037	7.57E-13
151444_at	FBgn0040984	CG4440	chr2L	-189.107311	0
151713_at	LD44245.3prir	CG18024	chr2L	-137.455565	5.21E-13
151809_at	FBgn0031745	CG8965	chr2L	-163.147154	1.68E-09
151981_at	FBgn0003430	CG16738	chr2L	-27.5060294	1.37E-11
152162_at	FBgn0028523	CG5888	chr2L	-119.311262	1.11E-16

152260_at	FBgn0032327	CG16833	chr2L	-8.53355566	2.00E-09
152498_at	FBgn0032099	CG32982	chr2L	-117.339094	2.13E-13
152870_at	FBgn0003145	CG6716	chr2L	-18.4609608	2.53E-14
153395_at	FBgn0000229	CG5680	chr2L	-25.6020206	3.15E-13
153728_at	FBgn0012037	CG8827	chr2L	-24.5789456	1.10E-08
153787_at	FBgn0032848	CG10722	chr2L	-35.9841101	1.97E-10
153835_at	FBgn0004893	CG10021	chr2L	-70.7609116	1.29E-09
153984_at	FBgn0026319	CG3048	chr2L	-171.674195	0
154420_at	FBgn0005771	CG4491	chr2L	-164.002134	1.04E-12
154426_at	FBgn0028519	CG4500	chr2L	-20.49812	0
154716_at	FBgn0015380	CG17348	chr2L	-70.3189381	4.55E-09
154983_at	FBgn0031621	CG15634	chr2L	-1111.11595	0
155069_at	FBgn0032693	CG10391	chr2L	-32.5728739	8.21E-13
155099_at	FBgn0000180	CG4722	chr2L	-439.64734	4.17E-13
141307_at	FBgn0034225	CG4827	chr2R	-7.62718715	2.44E-11
141373_at	FBgn0032993	CG18140	chr2h	-8.31432176	7.74E-06
141438_at	FBgn0004586	CG5058	chr2R	-189.393745	1.60E-13
141541_at	FBgn0034501	CG13868	chr2R	-558.881543	2.89E-15
141779_at	FBgn0004512	CG3879	chr2R	-181.3935	5.55E-16
142212_at	FBgn0034393	CG15095	chr2R	-140.902691	2.19E-13
142584_at	FBgn0033803	NA	chr2R	-14.6365742	2.59E-08
142609_at	FBgn0034424	CG7230	chr2R	-5.88558835	2.27E-07
142796_at	FBgn0034142	CG8306	chr2R	-3.73699132	7.77E-05
142966_at	FBgn0003731	CG10079	chr2R	-7.27505597	1.05E-10

143153_at	FBgn0000606	CG2328	chr2R	-19.5192547	0
143209_at	FBgn0001325	CG3340	chr2R	-568.870158	1.11E-16
143266_at	FBgn0002774	CG11680	chr2R	-3.90335779	5.50E-06
143299_at	FBgn0003067	CG17725	chr2R	-99.6308919	0
143376_at	FBgn0003715	CG16778	chr2R	-13.5788573	2.02E-08
143395_at	FBgn0003892	CG2411	chr2R	-233.952583	4.44E-15
143396_at	FBgn0003900	CG2956	chr2R	-172.139817	0
143584_at	FBgn0010109	CG8704	chr2R	-9.23892367	2.24E-13
143588_at	FBgn0010228	CG17921	chr2R	-118.186969	0
143702_at	FBgn0013272	CG5820	chr2R	-4107.50128	0
143714_at	FBgn0013725	CG10108	chr2R	-52.4210026	2.51E-11
145517_at	FBgn0031234	NA	chr2L	-8.13862842	2.24E-09
145519_at	FBgn0031236	CG3696	chr2L	-8.3916398	2.36E-05
145686_at	FBgn0031494	CG17219	chr2L	-5.8013834	0.00065889
145746_at	FBgn0031582	CG12676	chr2L	-16.7270351	5.11E-11
146345_at	FBgn0032532	CG31731	chr2L	-3.03224662	1.78E-06
146674_at	FBgn0033068	CG11212	chr2R	-213.464907	0
146743_at	FBgn0033174	CG11125	chr2R	-3.91701627	4.92E-10
146795_at	FBgn0033276	CG33087	chr2R	-3.3475452	0.000209
146921_at	FBgn0033483	CG12919	chr2R	-149.907374	7.57E-14
147034_at	FBgn0033649	CG13194	chr2R	-31.3745983	4.73E-09
147037_at	FBgn0033652	CG12443	chr2R	-56.3437271	1.74E-10
147068_at	FBgn0033700	CG8856	chr2R	-176.678123	1.78E-13
147192_at	FBgn0033920	CG8561	chr2R	-10.2889224	7.66E-06
147392_at	FBgn0034264	CG10933	chr2R	-21.2296887	1.86E-08

147549_at	FBgn0034514	CG13427	chr2R	-422.541279	0
147585_at	FBgn0034561	CG33152	chr2R	-3.36755831	1.03E-08
147586_at	FBgn0034562	CG33152	chr2R	-8.99559085	2.04E-06
147610_at	FBgn0034606	CG18375	chr2R	-4.00721485	1.32E-08
147701_at	FBgn0034764	CG9952	chr2R	-63.0642467	4.55E-15
147830_at	FBgn0034958	CG3907	chr2R	-32.9945079	9.44E-09
151221_at	FBgn0040745	CG30089	chr2R	-3.70844986	1.60E-07
151601_at	HL02328.3prir	NA	chr2R	-39.3134597	1.30E-08
151623_at	LD09801.3prir	CG12424	chr2R	-26.1554993	6.02E-09
151643_at	LD16058.3prir	CG7230	chr2R	-3759.80785	0
151656_at	LD19406.3prir	CG8166	chr2R	-22.9474936	2.53E-08
151735_at	LP11484.3prin	NA	chr2R	-108.682923	3.77E-13
151751_at	SD07514.3prii	CG8529	chr2R	-50.33268	1.68E-08
151811_at	FBgn0034644	CG10082	chr2R	-3.58979883	1.85E-09
151902_at	FBgn0034772	CG30092	chr2R	-25.8921821	1.07E-09
152060_at	FBgn0016762	CG12273	chr2R	-4.41680257	5.51E-09
152372_at	FBgn0034347	CG30118	chr2R	-3.93722899	0.00029306
152832_at	FBgn0033632	CG30023	chr2R	-26.2902273	1.34E-14
152874_at	FBgn0033559	CG30015	chr2R	-158.89723	1.23E-13
152886_at	FBgn0034602	CG15658	chr2R	-34.7495686	4.46E-14
152907_at	FBgn0004364	CG8896	chr2R	-2186.55991	0
153004_at	FBgn0033923	CG8603	chr2R	-153.715923	2.17E-13
153087_at	FBgn0034479	CG8654	chr2R	-19.9117923	5.33E-09
153146_at	FBgn0034220	CG6530	chr2R	-7.67090029	7.15E-05

153432_at	FBgn0022073	CG8846	chr2L	-34.8501483	1.39E-09
153535_at	FBgn0031993	CG8486	chr2L	-4.13510524	2.53E-05
153554_at	FBgn0033182	CG1621	chr2R	-10.6790383	1.81E-12
153620_at	FBgn0033552	CG12342	chr2R	-7.11368338	2.48E-07
153848_at	FBgn0033855	CG13333	chr2R	-2891.24762	0
153855_at	FBgn0034373	CG15085	chr2R	-24.424017	3.63E-13
153867_at	FBgn0000577	CG9015	chr2R	-29.1678495	7.08E-09
153969_at	FBgn0033785	CG13320	chr2R	-5.00596527	3.88E-06
154232_at	FBgn0033975	CG10228	chr2R	-5.04869076	2.02E-05
154356_at	FBgn0001123	CG2835	chr2R	-20.3983733	2.46E-08
154571_at	FBgn0032683	CG10275	chr2L	-7.53348957	4.20E-05
154691_at	FBgn0034847	CG3502	chr2R	-95.7656797	3.71E-13
154837_at	FBgn0034275	CG5002	chr2R	-26.2488299	9.94E-08
154882_at	FBgn0034041	CG30089	chr2R	-8.52056512	4.27E-06
154921_at	FBgn0032256	CG6187	chr2L	-5.57214336	0.00072704
141301_at	FBgn0004885	CG6863	chr3R	-16.4925889	4.50E-05
141352_at	FBgn0038071	CG6234	chr3R	-278.526666	3.00E-12
141377_at	FBgn0011653	CG15002	chr3L	-15.5664406	3.21E-09
141394_at	FBgn0035656	CG10479	chr3L	-31.9449854	1.47E-06
141428_at	FBgn0035289	CG12026	chr3L	-59.0207457	4.26E-14
141482_at	FBgn0037733	CG31352	chr3R	-9.89206364	1.35E-05
141505_at	FBgn0035752	CG8616	chr3L	-119.481543	7.80E-08
141518_at	FBgn0003117	CG3978	chr3R	-23.3828537	5.83E-05
141560_at	FBgn0036404	CG6451	chr3L	-3.87042948	2.04E-05
141687_at	FBgn0003507	CG3992	chr3R	-239.203027	1.12E-08

141688_at	FBgn0001257	CG15009	chr3L	-17.6844565	1.45E-12
141711_at	FBgn0035347	CG33232	chr3L	-52.7378358	7.76E-10
142177_at	FBgn0000449	CG12028	chr3L	-79.4066254	3.26E-09
142451_at	FBgn0036914	NA	chr3L	-18.5071661	0.00010657
142533_at	FBgn0021776	CG12249	chr3R	-181.630153	0
142586_at	FBgn0038981	CG5346	chr3R	-6.90442005	2.46E-06
142670_at	FBgn0004108	CG9704	chr3L	-48.0812029	1.38E-05
142737_at	FBgn0037007	CG5059	chr3L	-110.964391	1.67E-15
142813_at	FBgn0020299	CG31317	chr3R	-27.9350415	2.13E-05
142819_at	FBgn0000591	CG8365	chr3R	-89.4354483	1.05E-06
142830_at	FBgn0001316	CG17046	chr3L	-43.8816051	8.12E-06
142882_at	FBgn0039501	CG5987	chr3R	-16.2299283	0.00020905
142969_at	FBgn0004629	CG8050	chr3R	-462.588944	0
143088_at	FBgn0000216	CG3096	chr3L	-88.4839181	4.44E-16
143116_at	FBgn0000411	CG5893	chr3L	-433.430632	4.65E-09
143122_at	FBgn0000439	CG2189	chr3R	-11.8813426	0.00013494
143130_at	FBgn0000489	CG6117	chr3L	-97.237395	2.72E-07
143164_at	FBgn0000723	CG8874	chr3R	-102.42882	5.94E-07
143169_at	FBgn0001077	CG2047	chr3R	-36.1799225	2.13E-13
143180_at	FBgn0001138	CG9656	chr3R	-64.1606554	2.77E-06
143186_at	FBgn0001168	CG6494	chr3L	-192.139698	5.93E-13
143189_at	FBgn0001204	CG9768	chr3R	-25.5660847	2.71E-09
143199_at	FBgn0001235	CG17117	chr3R	-69.2983454	2.07E-06
143208_at	FBgn0001320	CG4717	chr3L	-75.5277385	3.23E-07

143251_at	FBgn0002629	CG6099	chr3R	-74.5526918	1.82E-10
143252_at	FBgn0002631	CG6096	chr3R	-10.2270505	5.37E-07
143254_at	FBgn0002633	CG8361	chr3R	-8.47283989	1.24E-09
143321_at	FBgn0003250	CG9668	chr3L	-4.54402625	5.22E-06
143367_at	FBgn0003510	CG17957	chr3R	-80.7849642	4.44E-16
143378_at	FBgn0003719	CG6868	chr3R	-42.9272849	4.77E-12
143379_at	FBgn0003720	CG1378	chr3R	-300.490445	7.85E-09
143419_at	FBgn0004052	CG17962	chr3L	-2052.64366	3.42E-11
143420_at	FBgn0004053	CG1046	chr3R	-17.9834383	2.86E-12
143472_at	FBgn0004449	CG5723	chr3L	-20.2150928	7.01E-05
143483_at	FBgn0004569	CG4531	chr3L	-77.8987025	7.04E-07
143496_at	FBgn0004595	CG17228	chr3R	-13.4958963	3.22E-06
143500_at	FBgn0004635	CG1004	chr3L	-99.2240408	5.95E-07
143583_at	FBgn0010105	CG17943	chr3L	-48.0897069	1.21E-07
143639_at	FBgn0011225	CG5695	chr3R	-23.601931	8.48E-06
143683_at	FBgn0011723	CG7260	chr3L	-7.26566073	5.07E-09
143718_at	FBgn0013755	CG7960	chr3L	-47.5349544	2.42E-13
143752_at	FBgn0014143	CG5069	chr3L	-3.35295244	1.52E-05
143756_at	FBgn0014343	CG10601	chr3L	-21.1209218	7.18E-05
143879_at	FBgn0016700	CG3320	chr3R	-3.85468485	5.57E-06
144007_at	FBgn0024150	CG10564	chr3L	-57.7767528	3.15E-06
144092_at	FBgn0025776	CG11551	chr3L	-58.8133152	8.88E-08
144112_at	FBgn0026063	CG17216	chr3R	-78.1260578	2.58E-14
144254_at	FBgn0028789	CG5133	chr3L	-6.50494053	9.33E-07
147919_at	FBgn0035123	CG18321	chr3L	-8.95800892	0.00012027

148012_at	FBgn0035287	CG13937	chr3L	-17.9358307	3.40E-08
148055_at	FBgn0035356	CG16986	chr3L	-13.5147183	3.16E-08
148089_at	FBgn0035415	CG14966	chr3L	-3.67941745	2.89E-06
148129_at	FBgn0035470	CG14980	chr3L	-7.39636302	6.81E-05
148164_at	FBgn0035525	CG11582	chr3L	-33.8681638	4.88E-07
148194_at	FBgn0035569	CG15876	chr3L	-153.828883	4.81E-12
148195_at	FBgn0035570	CG13712	chr3L	-97.1329873	1.90E-07
148197_at	FBgn0035572	CG13711	chr3L	-258.054523	7.42E-11
148227_at	FBgn0035618	CG10594	chr3L	-123.011943	1.79E-08
148232_at	FBgn0035625	CG5249	chr3L	-17.2887287	9.12E-07
148365_at	FBgn0035824	CG8281	chr3L	-5.41300192	3.58E-05
148404_at	FBgn0035898	CG6915	chr3L	-7.10506157	7.21E-09
148440_at	FBgn0035954	CG5093	chr3L	-146.586432	1.10E-07
148493_at	FBgn0036048	CG6736	chr3L	-546.294072	9.29E-11
148595_at	FBgn0036193	CG14135	chr3L	-5.27045711	4.95E-05
148702_at	FBgn0036349	CG14112	chr3L	-811.718552	4.33E-15
148703_at	FBgn0036350	CG14111	chr3L	-18.7627677	5.43E-11
148772_at	FBgn0036457	CG3396	chr3L	-4.69544006	6.89E-06
148773_s_at	FBgn0036458	CG3396	chr3L	-272.360466	7.22E-09
148806_at	FBgn0036503	CG13454	chr3L	-151.374884	8.89E-08
148822_at	FBgn0036530	CG7554	chr3L	-12.9494427	4.50E-11
148902_at	FBgn0036643	CG4109	chr3L	-18.2275933	0.00019765
148904_s_at	FBgn0036646	CG18217/CG4	chr3L	-8.61846201	2.71E-05
148988_at	FBgn0036779	CG13695	chr3L	-183.597495	2.17E-09

148995_at	FBgn0036787	CG4306	chr3L	-61.2324884	4.22E-06
148998_s_at	FBgn0036791	CG4216/CG72	chr3L	-7482.68439	8.76E-14
149010_at	FBgn0036810	CG6885	chr3L	-32.5370683	2.20E-10
149011_at	FBgn0036811	CG6884	chr3L	-3.08480609	4.40E-06
149027_at	FBgn0036840	CG11614	chr3L	-10.3196301	1.26E-05
149132_at	FBgn0037000	CG5130	chr3L	-4.03134163	4.88E-07
149138_at	FBgn0037011	CG4858	chr3L	-11.185106	2.93E-09
149180_at	FBgn0037085	CG7204	chr3L	-93.3067768	1.93E-07
149227_at	FBgn0037160	CG5723	chr3L	-3.85653685	6.87E-07
149319_at	FBgn0037309	CR31541	chr3R	-4.74748382	1.92E-05
149474_at	FBgn0037548	CG7900	chr3R	-12.2538246	9.02E-05
149558_at	FBgn0037694	CG16777	chr3R	-14.6083226	9.16E-05
149579_at	FBgn0037741	CG16908	chr3R	-8.82530233	7.90E-07
149620_at	FBgn0037797	CG12420	chr3R	-68.3599417	1.05E-14
149631_at	FBgn0037816	CG6345	chr3R	-6.38851949	1.36E-05
149686_at	FBgn0037898	CG18643	chr3R	-4.23177938	0.00040442
149706_at	FBgn0037947	CG6930	chr3R	-60.5473138	2.12E-06
149722_at	FBgn0037973	CG18547	chr3R	-9.05938215	7.25E-08
149763_at	FBgn0038028	CG10035	chr3R	-433.396055	0
149782_at	FBgn0038059	CG6489/CG31	chr3R	-53.5325376	3.83E-06
149832_at	FBgn0038134	CG8458	chr3R	-28.1901556	2.86E-06
149840_at	FBgn0038150	CG17045	chr3R	-17.6563666	1.82E-07
149906_i_at	FBgn0038243	CG8066	chr3R	-20.6342848	7.74E-05
149913_at	FBgn0038255	CG7552	chr3R	-30.7209562	6.92E-07
149919_at	FBgn0038263	CG33555	chr3R	-70.1981286	6.84E-11

149967_at	FBgn0038352	CG33207	chr3R	-6.19616142	1.67E-09
149974_at	FBgn0038363	CG18505	chr3R	-3.32355081	2.14E-05
150114_at	FBgn0038566	CG14317	chr3R	-122.767962	1.55E-15
150120_at	FBgn0038582	CG7988	chr3R	-5.21051293	1.43E-07
150139_at	FBgn0038619	CG7685	chr3R	-8.26046888	4.20E-07
150331_at	FBgn0038908	CG6637	chr3R	-4.81593196	1.22E-07
150437_at	FBgn0039068	CG13827	chr3R	-30.3710926	1.67E-05
150479_at	FBgn0039145	CG6000	chr3R	-3.91684042	6.80E-07
150554_at	FBgn0039283	CG13651	chr3R	-12.8984263	4.49E-11
150558_at	FBgn0039288	CG13653	chr3R	-70.7959377	1.20E-06
150571_at	FBgn0039306	CG11859	chr3R	-91.2809652	4.32E-07
150809_at	FBgn0039642	CG11882	chr3R	-7.72561785	6.11E-05
151178_at	FBgn0040701	NA	chr3L	-30.3035823	4.63E-13
151179_at	FBgn0040702	CG13713	chr3L	-129.832407	5.48E-09
151283_s_at	FBgn0040808	CG12487/CG1	chr3L	-837.347078	1.76E-11
151287_at	FBgn0040813	CG11051	chr3L	-6.67290706	1.45E-06
151571_at	GM01253.3pri	CG31320	chr3R	-4.04614121	0.0004549
151599_at	HL01913.3prir	CG1225	chr3L	-494.526105	8.61E-10
151695_at	LD33458.3prir	NA	chr3R	-12.5375366	1.30E-05
151767_at	FBgn0010222	CG18466	chr3R	-21.6774081	8.03E-06
151806_at	FBgn0036732	CG7571	chr3L	-74.3703315	5.96E-07
152007_at	FBgn0038332	CG6136	chr3R	-6.76931083	0.00011011
152094_at	FBgn0036545	CG17035	chr3L	-12.3631028	7.03E-10
152189_at	FBgn0038475	CG3962	chr3R	-10.1429827	2.35E-09

152200_at	FBgn0003308	CG7642	chr3R	-127.335956	8.73E-08
152325_at	FBgn0039114	CG10374	chr3R	-4.42788698	0.00099135
152335_at	FBgn0010452	CG11280	chr3L	-73.4871378	4.43E-09
152510_at	FBgn0038178	CG9322	chr3R	-270.425359	8.99E-09
152863_at	FBgn0035833	CG7565	chr3L	-75.5216053	7.10E-07
152877_at	FBgn0035315	CG8960	chr3L	-656.718847	1.49E-10
153157_at	FBgn0037213	CG12581	chr3R	-86.2158237	3.99E-12
153170_at	FBgn0037961	CG31368	chr3R	-183.766662	6.16E-08
153260_at	FBgn0026061	CG4123	chr3L	-4.22132007	2.15E-05
153274_at	FBgn0027914	CG10670	chr3L	-7.79408892	0.00070698
153328_at	FBgn0038183	CG9286	chr3R	-6.89193858	4.80E-06
153418_at	FBgn0039734	CG7908	chr3R	-39.714287	1.19E-05
153558_at	FBgn0026320	CG5185	chr3L	-475.312213	0
153583_at	FBgn0001224	CG4463	chr3L	-6.20941689	3.88E-08
153641_at	FBgn0036494	CG7250	chr3L	-26.5510726	2.10E-05
153856_at	FBgn0002528	CG3322	chr3L	-35.8758441	7.24E-06
153892_at	FBgn0036103	CG7958	chr3L	-474.484061	8.40E-11
153951_at	FBgn0001254	CG1934	chr3L	-102.285054	1.28E-07
153981_at	FBgn0037703	CG8165	chr3R	-3.03956184	0.00028587
154146_at	FBgn0001323	CG4761	chr3L	-9.77012675	0.00012517
154252_at	FBgn0002526	CG10236	chr3L	-73.4514287	2.08E-06
154290_at	FBgn0037186	CG11241	chr3L	-4.73662882	5.49E-08
154528_at	FBgn0003002	CG1133	chr3R	-3607.2853	1.90E-11
154602_at	FBgn0010389	CG7223	chr3R	-88.4844884	1.72E-06
154674_at	FBgn0039606	CG1448	chr3R	-215.586968	1.11E-15

154755_at	FBgn0004878	CG2102	chr3R	-17.1836272	1.86E-05
154821_at	FBgn0037699	CG8147	chr3R	-229.968072	2.83E-09
154833_at	FBgn0038385	CG4221	chr3R	-57.4516765	3.53E-08
154880_at	FBgn0000071	CG2198	chr3R	-494.711386	2.66E-14
154915_at	FBgn0037633	CG9839	chr3R	-4.31786387	0.00021687
154929_at	FBgn0039286	CG11849	chr3R	-2961.52153	3.38E-11
155072_at	FBgn0035745	CG32386	chr3L	-48.4698184	3.58E-09
141347_at	FBgn0030360	CG1806	chrX	-13.5716167	0.00014385
142521_at	FBgn0030960	CG7053	chrX	-55.0133243	1.12E-06
143185_at	FBgn0001150	CG7952	chrX	-37.6158991	8.08E-09
143237_at	FBgn0002561	CG3839	chrX	-36.9976416	8.15E-06
143353_at	FBgn0003411	CG1641	chrX	-21.2147049	1.10E-07
143359_at	FBgn0003463	CG9224	chrX	-10.8294942	0.00020881
143386_at	FBgn0003865	CG1502	chrX	-6.9924356	4.64E-08
143425_at	FBgn0004102	CG12154	chrX	-42.6792094	5.99E-06
143471_at	FBgn0004446	CG32659	chrX	-3.437929	0.00024739
143539_at	FBgn0004956	CG5993	chrX	-23.9178327	8.67E-05
143841_at	FBgn0015773	CG18657	chrX	-254.679889	1.55E-08
144605_at	FBgn0029885	CG3224	chrX	-4.35270862	1.10E-05
144619_at	FBgn0029907	CG4547	chrX	-74.8650758	9.50E-07
144630_s_at	FBgn0029920	CG4575/CG79	chrX	-32.4952768	8.40E-08
144635_at	FBgn0029927	CG14430	chrX	-46.1838632	3.30E-11
144639_at	FBgn0029931	CG14427	chrX	-52.7986568	7.74E-11
144784_at	FBgn0030180	CG12102	chrX	-3.95074905	5.58E-05

145025_at	FBgn0030510	CG12177	chrX	-5.68362222	0.00015319
145101_at	FBgn0030611	CG15027	chrX	-32.5738822	1.84E-05
145142_at	FBgn0030673	CG15601	chrX	-11.8368098	0.00027326
145145_at	FBgn0030676	CG12379	chrX	-8.49930075	0.0005472
145222_at	FBgn0030807	CG13000	chrX	-492.337804	9.89E-10
145319_at	FBgn0030948	CG6578	chrX	-3.90057495	2.78E-05
145381_at	FBgn0031040	CG14210	chrX	-3.77108576	7.13E-06
151326_at	FBgn0040853	CG32666	chrX	-18.8457617	8.78E-05
151471_at	GH01128.3pri	CG7893	chrX	-31.9988502	3.40E-05
151495_at	GH06717.3pri	CG2930	chrX	-25.2528433	1.03E-06
151863_at	FBgn0003300	CG1849	chrX	-27.3136727	2.21E-05
152332_at	FBgn0030251	CG2145	chrX	-92.0548005	2.53E-10
152338_at	FBgn0003053	CG12212	chrX	-107.823669	1.24E-07
152418_at	FBgn0001090	CG7088	chrX	-78.2572501	7.57E-10
152609_at	FBgn0030974	CG7358	chrX	-4.95357882	5.44E-08
153374_at	FBgn0004646	CG3039	chrX	-23.3886688	3.45E-07
