# Supplementary material for: Unmasking Activation of the Zygotic Genome Using Chromosomal Deletions in the Drosophila Embryo
Source: PLoS Biol. 2007 Apr 24;5(5):e117. doi: 10.1371/journal.pbio.0050117 (PMC1854917; doi:10.1371/journal.pbio.0050117)
Supplement: Supplementary file 5 [file pbio.0050117.st005.pdf]

Pure Zygotic Transcripts Time-Course (0-1h vs 2-3h)

141210_at	FBgn0037490	CG10053	chr3R	6.29778175	0.00249633
141238_at	FBgn0032046	CG10595	chr2L	21.2365033	9.68E-07
141263_at	FBgn0036263	CG5632	chr3L	2.99753083	0.00978979
141280_at	FBgn0032681	CG10283	chr2L	262.570092	2.64E-08
141297_at	FBgn0033806	CG4616	chr2R	1.6327159	0.07198484
141301_at	FBgn0004885	CG6863	chr3R	19.3184047	2.56E-05
141307_at	FBgn0034225	CG4827	chr2R	38.723768	2.18E-11
141347_at	FBgn0030360	CG1806	chrX	26.0881938	7.12E-08
141352_at	FBgn0038071	CG6234	chr3R	413.919007	3.43E-12
141377_at	FBgn0011653	CG15002	chr3L	48.1307974	2.29E-07
141394_at	FBgn0035656	CG10479	chr3L	131.941894	1.05E-08
141428_at	FBgn0035289	CG12026	chr3L	73.03643	8.07E-12
141432_at	FBgn0033344	CG8252	chr2R	4.90244218	0.02608725
141433_at	FBgn0032849	CG10757	chr2L	5.22328868	2.98E-07
141438_at	FBgn0004586	CG5058	chr2R	167.645125	2.11E-07
141443_at	FBgn0031717	CG6957	chr2L	52.2712565	2.38E-06
141482_at	FBgn0037733	CG31352	chr3R	5.72569195	3.49E-05
141494_at	FBgn0032922	CG9249	chr2L	3.28491674	0.06257998
141498_at	FBgn0024250	CG9653	chrX	6.55643241	6.88E-08
141505_at	FBgn0035752	CG8616	chr3L	4.99060544	3.67E-06
141518_at	FBgn0003117	CG3978	chr3R	33.886354	6.36E-05
141541_at	FBgn0034501	CG13868	chr2R	583.176677	3.04E-08
141557_at	FBgn0031286	CG3862	chr2L	-1.05767934	0.8529283
141560_at	FBgn0036404	CG6451	chr3L	3.76224008	1.67E-05

141590_at	FBgn0037968	CG12213	chr3R	1.84735499	0.00506314
141662_at	FBgn0028877	CG31732	chr2L	1.73112487	0.22977012
141673_at	FBgn0034049	CG8291	chr2R	2.36298486	8.46E-05
141675_at	FBgn0041575	CG12598	chrX	5.60558285	2.49E-05
141687_at	FBgn0003507	CG3992	chr3R	110.513192	4.16E-08
141688_at	FBgn0001257	CG15009	chr3L	28.3950171	5.84E-07
141711_at	FBgn0035347	CG33232	chr3L	4.31422479	4.96E-08
141719_at	FBgn0025806	NA	chr2R	2.44963769	0.00204842
141743_at	FBgn0035073	CG16896	chr2R	5.87103322	2.45E-07
141779_at	FBgn0004512	CG3879	chr2R	115.138846	1.39E-09
141856_at	GH09035.3pri	NA	chr3L	4.6584743	0.09459067
141873_at	GH12341.3pri	NA	chr2L	2.0730424	0.00496692
141976_at	LD21533.3prir	NA	chr3L	1.34792319	0.33225098
142024_at	LD29276.3prir	NA	chr3R	1.6205766	0.0706892
142033_at	LD32106.3prir	NA	chr3L	1.32771728	0.74166873
142046_at	LD36161.3prir	NA	chr3R	1.59221068	0.09126442
142086_at	LD47425.3prir	NA	chr2R	1.00732912	0.93704819
142165_at	FBgn0031245	CG3625	chr2L	43.1003262	6.61E-09
142177_at	FBgn0000449	CG12028	chr3L	59.6968208	9.73E-09
142205_at	FBgn0028491	CG2930	chrX	2.28866532	0.01945036
142210_at	FBgn0022740	CG5005	chr2R	3.77084536	0.01010147
142212_at	FBgn0034393	CG15095	chr2R	510.438859	1.82E-09
142256_at	FBgn0037887	CG31388	chr3R	4.27620698	0.00471494
142306_at	FBgn0035152	CG3386	chr3L	3.66334874	0.00041293
142352_at	FBgn0033769	CG8768	chr2R	2.23358455	0.01798568

142356_at	FBgn0016724	CG11064	chr4	1.25697482	0.67573931
142372_at	FBgn0033971	CG10209	chr2R	1.5491367	0.06440249
142388_at	FBgn0033110	CG9447	chr2R	2.07946117	0.05137951
142408_at	FBgn0036623	CG4729	chr3L	3.24081689	1.70E-05
142411_at	FBgn0038794	CG17205	chr3R	1.29123614	0.27799765
142432_at	FBgn0032377	CG14937	chr2L	778.185489	4.07E-10
142451_at	FBgn0036914	NA	chr3L	9.79090391	0.00035194
142470_s_at	FBgn0028842	CG5813	chr2L	3.17450194	0.00134859
142485_at	FBgn0033534	CG12052	chr2R	1.20644747	0.52804617
142486_at	FBgn0037927	CG14715	chr3R	3.70887781	0.0310146
142511_at	FBgn0035591	CG4611	chr3L	4.62628907	0.00013807
142521_at	FBgn0030960	CG7053	chrX	6.92859609	0.00018009
142533_at	FBgn0021776	CG12249	chr3R	532.099504	5.68E-10
142565_at	FBgn0033987	CG12424	chr2R	35.6905506	1.18E-05
142586_at	FBgn0038981	CG5346	chr3R	7.20009344	4.41E-06
142587_at	FBgn0038982	CG33099	chr3R	11.6317937	7.65E-05
142609_at	FBgn0034424	CG7230	chr2R	4.95242896	0.00093027
142611_at	FBgn0038829	NA	chr3R	4.58372219	2.38E-06
142642_at	FBgn0032077	CG33085	chr2L	1.76448711	0.09864637
142651_at	FBgn0034080	CG30094	chr2R	10.5155481	1.18E-08
142670_at	FBgn0004108	CG9704	chr3L	3.01161986	0.20170953
142737_at	FBgn0037007	CG5059	chr3L	61.3006178	1.89E-14
142740_at	FBgn0035906	CG6673	chr3L	5.3917516	7.87E-05
142749_at	FBgn0023416	CG1506	chr2L	13.7941181	0.0008407

142750_at	FBgn0015278	CG11621	chr3L	3.94672213	0.07708414
142776_at	FBgn0034303	CG17680	chr2R	4.53764385	6.89E-08
142797_at	FBgn0001169	CG5460	chr3R	3.09489278	0.04301196
142813_at	FBgn0020299	CG31317	chr3R	48.5750013	2.39E-06
142819_at	FBgn0000591	CG8365	chr3R	81.7772764	1.25E-06
142830_at	FBgn0001316	CG17046	chr3L	23.3850875	1.60E-05
142848_at	FBgn0032811	CG10268	chr2L	2.58281246	0.00268034
142869_at	FBgn0032290	CG6443	chr2L	5.01717429	1.45E-06
142882_at	FBgn0039501	CG5987	chr3R	16.843426	0.00018917
142885_at	FBgn0032799	CG10166	chr2L	2.18541667	0.00078088
142946_at	FBgn0020129	CG12240	chr2R	2.3750093	0.00310328
142966_at	FBgn0003731	CG10079	chr2R	5.11187582	7.95E-05
142969_at	FBgn0004629	CG8050	chr3R	1126.37802	8.52E-14
143076_at	FBgn0000109	CG18315	chr3L	4.59499138	1.53E-05
143088_at	FBgn0000216	CG3096	chr3L	137.932335	8.04E-12
143089_at	FBgn0000227	CG12205	chr2L	1004.75168	2.38E-11
143116_at	FBgn0000411	CG5893	chr3L	662.446472	3.99E-10
143122_at	FBgn0000439	CG2189	chr3R	21.7960538	2.52E-05
143130_at	FBgn0000489	CG6117	chr3L	179.703252	2.05E-07
143131_at	FBgn0000490	CG9885	chr2L	87.834257	6.59E-07
143153_at	FBgn0000606	CG2328	chr2R	9.75209239	1.89E-09
143164_at	FBgn0000723	CG8874	chr3R	168.922853	2.62E-07
143169_at	FBgn0001077	CG2047	chr3R	73.6660843	2.17E-09
143180_at	FBgn0001138	CG9656	chr3R	418.045923	1.53E-07
143185_at	FBgn0001150	CG7952	chrX	31.1858367	3.67E-08

143186_at	FBgn0001168	CG6494	chr3L	2113.93486	4.29E-12
143189_at	FBgn0001204	CG9768	chr3R	165.155486	4.54E-09
143199_at	FBgn0001235	CG17117	chr3R	455.315829	2.40E-08
143200_at	FBgn0001250	CG9623	chrX	14.5196711	0.00104394
143208_at	FBgn0001320	CG4717	chr3L	78.2529323	7.53E-08
143209_at	FBgn0001325	CG3340	chr2R	442.547573	2.42E-09
143221_at	FBgn0001987	CG3903	chr2L	45.0816669	7.66E-08
143237_at	FBgn0002561	CG3839	chrX	54.1376472	1.01E-06
143251_at	FBgn0002629	CG6099	chr3R	492.538793	3.32E-10
143252_at	FBgn0002631	CG6096	chr3R	84.97965	6.05E-08
143254_at	FBgn0002633	CG8361	chr3R	8.47851588	2.58E-09
143286_at	FBgn0002985	CG3851	chr2L	168.536698	2.37E-09
143299_at	FBgn0003067	CG17725	chr2R	166.572692	2.62E-08
143321_at	FBgn0003250	CG9668	chr3L	21.390396	1.24E-06
143331_at	FBgn0003313	CG4922	chr2L	5543.54967	3.07E-11
143333_at	FBgn0003326	CG17579	chr2R	36.3231102	1.87E-08
143334_at	FBgn0003328	CG8095	chr2R	2.90190184	0.18634617
143350_at	FBgn0003378	CG6132	chr3L	1.20774795	0.5123712
143353_at	FBgn0003411	CG1641	chrX	190.155068	3.11E-10
143356_at	FBgn0003448	CG3956	chr2L	170.030143	7.61E-09
143359_at	FBgn0003463	CG9224	chrX	26.434219	6.27E-06
143367_at	FBgn0003510	CG17957	chr3R	314.495279	4.44E-16
143376_at	FBgn0003715	CG16778	chr2R	5.51896313	4.07E-05
143378_at	FBgn0003719	CG6868	chr3R	265.213249	8.97E-10

143379_at	FBgn0003720	CG1378	chr3R	546.568972	2.24E-09
143386_at	FBgn0003865	CG1502	chrX	14.1284705	8.85E-08
143388_at	FBgn0003884	NA	chr3R	1.51689456	0.14646062
143396_at	FBgn0003900	CG2956	chr2R	137.572624	7.77E-15
143414_at	FBgn0004009	CG4889	chr2L	234.227181	1.96E-07
143419_at	FBgn0004052	CG17962	chr3L	9970.26334	6.55E-13
143420_at	FBgn0004053	CG1046	chr3R	32.9206155	4.95E-12
143425_at	FBgn0004102	CG12154	chrX	130.111862	9.59E-08
143440_at	FBgn0004227	CG4211	chrX	35.9053042	8.83E-05
143457_at	FBgn0004394	CG12287	chr2L	373.565417	3.47E-10
143471_at	FBgn0004446	CG32659	chrX	19.0012867	4.64E-05
143472_at	FBgn0004449	CG5723	chr3L	44.0712278	2.46E-05
143483_at	FBgn0004569	CG4531	chr3L	31.5773422	0.00015382
143487_at	FBgn0004579	CG6464	chr2L	72.0854958	1.86E-07
143496_at	FBgn0004595	CG17228	chr3R	9.77902566	1.31E-08
143500_at	FBgn0004635	CG1004	chr3L	149.647481	1.35E-07
143501_at	FBgn0004644	CG4637	chr3R	7.26174352	0.00367477
143521_at	FBgn0004859	CG2125	chr4	251.438536	2.19E-09
143539_at	FBgn0004956	CG5993	chrX	470.568363	1.07E-08
143578_at	FBgn0010014	CG4209	chrX	2.26768617	0.14360168
143583_at	FBgn0010105	CG17943	chr3L	72.8890222	9.01E-08
143584_at	FBgn0010109	CG8704	chr2R	15.8433464	3.72E-05
143588_at	FBgn0010228	CG17921	chr2R	3118.56138	3.79E-12
143595_at	FBgn0010323	CG2851	chr2L	81.6922508	6.24E-06
143637_at	FBgn0011206	CG4760	chr3L	4.63194426	8.26E-06

143639_at	FBgn0011225	CG5695	chr3R	-1.6363731	0.01892877
143643_at	FBgn0011274	CG6794	chr2L	1.8064668	0.50222644
143683_at	FBgn0011723	CG7260	chr3L	19.9116531	2.75E-08
143702_at	FBgn0013272	CG5820	chr2R	2188.3454	4.41E-11
143711_at	FBgn0013435	CG1362	chr2R	2.37919338	0.00036945
143714_at	FBgn0013725	CG10108	chr2R	86.8489817	5.96E-08
143718_at	FBgn0013755	CG7960	chr3L	173.179501	1.26E-13
143752_at	FBgn0014143	CG5069	chr3L	4.89692841	4.61E-06
143754_at	FBgn0014179	CG12245	chr2L	39.9890509	2.80E-06
143756_at	FBgn0014343	CG10601	chr3L	41.5250543	9.54E-06
143788_at	FBgn0015276	CG2152	chr3R	3.70451712	3.16E-07
143841_at	FBgn0015773	CG18657	chrX	310.64874	1.46E-08
143879_at	FBgn0016700	CG3320	chr3R	16.1329631	4.26E-07
144007_at	FBgn0024150	CG10564	chr3L	168.832996	1.26E-08
144012_at	FBgn0024234	CG5562	chr2R	176.374387	4.74E-08
144066_at	FBgn0025519	CG7773	chr2R	1.8724437	0.05418275
144092_at	FBgn0025776	CG11551	chr3L	43.2101007	4.75E-07
144112_at	FBgn0026063	CG17216	chr3R	1111.27657	1.65E-11
144144_at	FBgn0026417	CG2525	chr3R	5.84763454	2.87E-05
144171_at	FBgn0027104	CG10393	chr2L	6.14006964	0.00035471
144229_at	FBgn0028530	CG18124	chr2L	5.12344886	1.61E-07
144243_at	FBgn0028679	CG10913	chr2R	3.73122806	1.96E-05
144254_at	FBgn0028789	CG5133	chr3L	17.6618952	8.14E-11
144271_at	FBgn0028860	CG15266	chr2L	5.08161496	1.13E-06

144420_at	FBgn0029640	CG3526	chrX	2.03554958	0.07684559
144605_at	FBgn0029885	CG3224	chrX	2.96126417	6.93E-05
144619_at	FBgn0029907	CG4547	chrX	32.7939278	4.70E-07
144630_s_at	FBgn0029920	CG4575/CG79	chrX	138.704868	5.35E-09
144635_at	FBgn0029927	CG14430	chrX	55.0329914	1.66E-10
144639_at	FBgn0029931	CG14427	chrX	248.987103	2.70E-11
144678_at	FBgn0030001	CG15335	chrX	1.69726972	0.08946995
144710_at	FBgn0030057	CG12108	chrX	6.2007618	1.05E-06
144728_at	FBgn0030085	CG6999/CG32	chrX	3.3473449	0.02800373
144784_at	FBgn0030180	CG12102	chrX	4.41713455	7.68E-05
144791_at	FBgn0030190	NA	chrX	1.38316659	0.13568712
144811_at	FBgn0030215	CG15296	chrX	2.83995296	0.20141191
144868_at	FBgn0030292	CG11752	chrX	1.57971579	0.00788397
144920_at	FBgn0030373	CG12721	chrX	4.2668163	6.56E-06
144937_at	FBgn0030390	CG15731	chrX	2.20827904	0.06225411
145025_at	FBgn0030510	CG12177	chrX	9.62159051	4.55E-05
145070_at	FBgn0030571	CG14414	chrX	2.95525142	0.00090629
145101_at	FBgn0030611	CG15027	chrX	6.14777601	5.29E-06
145130_at	FBgn0030657	CG15645	chrX	7.09602248	1.08E-07
145142_at	FBgn0030673	CG15601	chrX	1.58893534	0.10627257
145145_at	FBgn0030676	CG12379	chrX	20.4382864	1.08E-05
145222_at	FBgn0030807	CG13000	chrX	2243.44512	3.97E-12
145319_at	FBgn0030948	CG6578	chrX	6.39948155	5.76E-08
145376_at	FBgn0031032	CG14204	chrX	5.65560925	4.81E-07
145381_at	FBgn0031040	CG14210	chrX	3.6956809	7.86E-06

145396_at	FBgn0031070	CG12702	chrX	2.99353204	0.00533003
145460_at	FBgn0031156	CG32514	chrX	1.20490706	0.49814487
145509_at	FBgn0031215	CG11450	chr2L	83.4343903	2.92E-07
145517_at	FBgn0031234	NA	chr2L	2.93341312	2.22E-05
145564_at	FBgn0031315	CG14341	chr2L	6.90727723	1.63E-07
145586_at	FBgn0031346	CG7428	chr2L	564.003533	1.30E-13
145621_at	FBgn0031393	CG15382	chr2L	117.484152	7.39E-10
145625_f_at	FBgn0031400	CG7074	chr2L	1.8490621	0.00508565
145663_at	FBgn0031452	CG2843	chr2L	4.77715673	7.29E-06
145669_at	FBgn0031463	CG15400	chr2L	1.31930834	0.50382649
145686_at	FBgn0031494	CG17219	chr2L	6.55806066	0.00026386
145753_at	FBgn0031595	CG15427	chr2L	23.2565008	3.29E-07
145759_at	FBgn0031603	CG15432	chr2L	13.8280162	2.68E-06
145774_at	FBgn0031623	CG15632	chr2L	4.80187129	0.00556788
145780_at	FBgn0031632	CG15628	chr2L	34.5779161	7.10E-10
145830_at	FBgn0031718	CG14014	chr2L	386.167458	6.36E-10
145836_at	FBgn0031726	CR7249	chr2L	2.75001521	0.00107787
145867_at	FBgn0031772	CG13994	chr2L	5.37102186	4.03E-07
145936_at	FBgn0031879	CG9138	chr2L	13.2245062	1.57E-05
146008_at	FBgn0031989	CG31900	chr2L	8.13640855	0.00012525
146014_at	FBgn0032002	CG8353	chr2L	16.4815974	5.36E-05
146070_at	FBgn0032097	CG32982	chr2L	3.22203074	0.02633768
146087_at	FBgn0032117	CG4435	chr2L	2.55361029	0.05129066
146107_at	FBgn0032150	CG13123	chr2L	2.86521172	0.00033682

146108_at	FBgn0032153	CG4537	chr2L	10.1454142	1.45E-07
146119_at	FBgn0032178	CG4804	chr2L	3.84773506	0.00495572
146147_at	FBgn0032230	CG13139	chr2L	49.7329607	1.10E-06
146157_at	FBgn0032247	CG5188	chr2L	4.23277765	2.62E-05
146188_at	FBgn0032293	CG6444	chr2L	4.55285876	1.88E-07
146201_at	FBgn0032309	CG17085	chr2L	1.58236955	0.08114348
146218_at	FBgn0032329	CG16840	chr2L	6.87470539	9.81E-09
146224_at	FBgn0032335	CG14915	chr2L	741.69487	9.33E-15
146239_at	FBgn0032357	CG32955	chr2L	3.86642795	1.45E-06
146281_at	FBgn0032421	CG17218	chr2L	4.59784067	2.46E-05
146318_at	FBgn0032489	CG15480	chr2L	38.6561219	9.41E-09
146319_at	FBgn0032490	CG16813	chr2L	1.91955013	0.01980704
146320_at	FBgn0032491	CG16815	chr2L	15.5341314	3.15E-07
146322_at	FBgn0032493	CG15479	chr2L	57.6142288	6.47E-10
146397_at	FBgn0032629	CG15138	chr2L	61.3385496	1.88E-06
146407_at	FBgn0032642	CG5110	chr2L	4.70093712	9.43E-08
146416_at	FBgn0032655	CG7527	chr2L	1.27944825	0.60038085
146436_at	FBgn0032682	CG10176	chr2L	59.1715232	5.13E-09
146470_at	FBgn0032744	CG15173	chr2L	9.24168294	0.00032185
146474_at	FBgn0032751	CG17343	chr2L	4.02894884	5.01E-05
146480_at	FBgn0032761	CG17347	chr2L	5.93143224	2.08E-07
146586_at	FBgn0032946	CG8663	chr2L	1.92771762	0.36316238
146670_at	FBgn0033060	CG7849	chr2R	4.71047546	3.82E-06
146674_at	FBgn0033068	CG11212	chr2R	493.937783	2.09E-10
146681_at	FBgn0033085	CG15908	chr2R	3.36824938	0.00046956

146682_at	FBgn0033086	CG9410	chr2R	-1.04176167	0.79125887
146714_at	FBgn0033133	CG12841	chr2R	1.44306253	0.18279896
146743_at	FBgn0033174	CG11125	chr2R	5.83326279	3.28E-06
146792_at	FBgn0033268	CG2297	chr2R	7.00783127	0.00011696
146862_at	FBgn0033372	CG13742	chr2R	3.15979922	0.00032338
146864_at	FBgn0033375	CG8078	chr2R	3.2038776	0.00019318
146878_at	FBgn0033406	CG8799	chr2R	1.24837641	0.22382987
146908_at	FBgn0033458	CG18446	chr2R	15.9181105	2.55E-08
146912_at	FBgn0033467	CG15863	chr2R	10.2448032	2.55E-06
146921_at	FBgn0033483	CG12919	chr2R	307.889404	3.66E-08
146936_at	FBgn0033507	CG12909	chr2R	9.14435319	6.58E-07
146948_at	FBgn0033523	CG12895	chr2R	7.16348277	2.47E-08
146986_at	FBgn0033588	CG13228	chr2R	3.61766462	0.0001914
147008_at	FBgn0033612	CG13210	chr2R	2.58627877	0.00134176
147039_at	FBgn0033655	NA	chr2R	8.97343623	7.10E-09
147068_at	FBgn0033700	CG8856	chr2R	730.923571	7.74E-10
147077_at	FBgn0033712	CG13163	chr2R	3.1580407	0.00230391
147080_at	FBgn0033716	CG8493	chr2R	2.92115271	0.0001867
147192_at	FBgn0033920	CG8561	chr2R	9.82375969	0.00029035
147209_at	FBgn0033944	CG30077	chr2R	2.59976195	0.0091592
147211_at	FBgn0033948	CG12863	chr2R	1.41016973	0.14507816
147252_at	FBgn0034023	CG12960	chr2R	1.34779821	0.7392676
147257_at	FBgn0034033	CG8204	chr2R	3.49208878	0.03151892
147260_at	FBgn0034039	CG30080	chr2R	373.942798	3.66E-09

147273_at	FBgn0034065	CG8395	chr2R	3.54231799	1.08E-05
147339_at	FBgn0034166	CG6472	chr2R	1.19494777	0.41463786
147369_at	FBgn0034232	CG4866	chr2R	8.35353335	2.07E-08
147379_at	FBgn0034248	CG14483	chr2R	8.96741323	6.37E-09
147384_at	FBgn0034254	CG10683	chr2R	1.81834233	0.08600483
147392_at	FBgn0034264	CG10933	chr2R	30.7069789	1.89E-05
147414_at	FBgn0034302	CG12767	chr2R	2.37938153	0.00263746
147488_at	FBgn0034429	CG18607	chr2R	1.34717448	0.4160014
147495_at	FBgn0034441	CG10081	chr2R	1.52414004	0.52848091
147497_at	FBgn0034443	CG10460	chr2R	12.7020531	2.68E-08
147513_at	FBgn0034467	CG15128	chr2R	11.8164179	7.67E-05
147514_at	FBgn0034468	CG11797	chr2R	19.0598432	3.11E-05
147549_at	FBgn0034514	CG13427	chr2R	20818.1547	9.37E-14
147552_at	FBgn0034518	CG33008	chr2R	4.59415426	0.00213436
147555_at	FBgn0034523	CG13434	chr2R	5.55446053	6.05E-07
147585_at	FBgn0034561	CG33152	chr2R	4.63815623	2.68E-06
147610_at	FBgn0034606	CG18375	chr2R	2.13377354	0.01157617
147690_at	FBgn0034752	CG4414	chr2R	1.58679849	0.37375614
147749_at	FBgn0034828	CG13545	chr2R	1.52078617	0.0390676
147768_at	FBgn0034851	CG11079	chr2R	1.28430298	0.42837503
147830_at	FBgn0034958	CG3907	chr2R	11.8004979	0.00077891
147911_at	FBgn0035109	CG13876	chr3L	1.86143068	0.10318408
147919_at	FBgn0035123	CG18321	chr3L	7.77387564	0.0002318
147971_at	FBgn0035210	CG2213	chr3L	4.06036642	1.36E-07
148012_at	FBgn0035287	CG13937	chr3L	1.3870385	0.08173054

148055_at	FBgn0035356	CG16986	chr3L	4.00937677	1.82E-05
148089_at	FBgn0035415	CG14966	chr3L	10.3139324	1.75E-07
148128_at	FBgn0035469	CG14977	chr3L	7.48968577	2.72E-08
148129_at	FBgn0035470	CG14980	chr3L	3.99346458	0.00016978
148149_at	FBgn0035504	CG15003	chr3L	1.8860913	0.33451289
148164_at	FBgn0035525	CG11582	chr3L	135.076722	3.76E-08
148191_at	FBgn0035563	CG13716	chr3L	4.47018359	0.01638524
148194_at	FBgn0035569	CG15876	chr3L	197.435406	2.51E-10
148195_at	FBgn0035570	CG13712	chr3L	152.78208	7.29E-09
148197_at	FBgn0035572	CG13711	chr3L	1120.43505	3.16E-11
148227_at	FBgn0035618	CG10594	chr3L	993.501597	3.54E-11
148232_at	FBgn0035625	CG5249	chr3L	11.6604675	4.32E-05
148235_at	FBgn0035636	CG10546	chr3L	1.1804708	0.51166254
148365_at	FBgn0035824	CG8281	chr3L	2.29856269	0.00374651
148379_at	FBgn0035854	CG8005	chr3L	4.65180019	8.35E-06
148404_at	FBgn0035898	CG6915	chr3L	10.4619006	1.23E-08
148406_at	FBgn0035900	CG6694	chr3L	2.5687435	0.00120365
148440_at	FBgn0035954	CG5093	chr3L	159.884752	5.90E-08
148441_at	FBgn0035956	CG5187	chr3L	5.72871119	0.00054852
148460_at	FBgn0035982	CG4461	chr3L	2.02528772	0.01943757
148465_at	FBgn0035996	CG3448	chr3L	2.28765821	0.21769827
148493_at	FBgn0036048	CG6736	chr3L	2226.96198	4.32E-11
148535_at	FBgn0036105	CG14149	chr3L	5.6004495	6.40E-05
148546_at	FBgn0036121	CG6310	chr3L	4.67387789	8.18E-06

148594_at	FBgn0036192	CG14133	chr3L	4.66065481	1.62E-06
148595_at	FBgn0036193	CG14135	chr3L	7.04033302	0.00011948
148667_at	FBgn0036297	CG14121	chr3L	8.95330887	4.96E-06
148681_at	FBgn0036324	CG12520	chr3L	1.11035312	0.943225
148702_at	FBgn0036349	CG14112	chr3L	1974.34206	2.24E-13
148703_at	FBgn0036350	CG14111	chr3L	62.549333	3.02E-07
148705_at	FBgn0036352	CG14110	chr3L	1.66564212	0.34846268
148772_at	FBgn0036457	CG3396	chr3L	7.56627236	4.17E-07
148773_s_at	FBgn0036458	CG3396	chr3L	481.543058	2.72E-10
148806_at	FBgn0036503	CG13454	chr3L	287.68052	1.58E-08
148822_at	FBgn0036530	CG7554	chr3L	18.4153766	2.04E-11
148849_at	FBgn0036573	CG5161	chr3L	2.23861652	0.01903974
148875_at	FBgn0036602	CG13042	chr3L	1.8568076	0.00514377
148902_at	FBgn0036643	CG4109	chr3L	1.92884497	0.04914282
148904_s_at	FBgn0036646	CG18217/CG4	chr3L	3.12411175	0.00085764
148920_at	FBgn0036670	CG13029	chr3L	1.45355125	0.31813924
148961_at	FBgn0036734	CG7564	chr3L	3.68323957	0.00020394
148988_at	FBgn0036779	CG13695	chr3L	178.203525	4.16E-09
148995_at	FBgn0036787	CG4306	chr3L	13.6455093	0.00079129
148998_s_at	FBgn0036791	CG4216/CG72	chr3L	9176.09729	3.74E-13
149010_at	FBgn0036810	CG6885	chr3L	45.9320003	7.01E-10
149011_at	FBgn0036811	CG6884	chr3L	17.0513181	1.58E-10
149027_at	FBgn0036840	CG11614	chr3L	12.0262307	1.25E-05
149132_at	FBgn0037000	CG5130	chr3L	3.98327591	1.43E-05
149138_at	FBgn0037011	CG4858	chr3L	5.95044679	2.03E-07

149180_at	FBgn0037085	CG7204	chr3L	95.8164362	2.90E-08
149227_at	FBgn0037160	CG5723	chr3L	6.79931543	4.29E-07
149319_at	FBgn0037309	CR31541	chr3R	10.9945897	0.00023217
149338_at	FBgn0037340	CG14671	chr3R	5.40471956	1.65E-07
149342_at	FBgn0037349	CG14672	chr3R	1.47736009	0.07367261
149343_at	FBgn0037350	CG2911	chr3R	2.7862359	0.00150441
149421_at	FBgn0037469	CG1939	chr3R	14.1121838	1.33E-08
149474_at	FBgn0037548	CG7900	chr3R	6.9919263	4.91E-05
149475_at	FBgn0037550	CG9667	chr3R	7.58885873	1.18E-09
149477_at	FBgn0037562	CG11671	chr3R	3.15920333	0.02695534
149482_at	FBgn0037568	CG9617	chr3R	4.4069782	1.50E-05
149538_at	FBgn0037661	CG11994	chr3R	2.87769128	0.0012813
149542_at	FBgn0037667	CG16734	chr3R	1.37879809	0.34147693
149558_at	FBgn0037694	CG16777	chr3R	11.5246828	0.00053237
149567_at	FBgn0037713	CG16790	chr3R	3.17063404	0.00150454
149579_at	FBgn0037741	CG16908	chr3R	1.78556318	0.02084708
149587_at	FBgn0037753	CG12947	chr3R	5.41826576	6.58E-05
149620_at	FBgn0037797	CG12420	chr3R	321.380121	2.00E-11
149631_at	FBgn0037816	CG6345	chr3R	6.39064421	1.94E-06
149651_at	FBgn0037845	CG14694	chr3R	2.83821525	0.02333028
149676_at	FBgn0037876	CG4820	chr3R	1.95703384	0.02023379
149679_at	FBgn0037883	CG14701	chr3R	2.03330155	0.00099844
149686_at	FBgn0037898	CG18643	chr3R	7.97127873	8.12E-05
149688_at	FBgn0037902	CG5281	chr3R	6.03426145	6.15E-07

149706_at	FBgn0037947	CG6930	chr3R	40.4876941	6.03E-06
149722_at	FBgn0037973	CG18547	chr3R	3.08939562	8.11E-06
149763_at	FBgn0038028	CG10035	chr3R	655.568547	0
149782_at	FBgn0038059	CG6489/CG31	chr3R	7.2669148	0.00108421
149796_at	FBgn0038079	CG14394	chr3R	2.04490241	0.07374463
149832_at	FBgn0038134	CG8458	chr3R	73.5282993	3.98E-07
149840_at	FBgn0038150	CG17045	chr3R	57.7826099	2.46E-07
149855_at	FBgn0038172	CG9621	chr3R	1.22611902	0.65931354
149885_at	FBgn0038216	CG31322	chr3R	1.37206174	0.28409445
149906_i_at	FBgn0038243	CG8066	chr3R	1.592488	0.24340187
149912_at	FBgn0038254	CG12600	chr3R	2.04471182	0.03401396
149913_at	FBgn0038255	CG7552	chr3R	20.2741318	2.36E-05
149919_at	FBgn0038263	CG33555	chr3R	20.325565	9.48E-12
149951_at	FBgn0038324	CG5038	chr3R	1.35122	0.69225795
149967_at	FBgn0038352	CG33207	chr3R	8.49448606	6.55E-09
149974_at	FBgn0038363	CG18505	chr3R	10.3202374	2.71E-05
149991_at	FBgn0038389	CG5516	chr3R	3.73262005	4.44E-06
149995_at	FBgn0038396	CG5013	chr3R	5.27179546	1.18E-05
150048_at	FBgn0038479	CG17477	chr3R	1.36208192	0.18312836
150100_at	FBgn0038545	CG7713	chr3R	2.4255164	0.00020487
150114_at	FBgn0038566	CG14317	chr3R	221.893824	3.00E-11
150120_at	FBgn0038582	CG7988	chr3R	4.67696812	3.50E-06
150130_at	FBgn0038602	CG7126	chr3R	3.65299699	0.08983728
150139_at	FBgn0038619	CG7685	chr3R	2.0587272	0.00346003
150157_at	FBgn0038643	CG14300	chr3R	2.97414035	0.0065498

150316_at	FBgn0038891	CG15499	chr3R	1.61794817	0.28540807
150331_at	FBgn0038908	CG6637	chr3R	5.20214888	1.05E-06
150356_at	FBgn0038951	CG5380	chr3R	1.8832754	0.02156867
150396_at	FBgn0039014	CG6982	chr3R	1.12881854	0.41309873
150437_at	FBgn0039068	CG13827	chr3R	1.36253112	0.34541195
150479_at	FBgn0039145	CG6000	chr3R	3.02487322	4.78E-05
150545_at	FBgn0039259	CG11781	chr3R	4.1424581	2.98E-06
150554_at	FBgn0039283	CG13651	chr3R	22.8711186	7.50E-11
150558_at	FBgn0039288	CG13653	chr3R	221.857363	1.34E-07
150571_at	FBgn0039306	CG11859	chr3R	-1.04932657	0.96019659
150597_at	FBgn0039347	CG5071	chr3R	3.08431427	0.01695893
150682_at	FBgn0039452	CG32950	chr3R	1.64690249	0.01971933
150809_at	FBgn0039642	CG11882	chr3R	1.17551195	0.36128835
150825_at	FBgn0039670	CG7567	chr3R	1.23695416	0.25175394
150917_at	FBgn0039810	CG15549	chr3R	1.17137463	0.57623645
151100_at	FBgn0040614	NA	chr3R	1.19325379	0.36055191
151110_at	FBgn0040623	CG11500	chr3R	3.88693263	5.17E-07
151178_at	FBgn0040701	NA	chr3L	115.803578	2.97E-08
151179_at	FBgn0040702	CG13713	chr3L	187.074655	3.20E-09
151260_at	FBgn0040789	CG32202	chr3L	7.20251001	2.26E-08
151283_s_at	FBgn0040808	CG12487/CG1	chr3L	1967.78564	2.72E-11
151287_at	FBgn0040813	CG11051	chr3L	59.5166026	3.48E-07
151326_at	FBgn0040853	CG32666	chrX	4.0966884	0.00095622
151329_f_at	FBgn0040855	NA	chrX	-1.22579829	0.31192759

151381_at	FBgn0040912	NA	chrX	1.13159168	0.4710075
151444_at	FBgn0040984	CG4440	chr2L	1791.11005	6.66E-16
151460_i_at	FBgn0041005	NA	chr4	-1.15060115	0.4356147
151471_at	GH01128.3pri	CG7893	chrX	18.4740349	4.21E-05
151473_i_at	GH01240.3pri	CG3139	chr2L	1.0954131	0.88791149
151495_at	GH06717.3pri	CG2930	chrX	29.5806184	3.30E-09
151503_at	GH10173.3pri	CG10521	chrX	12.5337116	3.13E-05
151509_at	GH11902.3pri	NA	chr4	167.287632	2.94E-06
151571_at	GM01253.3pri	CG31320	chr3R	3.5608969	0.00153479
151576_s_at	GM04312.3pri	CG17964	chr4	12.785923	3.92E-06
151599_at	HL01913.3prir	CG1225	chr3L	1129.17961	1.01E-09
151623_at	LD09801.3prir	CG12424	chr2R	189.841901	1.04E-07
151643_at	LD16058.3prir	CG7230	chr2R	825.732616	9.83E-11
151656_at	LD19406.3prir	CG8166	chr2R	140.646622	7.19E-07
151695_at	LD33458.3prir	NA	chr3R	71.3876	6.04E-07
151713_at	LD44245.3prir	CG18024	chr2L	2524.56653	3.24E-10
151717_s_at	LD45505.3prir	CG11988	chr3R	2.04630347	0.0008136
151735_at	LP11484.3prin	NA	chr2R	387.12325	4.42E-09
151748_at	SD05937.3pri	NA	chr3L	17.3903246	0.0005097
151766_at	FBgn0038597	CG8064	chr3R	3.04384303	6.12E-06
151767_at	FBgn0010222	CG18466	chr3R	2.64622637	0.00539689
151806_at	FBgn0036732	CG7571	chr3L	217.012548	3.24E-08
151809_at	FBgn0031745	CG8965	chr2L	120.466221	1.75E-07
151811_at	FBgn0034644	CG10082	chr2R	1.46862793	0.06368593
151846_at	FBgn0030799	CG4872	chrX	1.98916792	0.04738916

151863_at	FBgn0003300	CG1849	chrX	48.0888759	4.00E-07
151896_at	FBgn0035528	CG15012	chr3L	6.98259852	3.37E-07
151902_at	FBgn0034772	CG30092	chr2R	171.239902	8.51E-10
151964_at	FBgn0034977	CG3253	chr2R	66.810037	1.26E-05
151977_at	FBgn0034589	CG9398	chr2R	10.3068439	0.0016033
151981_at	FBgn0003430	CG16738	chr2L	147.46077	1.17E-09
151991_at	FBgn0033566	CG18004	chr2R	3.02826834	1.12E-05
151996_at	FBgn0034797	CG12781	chr2R	449.925955	1.64E-08
152004_at	FBgn0000320	CG9554	chr2L	48.9666735	2.24E-05
152007_at	FBgn0038332	CG6136	chr3R	1.81049191	0.04708519
152010_at	FBgn0031421	CG9867	chr2L	2.91670827	0.00121907
152046_at	FBgn0031237	CG11606	chr2L	2.79357959	0.02428481
152060_at	FBgn0016762	CG12273	chr2R	4.6671653	1.03E-06
152094_at	FBgn0036545	CG17035	chr3L	5.36322154	5.28E-07
152113_at	FBgn0011746	CG8084	chr2R	6.47892999	0.00018626
152120_at	FBgn0038194	CG3050	chr3R	16.2418725	9.87E-05
152162_at	FBgn0028523	CG5888	chr2L	123.710085	2.03E-08
152189_at	FBgn0038475	CG3962	chr3R	14.4906812	3.77E-10
152200_at	FBgn0003308	CG7642	chr3R	399.377848	4.24E-09
152208_at	FBgn0010470	CG9847	chr2R	2.91580111	0.01844439
152223_at	FBgn0039137	CG13604	chr3R	2.96374138	0.06075273
152260_at	FBgn0032327	CG16833	chr2L	7.83129868	1.40E-06
152268_at	FBgn0033186	CG1602	chr2R	1.05236476	0.80859308
152325_at	FBgn0039114	CG10374	chr3R	8.85541382	0.00015949

152332_at	FBgn0030251	CG2145	chrX	83.8705626	3.78E-10
152335_at	FBgn0010452	CG11280	chr3L	232.284769	7.43E-08
152338_at	FBgn0003053	CG12212	chrX	70.0837823	1.78E-06
152372_at	FBgn0034347	CG30118	chr2R	1.69391588	0.06580838
152405_at	FBgn0027542	CG6014	chr3L	1.87150034	0.43517644
152418_at	FBgn0001090	CG7088	chrX	11421.2125	2.25E-12
152470_at	FBgn0032793	CG10189	chr2L	3.04298659	0.00944422
152488_at	FBgn0033066	CG8343	chr2R	1.14265512	0.58086716
152493_at	FBgn0031454	CG32951	chr2L	2.97638684	0.00020233
152498_at	FBgn0032099	CG32982	chr2L	136.222217	7.64E-08
152510_at	FBgn0038178	CG9322	chr3R	2377.68925	9.78E-11
152601_at	FBgn0023511	CG3810	chrX	1.14009129	0.7676823
152609_at	FBgn0030974	CG7358	chrX	7.6269977	2.37E-09
152627_at	FBgn0030052	CG12065	chrX	1.28840681	0.94379598
152702_at	FBgn0033962	CG10153	chr2R	3.35403862	8.01E-05
152705_at	FBgn0024252	CG6829	chr2R	3.27523343	0.00015261
152715_at	FBgn0033188	CG1600	chr2R	5.06253668	0.00241012
152832_at	FBgn0033632	CG30023	chr2R	18.3300973	3.69E-09
152835_at	FBgn0035980	CG4447	chr3L	3.57747692	3.09E-05
152863_at	FBgn0035833	CG7565	chr3L	1.70965337	0.06567862
152870_at	FBgn0003145	CG6716	chr2L	147.838449	1.29E-10
152874_at	FBgn0033559	CG30015	chr2R	65.9269621	5.61E-08
152877_at	FBgn0035315	CG8960	chr3L	5028.26257	1.03E-10
152886_at	FBgn0034602	CG15658	chr2R	107.112263	7.12E-09
152891_at	FBgn0031915	CG12789	chr2L	1.7879561	0.12164174

152907_at	FBgn0004364	CG8896	chr2R	190.014768	2.63E-08
152934_at	FBgn0023520	CG3857	chrX	4.62919156	3.23E-06
152995_at	FBgn0033261	CG18316	chr2R	12.6301387	5.80E-10
153004_at	FBgn0033923	CG8603	chr2R	222.445717	3.28E-08
153031_at	FBgn0038037	CG11466	chr3R	1.28181413	0.50468765
153079_at	FBgn0034214	CG6550	chr2R	3.81474303	1.92E-05
153087_at	FBgn0034479	CG8654	chr2R	12.0928179	0.00027597
153105_at	FBgn0036291	CG10681	chr3L	2.88534782	0.00068766
153106_at	FBgn0031630	CG15629	chr2L	2.46228426	0.01849366
153132_at	FBgn0037815	CG4043	chr3R	5.10539756	3.49E-05
153157_at	FBgn0037213	CG12581	chr3R	190.899469	7.75E-10
153170_at	FBgn0037961	CG31368	chr3R	2.87210354	0.00012571
153189_at	FBgn0035401	CG1291	chr3L	2.63312174	0.00105874
153223_at	FBgn0036191	CG7334	chr3L	2.56767788	0.0129097
153225_at	FBgn0038671	CG31043	chr3R	-1.44885846	0.20107698
153235_at	FBgn0023184	NA	chr2R	3.88655995	0.00489507
153240_at	FBgn0038876	CG5919	chr3R	2.94754773	0.00026267
153247_at	FBgn0032937	CG8674	chr2L	3.21928261	1.22E-05
153260_at	FBgn0026061	CG4123	chr3L	10.7791583	6.25E-06
153274_at	FBgn0027914	CG10670	chr3L	3.14104379	0.00607281
153288_at	FBgn0039489	CG5880	chr3R	3.05657656	3.21E-05
153294_at	FBgn0035048	CG4806	chr2R	3.22342806	5.46E-05
153315_at	FBgn0034050	CG8297	chr2R	2.59225029	0.00096348
153328_at	FBgn0038183	CG9286	chr3R	10.6198795	1.39E-06

153334_at	FBgn0032871	CG2611	chr2L	3.8974253	0.00027621
153356_at	FBgn0032445	CG6153	chr2L	1.26810687	0.32928552
153374_at	FBgn0004646	CG3039	chrX	121.993219	9.73E-11
153389_at	FBgn0036692	CG33522	chr3L	1.66168301	0.32930315
153395_at	FBgn0000229	CG5680	chr2L	1.64571271	0.11048183
153418_at	FBgn0039734	CG7908	chr3R	13.1303507	0.00015142
153424_at	FBgn0013743	CG1056	chr3R	6.87095807	4.63E-05
153432_at	FBgn0022073	CG8846	chr2L	36.3448158	1.10E-09
153515_at	FBgn0002849	CG1897	chr3R	55.5078902	1.57E-05
153535_at	FBgn0031993	CG8486	chr2L	2.93152667	0.00220226
153554_at	FBgn0033182	CG1621	chr2R	16.2319503	4.55E-09
153558_at	FBgn0026320	CG5185	chr3L	515.236182	9.67E-11
153583_at	FBgn0001224	CG4463	chr3L	12.9207998	2.36E-08
153620_at	FBgn0033552	CG12342	chr2R	3.64091867	3.63E-05
153639_at	FBgn0039691	CG1972	chr3R	2.57498299	0.02622311
153641_at	FBgn0036494	CG7250	chr3L	18.2789207	1.33E-05
153671_at	FBgn0020360	CG6219	chr3R	2.27678953	0.00421949
153672_at	FBgn0023521	CG3587	chrX	1.25198233	0.20119732
153693_at	FBgn0031510	CG18555	chr2L	2.50143236	0.00165937
153728_at	FBgn0012037	CG8827	chr2L	50.5545663	4.05E-07
153736_at	FBgn0034496	CG9143	chr2R	2.67497748	0.00196022
153762_at	FBgn0003963	CG2762	chr2L	3.84652399	0.00053645
153777_at	FBgn0037513	CG3027	chr3R	2.26562022	0.00228096
153787_at	FBgn0032848	CG10722	chr2L	3.67756031	0.00290736
153827_at	FBgn0033842	CG5970	chr2R	2.61987455	6.62E-05

153835_at	FBgn0004893	CG10021	chr2L	73.9477538	8.01E-06
153848_at	FBgn0033855	CG13333	chr2R	10536.0486	6.39E-13
153855_at	FBgn0034373	CG15085	chr2R	71.1234624	1.98E-07
153856_at	FBgn0002528	CG3322	chr3L	352.257703	5.85E-09
153867_at	FBgn0000577	CG9015	chr2R	27.3931185	2.92E-05
153892_at	FBgn0036103	CG7958	chr3L	1310.7986	2.84E-11
153951_at	FBgn0001254	CG1934	chr3L	176.388281	3.78E-08
153974_at	FBgn0034109	CG7747	chr2R	4.49592904	1.01E-05
153975_at	FBgn0030418	CG4004	chrX	3.89050703	0.0002712
153981_at	FBgn0037703	CG8165	chr3R	4.95031123	6.48E-05
153984_at	FBgn0026319	CG3048	chr2L	1921.10546	8.43E-11
154010_at	FBgn0027528	CG9634	chrX	4.51525202	1.23E-07
154088_at	FBgn0033381	CG11784	chr2R	7.89358321	9.83E-10
154128_at	FBgn0012051	CG7563	chr2R	4.76419024	0.00022174
154146_at	FBgn0001323	CG4761	chr3L	9.89918403	0.00034499
154158_at	FBgn0034852	CG3496	chr2R	2.39308323	0.00038304
154204_at	FBgn0034572	CG9346	chr2R	3.25813631	0.00059513
154232_at	FBgn0033975	CG10228	chr2R	2.6352572	0.00804505
154252_at	FBgn0002526	CG10236	chr3L	8.76488433	0.01012418
154259_at	FBgn0031846	CG11331	chr2L	3.96749708	1.21E-05
154267_at	FBgn0010417	CG32211	chr3L	3.14775583	1.15E-05
154290_at	FBgn0037186	CG11241	chr3L	3.46231928	2.07E-05
154291_at	FBgn0030550	CG32604	chrX	5.25460125	2.09E-07
154304_at	FBgn0037028	CG3618	chr3L	3.53586494	0.00015292

154318_at	FBgn0033304	CG2397	chr2R	1.85324859	0.24075815
154344_at	FBgn0038948	CG5383	chr3R	2.25749444	0.00028304
154350_at	FBgn0033735	CG8525	chr2R	3.31000203	0.00011532
154356_at	FBgn0001123	CG2835	chr2R	23.1937618	4.40E-06
154376_at	FBgn0036570	CG5222	chr3L	3.47888794	0.00027946
154392_at	FBgn0038672	CG6005	chr3R	1.78517555	0.03173345
154420_at	FBgn0005771	CG4491	chr2L	247.635827	1.16E-09
154426_at	FBgn0028519	CG4500	chr2L	37.1357202	1.54E-11
154435_at	FBgn0038586	CG7168	chr3R	1.57300692	0.03576371
154438_at	FBgn0034988	CG3362	chr2R	2.91818153	1.33E-05
154528_at	FBgn0003002	CG1133	chr3R	11409.6608	1.06E-11
154576_at	FBgn0031017	CG32533	chrX	4.49375038	1.71E-05
154602_at	FBgn0010389	CG7223	chr3R	100.096909	2.06E-07
154658_at	FBgn0031050	CG12235	chrX	1.12160631	0.53984222
154674_at	FBgn0039606	CG1448	chr3R	308.763979	1.78E-14
154691_at	FBgn0034847	CG3502	chr2R	30.1306398	6.34E-07
154716_at	FBgn0015380	CG17348	chr2L	193.832294	3.83E-08
154755_at	FBgn0004878	CG2102	chr3R	30.4495355	2.09E-05
154821_at	FBgn0037699	CG8147	chr3R	407.34649	7.31E-09
154833_at	FBgn0038385	CG4221	chr3R	42.0186845	1.61E-07
154837_at	FBgn0034275	CG5002	chr2R	61.1447297	1.52E-06
154849_at	FBgn0034086	CG8441	chr2R	8.45457766	2.50E-07
154880_at	FBgn0000071	CG2198	chr3R	1207.9449	8.46E-11
154882_at	FBgn0034041	CG30089	chr2R	30.0781919	1.40E-06
154915_at	FBgn0037633	CG9839	chr3R	7.31044375	3.63E-05

154921_at	FBgn0032256	CG6187	chr2L	3.22961739	0.05789564
154929_at	FBgn0039286	CG11849	chr3R	15860.2585	3.78E-12
154934_at	FBgn0034631	CG10496	chr2R	1.76906239	0.04866728
154971_at	FBgn0031320	CG5126	chr2L	2.23643596	0.00066109
154979_at	FBgn0037978	CG3571	chr3R	3.00056589	7.11E-06
154983_at	FBgn0031621	CG15634	chr2L	566.963356	9.25E-10
154994_at	FBgn0034640	CG9858	chr2R	3.82707877	3.92E-05
155061_at	FBgn0031157	CG1503	chrX	1.54272712	0.04503401
155069_at	FBgn0032693	CG10391	chr2L	122.210479	2.39E-08
155072_at	FBgn0035745	CG32386	chr3L	33.9745525	2.38E-08
155075_at	FBgn0035520	CG11586	chr3L	3.2893211	5.66E-05
155092_at	FBgn0034351	CG5190	chr2R	2.72116848	0.00033933
155099_at	FBgn0000180	CG4722	chr2L	3138.76554	3.97E-11
155137_at	FBgn0033891	CG8067	chr2R	1.44048892	0.10857695
