# Supplementary material for: Unmasking Activation of the Zygotic Genome Using Chromosomal Deletions in the Drosophila Embryo
Source: PLoS Biol. 2007 Apr 24;5(5):e117. doi: 10.1371/journal.pbio.0050117 (PMC1854917; doi:10.1371/journal.pbio.0050117)
Supplement: Supplementary file 6 [file pbio.0050117.st006.pdf]

Table S6: Maternal-Zygotic

affy_probe_set_id	fbgn for the probe set	CG id	Chr.Location	fold-changes	p-value
141223_at	FBgn0032017	CG7810	chr2L	-5.088027548	0.000483578
141270_at	FBgn0031291	CG33526	chr2L	-12.17062075	1.46E-06
141296_at	FBgn0031950	CG14536	chr2L	-5.802610172	6.12E-06
141350_at	FBgn0031657	CG3756	chr2L	-4.926090283	1.02E-06
141359_at	FBgn0031590	CG3702	chr2L	-3.375524398	3.29E-10
141487_at	FBgn0003278	CG4033	chr2L	-5.880525505	2.02E-05
141496_at	FBgn0032466	CG5092	chr2L	-3.994881581	2.77E-07
141525_at	FBgn0024245	CG17559	chr2L	-8.568991884	0
141548_at	FBgn0031290	CG4276	chr2L	-28.79648141	1.72E-08
141567_at	FBgn0028894	CG5869	chr2L	-12.22494965	2.21E-11
141598_at	FBgn0032731	CG10641	chr2L	-5.801596049	3.55E-05
141633_at	FBgn0032036	CG13384	chr2L	-25.40581335	1.12E-10
141644_at	FBgn0001309	CG3696	chr2L	-4.352878595	3.33E-16
141679_at	FBgn0031605	CG15438	chr2L	-3.416472386	3.65E-08
141782_at	FBgn0028633	CG2615	chr2L	-3.222173227	2.32E-07
141928_at	GM06264.3prime-hit	NA	chr2L	-4.508608687	2.88E-06
142449_at	FBgn0032205	CG4957	chr2L	-3.32748174	0.000740452
142821_at	FBgn0015772	CG10637	chr2L	-6.137746258	2.88E-08
142875_at	FBgn0032646	CG6412	chr2L	-3.091166932	0.000115976
142899_at	FBgn0032197	CG5694	chr2L	-3.326785221	9.30E-12
142975_at	FBgn0011673	CG7438	chr2L	-3.159890912	1.43E-06
143117_at	FBgn0000413	CG5102	chr2L	-7.713908625	0
143223_at	FBgn0001994	CG7664	chr2L	-3.938191125	1.13E-09
143330_at	FBgn0003310	CG4385	chr2L	-4.7288412	4.66E-15
143377_at	FBgn0003716	CG14026	chr2L	-3.323991563	1.13E-11
143573_at	FBgn0005672	CG10334	chr2L	-70.06461937	1.25E-14
143636_at	FBgn0011202	CG1768	chr2L	-16.79091043	2.80E-10
143663_at	FBgn0011584	CG4758	chr2L	-12.07519499	1.80E-06
143668_at	FBgn0011638	CG10922	chr2L	-8.531417089	2.33E-05
143743_at	FBgn0014015	CG33529	chr2L	-19.87204014	1.24E-09
143774_at	FBgn0015000	CG3469	chr2L	-9.863871721	1.53E-05
143794_at	FBgn0015299	CG8396	chr2L	-4.039645105	3.20E-07
143820_at	FBgn0015567	CG4260	chr2L	-5.117495744	3.12E-05
143833_at	FBgn0015621	CG3642	chr2L	-3.95749928	1.22E-12
143858_at	FBgn0015924	CG4280	chr2L	-6.653246787	0.000182444
144016_at	FBgn0024320	CG5722	chr2L	-4.152978842	1.31E-06
144125_at	FBgn0026379	CG5671	chr2L	-8.07735922	4.29E-06
144234_at	FBgn0028538	CG7578	chr2L	-38.2410798	2.92E-11
144247_at	FBgn0028694	CG18174	chr2L	-9.415799279	5.51E-08
145523_at	FBgn0031243	CG17078	chr2L	-71.94077512	3.91E-12
145649_at	FBgn0031434	CG3227	chr2L	-4.908864877	0
145680_at	FBgn0031475	CG31694	chr2L	-5.571390643	1.30E-10
145713_at	FBgn0031535	CG12795	chr2L	-3.53613419	2.97E-09
145761_at	FBgn0031607	CG15440	chr2L	-3.116810414	1.13E-08
145783_at	FBgn0031636	CG12194	chr2L	-19.05647048	0
145894_at	FBgn0031806	CG9506	chr2L	-35.85515304	0
145947_at	FBgn0031898	CG4567	chr2L	-3.465425309	2.87E-06
146109_at	FBgn0032154	CG5924	chr2L	-3.610470755	4.15E-06
146130_at	FBgn0032194	CG4901	chr2L	-23.31147448	3.67E-08
146131_at	FBgn0032195	CG4908	chr2L	-7.543825368	2.56E-06
146133_at	FBgn0032202	CG18619	chr2L	-12.70030427	1.52E-13
146139_at	FBgn0032217	CG4972	chr2L	-3.554271503	2.01E-07
146156_at	FBgn0032244	CG5313	chr2L	-9.749382467	2.95E-10
146191_at	FBgn0032298	CG6724	chr2L	-6.305098068	9.59E-13

146237_at	FBgn0032355	CG6392	chr2L	-14.97187409	3.44E-07
146263_at	FBgn0032390	CG16969	chr2L	-21.25715629	2.45E-08
146307_at	FBgn0032468	CG9933	chr2L	-16.00081786	2.82E-08
146597_at	FBgn0032960	CG11628	chr2L	-7.33554796	2.61E-12
151005_at	FBgn0040518	CG11628	chr2L	-5.887638368	0.00013749
151611_at	LD03769.3prime-hit	CG5300	chr2L	-3.909221645	5.73E-13
151621_at	LD09511.3prime-hit	CG7466	chr2L	-4.427092488	9.10E-09
151676_at	LD28680.3prime-hit	CG31898	chr2L	-3.499289604	1.79E-05
151682_at	LD29596.3prime-hit	CG1759	chr2L	-10.65092129	2.22E-16
151752_s_at	SD07726.3prime-hit	CG33123	chr2L	-10.50007729	1.28E-05
151787_at	FBgn0032157	CG5899	chr2L	-6.713499004	1.09E-06
151854_at	FBgn0027609	CG15437	chr2L	-4.639697228	7.52E-09
151878_at	FBgn0028541	CG7364	chr2L	-6.04435222	9.55E-11
151989_at	FBgn0031645	CG3036	chr2L	-67.32413863	0
152360_at	FBgn0000675	CG5686	chr2L	-5.62519481	5.61E-09
152413_at	FBgn0022153	CG3054	chr2L	-6.620669698	1.36E-05
152479_at	FBgn0032395	CG6734	chr2L	-5.314842393	5.49E-05
152487_at	FBgn0024371	CG1071	chr2L	-15.03219338	0
152508_at	FBgn0031897	CG13784	chr2L	-14.75847568	0
152525_at	FBgn0010460	CG5461	chr2L	-15.50742626	0
152549_at	FBgn0017551	CG10800	chr2L	-10.67303809	5.55E-09
152652_at	FBgn0028926	CG4185	chr2L	-3.859122019	3.50E-09
152656_at	FBgn0020304	CG3365	chr2L	-3.326782123	1.20E-12
152684_at	FBgn0032358	CG4851	chr2L	-6.606707621	4.51E-14
152727_at	FBgn0031971	CG7224	chr2L	-4.204839094	2.00E-05
152854_at	FBgn0028507	CG3793	chr2L	-4.178662863	4.10E-05
152899_at	FBgn0016930	CG4551	chr2L	-3.053617416	2.21E-05
152978_at	FBgn0031536	CG3248	chr2L	-4.108941838	0.000184786
153172_at	FBgn0032079	CG31886	chr2L	-9.78617054	0.000297174
153191_at	FBgn0032841	CG10728	chr2L	-6.943835107	3.03E-11
153232_at	FBgn0032121	CG33298	chr2L	-10.38134002	5.80E-14
153281_at	FBgn0031661	CG8890	chr2L	-3.14336209	2.30E-10
153299_at	FBgn0032451	CG12292	chr2L	-28.0218796	5.88E-09
153385_at	FBgn0031899	CG4675	chr2L	-15.11713897	0
153597_at	FBgn0024291	CG5216	chr2L	-7.536931519	4.60E-14
153624_at	FBgn0032694	CG15162	chr2L	-71.53164055	2.33E-07
153634_at	FBgn0031911	CG5229	chr2L	-4.335357529	1.44E-09
153717_at	FBgn0031435	CG9883	chr2L	-11.78651761	0
153746_at	FBgn0005683	CG5354	chr2L	-7.103376776	1.35E-13
153768_at	FBgn0032873	CG2614	chr2L	-4.49819195	0.000117004
153866_at	FBgn0031303	CG4710	chr2L	-70.67737187	2.48E-11
153872_at	FBgn0010300	CG10719	chr2L	-4.516031005	1.33E-15
153912_at	FBgn0032691	CG10414	chr2L	-60.24494896	9.50E-11
153943_at	FBgn0031250	CG11907	chr2L	-11.55528308	4.43E-06
153957_at	FBgn0031752	CG9044	chr2L	-12.81603876	2.10E-07
154083_at	FBgn0019890	CG8954	chr2L	-8.65376031	6.66E-16
154102_at	FBgn0032118	CG3766	chr2L	-9.417858696	6.17E-12
154115_at	FBgn0032393	CG12264	chr2L	-4.810512296	9.81E-11
154120_at	FBgn0010382	CG3938	chr2L	-31.66426964	0
154152_at	FBgn0020240	CG7586	chr2L	-7.064813225	0.000158386
154192_at	FBgn0023388	CG1099	chr2L	-6.863809834	1.59E-06
154346_at	FBgn0002973	CG3779	chr2L	-27.27806564	0
154362_at	FBgn0031633	CG2976	chr2L	-3.206071483	4.57E-10
154386_at	FBgn0015600	CG9660	chr2L	-59.19357155	4.42E-14
154411_at	FBgn0019686	CG10895	chr2L	-6.291032755	3.19E-12
154615_at	FBgn0031875	CG3430	chr2L	-11.01374156	1.36E-05
154673_at	FBgn0020616	CG3423	chr2L	-25.75732516	2.32E-10

154677_at	FBgn0011207	CG3959	chr2L	-7.467560387	5.30E-10
154678_at	FBgn0003444	CG11561	chr2L	-4.721163851	2.22E-16
154877_at	FBgn0031769	CG9135	chr2L	-3.240885248	1.29E-08
155059_at	FBgn0032880	CG9318	chr2L	-4.557400982	6.57E-05
155080_at	FBgn0032348	CG4751	chr2L	-13.06916781	1.11E-16
155094_at	FBgn0010229	CG8676	chr2L	-15.1885988	1.33E-08
141244_at	FBgn0033802	CG17724	chr2R	-3.131082083	9.52E-07
141249_at	FBgn0033740	CG8828	chr2R	-4.032925516	4.05E-05
141261_at	FBgn0033454	CG1671	chr2R	-4.429888355	8.16E-08
141451_at	FBgn0035026	CG12252	chr2R	-3.600314517	4.37E-05
141454_at	FBgn0033609	CG13213	chr2R	-48.6347651	2.95E-10
141455_at	FBgn0032886	CG9328	chr2L	-3.047563679	1.39E-08
141467_at	FBgn0003175	CG4444	chr2R	-8.439879628	1.18E-12
141514_at	FBgn0025185	CG1605	chr2R	-3.472117286	5.75E-08
141565_at	FBgn0003701	CG5785	chr2R	-4.047116361	6.47E-10
141591_at	FBgn0023172	CG9635	chr2R	-17.7589146	4.72E-08
141614_at	FBgn0034504	CG8929	chr2R	-7.367951884	1.11E-16
141635_at	FBgn0034964	CG3173	chr2R	-6.421354765	4.75E-13
141716_at	FBgn0022764	CG8815	chr2R	-6.864765104	3.35E-11
141767_at	FBgn0003044	CG5109	chr2R	-7.507348078	5.11E-13
141770_at	FBgn0004055	CG3533	chr2R	-3.455394186	8.67E-05
141783_at	FBgn0033796	CG17054	chr2R	-21.26165337	3.90E-09
141796_at	FBgn0034271	CG4996	chr2R	-6.460075524	0.000196176
141803_at	FBgn0034280	CG11430	chr2R	-23.20396103	1.17E-08
141809_at	FBgn0034447	CG7744	chr2R	-6.738184552	4.11E-08
142064_at	LD41588.3prime-hit	NA	chr2R	-6.393841801	6.92E-06
142143_at	FBgn0016078	CG8804	chr2R	-6.861927625	1.39E-05
142245_at	FBgn0025790	CG10327	chr2R	-16.00025169	1.78E-15
142263_at	FBgn0035075	CG32836	chr2R	-4.777073691	1.06E-06
142296_at	FBgn0028552	CG3988	chr2R	-21.0706243	2.30E-08
142399_at	FBgn0033631	CG9027	chr2R	-3.526979329	5.18E-11
142479_at	FBgn0034342	CG17533	chr2R	-6.175429195	1.56E-05
142509_at	FBgn0033846	CG6061	chr2R	-17.92490027	4.76E-09
142585_at	FBgn0033804	CG17724	chr2R	-8.456548628	8.52E-05
142621_at	FBgn0033974	CG33506	chr2R	-6.763772788	4.06E-05
142653_at	FBgn0034058	CG8315	chr2R	-3.128207383	6.97E-06
142659_at	FBgn0027519	CG8325	chr2R	-7.156362669	1.35E-06
142872_at	FBgn0011704	CG8975	chr2R	-17.02038721	0
142919_s_at	FBgn0033398	CG2049	chr2R	-5.725508787	4.94E-10
143000_at	FBgn0032444	CG5525	chr2L	-4.382909009	3.64E-11
143080_at	FBgn0000142	CG8787	chr2R	-5.88532481	1.74E-06
143231_at	FBgn0002525	CG6944	chr2L	-3.720162494	9.02E-11
143303_at	FBgn0003082	CG11205	chr2R	-4.423103843	1.11E-05
143352_at	FBgn0003391	CG3722	chr2R	-3.570681236	1.71E-09
143380_at	FBgn0003732	CG10223	chr2L	-4.224178337	2.59E-10
143558_at	FBgn0005624	CG3886	chr2R	-56.62084153	0
143631_at	FBgn0010638	CG10130	chr2R	-9.78635983	4.44E-16
143665_at	FBgn0011592	CG8581	chr2R	-7.690035282	1.11E-16
143721_at	FBgn0013765	CG4832	chr2R	-9.225847902	3.33E-16
143753_at	FBgn0014151	CG5403	chr2R	-5.714580356	5.66E-15
143772_at	FBgn0014870	CG8912	chr2R	-3.443837495	1.63E-07
143886_at	FBgn0017414	CG12346	chr2R	-3.126281553	0.00014486
143907_at	FBgn0019968	CG8183	chr2R	-34.11820734	5.46E-09
145515_at	FBgn0031227	CG3709	chr2L	-7.25382627	5.77E-06
145520_at	FBgn0031238	CG3645	chr2L	-3.022302885	0.00025563
145561_at	FBgn0031309	CG5041	chr2L	-4.226992664	4.27E-09
145664_at	FBgn0031453	CG9894	chr2L	-4.764312856	1.41E-12

145681_at	FBgn0031477	CG31694	chr2L	-3.47574739	4.64E-08
145747_at	FBgn0031583	CG12676	chr2L	-4.220838058	9.03E-07
146045_at	FBgn0032060	CG9298	chr2L	-15.55041476	6.14E-09
146137_at	FBgn0032210	CG5603	chr2L	-7.718532093	0.000112002
146264_s_at	FBgn0032392	CG12264/CG31	chr2L	-4.283654364	2.57E-07
146745_at	FBgn0033183	CG1620	chr2R	-7.325241186	2.93E-07
146780_at	FBgn0033242	CG2916	chr2R	-53.78180745	0
146788_at	FBgn0033259	CG11210	chr2R	-3.085793627	2.05E-06
146833_at	FBgn0033329	CG14767	chr2R	-4.790912057	7.27E-09
146871_at	FBgn0033392	CG8027	chr2R	-4.140084378	0.000126774
146913_at	FBgn0033468	CG1418	chr2R	-9.963345891	1.77E-06
146935_at	FBgn0033506	CG3298	chr2R	-19.49422756	3.35E-07
146946_s_at	FBgn0033518	CG12405/CG12	chr2R	-5.302440144	3.26E-14
146951_at	FBgn0033526	CG12892	chr2R	-5.867456141	1.08E-11
146953_at	FBgn0033528	CG11761	chr2R	-6.345010099	1.29E-08
147023_at	FBgn0033638	CG9005	chr2R	-4.339045242	2.82E-05
147079_at	FBgn0033714	CG8487	chr2R	-13.96701084	8.66E-07
147081_at	FBgn0033718	CG13162	chr2R	-32.189997	7.69E-09
147123_at	FBgn0033784	CG13322	chr2R	-6.856614864	2.32E-05
147130_at	FBgn0033795	CG17054	chr2R	-24.09636488	1.46E-08
147246_at	FBgn0034016	CG8171	chr2R	-4.363352711	1.58E-10
147272_at	FBgn0034062	CG8388	chr2R	-4.237423642	6.87E-05
147278_at	FBgn0034073	CG8414	chr2R	-33.96201356	2.22E-09
147342_at	FBgn0034170	CG33456	chr2R	-14.34332525	3.75E-09
147361_at	FBgn0034210	CG6568	chr2R	-6.930620788	6.10E-05
147376_at	FBgn0034240	CG4903	chr2R	-3.238042096	3.92E-09
147380_at	FBgn0034249	CG6477	chr2R	-13.27369686	1.47E-07
147434_at	FBgn0034334	CG17522	chr2R	-64.06891216	1.03E-11
147511_at	FBgn0034465	CG9854	chr2R	-95.65693299	0
147558_at	FBgn0034527	CG9945	chr2R	-4.811716466	2.35E-08
147592_at	FBgn0034570	CG10543	chr2R	-3.515191478	7.92E-08
147674_at	FBgn0034726	CG11301	chr2R	-30.43690626	4.06E-09
147766_at	FBgn0034849	CG3500	chr2R	-4.236204171	1.60E-07
147831_at	FBgn0034963	CG13567	chr2R	-3.418062792	0.000115767
151517_at	GH13170.3prime-hit	CG5373	chr2R	-5.790724694	4.29E-05
151545_at	GH20047.3prime-hit	CG8205	chr2R	-7.784391868	1.58E-13
151573_at	GM02568.3prime-hit	CG13344	chr2R	-5.854669202	6.30E-07
151647_at	LD17611.3prime-hit	CG33505	chr2R	-13.66479756	9.38E-07
151829_at	FBgn0034088	CG8445	chr2R	-6.457275331	7.50E-06
151910_at	FBgn0019886	CG4589	chr2R	-19.49644908	6.94E-10
152018_at	FBgn0033522	CG11763	chr2R	-3.100389125	1.62E-07
152198_at	FBgn0027561	CG18659	chr2R	-4.558712157	8.84E-05
152264_at	FBgn0033988	CG7761	chr2R	-23.1544272	2.91E-14
152427_at	FBgn0003353	CG3182	chr2R	-5.849458328	1.36E-06
152430_at	FBgn0033780	CG3845	chr2R	-3.379576237	2.79E-07
152444_at	FBgn0011236	CG5575	chr2R	-15.69390656	2.42E-08
152450_at	FBgn0031487	CG3558	chr2L	-6.533067289	1.75E-07
152461_at	FBgn0033470	CG12131	chr2R	-7.409995382	3.45E-08
152515_at	FBgn0003074	CG8251	chr2R	-4.721422994	2.19E-05
152598_at	FBgn0034335	CG5164	chr2R	-48.50174532	0
152638_at	FBgn0034987	CG3363	chr2R	-7.624147106	7.66E-15
152663_at	FBgn0033272	CG8707	chr2R	-6.623970892	6.50E-11
152719_at	FBgn0025830	CG5859	chr2R	-16.88741753	2.37E-07
152792_at	FBgn0031987	CG12375	chr2L	-18.98534526	2.03E-07
152868_at	FBgn0033636	CG10897	chr2R	-6.855809925	4.61E-13
152896_at	FBgn0003396	CG7734	chr2R	-34.97482251	9.65E-12
152964_at	FBgn0033915	CG8485	chr2R	-3.666152815	3.28E-09

152991_at	FBgn0034349	CG5186	chr2R	-51.1379384	2.00E-10
153061_at	FBgn0035055	CG30421	chr2R	-16.81553467	2.55E-07
153112_at	FBgn0034946	CG3065	chr2R	-7.034604257	7.74E-07
153131_at	FBgn0033241	CG2915	chr2R	-6.140474488	1.11E-15
153163_at	FBgn0014877	CG6155	chr2R	-7.161816522	1.88E-11
153179_at	FBgn0033486	CG11866	chr2R	-19.10738439	2.32E-08
153245_at	FBgn0034982	CG4065	chr2R	-3.611654933	0.000332991
153246_at	FBgn0033209	CG12107	chr2R	-5.330592584	2.94E-05
153279_at	FBgn0027912	CG9035	chr2R	-9.178382315	4.09E-09
153329_at	FBgn0033637	CG9006	chr2R	-40.44892239	2.80E-10
153387_at	FBgn0033583	CG7736	chr2R	-4.569221111	9.59E-07
153451_at	FBgn0013762	CG8203	chr2R	-6.445438907	6.12E-05
153473_at	FBgn0033181	CG1624	chr2R	-4.597114461	1.24E-12
153493_at	FBgn0034068	CG8400	chr2R	-3.182741629	1.69E-08
153569_at	FBgn0032165	CG31712	chr2L	-4.89930918	3.11E-10
153579_at	FBgn0011703	CG5371	chr2L	-5.303145111	3.87E-12
153632_at	FBgn0033117	CG3358	chr2R	-4.482072351	5.14E-09
153659_at	FBgn0000054	CG15845	chr2R	-30.14396129	2.72E-11
153662_at	FBgn0034217	CG18468	chr2R	-3.550924297	0.000580962
153667_at	FBgn0027885	CG6582	chr2L	-3.100591909	3.67E-09
153737_at	FBgn0022772	CG10667	chr2R	-4.061175299	4.14E-09
153809_at	FBgn0014269	CG15107	chr2R	-119.5483233	3.17E-12
153961_at	FBgn0010314	CG3738	chr2L	-3.503950505	4.33E-09
153986_at	FBgn0034083	CG8434	chr2R	-12.08236806	4.23E-07
153993_at	FBgn0033094	CG3403	chr2R	-5.559633489	3.43E-12
154004_at	FBgn0034970	CG4005	chr2R	-5.080423348	1.13E-08
154028_at	FBgn0031876	CG9200	chr2L	-3.335447927	3.44E-06
154119_at	FBgn0027507	CG1344	chr2R	-3.450112832	3.94E-10
154147_at	FBgn0034585	CG4030	chr2R	-4.905420821	1.57E-08
154185_at	FBgn0000578	CG15112	chr2R	-6.291422514	5.06E-12
154213_at	FBgn0033992	CG11798	chr2R	-12.80328376	1.36E-13
154234_at	FBgn0023081	CG4012	chr2R	-4.487194211	2.17E-11
154247_at	FBgn0034961	CG3163	chr2R	-3.00361908	2.15E-07
154254_at	FBgn0034650	CG10318	chr2R	-18.43770033	2.44E-08
154285_at	FBgn0034158	CG5522	chr2R	-7.085436254	3.00E-15
154394_at	FBgn0031598	CG3338	chr2L	-5.522016198	1.73E-06
154434_at	FBgn0032481	CG16972	chr2L	-5.785642644	0.000632709
154470_at	FBgn0033859	CG6197	chr2R	-5.792136869	1.78E-05
154485_at	FBgn0001301	CG7210	chr2L	-3.084833079	4.34E-09
154495_at	FBgn0034259	CG6459	chr2R	-7.888017089	8.77E-07
154622_at	FBgn0033616	CG7745	chr2R	-6.776904238	3.32E-05
154643_at	FBgn0031884	CG13777	chr2L	-3.161020585	2.48E-07
154653_at	FBgn0010238	CG12369	chr2R	-5.08222935	1.55E-15
154771_at	FBgn0033413	CG8806	chr2R	-4.292285339	6.78E-08
154794_at	FBgn0032259	CG6144	chr2L	-3.24185677	6.57E-07
154892_at	FBgn0034488	CG11208	chr2R	-18.67736621	7.36E-10
154954_at	FBgn0015295	CG18247	chr2R	-4.082016865	5.21E-05
154966_at	FBgn0034036	CG33139	chr2R	-5.682145174	1.09E-11
154998_at	FBgn0010356	CG7704	chr2R	-3.060469534	3.21E-08
155001_at	FBgn0033677	CG8321	chr2R	-5.593762954	0.000188141
155031_at	FBgn0033346	CG11770	chr2R	-4.022003275	2.08E-06
155070_at	FBgn0033088	CG3271	chr2R	-4.028572068	1.79E-09
155073_at	FBgn0028563	CG8714	chr2R	-8.005655275	9.72E-06
155120_at	FBgn0034643	CG10321	chr2R	-4.762800371	0.000121297
155123_at	FBgn0015907	CG13425	chr2R	-3.147924459	5.91E-12
155155_at	FBgn0033569	CG12942	chr2R	-5.8058416	8.31E-07
141206_at	FBgn0025725	CG7961	chr3L	-5.563806719	4.80E-08

141225_at	FBgn0039013	CG4813	chr3R	-3.491153957	1.50E-06
141231_at	FBgn0028688	CG5378	chr3R	-6.360254782	6.55E-10
141254_at	FBgn0037360	CG2182	chr3R	-6.01796248	6.89E-09
141265_at	FBgn0036033	CG6757	chr3L	-5.81684775	1.18E-05
141268_at	FBgn0035394	CG32486	chr3L	-11.73736104	6.94E-11
141276_at	FBgn0022359	CG4649	chr3R	-3.32115313	1.11E-06
141302_at	FBgn0037368	CG1239	chr3R	-44.64333593	1.26E-06
141370_at	FBgn0027945	CG7758	chr3L	-25.291461	2.98E-06
141376_at	FBgn0037795	CG6264	chr3R	-26.21595257	2.68E-05
141413_at	FBgn0038364	CG4699	chr3R	-8.058319468	4.07E-09
141425_at	FBgn0038810	CG5434	chr3R	-14.91007016	1.21E-08
141452_at	FBgn0035137	CG1233	chr3L	-9.6923607	4.00E-07
141477_at	FBgn0038318	CG6236	chr3R	-3.567062435	5.51E-05
141490_at	FBgn0039146	CG6057	chr3R	-4.069799938	3.00E-05
141508_at	FBgn0037120	CG11247	chr3L	-79.003008	2.16E-06
141509_at	FBgn0035145	CG12031	chr3L	-14.89818529	7.23E-07
141511_at	FBgn0016794	CG1044	chr3L	-16.33201045	5.18E-07
141524_at	FBgn0035688	CG10289	chr3L	-33.03407054	3.84E-11
141546_at	FBgn0037250	CG1074	chr3R	-3.183830048	2.16E-06
141551_at	FBgn0035987	CG3689	chr3L	-50.5630764	1.80E-07
141569_at	FBgn0039122	CG10192	chr3R	-4.881906375	7.32E-07
141572_at	FBgn0039590	CG10011	chr3R	-11.6802667	6.97E-05
141606_at	FBgn0028736	CG1333	chr3L	-3.846207491	2.11E-05
141611_at	FBgn0037373	CG2095	chr3R	-6.003608604	6.48E-09
141612_at	FBgn0036917	CG8013	chr3L	-211.2518911	2.24E-08
141622_at	FBgn0036558	CG5841	chr3L	-3.926827001	1.38E-07
141628_at	FBgn0024832	CG7057	chr3R	-71.3395788	1.94E-07
141647_at	FBgn0035436	CG12016	chr3L	-478.0379588	2.75E-09
141652_at	FBgn0036772	CG5290	chr3L	-13.82178696	0.000298413
141659_at	FBgn0038146	CG9799	chr3R	-4.090687215	0.000223968
141663_at	FBgn0036668	CG9715	chr3L	-4.504209986	9.32E-10
141666_at	FBgn0039279	CG11844	chr3R	-12.76497947	5.70E-08
141683_at	FBgn0038872	CG5874	chr3R	-67.2086695	1.80E-11
141685_at	FBgn0037515	CG3066	chr3R	-3.380538267	0.000122858
141693_at	FBgn0038168	CG9591	chr3R	-13.24647055	6.83E-07
141737_at	FBgn0036912	CG8491	chr3L	-23.47571543	2.10E-06
141763_at	FBgn0035235	CG7879	chr3L	-33.01744764	2.06E-06
141781_at	FBgn0038578	CG7957	chr3R	-9.427091067	3.10E-08
141788_at	FBgn0037622	CG8202	chr3R	-4.757924976	0.000809871
141791_at	FBgn0010379	CG4006	chr3R	-5.815484977	1.43E-07
141798_at	FBgn0025802	CG6939	chr3R	-21.8974673	5.21E-05
141800_at	FBgn0035149	CG17183	chr3L	-100.8679287	1.65E-07
142179_s_at	FBgn0028495	CG18616	chr3R	-3.093283088	0.000130908
142207_at	FBgn0039189	CG18528	chr3R	-15.27936156	1.72E-05
142481_at	FBgn0037810	CG12819/CG12	chr3R	-4.748134472	2.27E-07
142483_at	FBgn0035846	CG7487	chr3L	-13.8250443	2.54E-05
142488_s_at	FBgn0037930	CG14715	chr3R	-3.653270867	7.07E-09
142517_at	FBgn0035128	CG1225	chr3L	-62.8842812	1.26E-06
142531_at	FBgn0004926	CG4153	chr3L	-7.247608731	6.65E-10
142545_at	FBgn0010173	CG9633	chr3R	-41.12185484	2.05E-07
142599_at	FBgn0039011	CG31151	chr3R	-3.067002323	0.000181752
142672_at	FBgn0039099	CG10157	chr3R	-7.546758784	1.22E-11
142675_at	FBgn0027512	CG10254	chr3R	-42.2416592	8.61E-07
142708_at	FBgn0039558	CG4980	chr3R	-3.445002098	2.54E-08
142718_at	FBgn0027500	CG17286	chr3L	-4.21122399	7.46E-08
142743_at	FBgn0015618	CG10572	chr3L	-3.475907749	1.63E-05
142758_at	FBgn0024326	CG9738	chr3R	-6.314421661	1.00E-07

142771_at	FBgn0038235	CG8461	chr3R	-3.59773839	5.52E-07
142820_at	FBgn0001139	CG8384	chr3R	-5.979868371	2.24E-10
142905_at	FBgn0036052	CG10809	chr3L	-6.237093993	7.01E-08
142911_at	FBgn0035614	CG10622	chr3L	-3.491104306	1.74E-07
142913_at	FBgn0002932	CG11988	chr3R	-15.07547707	4.35E-06
142992_at	FBgn0035236	CG12004	chr3L	-16.63936376	2.38E-10
142994_at	FBgn0038253	CG31385	chr3R	-5.369900274	1.76E-05
142999_at	FBgn0010280	CG5444	chr3L	-17.94889565	5.01E-12
143147_at	FBgn0000565	CG7266	chr3L	-5.630391093	8.02E-06
143176_at	FBgn0001099	CG33088	chr3L	-8.215797454	1.35E-09
143177_at	FBgn0001108	CG9206	chr3L	-3.64809726	0.000137747
143198_at	FBgn0001233	CG1242	chr3L	-8.193411573	6.14E-13
143225_at	FBgn0002183	CG1828	chr3L	-4.430060641	1.18E-06
143226_at	FBgn0002354	CG7620	chr3R	-9.090600222	4.45E-08
143269_at	FBgn0002781	CG32491	chr3R	-4.27337597	1.76E-07
143293_at	FBgn0003041	CG8114	chr3L	-71.79245411	5.11E-15
143328_at	FBgn0003292	CG6097	chr3L	-12.85316931	3.34E-06
143335_at	FBgn0003334	CG9495	chr3R	-24.81029375	6.27E-06
143384_at	FBgn0003862	CG8651	chr3R	-8.956433936	2.45E-08
143547_at	FBgn0005585	CG9429	chr3R	-5.994418869	4.16E-11
143575_at	FBgn0005696	CG5923	chr3R	-72.01755524	2.54E-07
143597_at	FBgn0010333	CG2248	chr3L	-3.37556332	1.46E-06
143628_at	FBgn0010438	CG4337	chr3R	-6.853484031	2.68E-09
143657_at	FBgn0011336	CG7748	chr3R	-16.1016926	1.10E-13
143708_at	FBgn0013347	CG5163	chr3R	-3.145417657	3.56E-08
143738_at	FBgn0013998	CG33101	chr3R	-3.754124903	0.000150459
143740_at	FBgn0014006	CG4720	chr3R	-9.162751979	0.000330001
143744_at	FBgn0014023	CG9378	chr3R	-8.728859265	1.40E-06
143802_at	FBgn0015351	CG14906	chr3R	-3.900062786	3.99E-06
143808_at	FBgn0015513	CG10379	chr3R	-99.45515277	1.20E-13
143844_at	FBgn0015793	CG7062	chr3L	-98.71989824	2.23E-07
143848_at	FBgn0015806	CG10539	chr3L	-5.92934968	3.05E-09
143851_at	FBgn0015829	CG1276	chr3L	-3.900657865	3.58E-07
143981_at	FBgn0023395	CG9594	chr3L	-4.016423365	0.000104897
144063_at	FBgn0025456	CG5413	chr3R	-7.144856462	2.23E-08
144123_at	FBgn0026371	CG7186	chr3L	-5.769345631	1.32E-09
147914_at	FBgn0035116	CG32346	chr3L	-7.751258544	0.000732266
147985_at	FBgn0035229	CG7852	chr3L	-79.9302359	3.43E-07
148003_at	FBgn0035272	CG13922	chr3L	-17.59488329	1.21E-06
148040_at	FBgn0035334	CG8993	chr3L	-14.33159716	0.000126349
148054_at	FBgn0035355	CG16985	chr3L	-4.047349316	4.38E-06
148067_at	FBgn0035374	CG2101	chr3L	-5.069256636	1.52E-08
148076_at	FBgn0035390	CG1893	chr3L	-3.951152461	3.86E-07
148165_at	FBgn0035526	CG1316	chr3L	-5.89679293	1.44E-10
148166_at	FBgn0035529	CG1319	chr3L	-3.807505917	4.70E-07
148168_at	FBgn0035531	CG32250	chr3L	-3.731969852	2.17E-08
148169_at	FBgn0035532	CG15014	chr3L	-5.352539267	1.34E-07
148173_at	FBgn0035541	CG15019	chr3L	-13.32683174	5.74E-11
148218_at	FBgn0035603	CG10635	chr3L	-5.300579003	1.38E-08
148260_at	FBgn0035674	CG13295	chr3L	-27.98196991	2.17E-05
148283_at	FBgn0035704	CG10144	chr3L	-7.682180146	2.55E-07
148369_at	FBgn0035830	CG8209	chr3L	-3.43717647	7.09E-07
148394_at	FBgn0035877	CG7083	chr3L	-22.57290467	2.62E-06
148413_at	FBgn0035908	CG6817	chr3L	-4.127003674	4.08E-07
148469_at	FBgn0036007	CG3424	chr3L	-67.62847262	4.03E-08
148557_at	FBgn0036135	CG7636	chr3L	-20.43201644	3.28E-08
148582_r_at	FBgn0036171	CG11711	chr3L	-17.8502798	0.000176285

148604_at	FBgn0036207	CG10907	chr3L	-3.12633758	3.45E-05
148672_at	FBgn0036308	CG32113	chr3L	-7.171094959	3.62E-05
148676_at	FBgn0036317	CG10948	chr3L	-27.05566069	0.000657879
148768_at	FBgn0036450	CG13472	chr3L	-14.26757989	1.20E-10
148839_at	FBgn0036556	CG5830	chr3L	-3.313540721	2.23E-05
148854_at	FBgn0036581	CG5057	chr3L	-3.567289898	6.86E-09
148886_at	FBgn0036613	CG13037	chr3L	-10.79107029	6.36E-06
148901_at	FBgn0036642	CG4169	chr3L	-4.298103534	5.10E-08
148903_at	FBgn0036644	CG4195	chr3L	-29.48022257	1.19E-09
148914_at	FBgn0036660	CG13025	chr3L	-8.128087139	4.45E-05
148949_at	FBgn0036720	CG32174	chr3L	-4.055134231	8.94E-05
148950_at	FBgn0036721	CG3874	chr3L	-14.81814561	1.57E-11
148966_at	FBgn0036745	CG7484	chr3L	-7.283564906	5.37E-08
149034_at	FBgn0036853	CG9730	chr3L	-9.244024976	5.65E-06
149035_at	FBgn0036855	CG14084	chr3L	-3.843514962	4.37E-06
149078_at	FBgn0036918	CG7770	chr3L	-3.632413332	4.20E-06
149080_at	FBgn0036922	CG14182	chr3L	-38.62789049	1.19E-06
149106_at	FBgn0036958	NA	chr3L	-4.568342123	6.19E-07
149123_at	FBgn0036983	CG5408	chr3L	-8.684499308	2.22E-11
149126_at	FBgn0036989	CG5231	chr3L	-3.459653925	0.000189911
149129_at	FBgn0036994	CG5199	chr3L	-3.563836621	0.000688948
149143_at	FBgn0037017	CG4074	chr3L	-188.2833678	4.33E-08
149147_at	FBgn0037025	CG11451	chr3L	-15.7890098	0.000606525
149168_at	FBgn0037061	CG12975	chr3L	-4.410448112	1.22E-05
149195_at	FBgn0037110	CG14577	chr3L	-3.007865339	0.00017707
149220_at	FBgn0037149	CG14561	chr3L	-7.072018204	1.45E-11
149347_at	FBgn0037363	CG1347	chr3R	-86.93991643	5.76E-08
149426_at	FBgn0037480	CG10061	chr3R	-8.770610527	0.000866227
149495_at	FBgn0037583	CG9684	chr3R	-51.52854862	3.02E-06
149544_at	FBgn0037669	CG9740	chr3R	-4.772010865	5.43E-10
149547_at	FBgn0037674	CG8454	chr3R	-17.02736041	1.67E-05
149574_at	FBgn0037729	CG8362	chr3R	-13.82068412	1.82E-05
149625_at	FBgn0037802	CG6284	chr3R	-9.720315607	0.000218036
149633_at	FBgn0037819	CG14688	chr3R	-6.074089249	4.16E-09
149681_at	FBgn0037890	CG17734	chr3R	-4.64939551	4.43E-07
149683_at	FBgn0037893	CG6719	chr3R	-4.321398319	6.47E-08
149696_at	FBgn0037922	CG14711	chr3R	-18.14643944	1.88E-05
149751_s_at	FBgn0038015	CG10042	chr3R	-20.30891884	3.78E-05
149779_at	FBgn0038053	CG18549	chr3R	-4.290767838	2.56E-06
149853_at	FBgn0038166	CG9588	chr3R	-4.313592583	0.000198525
149866_at	FBgn0038192	CG9924	chr3R	-3.628713485	1.40E-07
149907_r_at	FBgn0038243	CG8066	chr3R	-17.20630164	0.000123743
149940_at	FBgn0038302	CG4210	chr3R	-15.62019155	3.56E-05
149947_at	FBgn0038314	CG14865	chr3R	-3.698977579	6.52E-06
149979_at	FBgn0038369	CG4560	chr3R	-18.85571185	7.74E-09
150001_at	FBgn0038406	CG6046	chr3R	-3.164106608	1.21E-08
150016_at	FBgn0038430	CG14895	chr3R	-7.077932511	1.11E-09
150135_at	FBgn0038609	CG7671	chr3R	-35.27692845	1.13E-09
150269_at	FBgn0038813	CG5206	chr3R	-41.1006593	9.19E-06
150308_at	FBgn0038875	CG6056	chr3R	-7.196506567	3.36E-12
150345_at	FBgn0038933	CG31163	chr3R	-5.297178761	7.37E-07
150389_at	FBgn0039000	CG6954	chr3R	-28.15555992	1.32E-06
150435_at	FBgn0039065	CG4449	chr3R	-12.23563491	1.58E-06
150436_at	FBgn0039067	CG4448	chr3R	-46.30991082	2.85E-06
150466_at	FBgn0039111	CG10371	chr3R	-5.327160686	2.16E-07
150476_at	FBgn0039138	CG31184	chr3R	-3.090338923	2.81E-07
150477_at	FBgn0039139	CG5933	chr3R	-10.81097818	1.14E-09

150481_at	FBgn0039149	CG18428	chr3R	-3.092735731	6.99E-07
150515_at	FBgn0039204	CG6607	chr3R	-7.405020183	3.10E-05
150516_at	FBgn0039205	CG13623	chr3R	-8.322958786	7.01E-10
150518_at	FBgn0039216	CG31126	chr3R	-5.428562747	2.66E-07
150520_at	FBgn0039218	CG13628	chr3R	-3.039761087	1.23E-08
150544_at	FBgn0039258	CG11780	chr3R	-5.827547924	0.000462671
150551_at	FBgn0039271	CG11839	chr3R	-4.013695292	0.000231873
150552_at	FBgn0039273	CG9996	chr3R	-109.1093104	1.12E-07
150590_at	FBgn0039337	CG5121	chr3R	-3.564704137	9.88E-06
150602_at	FBgn0039357	CG4743	chr3R	-3.026929979	2.75E-05
150641_at	FBgn0039404	CG14543	chr3R	-24.42600206	3.97E-06
150691_at	FBgn0039461	CG5500	chr3R	-3.992255829	0.000406601
150758_at	FBgn0039559	CG4976	chr3R	-13.73185748	1.56E-08
150761_at	FBgn0039565	CG4884	chr3R	-3.617888067	4.46E-06
150841_at	FBgn0039692	CG1973	chr3R	-7.900969693	0.000420313
150865_at	FBgn0039732	CG15525	chr3R	-3.314925452	2.86E-07
151021_at	FBgn0040534	CG11985	chr3R	-4.693200423	6.44E-08
151063_at	FBgn0040575	CG15922	chr3R	-3.065963799	3.75E-06
151087_at	FBgn0040602	CG14545	chr3R	-6.520780077	2.21E-05
151494_at	GH06422.3prime-hit	CR32477	chr3L	-39.85956149	8.57E-12
151529_at	GH15453.3prime-hit	CG7507	chr3L	-3.183667276	0.000749585
151585_at	GM06601.3prime-hit	CG32438	chr3L	-3.416451892	1.21E-05
151586_at	GM06806.3prime-hit	CG7369	chr3L	-21.77439209	4.65E-08
151636_at	LD12912.3prime-hit	CG8582	chr3L	-3.73354021	7.88E-08
151637_at	LD13050.3prime-hit	NA	chr3L	-4.879000661	5.65E-06
151661_s_at	LD22004.3prime-hit	CG9704	chr3L	-24.92601617	5.55E-16
151668_r_at	LD27185.3prime-hit	CG6673	chr3L	-13.16841817	0.000668353
151681_at	LD29569.3prime-hit	CG32306	chr3L	-13.68170051	2.16E-12
151728_at	LP02352.3prime-hit	CG18596	chr3R	-14.14516905	7.97E-05
151737_at	SD01201.3prime-hit	CG32210	chr3L	-4.190405401	1.30E-07
151742_at	SD03655.3prime-hit	CG12004	chr3L	-72.06371942	2.58E-08
151747_s_at	SD04735.3prime-hit	CG5444	chr3L	-8.221880879	9.60E-11
151758_at	SD09884.3prime-hit	CG11100	chr3L	-13.35591637	8.29E-05
151759_s_at	SD10401.3prime-hit	CG12819/CG12	chr3R	-8.344690566	2.38E-07
151761_at	SD10848.3prime-hit	CG3743	chr3L	-5.193384591	7.81E-08
151794_at	FBgn0028399	CG4672	chr3L	-3.547026246	1.10E-07
151804_at	FBgn0037637	CG9836	chr3R	-26.91861281	2.80E-07
151807_at	FBgn0039537	CG5590	chr3R	-3.488746293	6.33E-06
151814_at	FBgn0035714	CG8549	chr3L	-10.78693623	1.73E-07
151840_at	FBgn0027615	CG6404	chr3L	-3.8306859	0.0006898
151904_at	FBgn0027598	CG31012	chr3R	-22.02426964	7.74E-09
151918_s_at	FBgn0027594	CG2086	chr3L	-13.15174488	7.63E-09
151926_at	FBgn0039348	CG4673	chr3R	-5.672507834	3.53E-08
151934_at	FBgn0028500	CG9063	chr3L	-6.277636121	1.46E-08
151982_at	FBgn0037747	CG8481	chr3R	-11.97316384	2.60E-05
152008_at	FBgn0039790	CG2246	chr3R	-4.489591617	0.000542726
152037_at	FBgn0035889	CG33057/CG71	chr3L	-50.01951083	2.36E-06
152067_at	FBgn0004913	CG1119	chr3R	-5.162263328	0.000238041
152158_at	FBgn0038877	CG3308	chr3R	-4.266331068	0.000177148
152177_at	FBgn0027565	CG5498	chr3L	-3.453314027	0.000987841
152192_at	FBgn0015622	CG11958	chr3R	-3.775124926	8.47E-09
152207_at	FBgn0038108	CG7518	chr3R	-4.272584989	1.34E-05
152215_at	FBgn0028487	CG9611	chr3R	-3.144517216	4.72E-07
152237_at	FBgn0038301	CG6654	chr3R	-4.098402977	0.000173738
152253_at	FBgn0037216	CG12582	chr3R	-47.40099279	3.61E-07
152287_at	FBgn0026208	CG4143	chr3L	-8.653153666	8.24E-10
152311_at	FBgn0037049	CG32434	chr3L	-38.98359738	5.72E-14

152314_at	FBgn0036512	CG16979	chr3L	-3.957512464	0.000591098
152324_at	FBgn0037136	CG7148	chr3L	-6.132982978	8.94E-08
152327_at	FBgn0028484	CG14992	chr3L	-199.2751464	7.53E-09
152354_at	FBgn0039714	CG7816	chr3R	-3.230091382	1.56E-05
152355_at	FBgn0015828	CG10415	chr3L	-4.872408263	1.20E-07
152383_at	FBgn0000116	CG32031	chr3L	-14.88804659	2.09E-08
152411_at	FBgn0037439	CG10286	chr3R	-7.83036737	3.19E-06
152417_at	FBgn0028684	CG10370	chr3R	-4.007512478	5.26E-08
152420_at	FBgn0022787	CG4261	chr3R	-12.49835514	5.22E-07
152443_at	FBgn0036253	CG11534	chr3L	-3.255572944	6.44E-05
152465_at	FBgn0017453	CG5215	chr3L	-10.80439579	1.21E-07
152530_at	FBgn0015270	CG3041	chr3R	-58.71430047	2.12E-06
152539_at	FBgn0037378	CG2046	chr3R	-6.921413285	8.53E-08
152563_at	FBgn0038803	CG5191	chr3R	-5.614024679	2.92E-08
152566_at	FBgn0037261	CG9775	chr3R	-63.94203182	2.00E-15
152621_at	FBgn0036366	CG10133	chr3L	-9.590796688	0.000106456
152631_at	FBgn0003691	CG12284	chr3L	-5.214562733	1.23E-09
152640_at	FBgn0035237	CG13917	chr3L	-64.380826	9.60E-08
152706_at	FBgn0038617	CG12333	chr3R	-13.07501121	1.86E-05
152720_at	FBgn0037438	CG31367	chr3R	-3.109238098	4.35E-07
152735_at	FBgn0036136	CG6233	chr3L	-25.34204813	5.63E-07
152738_at	FBgn0027948	CG5000	chr3R	-26.85363808	1.03E-12
152758_at	FBgn0037709	CG8199	chr3R	-4.220302656	0.000106249
152769_at	FBgn0039715	CG7814	chr3R	-4.818399315	5.39E-05
152771_at	FBgn0039266	CG11791	chr3R	-31.89177018	5.60E-11
152793_at	FBgn0037356	CG12170	chr3R	-26.77501368	4.38E-06
152811_at	FBgn0035244	CG7955	chr3L	-4.793470536	0.000197514
152812_at	FBgn0037663	CG9746	chr3R	-11.16000154	4.38E-07
152837_at	FBgn0039349	CG4685	chr3R	-6.008342453	6.76E-06
152845_at	FBgn0036405	CG6833	chr3L	-4.765046092	1.58E-07
152846_at	FBgn0010313	CG2530	chr3R	-3.103429506	1.52E-06
152864_at	FBgn0039135	CG13603	chr3R	-7.978225771	2.11E-07
152898_at	FBgn0011716	CG1539	chr3R	-156.5226443	7.79E-10
152914_at	FBgn0036684	CG3764	chr3L	-22.75693077	5.60E-09
152917_at	FBgn0036137	CG7628	chr3L	-3.756365267	4.47E-06
152918_at	FBgn0037308	CG31543	chr3R	-7.642247259	6.18E-06
152923_at	FBgn0037044	CG10585	chr3L	-8.290974811	5.38E-07
152924_at	FBgn0038536	CG7655	chr3R	-4.918255432	1.96E-07
152957_at	FBgn0036283	CG32103	chr3L	-4.166104754	2.17E-07
153017_at	FBgn0017429	CG5989	chr3L	-4.304824067	1.15E-07
153049_at	FBgn0037580	CG7415	chr3R	-42.48368026	2.75E-07
153052_at	FBgn0039296	CG10420	chr3R	-10.56639127	0.000176992
153065_at	FBgn0028582	CG8532	chr3L	-3.361241823	7.19E-06
153091_at	FBgn0025455	CG6292	chr3L	-3.759538285	4.17E-05
153107_at	FBgn0037384	CG10981	chr3R	-3.507929871	0.00080537
153152_at	FBgn0039274	CG11920	chr3R	-3.519200799	1.77E-07
153180_at	FBgn0036534	CG6169	chr3L	-3.314620912	3.41E-05
153195_at	FBgn0014930	CG2846	chr3R	-4.670501553	0.000291554
153221_at	FBgn0039643	CG11886	chr3R	-54.78407525	6.66E-16
153228_at	FBgn0039578	CG14066	chr3R	-8.921032973	5.92E-08
153251_at	FBgn0036930	CG7749	chr3L	-6.750653416	1.44E-09
153275_at	FBgn0026210	NA	chr3R	-14.85487227	0.00022523
153282_at	FBgn0035896	CG6983	chr3L	-50.94542298	5.32E-06
153295_at	FBgn0037901	CG6744	chr3R	-27.36690392	5.13E-06
153301_at	FBgn0004493	CG6349	chr3R	-5.857880496	4.11E-09
153343_at	FBgn0039509	CG3350	chr3R	-6.300887118	1.26E-08
153349_at	FBgn0038608	CG7670	chr3R	-17.31819777	0.000174463

153373_at	FBgn0037607	CG8036	chr3R	-3.011392358	6.91E-06
153383_at	FBgn0037277	CG17735	chr3R	-5.92792014	1.24E-09
153407_at	FBgn0001247	CG5517	chr3L	-116.3213138	2.67E-07
153475_at	FBgn0003177	CG31349	chr3R	-5.867300509	2.24E-10
153497_at	FBgn0035105	CG7042	chr3L	-4.452924125	6.41E-05
153513_at	FBgn0035689	CG7376	chr3L	-4.588980783	3.79E-05
153514_at	FBgn0039169	CG5669	chr3R	-7.294603727	1.75E-06
153575_at	FBgn0035148	CG3402	chr3L	-16.17548518	2.84E-08
153576_at	FBgn0036182	CG6084	chr3L	-3.390947312	6.47E-07
153605_at	FBgn0039869	CG1890	chr3R	-10.67151251	1.80E-05
153606_at	FBgn0038760	CG12254	chr3R	-7.604318204	1.47E-07
153633_at	FBgn0023423	CG3412	chr3R	-3.192067357	0.000304071
153643_at	FBgn0038584	CG7175	chr3R	-3.55704069	5.15E-05
153645_at	FBgn0003600	CG6476	chr3R	-7.2697768	1.90E-11
153646_at	FBgn0027620	CG1966	chr3R	-5.882049519	1.03E-08
153674_at	FBgn0017572	CG4083	chr3L	-4.326840445	2.60E-07
153699_at	FBgn0011016	CG5474	chr3L	-4.729726664	3.80E-06
153703_at	FBgn0000575	CG1007	chr3L	-28.61162187	7.29E-14
153704_at	FBgn0035267	CG13921	chr3L	-13.89598084	3.14E-10
153731_at	FBgn0001225	CG4183	chr3L	-6.870225113	4.27E-08
153758_at	FBgn0000338	CG17894	chr3R	-6.318289404	5.36E-06
153779_at	FBgn0000368	CG6383	chr3R	-43.60121676	3.77E-14
153801_at	FBgn0035838	CG7942	chr3L	-3.215322595	5.34E-07
153807_at	FBgn0036090	CG8009	chr3L	-3.199429822	5.68E-07
153850_at	FBgn0038100	CG12358	chr3R	-3.487429562	1.00E-07
153963_at	FBgn0035114	CG33229	chr3L	-18.31558425	2.99E-13
153982_at	FBgn0015019	CG8977	chr3R	-19.16543687	4.77E-10
154005_at	FBgn0027531	CG7600	chr3L	-6.5108884	6.23E-05
154050_at	FBgn0027518	CG7609	chr3R	-4.721869281	0.000231178
154093_at	FBgn0004107	CG10498	chr3R	-3.115070671	1.70E-08
154103_at	FBgn0036299	CG10620	chr3L	-3.084888714	8.39E-08
154107_at	FBgn0038552	CG18012	chr3R	-4.099054792	4.33E-07
154111_at	FBgn0028692	CG11888	chr3R	-6.502489628	1.33E-08
154205_at	FBgn0039141	CG5977	chr3R	-7.654863735	9.74E-07
154212_at	FBgn0039019	CG6990	chr3R	-3.046575703	1.84E-07
154224_at	FBgn0035205	CG2469	chr3L	-3.016819905	7.92E-08
154293_at	FBgn0015660	CG4029	chr3R	-14.76117733	5.52E-10
154327_at	FBgn0020389	CG8363	chr3L	-5.326522917	1.22E-05
154363_at	FBgn0039600	CG1646	chr3R	-3.592069665	4.00E-07
154388_at	FBgn0010340	CG9852	chr3R	-13.21585042	0.000293831
154402_at	FBgn0039589	CG9986	chr3R	-8.368518839	1.92E-09
154480_at	FBgn0027660	CG3897	chr3L	-80.81714291	2.48E-11
154482_at	FBgn0037556	CG9636	chr3R	-3.555287975	0.000816252
154532_at	FBgn0000629	CG6502	chr3L	-12.38065375	5.39E-07
154538_at	FBgn0036513	CG7764	chr3L	-3.073205791	5.39E-05
154549_at	FBgn0038433	CG14896	chr3R	-15.69983681	3.99E-09
154565_at	FBgn0037293	CG12007	chr3R	-40.45020754	5.50E-06
154573_at	FBgn0037700	CG8149	chr3R	-3.247736417	1.83E-06
154604_at	FBgn0038304	CG12241	chr3R	-3.782978206	0.000410021
154631_at	FBgn0038103	CG31342	chr3R	-5.309781458	3.81E-05
154732_at	FBgn0039187	CG6454	chr3R	-8.944985507	2.12E-07
154739_at	FBgn0039144	CG5429	chr3R	-16.14289456	1.59E-08
154773_at	FBgn0013334	CG8884	chr3R	-8.85976931	1.59E-07
154782_at	FBgn0035247	CG13929	chr3L	-4.23650976	3.46E-08
154798_at	FBgn0039499	CG18766	chr3R	-24.95659333	7.17E-06
154809_at	FBgn0039636	CG11877	chr3R	-3.303114789	3.55E-05
154843_at	FBgn0035850	CG7986	chr3L	-3.686306983	4.18E-09

154866_at	FBgn0000140	CG6875	chr3R	-40.6535089	8.04E-14
154894_at	FBgn0016792	CG8374	chr3R	-13.4984849	8.08E-09
154900_at	FBgn0036516	CG7656	chr3L	-5.716972917	7.40E-07
154920_at	FBgn0035388	CG2162	chr3L	-28.40342531	2.63E-10
154936_at	FBgn0038184	CG9285	chr3R	-4.193070081	4.81E-08
154946_at	FBgn0036761	CG5546	chr3L	-11.87291117	1.30E-11
154956_at	FBgn0039073	CG4408	chr3R	-67.96957935	1.52E-10
154969_at	FBgn0037644	CG11964	chr3R	-12.21082604	8.90E-08
154996_at	FBgn0005674	CG5394	chr3R	-7.135257211	6.36E-06
155012_at	FBgn0038538	CG7660	chr3R	-11.78415037	3.99E-11
155014_at	FBgn0036988	CG5262	chr3L	-12.73708659	0.000329229
155022_at	FBgn0011771	CG5837	chr3L	-22.69445846	3.15E-05
155041_at	FBgn0013343	CG31136	chr3R	-3.898882227	1.07E-08
155043_at	FBgn0001179	CG8019	chr3L	-4.826217375	1.14E-08
155067_at	FBgn0036037	CG6754	chr3L	-37.29676081	0.00014658
155107_at	FBgn0022349	CG1910	chr3R	-3.659083699	1.18E-07
155131_at	FBgn0037719	CG9424	chr3R	-6.781984678	1.98E-09
155136_at	FBgn0037371	CG2097	chr3R	-11.83976655	2.74E-11
141427_at	FBgn0031098	CG17068	chrX	-7.703736424	1.86E-07
141459_at	FBgn0029972	CG1422	chrX	-12.20038576	4.13E-07
141464_at	FBgn0003048	CG3443	chrX	-17.23106155	0.000397106
141492_at	FBgn0030242	CG1655	chrX	-7.995791836	1.33E-05
141493_at	FBgn0000719	CG9559	chrX	-14.22507534	1.14E-06
141585_at	FBgn0031187	CG14619	chrX	-7.385410375	1.73E-08
141657_at	FBgn0030211	CG33175	chrX	-195.496528	1.43E-08
141681_at	FBgn0030235	CG1691	chrX	-6.762213328	9.05E-08
141692_at	FBgn0030650	CG33206	chrX	-5.039857506	8.55E-05
141713_at	FBgn0004643	CG9900	chrX	-144.0344433	8.33E-08
141722_at	FBgn0004050	CG7803	chrX	-8.864567314	3.59E-07
141734_at	FBgn0030808	CG4937	chrX	-4.511635169	1.41E-05
141747_at	FBgn0030606	CG9053	chrX	-3.320673006	7.27E-05
141751_at	FBgn0023544	CG4399	chrX	-3.033206945	5.16E-05
141759_at	FBgn0020383	CG9209	chrX	-7.853184509	9.76E-07
141774_at	FBgn0031020	CG12202	chrX	-29.04859482	2.80E-05
142170_at	FBgn0011837	CG4070	chrX	-4.648347134	8.94E-07
142203_at	FBgn0027792	CG14788	chrX	-4.173205626	6.11E-06
142338_at	FBgn0004368	CG6899	chrX	-75.96554283	5.36E-07
142464_at	FBgn0031059	CG14229	chrX	-3.553256497	4.19E-05
142541_at	FBgn0029613	CG14048	chrX	-5.353299407	2.32E-08
142777_at	FBgn0004655	CG3707	chrX	-10.39996027	4.74E-05
142823_at	FBgn0020547	CG3000	chrX	-27.39046451	1.78E-05
142824_at	FBgn0022768	CG2984	chrX	-3.349488096	0.000580775
142871_s_at	FBgn0030734	CG9911	chrX	-5.196175346	0.000203574
142977_at	FBgn0030086	CG7033	chrX	-4.210949617	9.98E-06
143083_at	FBgn0000163	CG5055	chrX	-5.044842131	1.79E-05
143092_at	FBgn0000259	CG15224	chrX	-9.810282585	7.23E-07
143133_at	FBgn0000499	CG18361	chrX	-7.04322649	5.99E-09
143135_at	FBgn0000524	CG3929	chrX	-14.96306892	2.68E-05
143161_at	FBgn0000709	CG1484	chrX	-13.66737255	1.11E-06
143175_at	FBgn0001092	CG8893	chrX	-5.885673966	8.18E-07
143302_at	FBgn0003079	CG2845	chrX	-5.805549591	1.73E-05
143373_at	FBgn0003659	CG33070	chrX	-11.1950905	0.00013737
143735_at	FBgn0013987	CG3086	chrX	-4.58358372	4.49E-07
143963_at	FBgn0022724	CG7128	chrX	-10.71351053	7.51E-05
143994_at	FBgn0023518	CG3848	chrX	-3.12646093	0.000308342
144094_at	FBgn0025800	CG2262	chrX	-67.07769398	1.18E-06
144114_at	FBgn0026076	CG9038	chrX	-20.03665112	1.79E-12

144119_at	FBgn0026089	CG14817	chrX	-4.474999764	5.20E-07
144174_at	FBgn0027108	NA	chrX	-3.831287721	7.65E-07
144179_at	FBgn0027359	CG1728	chrX	-3.711829201	8.05E-05
144245_at	FBgn0028686	CG16916	chrX	-3.011540103	1.66E-05
144358_at	FBgn0029541	CG3034	chrX	-3.240945414	4.81E-07
144430_at	FBgn0029650	CG3939	chrX	-3.374287903	1.47E-05
144482_at	FBgn0029714	CG3527	chrX	-3.218030992	1.85E-05
144485_at	FBgn0029718	CG7038	chrX	-5.61004717	2.92E-07
144536_at	FBgn0029786	CG3171	chrX	-9.818029895	4.40E-05
144548_at	FBgn0029800	CG15929	chrX	-11.44674504	1.13E-09
144585_at	FBgn0029853	CG3781	chrX	-15.09777648	7.93E-07
144595_at	FBgn0029868	CG3446	chrX	-7.268428223	1.94E-10
144604_at	FBgn0029884	CG32742	chrX	-11.26125403	0.000990755
144653_at	FBgn0029959	CG12156	chrX	-5.685153689	3.68E-08
144709_at	FBgn0030056	CG11284	chrX	-4.136419489	0.000133872
144730_at	FBgn0030090	CG12664	chrX	-4.516312778	0.00014123
144790_at	FBgn0030189	CG2909	chrX	-6.986920024	1.15E-06
144803_at	FBgn0030205	CG17255	chrX	-6.654556921	4.17E-05
144831_at	FBgn0030239	CG17333	chrX	-53.150914	4.11E-06
144867_at	FBgn0030291	CG1738	chrX	-248.8088508	7.48E-09
144883_at	FBgn0030311	CG11699	chrX	-3.766611386	0.000362995
144884_at	FBgn0030312	CG1558	chrX	-5.671837263	6.64E-08
144889_at	FBgn0030320	CG2247	chrX	-3.018366497	6.38E-05
144972_at	FBgn0030434	CG4400	chrX	-7.800869838	3.03E-08
145032_at	FBgn0030522	CG11103	chrX	-4.621530034	4.74E-07
145056_at	FBgn0030552	CG15871	chrX	-5.984757929	0.000856879
145071_at	FBgn0030572	CG14413	chrX	-9.246099695	0.000191093
145095_at	FBgn0030602	CG32594	chrX	-29.49693294	1.36E-05
145118_at	FBgn0030637	CG9195	chrX	-3.590674784	7.36E-05
145131_at	FBgn0030659	CG9215	chrX	-3.05796915	0.00066398
145132_at	FBgn0030660	CG8097	chrX	-12.84398268	1.55E-09
145155_at	FBgn0030694	CG15602	chrX	-5.634448072	1.84E-05
145163_at	FBgn0030711	CG8928	chrX	-4.142614096	2.77E-07
145251_at	FBgn0030855	CG5800	chrX	-4.159602684	6.45E-06
145261_at	FBgn0030867	CG32560	chrX	-83.13823592	5.11E-08
145276_at	FBgn0030894	CG7192	chrX	-3.353146741	4.22E-05
145379_at	FBgn0031036	CG14220	chrX	-18.46751952	3.27E-05
145395_at	FBgn0031069	CG12703	chrX	-4.66864325	9.51E-07
151363_at	FBgn0040890	CG14199	chrX	-4.493443224	2.10E-05
151370_at	FBgn0040899	CG17776	chrX	-3.645144678	2.62E-06
151389_at	FBgn0040920	CG32736	chrX	-6.019656659	0.000693215
151390_at	FBgn0040921	CG32736	chrX	-10.18516595	9.80E-10
151397_at	FBgn0040928	CG15345	chrX	-16.04096937	5.89E-06
151504_i_at	GH10316.complete-hit	CG3620	chrX	-4.372962594	5.68E-05
151557_i_at	GH23604.3prime-hit	CG32737	chrX	-28.65856574	5.18E-07
151603_at	HL05860.3prime-hit	CG7053	chrX	-43.42567078	1.46E-10
151606_at	HL07962.3prime-hit	CG1841	chrX	-32.06380721	2.50E-05
151610_at	LD03548.3prime-hit	CG3026	chrX	-3.045337843	0.000124416
151660_at	LD21771.3prime-hit	CG3429	chrX	-5.248301022	0.000130592
151703_at	LD38919.3prime-hit	CG14224	chrX	-3.630274401	1.89E-06
151740_at	SD02216.3prime-hit	CG7033	chrX	-6.915792979	2.87E-09
151790_at	FBgn0031174	CG1486	chrX	-9.829463177	5.20E-08
151869_at	FBgn0030328	CG2446	chrX	-3.128449799	1.82E-05
151908_at	FBgn0030668	CG8128	chrX	-4.346394338	0.000112962
151972_at	FBgn0030679	CG8206	chrX	-5.737660422	3.71E-07
152009_at	FBgn0030692	CG8470	chrX	-3.441983207	5.90E-06
152014_at	FBgn0025633	CG13366	chrX	-30.85949707	2.19E-08

152024_at	FBgn0029818	CG3033	chrX	-14.14105245	0.000263137
152069_at	FBgn0005410	CG1903	chrX	-84.13362361	7.96E-07
152239_at	FBgn0028486	CG2967	chrX	-94.25815136	3.49E-07
152259_at	FBgn0030671	CG8134	chrX	-25.03535207	3.72E-05
152295_at	FBgn0027553	CG32721	chrX	-3.164694764	1.05E-05
152337_at	FBgn0029878	CG10695	chrX	-8.677474705	1.17E-06
152404_at	FBgn0030327	CG2448	chrX	-25.59796799	1.02E-11
152433_at	FBgn0026144	CG1435	chrX	-12.51624159	0.000582523
152453_at	FBgn0023169	CG3051	chrX	-3.942546303	9.05E-06
152691_at	FBgn0030061	CG1785	chrX	-4.002889853	1.75E-06
152692_at	FBgn0027621	CG3400	chrX	-29.27015423	6.86E-13
152858_at	FBgn0003302	CG4336	chrX	-3.585346729	7.98E-05
152953_at	FBgn0030411	CG2540	chrX	-4.297267269	1.40E-05
152977_at	FBgn0029666	CG10803	chrX	-31.74607122	6.98E-06
153035_at	FBgn0030028	CG10965	chrX	-14.03317819	6.13E-09
153044_at	FBgn0015565	CG2913	chrX	-9.588917164	1.11E-08
153060_at	FBgn0023526	CG2865	chrX	-125.1186497	4.06E-08
153081_at	FBgn0011754	CG1830	chrX	-4.938470409	4.30E-05
153153_at	FBgn0015926	CG6157	chrX	-3.191185533	3.12E-05
153167_at	FBgn0030241	CG11207	chrX	-4.290438436	1.88E-07
153190_at	FBgn0030753	CG4420	chrX	-4.146022859	5.43E-06
153204_at	FBgn0030406	CG1463	chrX	-3.983016975	2.88E-06
153222_at	FBgn0030274	CG1908	chrX	-65.10946786	9.62E-07
153230_at	FBgn0024251	CG32527	chrX	-46.48266827	1.02E-05
153242_at	FBgn0023515	CG14814	chrX	-6.128983033	1.25E-07
153253_at	FBgn0031003	CG7889	chrX	-9.30963091	0.000680097
153332_at	FBgn0029958	CG12151	chrX	-28.54075047	2.14E-05
153463_at	FBgn0030243	CG2186	chrX	-5.665643058	1.29E-06
153517_at	FBgn0029970	CG17256	chrX	-4.462138392	8.63E-06
153571_at	FBgn0026751	CG4208	chrX	-3.591412804	0.000940656
153592_at	FBgn0024332	CG4206	chrX	-5.505411065	9.49E-06
153617_at	FBgn0030699	CG8578	chrX	-14.10264933	6.71E-05
153647_at	FBgn0014133	CG1822	chrX	-3.677547286	4.41E-05
153675_at	FBgn0003964	CG4380	chrX	-90.89055912	6.18E-07
153695_at	FBgn0030878	CG6769	chrX	-6.105533917	3.43E-05
153702_at	FBgn0031046	CG12238	chrX	-16.46187595	7.38E-08
153708_at	FBgn0029516	CG2995	chrX	-18.91273558	0.00012197
153751_at	FBgn0030003	CG2116	chrX	-5.241721318	3.53E-06
153783_at	FBgn0029664	CG10802	chrX	-5.516326608	1.29E-05
153784_at	FBgn0023130	CG3771	chrX	-3.980155621	0.000331434
153789_at	FBgn0029882	CG3226	chrX	-4.09169511	1.40E-06
153790_at	FBgn0029721	CG7010	chrX	-6.113921441	1.28E-05
153910_at	FBgn0030858	CG8211	chrX	-4.287969878	1.62E-06
153917_at	FBgn0023536	CG3156	chrX	-9.435930641	0.000941157
153942_at	FBgn0030710	CG8924	chrX	-4.984426518	2.47E-06
154013_at	FBgn0031148	CG1753	chrX	-3.681911962	7.54E-07
154039_at	FBgn0031105	CG11738	chrX	-3.105319257	5.06E-06
154041_at	FBgn0027521	CG3679	chrX	-8.559604934	6.30E-08
154060_at	FBgn0011606	CG8590	chrX	-4.150239193	1.77E-05
154096_at	FBgn0029737	CG6903	chrX	-3.479953904	7.15E-06
154099_at	FBgn0000257	CG12230	chrX	-26.10930277	3.23E-05
154130_at	FBgn0030115	CG15797	chrX	-4.115124838	5.64E-06
154137_at	FBgn0010315	CG9096	chrX	-3.590181537	3.54E-05
154188_at	FBgn0030817	CG4991	chrX	-10.43201701	1.32E-05
154221_at	FBgn0011761	CG4193	chrX	-31.18374974	3.90E-11
154236_at	FBgn0030063	CG1789	chrX	-5.713622583	5.78E-08
154246_at	FBgn0030891	CG7098	chrX	-4.145644635	2.70E-05

154261_at	FBgn0030973	CG7332	chrX	-5.37043855	2.30E-06
154279_at	FBgn0029680	CG2934	chrX	-3.055845605	1.70E-05
154323_at	FBgn0030944	CG6617	chrX	-3.430951258	2.30E-05
154337_at	FBgn0030967	CG7282	chrX	-3.86594016	1.71E-06
154351_at	FBgn0020369	CG1489	chrX	-3.901845312	1.21E-07
154417_at	FBgn0030146	CG32697	chrX	-3.507067225	0.000196746
154424_at	FBgn0028469	CG8062	chrX	-3.503806404	0.000509276
154460_at	FBgn0030557	CG12047	chrX	-5.663518125	1.20E-07
154462_at	FBgn0030849	CG5675	chrX	-17.99951021	0.000113735
154489_at	FBgn0000376	CG2714	chrX	-4.085252886	0.000125077
154490_at	FBgn0031118	CG1412	chrX	-5.19958004	8.70E-06
154558_at	FBgn0026080	CG6121	chrX	-6.766100342	6.52E-08
154638_at	FBgn0031062	CG14230	chrX	-4.052445284	2.95E-05
154705_at	FBgn0031061	CG14232	chrX	-21.36157873	1.39E-06
154709_at	FBgn0030321	CG1703	chrX	-4.37184666	4.07E-06
154712_at	FBgn0030871	CG8142	chrX	-6.004541924	1.04E-07
154744_at	FBgn0026143	CG3658	chrX	-21.52929289	6.24E-05
154753_at	FBgn0030870	CG6398	chrX	-3.979111152	9.09E-06
154770_at	FBgn0030854	CG8289	chrX	-6.528325547	5.70E-07
154841_at	FBgn0030435	CG4645	chrX	-4.585907038	3.10E-07
154933_at	FBgn0026749	CG1989	chrX	-4.388404191	6.88E-06
154939_at	FBgn0010269	CG15793	chrX	-8.423822007	6.28E-09
154944_at	FBgn0030856	CG32555	chrX	-5.850908528	5.96E-08
154982_at	FBgn0031120	CG18492	chrX	-4.062853084	3.04E-06
155024_at	FBgn0031091	CG9576	chrX	-7.634945776	8.41E-05
155026_at	FBgn0030612	CG5599	chrX	-6.892586239	7.57E-06
155028_at	FBgn0023527	CG3071	chrX	-5.657478894	8.57E-07
155109_at	FBgn0025617	CG7359	chrX	-3.142043126	0.000344836
155132_at	FBgn0000520	CG2711	chrX	-15.1576915	4.01E-05
