# Supplementary material for: Unmasking Activation of the Zygotic Genome Using Chromosomal Deletions in the Drosophila Embryo
Source: PLoS Biol. 2007 Apr 24;5(5):e117. doi: 10.1371/journal.pbio.0050117 (PMC1854917; doi:10.1371/journal.pbio.0050117)
Supplement: Supplementary file 7 [file pbio.0050117.st007.pdf]

Table S7: Maternal Degraded and Zygotic

affy_probe_set_id	fbgn for the probe set	CG id	Chr.Location	fold-changes	p-value
141270_at	FBgn0031291	CG33526	chr2L	-12.1706207	1.46E-06
141296_at	FBgn0031950	CG14536	chr2L	-5.80261017	6.12E-06
141350_at	FBgn0031657	CG3756	chr2L	-4.92609028	1.02E-06
141359_at	FBgn0031590	CG3702	chr2L	-3.3755244	3.29E-10
141496_at	FBgn0032466	CG5092	chr2L	-3.99488158	2.77E-07
141548_at	FBgn0031290	CG4276	chr2L	-28.7964814	1.72E-08
141598_at	FBgn0032731	CG10641	chr2L	-5.80159605	3.55E-05
141633_at	FBgn0032036	CG13384	chr2L	-25.4058134	1.12E-10
141679_at	FBgn0031605	CG15438	chr2L	-3.41647239	3.65E-08
141782_at	FBgn0028633	CG2615	chr2L	-3.22217323	2.32E-07
142449_at	FBgn0032205	CG4957	chr2L	-3.32748174	0.00074045
142821_at	FBgn0015772	CG10637	chr2L	-6.13774626	2.88E-08
142875_at	FBgn0032646	CG6412	chr2L	-3.09116693	0.00011598
142899_at	FBgn0032197	CG5694	chr2L	-3.32678522	9.30E-12
142975_at	FBgn0011673	CG7438	chr2L	-3.15989091	1.43E-06
143117_at	FBgn0000413	CG5102	chr2L	-7.71390862	0
143330_at	FBgn0003310	CG4385	chr2L	-4.7288412	4.66E-15
143377_at	FBgn0003716	CG14026	chr2L	-3.32399156	1.13E-11
143573_at	FBgn0005672	CG10334	chr2L	-70.0646194	1.25E-14
143636_at	FBgn0011202	CG1768	chr2L	-16.7909104	2.80E-10
143663_at	FBgn0011584	CG4758	chr2L	-12.075195	1.80E-06
143668_at	FBgn0011638	CG10922	chr2L	-8.53141709	2.33E-05
143743_at	FBgn0014015	CG33529	chr2L	-19.8720401	1.24E-09
143774_at	FBgn0015000	CG3469	chr2L	-9.86387172	1.53E-05
143794_at	FBgn0015299	CG8396	chr2L	-4.0396451	3.20E-07
143820_at	FBgn0015567	CG4260	chr2L	-5.11749574	3.12E-05
143833_at	FBgn0015621	CG3642	chr2L	-3.95749928	1.22E-12
143858_at	FBgn0015924	CG4280	chr2L	-6.65324679	0.00018244
144125_at	FBgn0026379	CG5671	chr2L	-8.07735922	4.29E-06
144234_at	FBgn0028538	CG7578	chr2L	-38.2410798	2.92E-11
144247_at	FBgn0028694	CG18174	chr2L	-9.41579928	5.51E-08
145523_at	FBgn0031243	CG17078	chr2L	-71.9407751	3.91E-12
145680_at	FBgn0031475	CG31694	chr2L	-5.57139064	1.30E-10
145713_at	FBgn0031535	CG12795	chr2L	-3.53613419	2.97E-09
145947_at	FBgn0031898	CG4567	chr2L	-3.46542531	2.87E-06
146130_at	FBgn0032194	CG4901	chr2L	-23.3114745	3.67E-08
146131_at	FBgn0032195	CG4908	chr2L	-7.54382537	2.56E-06
146139_at	FBgn0032217	CG4972	chr2L	-3.5542715	2.01E-07
146156_at	FBgn0032244	CG5313	chr2L	-9.74938247	2.95E-10
146237_at	FBgn0032355	CG6392	chr2L	-14.9718741	3.44E-07
146263_at	FBgn0032390	CG16969	chr2L	-21.2571563	2.45E-08
146307_at	FBgn0032468	CG9933	chr2L	-16.0008179	2.82E-08
146597_at	FBgn0032960	CG11628	chr2L	-7.33554796	2.61E-12
151005_at	FBgn0040518	CG11628	chr2L	-5.88763837	0.00013749
151611_at	LD03769.3prime-hit	CG5300	chr2L	-3.90922164	5.73E-13
151682_at	LD29596.3prime-hit	CG1759	chr2L	-10.6509213	2.22E-16
151752_s_at	SD07726.3prime-hit	CG33123	chr2L	-10.5000773	1.28E-05
151787_at	FBgn0032157	CG5899	chr2L	-6.713499	1.09E-06
151854_at	FBgn0027609	CG15437	chr2L	-4.63969723	7.52E-09

151878_at	FBgn0028541	CG7364	chr2L	-6.04435222	9.55E-11
151989_at	FBgn0031645	CG3036	chr2L	-67.3241386	0
152360_at	FBgn0000675	CG5686	chr2L	-5.62519481	5.61E-09
152413_at	FBgn0022153	CG3054	chr2L	-6.6206697	1.36E-05
152479_at	FBgn0032395	CG6734	chr2L	-5.31484239	5.49E-05
152487_at	FBgn0024371	CG1071	chr2L	-15.0321934	0
152508_at	FBgn0031897	CG13784	chr2L	-14.7584757	0
152525_at	FBgn0010460	CG5461	chr2L	-15.5074263	0
152549_at	FBgn0017551	CG10800	chr2L	-10.6730381	5.55E-09
152727_at	FBgn0031971	CG7224	chr2L	-4.20483909	2.00E-05
152854_at	FBgn0028507	CG3793	chr2L	-4.17866286	4.10E-05
153172_at	FBgn0032079	CG31886	chr2L	-9.78617054	0.00029717
153232_at	FBgn0032121	CG33298	chr2L	-10.38134	5.80E-14
153281_at	FBgn0031661	CG8890	chr2L	-3.14336209	2.30E-10
153299_at	FBgn0032451	CG12292	chr2L	-28.0218796	5.88E-09
153597_at	FBgn0024291	CG5216	chr2L	-7.53693152	4.60E-14
153624_at	FBgn0032694	CG15162	chr2L	-71.5316406	2.33E-07
153634_at	FBgn0031911	CG5229	chr2L	-4.33535753	1.44E-09
153746_at	FBgn0005683	CG5354	chr2L	-7.10337678	1.35E-13
153768_at	FBgn0032873	CG2614	chr2L	-4.49819195	0.000117
153866_at	FBgn0031303	CG4710	chr2L	-70.6773719	2.48E-11
153872_at	FBgn0010300	CG10719	chr2L	-4.516031	1.33E-15
153912_at	FBgn0032691	CG10414	chr2L	-60.244949	9.50E-11
153943_at	FBgn0031250	CG11907	chr2L	-11.5552831	4.43E-06
153957_at	FBgn0031752	CG9044	chr2L	-12.8160388	2.10E-07
154102_at	FBgn0032118	CG3766	chr2L	-9.4178587	6.17E-12
154115_at	FBgn0032393	CG12264	chr2L	-4.8105123	9.81E-11
154120_at	FBgn0010382	CG3938	chr2L	-31.6642696	0
154152_at	FBgn0020240	CG7586	chr2L	-7.06481322	0.00015839
154192_at	FBgn0023388	CG1099	chr2L	-6.86380983	1.59E-06
154346_at	FBgn0002973	CG3779	chr2L	-27.2780656	0
154362_at	FBgn0031633	CG2976	chr2L	-3.20607148	4.57E-10
154386_at	FBgn0015600	CG9660	chr2L	-59.1935715	4.42E-14
154411_at	FBgn0019686	CG10895	chr2L	-6.29103276	3.19E-12
154615_at	FBgn0031875	CG3430	chr2L	-11.0137416	1.36E-05
154673_at	FBgn0020616	CG3423	chr2L	-25.7573252	2.32E-10
154677_at	FBgn0011207	CG3959	chr2L	-7.46756039	5.30E-10
154877_at	FBgn0031769	CG9135	chr2L	-3.24088525	1.29E-08
155059_at	FBgn0032880	CG9318	chr2L	-4.55740098	6.57E-05
155080_at	FBgn0032348	CG4751	chr2L	-13.0691678	1.11E-16
155094_at	FBgn0010229	CG8676	chr2L	-15.1885988	1.33E-08
141249_at	FBgn0033740	CG8828	chr2R	-4.03292552	4.05E-05
141261_at	FBgn0033454	CG1671	chr2R	-4.42988836	8.16E-08
141451_at	FBgn0035026	CG12252	chr2R	-3.60031452	4.37E-05
141454_at	FBgn0033609	CG13213	chr2R	-48.6347651	2.95E-10
141467_at	FBgn0003175	CG4444	chr2R	-8.43987963	1.18E-12
141514_at	FBgn0025185	CG1605	chr2R	-3.47211729	5.75E-08
141591_at	FBgn0023172	CG9635	chr2R	-17.7589146	4.72E-08
141635_at	FBgn0034964	CG3173	chr2R	-6.42135477	4.75E-13
141716_at	FBgn0022764	CG8815	chr2R	-6.8647651	3.35E-11
141767_at	FBgn0003044	CG5109	chr2R	-7.50734808	5.11E-13

141783_at	FBgn0033796	CG17054	chr2R	-21.2616534	3.90E-09
141796_at	FBgn0034271	CG4996	chr2R	-6.46007552	0.00019618
141803_at	FBgn0034280	CG11430	chr2R	-23.203961	1.17E-08
141809_at	FBgn0034447	CG7744	chr2R	-6.73818455	4.11E-08
142143_at	FBgn0016078	CG8804	chr2R	-6.86192763	1.39E-05
142245_at	FBgn0025790	CG10327	chr2R	-16.0002517	1.78E-15
142263_at	FBgn0035075	CG32836	chr2R	-4.77707369	1.06E-06
142296_at	FBgn0028552	CG3988	chr2R	-21.0706243	2.30E-08
142399_at	FBgn0033631	CG9027	chr2R	-3.52697933	5.18E-11
142479_at	FBgn0034342	CG17533	chr2R	-6.1754292	1.56E-05
142509_at	FBgn0033846	CG6061	chr2R	-17.9249003	4.76E-09
142585_at	FBgn0033804	CG17724	chr2R	-8.45654863	8.52E-05
142621_at	FBgn0033974	CG33506	chr2R	-6.76377279	4.06E-05
142653_at	FBgn0034058	CG8315	chr2R	-3.12820738	6.97E-06
142659_at	FBgn0027519	CG8325	chr2R	-7.15636267	1.35E-06
142872_at	FBgn0011704	CG8975	chr2R	-17.0203872	0
142919_s_at	FBgn0033398	CG2049	chr2R	-5.72550879	4.94E-10
143000_at	FBgn0032444	CG5525	chr2L	-4.38290901	3.64E-11
143080_at	FBgn0000142	CG8787	chr2R	-5.88532481	1.74E-06
143558_at	FBgn0005624	CG3886	chr2R	-56.6208415	0
143631_at	FBgn0010638	CG10130	chr2R	-9.78635983	4.44E-16
143721_at	FBgn0013765	CG4832	chr2R	-9.2258479	3.33E-16
143907_at	FBgn0019968	CG8183	chr2R	-34.1182073	5.46E-09
145520_at	FBgn0031238	CG3645	chr2L	-3.02230289	0.00025563
145561_at	FBgn0031309	CG5041	chr2L	-4.22699266	4.27E-09
145681_at	FBgn0031477	CG31694	chr2L	-3.47574739	4.64E-08
146045_at	FBgn0032060	CG9298	chr2L	-15.5504148	6.14E-09
146264_s_at	FBgn0032392	CG12264/CG3	chr2L	-4.28365436	2.57E-07
146745_at	FBgn0033183	CG1620	chr2R	-7.32524119	2.93E-07
146788_at	FBgn0033259	CG11210	chr2R	-3.08579363	2.05E-06
146833_at	FBgn0033329	CG14767	chr2R	-4.79091206	7.27E-09
146913_at	FBgn0033468	CG1418	chr2R	-9.96334589	1.77E-06
146935_at	FBgn0033506	CG3298	chr2R	-19.4942276	3.35E-07
146951_at	FBgn0033526	CG12892	chr2R	-5.86745614	1.08E-11
146953_at	FBgn0033528	CG11761	chr2R	-6.3450101	1.29E-08
147023_at	FBgn0033638	CG9005	chr2R	-4.33904524	2.82E-05
147079_at	FBgn0033714	CG8487	chr2R	-13.9670108	8.66E-07
147081_at	FBgn0033718	CG13162	chr2R	-32.189997	7.69E-09
147123_at	FBgn0033784	CG13322	chr2R	-6.85661486	2.32E-05
147130_at	FBgn0033795	CG17054	chr2R	-24.0963649	1.46E-08
147246_at	FBgn0034016	CG8171	chr2R	-4.36335271	1.58E-10
147278_at	FBgn0034073	CG8414	chr2R	-33.9620136	2.22E-09
147342_at	FBgn0034170	CG33456	chr2R	-14.3433252	3.75E-09
147361_at	FBgn0034210	CG6568	chr2R	-6.93062079	6.10E-05
147376_at	FBgn0034240	CG4903	chr2R	-3.2380421	3.92E-09
147380_at	FBgn0034249	CG6477	chr2R	-13.2736969	1.47E-07
147434_at	FBgn0034334	CG17522	chr2R	-64.0689122	1.03E-11
147558_at	FBgn0034527	CG9945	chr2R	-4.81171647	2.35E-08
147592_at	FBgn0034570	CG10543	chr2R	-3.51519148	7.92E-08
147674_at	FBgn0034726	CG11301	chr2R	-30.4369063	4.06E-09
147766_at	FBgn0034849	CG3500	chr2R	-4.23620417	1.60E-07

147831_at	FBgn0034963	CG13567	chr2R	-3.41806279	0.00011577
151545_at	GH20047.3prime-hit	CG8205	chr2R	-7.78439187	1.58E-13
151573_at	GM02568.3prime-hit	CG13344	chr2R	-5.8546692	6.30E-07
151647_at	LD17611.3prime-hit	CG33505	chr2R	-13.6647976	9.38E-07
151910_at	FBgn0019886	CG4589	chr2R	-19.4964491	6.94E-10
152018_at	FBgn0033522	CG11763	chr2R	-3.10038912	1.62E-07
152198_at	FBgn0027561	CG18659	chr2R	-4.55871216	8.84E-05
152264_at	FBgn0033988	CG7761	chr2R	-23.1544272	2.91E-14
152427_at	FBgn0003353	CG3182	chr2R	-5.84945833	1.36E-06
152430_at	FBgn0033780	CG3845	chr2R	-3.37957624	2.79E-07
152444_at	FBgn0011236	CG5575	chr2R	-15.6939066	2.42E-08
152450_at	FBgn0031487	CG3558	chr2L	-6.53306729	1.75E-07
152461_at	FBgn0033470	CG12131	chr2R	-7.40999538	3.45E-08
152515_at	FBgn0003074	CG8251	chr2R	-4.72142299	2.19E-05
152598_at	FBgn0034335	CG5164	chr2R	-48.5017453	0
152663_at	FBgn0033272	CG8707	chr2R	-6.62397089	6.50E-11
152719_at	FBgn0025830	CG5859	chr2R	-16.8874175	2.37E-07
152792_at	FBgn0031987	CG12375	chr2L	-18.9853453	2.03E-07
152868_at	FBgn0033636	CG10897	chr2R	-6.85580993	4.61E-13
152896_at	FBgn0003396	CG7734	chr2R	-34.9748225	9.65E-12
152964_at	FBgn0033915	CG8485	chr2R	-3.66615282	3.28E-09
152991_at	FBgn0034349	CG5186	chr2R	-51.1379384	2.00E-10
153061_at	FBgn0035055	CG30421	chr2R	-16.8155347	2.55E-07
153112_at	FBgn0034946	CG3065	chr2R	-7.03460426	7.74E-07
153163_at	FBgn0014877	CG6155	chr2R	-7.16181652	1.88E-11
153179_at	FBgn0033486	CG11866	chr2R	-19.1073844	2.32E-08
153245_at	FBgn0034982	CG4065	chr2R	-3.61165493	0.00033299
153246_at	FBgn0033209	CG12107	chr2R	-5.33059258	2.94E-05
153279_at	FBgn0027912	CG9035	chr2R	-9.17838231	4.09E-09
153329_at	FBgn0033637	CG9006	chr2R	-40.4489224	2.80E-10
153387_at	FBgn0033583	CG7736	chr2R	-4.56922111	9.59E-07
153451_at	FBgn0013762	CG8203	chr2R	-6.44543891	6.12E-05
153493_at	FBgn0034068	CG8400	chr2R	-3.18274163	1.69E-08
153579_at	FBgn0011703	CG5371	chr2L	-5.30314511	3.87E-12
153659_at	FBgn0000054	CG15845	chr2R	-30.1439613	2.72E-11
153737_at	FBgn0022772	CG10667	chr2R	-4.0611753	4.14E-09
153809_at	FBgn0014269	CG15107	chr2R	-119.548323	3.17E-12
153961_at	FBgn0010314	CG3738	chr2L	-3.50395051	4.33E-09
153986_at	FBgn0034083	CG8434	chr2R	-12.0823681	4.23E-07
153993_at	FBgn0033094	CG3403	chr2R	-5.55963349	3.43E-12
154004_at	FBgn0034970	CG4005	chr2R	-5.08042335	1.13E-08
154147_at	FBgn0034585	CG4030	chr2R	-4.90542082	1.57E-08
154213_at	FBgn0033992	CG11798	chr2R	-12.8032838	1.36E-13
154234_at	FBgn0023081	CG4012	chr2R	-4.48719421	2.17E-11
154254_at	FBgn0034650	CG10318	chr2R	-18.4377003	2.44E-08
154434_at	FBgn0032481	CG16972	chr2L	-5.78564264	0.00063271
154495_at	FBgn0034259	CG6459	chr2R	-7.88801709	8.77E-07
154622_at	FBgn0033616	CG7745	chr2R	-6.77690424	3.32E-05
154643_at	FBgn0031884	CG13777	chr2L	-3.16102058	2.48E-07
154771_at	FBgn0033413	CG8806	chr2R	-4.29228534	6.78E-08
154892_at	FBgn0034488	CG11208	chr2R	-18.6773662	7.36E-10

154954_at	FBgn0015295	CG18247	chr2R	-4.08201687	5.21E-05
154966_at	FBgn0034036	CG33139	chr2R	-5.68214517	1.09E-11
154998_at	FBgn0010356	CG7704	chr2R	-3.06046953	3.21E-08
155001_at	FBgn0033677	CG8321	chr2R	-5.59376295	0.00018814
155031_at	FBgn0033346	CG11770	chr2R	-4.02200328	2.08E-06
155070_at	FBgn0033088	CG3271	chr2R	-4.02857207	1.79E-09
155073_at	FBgn0028563	CG8714	chr2R	-8.00565528	9.72E-06
155120_at	FBgn0034643	CG10321	chr2R	-4.76280037	0.0001213
155155_at	FBgn0033569	CG12942	chr2R	-5.8058416	8.31E-07
141206_at	FBgn0025725	CG7961	chr3L	-5.56380672	4.80E-08
141231_at	FBgn0028688	CG5378	chr3R	-6.36025478	6.55E-10
141254_at	FBgn0037360	CG2182	chr3R	-6.01796248	6.89E-09
141265_at	FBgn0036033	CG6757	chr3L	-5.81684775	1.18E-05
141302_at	FBgn0037368	CG1239	chr3R	-44.6433359	1.26E-06
141370_at	FBgn0027945	CG7758	chr3L	-25.291461	2.98E-06
141376_at	FBgn0037795	CG6264	chr3R	-26.2159526	2.68E-05
141413_at	FBgn0038364	CG4699	chr3R	-8.05831947	4.07E-09
141425_at	FBgn0038810	CG5434	chr3R	-14.9100702	1.21E-08
141452_at	FBgn0035137	CG1233	chr3L	-9.6923607	4.00E-07
141477_at	FBgn0038318	CG6236	chr3R	-3.56706244	5.51E-05
141490_at	FBgn0039146	CG6057	chr3R	-4.06979994	3.00E-05
141508_at	FBgn0037120	CG11247	chr3L	-79.003008	2.16E-06
141509_at	FBgn0035145	CG12031	chr3L	-14.8981853	7.23E-07
141511_at	FBgn0016794	CG1044	chr3L	-16.3320104	5.18E-07
141524_at	FBgn0035688	CG10289	chr3L	-33.0340705	3.84E-11
141546_at	FBgn0037250	CG1074	chr3R	-3.18383005	2.16E-06
141551_at	FBgn0035987	CG3689	chr3L	-50.5630764	1.80E-07
141569_at	FBgn0039122	CG10192	chr3R	-4.88190638	7.32E-07
141572_at	FBgn0039590	CG10011	chr3R	-11.6802667	6.97E-05
141606_at	FBgn0028736	CG1333	chr3L	-3.84620749	2.11E-05
141611_at	FBgn0037373	CG2095	chr3R	-6.0036086	6.48E-09
141612_at	FBgn0036917	CG8013	chr3L	-211.251891	2.24E-08
141622_at	FBgn0036558	CG5841	chr3L	-3.926827	1.38E-07
141628_at	FBgn0024832	CG7057	chr3R	-71.3395788	1.94E-07
141647_at	FBgn0035436	CG12016	chr3L	-478.037959	2.75E-09
141652_at	FBgn0036772	CG5290	chr3L	-13.821787	0.00029841
141659_at	FBgn0038146	CG9799	chr3R	-4.09068722	0.00022397
141663_at	FBgn0036668	CG9715	chr3L	-4.50420999	9.32E-10
141666_at	FBgn0039279	CG11844	chr3R	-12.7649795	5.70E-08
141683_at	FBgn0038872	CG5874	chr3R	-67.2086695	1.80E-11
141693_at	FBgn0038168	CG9591	chr3R	-13.2464705	6.83E-07
141737_at	FBgn0036912	CG8491	chr3L	-23.4757154	2.10E-06
141763_at	FBgn0035235	CG7879	chr3L	-33.0174476	2.06E-06
141781_at	FBgn0038578	CG7957	chr3R	-9.42709107	3.10E-08
141788_at	FBgn0037622	CG8202	chr3R	-4.75792498	0.00080987
141791_at	FBgn0010379	CG4006	chr3R	-5.81548498	1.43E-07
141798_at	FBgn0025802	CG6939	chr3R	-21.8974673	5.21E-05
141800_at	FBgn0035149	CG17183	chr3L	-100.867929	1.65E-07
142179_s_at	FBgn0028495	CG18616	chr3R	-3.09328309	0.00013091
142207_at	FBgn0039189	CG18528	chr3R	-15.2793616	1.72E-05
142481_at	FBgn0037810	CG12819/CG1	chr3R	-4.74813447	2.27E-07

142483_at	FBgn0035846	CG7487	chr3L	-13.8250443	2.54E-05
142517_at	FBgn0035128	CG1225	chr3L	-62.8842812	1.26E-06
142531_at	FBgn0004926	CG4153	chr3L	-7.24760873	6.65E-10
142545_at	FBgn0010173	CG9633	chr3R	-41.1218548	2.05E-07
142599_at	FBgn0039011	CG31151	chr3R	-3.06700232	0.00018175
142675_at	FBgn0027512	CG10254	chr3R	-42.2416592	8.61E-07
142718_at	FBgn0027500	CG17286	chr3L	-4.21122399	7.46E-08
142758_at	FBgn0024326	CG9738	chr3R	-6.31442166	1.00E-07
142905_at	FBgn0036052	CG10809	chr3L	-6.23709399	7.01E-08
142992_at	FBgn0035236	CG12004	chr3L	-16.6393638	2.38E-10
142994_at	FBgn0038253	CG31385	chr3R	-5.36990027	1.76E-05
143147_at	FBgn0000565	CG7266	chr3L	-5.63039109	8.02E-06
143177_at	FBgn0001108	CG9206	chr3L	-3.64809726	0.00013775
143198_at	FBgn0001233	CG1242	chr3L	-8.19341157	6.14E-13
143225_at	FBgn0002183	CG1828	chr3L	-4.43006064	1.18E-06
143226_at	FBgn0002354	CG7620	chr3R	-9.09060022	4.45E-08
143293_at	FBgn0003041	CG8114	chr3L	-71.7924541	5.11E-15
143328_at	FBgn0003292	CG6097	chr3L	-12.8531693	3.34E-06
143335_at	FBgn0003334	CG9495	chr3R	-24.8102938	6.27E-06
143384_at	FBgn0003862	CG8651	chr3R	-8.95643394	2.45E-08
143547_at	FBgn0005585	CG9429	chr3R	-5.99441887	4.16E-11
143575_at	FBgn0005696	CG5923	chr3R	-72.0175552	2.54E-07
143628_at	FBgn0010438	CG4337	chr3R	-6.85348403	2.68E-09
143657_at	FBgn0011336	CG7748	chr3R	-16.1016926	1.10E-13
143738_at	FBgn0013998	CG33101	chr3R	-3.7541249	0.00015046
143740_at	FBgn0014006	CG4720	chr3R	-9.16275198	0.00033
143744_at	FBgn0014023	CG9378	chr3R	-8.72885927	1.40E-06
143802_at	FBgn0015351	CG14906	chr3R	-3.90006279	3.99E-06
143808_at	FBgn0015513	CG10379	chr3R	-99.4551528	1.20E-13
143844_at	FBgn0015793	CG7062	chr3L	-98.7198982	2.23E-07
143848_at	FBgn0015806	CG10539	chr3L	-5.92934968	3.05E-09
143981_at	FBgn0023395	CG9594	chr3L	-4.01642337	0.0001049
144123_at	FBgn0026371	CG7186	chr3L	-5.76934563	1.32E-09
147914_at	FBgn0035116	CG32346	chr3L	-7.75125854	0.00073227
147985_at	FBgn0035229	CG7852	chr3L	-79.9302359	3.43E-07
148003_at	FBgn0035272	CG13922	chr3L	-17.5948833	1.21E-06
148040_at	FBgn0035334	CG8993	chr3L	-14.3315972	0.00012635
148067_at	FBgn0035374	CG2101	chr3L	-5.06925664	1.52E-08
148076_at	FBgn0035390	CG1893	chr3L	-3.95115246	3.86E-07
148165_at	FBgn0035526	CG1316	chr3L	-5.89679293	1.44E-10
148166_at	FBgn0035529	CG1319	chr3L	-3.80750592	4.70E-07
148168_at	FBgn0035531	CG32250	chr3L	-3.73196985	2.17E-08
148169_at	FBgn0035532	CG15014	chr3L	-5.35253927	1.34E-07
148173_at	FBgn0035541	CG15019	chr3L	-13.3268317	5.74E-11
148260_at	FBgn0035674	CG13295	chr3L	-27.9819699	2.17E-05
148283_at	FBgn0035704	CG10144	chr3L	-7.68218015	2.55E-07
148394_at	FBgn0035877	CG7083	chr3L	-22.5729047	2.62E-06
148413_at	FBgn0035908	CG6817	chr3L	-4.12700367	4.08E-07
148469_at	FBgn0036007	CG3424	chr3L	-67.6284726	4.03E-08
148557_at	FBgn0036135	CG7636	chr3L	-20.4320164	3.28E-08
148582_r_at	FBgn0036171	CG11711	chr3L	-17.8502798	0.00017629

148672_at	FBgn0036308	CG32113	chr3L	-7.17109496	3.62E-05
148676_at	FBgn0036317	CG10948	chr3L	-27.0556607	0.00065788
148768_at	FBgn0036450	CG13472	chr3L	-14.2675799	1.20E-10
148886_at	FBgn0036613	CG13037	chr3L	-10.7910703	6.36E-06
148901_at	FBgn0036642	CG4169	chr3L	-4.29810353	5.10E-08
148903_at	FBgn0036644	CG4195	chr3L	-29.4802226	1.19E-09
148914_at	FBgn0036660	CG13025	chr3L	-8.12808714	4.45E-05
148950_at	FBgn0036721	CG3874	chr3L	-14.8181456	1.57E-11
149034_at	FBgn0036853	CG9730	chr3L	-9.24402498	5.65E-06
149035_at	FBgn0036855	CG14084	chr3L	-3.84351496	4.37E-06
149080_at	FBgn0036922	CG14182	chr3L	-38.6278905	1.19E-06
149106_at	FBgn0036958	NA	chr3L	-4.56834212	6.19E-07
149126_at	FBgn0036989	CG5231	chr3L	-3.45965393	0.00018991
149129_at	FBgn0036994	CG5199	chr3L	-3.56383662	0.00068895
149143_at	FBgn0037017	CG4074	chr3L	-188.283368	4.33E-08
149147_at	FBgn0037025	CG11451	chr3L	-15.7890098	0.00060652
149168_at	FBgn0037061	CG12975	chr3L	-4.41044811	1.22E-05
149195_at	FBgn0037110	CG14577	chr3L	-3.00786534	0.00017707
149347_at	FBgn0037363	CG1347	chr3R	-86.9399164	5.76E-08
149426_at	FBgn0037480	CG10061	chr3R	-8.77061053	0.00086623
149495_at	FBgn0037583	CG9684	chr3R	-51.5285486	3.02E-06
149547_at	FBgn0037674	CG8454	chr3R	-17.0273604	1.67E-05
149574_at	FBgn0037729	CG8362	chr3R	-13.8206841	1.82E-05
149625_at	FBgn0037802	CG6284	chr3R	-9.72031561	0.00021804
149681_at	FBgn0037890	CG17734	chr3R	-4.64939551	4.43E-07
149683_at	FBgn0037893	CG6719	chr3R	-4.32139832	6.47E-08
149696_at	FBgn0037922	CG14711	chr3R	-18.1464394	1.88E-05
149751_s_at	FBgn0038015	CG10042	chr3R	-20.3089188	3.78E-05
149779_at	FBgn0038053	CG18549	chr3R	-4.29076784	2.56E-06
149853_at	FBgn0038166	CG9588	chr3R	-4.31359258	0.00019852
149866_at	FBgn0038192	CG9924	chr3R	-3.62871348	1.40E-07
149907_r_at	FBgn0038243	CG8066	chr3R	-17.2063016	0.00012374
149979_at	FBgn0038369	CG4560	chr3R	-18.8557119	7.74E-09
150016_at	FBgn0038430	CG14895	chr3R	-7.07793251	1.11E-09
150135_at	FBgn0038609	CG7671	chr3R	-35.2769285	1.13E-09
150269_at	FBgn0038813	CG5206	chr3R	-41.1006593	9.19E-06
150389_at	FBgn0039000	CG6954	chr3R	-28.1555599	1.32E-06
150435_at	FBgn0039065	CG4449	chr3R	-12.2356349	1.58E-06
150436_at	FBgn0039067	CG4448	chr3R	-46.3099108	2.85E-06
150466_at	FBgn0039111	CG10371	chr3R	-5.32716069	2.16E-07
150477_at	FBgn0039139	CG5933	chr3R	-10.8109782	1.14E-09
150515_at	FBgn0039204	CG6607	chr3R	-7.40502018	3.10E-05
150518_at	FBgn0039216	CG31126	chr3R	-5.42856275	2.66E-07
150544_at	FBgn0039258	CG11780	chr3R	-5.82754792	0.00046267
150552_at	FBgn0039273	CG9996	chr3R	-109.10931	1.12E-07
150602_at	FBgn0039357	CG4743	chr3R	-3.02692998	2.75E-05
150641_at	FBgn0039404	CG14543	chr3R	-24.4260021	3.97E-06
150691_at	FBgn0039461	CG5500	chr3R	-3.99225583	0.0004066
150758_at	FBgn0039559	CG4976	chr3R	-13.7318575	1.56E-08
150761_at	FBgn0039565	CG4884	chr3R	-3.61788807	4.46E-06
150841_at	FBgn0039692	CG1973	chr3R	-7.90096969	0.00042031

151087_at	FBgn0040602	CG14545	chr3R	-6.52078008	2.21E-05
151529_at	GH15453.3prime-hit	CG7507	chr3L	-3.18366728	0.00074958
151585_at	GM06601.3prime-hit	CG32438	chr3L	-3.41645189	1.21E-05
151586_at	GM06806.3prime-hit	CG7369	chr3L	-21.7743921	4.65E-08
151637_at	LD13050.3prime-hit	NA	chr3L	-4.87900066	5.65E-06
151681_at	LD29569.3prime-hit	CG32306	chr3L	-13.6817005	2.16E-12
151728_at	LP02352.3prime-hit	CG18596	chr3R	-14.145169	7.97E-05
151737_at	SD01201.3prime-hit	CG32210	chr3L	-4.1904054	1.30E-07
151742_at	SD03655.3prime-hit	CG12004	chr3L	-72.0637194	2.58E-08
151758_at	SD09884.3prime-hit	CG11100	chr3L	-13.3559164	8.29E-05
151759_s_at	SD10401.3prime-hit	CG12819/CG1	chr3R	-8.34469057	2.38E-07
151761_at	SD10848.3prime-hit	CG3743	chr3L	-5.19338459	7.81E-08
151804_at	FBgn0037637	CG9836	chr3R	-26.9186128	2.80E-07
151807_at	FBgn0039537	CG5590	chr3R	-3.48874629	6.33E-06
151814_at	FBgn0035714	CG8549	chr3L	-10.7869362	1.73E-07
151840_at	FBgn0027615	CG6404	chr3L	-3.8306859	0.0006898
151904_at	FBgn0027598	CG31012	chr3R	-22.0242696	7.74E-09
151918_s_at	FBgn0027594	CG2086	chr3L	-13.1517449	7.63E-09
151926_at	FBgn0039348	CG4673	chr3R	-5.67250783	3.53E-08
151934_at	FBgn0028500	CG9063	chr3L	-6.27763612	1.46E-08
151982_at	FBgn0037747	CG8481	chr3R	-11.9731638	2.60E-05
152008_at	FBgn0039790	CG2246	chr3R	-4.48959162	0.00054273
152037_at	FBgn0035889	CG33057/CG7	chr3L	-50.0195108	2.36E-06
152067_at	FBgn0004913	CG1119	chr3R	-5.16226333	0.00023804
152158_at	FBgn0038877	CG3308	chr3R	-4.26633107	0.00017715
152177_at	FBgn0027565	CG5498	chr3L	-3.45331403	0.00098784
152192_at	FBgn0015622	CG11958	chr3R	-3.77512493	8.47E-09
152207_at	FBgn0038108	CG7518	chr3R	-4.27258499	1.34E-05
152237_at	FBgn0038301	CG6654	chr3R	-4.09840298	0.00017374
152253_at	FBgn0037216	CG12582	chr3R	-47.4009928	3.61E-07
152287_at	FBgn0026208	CG4143	chr3L	-8.65315367	8.24E-10
152311_at	FBgn0037049	CG32434	chr3L	-38.9835974	5.72E-14
152314_at	FBgn0036512	CG16979	chr3L	-3.95751246	0.0005911
152324_at	FBgn0037136	CG7148	chr3L	-6.13298298	8.94E-08
152327_at	FBgn0028484	CG14992	chr3L	-199.275146	7.53E-09
152354_at	FBgn0039714	CG7816	chr3R	-3.23009138	1.56E-05
152355_at	FBgn0015828	CG10415	chr3L	-4.87240826	1.20E-07
152383_at	FBgn0000116	CG32031	chr3L	-14.8880466	2.09E-08
152411_at	FBgn0037439	CG10286	chr3R	-7.83036737	3.19E-06
152417_at	FBgn0028684	CG10370	chr3R	-4.00751248	5.26E-08
152420_at	FBgn0022787	CG4261	chr3R	-12.4983551	5.22E-07
152443_at	FBgn0036253	CG11534	chr3L	-3.25557294	6.44E-05
152465_at	FBgn0017453	CG5215	chr3L	-10.8043958	1.21E-07
152530_at	FBgn0015270	CG3041	chr3R	-58.7143005	2.12E-06
152539_at	FBgn0037378	CG2046	chr3R	-6.92141328	8.53E-08
152563_at	FBgn0038803	CG5191	chr3R	-5.61402468	2.92E-08
152566_at	FBgn0037261	CG9775	chr3R	-63.9420318	2.00E-15
152621_at	FBgn0036366	CG10133	chr3L	-9.59079669	0.00010646
152631_at	FBgn0003691	CG12284	chr3L	-5.21456273	1.23E-09
152640_at	FBgn0035237	CG13917	chr3L	-64.380826	9.60E-08
152706_at	FBgn0038617	CG12333	chr3R	-13.0750112	1.86E-05

152735_at	FBgn0036136	CG6233	chr3L	-25.3420481	5.63E-07
152738_at	FBgn0027948	CG5000	chr3R	-26.8536381	1.03E-12
152758_at	FBgn0037709	CG8199	chr3R	-4.22030266	0.00010625
152769_at	FBgn0039715	CG7814	chr3R	-4.81839932	5.39E-05
152793_at	FBgn0037356	CG12170	chr3R	-26.7750137	4.38E-06
152811_at	FBgn0035244	CG7955	chr3L	-4.79347054	0.00019751
152812_at	FBgn0037663	CG9746	chr3R	-11.1600015	4.38E-07
152837_at	FBgn0039349	CG4685	chr3R	-6.00834245	6.76E-06
152845_at	FBgn0036405	CG6833	chr3L	-4.76504609	1.58E-07
152846_at	FBgn0010313	CG2530	chr3R	-3.10342951	1.52E-06
152864_at	FBgn0039135	CG13603	chr3R	-7.97822577	2.11E-07
152898_at	FBgn0011716	CG1539	chr3R	-156.522644	7.79E-10
152914_at	FBgn0036684	CG3764	chr3L	-22.7569308	5.60E-09
152917_at	FBgn0036137	CG7628	chr3L	-3.75636527	4.47E-06
152918_at	FBgn0037308	CG31543	chr3R	-7.64224726	6.18E-06
152923_at	FBgn0037044	CG10585	chr3L	-8.29097481	5.38E-07
152957_at	FBgn0036283	CG32103	chr3L	-4.16610475	2.17E-07
153017_at	FBgn0017429	CG5989	chr3L	-4.30482407	1.15E-07
153049_at	FBgn0037580	CG7415	chr3R	-42.4836803	2.75E-07
153052_at	FBgn0039296	CG10420	chr3R	-10.5663913	0.00017699
153065_at	FBgn0028582	CG8532	chr3L	-3.36124182	7.19E-06
153091_at	FBgn0025455	CG6292	chr3L	-3.75953828	4.17E-05
153107_at	FBgn0037384	CG10981	chr3R	-3.50792987	0.00080537
153180_at	FBgn0036534	CG6169	chr3L	-3.31462091	3.41E-05
153195_at	FBgn0014930	CG2846	chr3R	-4.67050155	0.00029155
153221_at	FBgn0039643	CG11886	chr3R	-54.7840752	6.66E-16
153228_at	FBgn0039578	CG14066	chr3R	-8.92103297	5.92E-08
153251_at	FBgn0036930	CG7749	chr3L	-6.75065342	1.44E-09
153282_at	FBgn0035896	CG6983	chr3L	-50.945423	5.32E-06
153295_at	FBgn0037901	CG6744	chr3R	-27.3669039	5.13E-06
153301_at	FBgn0004493	CG6349	chr3R	-5.8578805	4.11E-09
153349_at	FBgn0038608	CG7670	chr3R	-17.3181978	0.00017446
153373_at	FBgn0037607	CG8036	chr3R	-3.01139236	6.91E-06
153383_at	FBgn0037277	CG17735	chr3R	-5.92792014	1.24E-09
153407_at	FBgn0001247	CG5517	chr3L	-116.321314	2.67E-07
153497_at	FBgn0035105	CG7042	chr3L	-4.45292413	6.41E-05
153513_at	FBgn0035689	CG7376	chr3L	-4.58898078	3.79E-05
153514_at	FBgn0039169	CG5669	chr3R	-7.29460373	1.75E-06
153575_at	FBgn0035148	CG3402	chr3L	-16.1754852	2.84E-08
153576_at	FBgn0036182	CG6084	chr3L	-3.39094731	6.47E-07
153605_at	FBgn0039869	CG1890	chr3R	-10.6715125	1.80E-05
153606_at	FBgn0038760	CG12254	chr3R	-7.6043182	1.47E-07
153633_at	FBgn0023423	CG3412	chr3R	-3.19206736	0.00030407
153643_at	FBgn0038584	CG7175	chr3R	-3.55704069	5.15E-05
153645_at	FBgn0003600	CG6476	chr3R	-7.2697768	1.90E-11
153646_at	FBgn0027620	CG1966	chr3R	-5.88204952	1.03E-08
153699_at	FBgn0011016	CG5474	chr3L	-4.72972666	3.80E-06
153703_at	FBgn0000575	CG1007	chr3L	-28.6116219	7.29E-14
153704_at	FBgn0035267	CG13921	chr3L	-13.8959808	3.14E-10
153731_at	FBgn0001225	CG4183	chr3L	-6.87022511	4.27E-08
153758_at	FBgn0000338	CG17894	chr3R	-6.3182894	5.36E-06

153779_at	FBgn0000368	CG6383	chr3R	-43.6012168	3.77E-14
153963_at	FBgn0035114	CG33229	chr3L	-18.3155843	2.99E-13
153982_at	FBgn0015019	CG8977	chr3R	-19.1654369	4.77E-10
154005_at	FBgn0027531	CG7600	chr3L	-6.5108884	6.23E-05
154050_at	FBgn0027518	CG7609	chr3R	-4.72186928	0.00023118
154093_at	FBgn0004107	CG10498	chr3R	-3.11507067	1.70E-08
154103_at	FBgn0036299	CG10620	chr3L	-3.08488871	8.39E-08
154107_at	FBgn0038552	CG18012	chr3R	-4.09905479	4.33E-07
154111_at	FBgn0028692	CG11888	chr3R	-6.50248963	1.33E-08
154205_at	FBgn0039141	CG5977	chr3R	-7.65486373	9.74E-07
154293_at	FBgn0015660	CG4029	chr3R	-14.7611773	5.52E-10
154327_at	FBgn0020389	CG8363	chr3L	-5.32652292	1.22E-05
154388_at	FBgn0010340	CG9852	chr3R	-13.2158504	0.00029383
154402_at	FBgn0039589	CG9986	chr3R	-8.36851884	1.92E-09
154480_at	FBgn0027660	CG3897	chr3L	-80.8171429	2.48E-11
154482_at	FBgn0037556	CG9636	chr3R	-3.55528798	0.00081625
154532_at	FBgn0000629	CG6502	chr3L	-12.3806538	5.39E-07
154538_at	FBgn0036513	CG7764	chr3L	-3.07320579	5.39E-05
154549_at	FBgn0038433	CG14896	chr3R	-15.6998368	3.99E-09
154565_at	FBgn0037293	CG12007	chr3R	-40.4502075	5.50E-06
154604_at	FBgn0038304	CG12241	chr3R	-3.78297821	0.00041002
154631_at	FBgn0038103	CG31342	chr3R	-5.30978146	3.81E-05
154732_at	FBgn0039187	CG6454	chr3R	-8.94498551	2.12E-07
154739_at	FBgn0039144	CG5429	chr3R	-16.1428946	1.59E-08
154773_at	FBgn0013334	CG8884	chr3R	-8.85976931	1.59E-07
154798_at	FBgn0039499	CG18766	chr3R	-24.9565933	7.17E-06
154809_at	FBgn0039636	CG11877	chr3R	-3.30311479	3.55E-05
154866_at	FBgn0000140	CG6875	chr3R	-40.6535089	8.04E-14
154894_at	FBgn0016792	CG8374	chr3R	-13.4984849	8.08E-09
154920_at	FBgn0035388	CG2162	chr3L	-28.4034253	2.63E-10
154936_at	FBgn0038184	CG9285	chr3R	-4.19307008	4.81E-08
154946_at	FBgn0036761	CG5546	chr3L	-11.8729112	1.30E-11
154956_at	FBgn0039073	CG4408	chr3R	-67.9695793	1.52E-10
154969_at	FBgn0037644	CG11964	chr3R	-12.210826	8.90E-08
154996_at	FBgn0005674	CG5394	chr3R	-7.13525721	6.36E-06
155012_at	FBgn0038538	CG7660	chr3R	-11.7841504	3.99E-11
155014_at	FBgn0036988	CG5262	chr3L	-12.7370866	0.00032923
155022_at	FBgn0011771	CG5837	chr3L	-22.6944585	3.15E-05
155067_at	FBgn0036037	CG6754	chr3L	-37.2967608	0.00014658
155107_at	FBgn0022349	CG1910	chr3R	-3.6590837	1.18E-07
155131_at	FBgn0037719	CG9424	chr3R	-6.78198468	1.98E-09
155136_at	FBgn0037371	CG2097	chr3R	-11.8397666	2.74E-11
141427_at	FBgn0031098	CG17068	chrX	-7.70373642	1.86E-07
141464_at	FBgn0003048	CG3443	chrX	-17.2310616	0.00039711
141492_at	FBgn0030242	CG1655	chrX	-7.99579184	1.33E-05
141493_at	FBgn0000719	CG9559	chrX	-14.2250753	1.14E-06
141585_at	FBgn0031187	CG14619	chrX	-7.38541038	1.73E-08
141657_at	FBgn0030211	CG33175	chrX	-195.496528	1.43E-08
141681_at	FBgn0030235	CG1691	chrX	-6.76221333	9.05E-08
141692_at	FBgn0030650	CG33206	chrX	-5.03985751	8.55E-05
141713_at	FBgn0004643	CG9900	chrX	-144.034443	8.33E-08

141722_at	FBgn0004050	CG7803	chrX	-8.86456731	3.59E-07
141734_at	FBgn0030808	CG4937	chrX	-4.51163517	1.41E-05
141747_at	FBgn0030606	CG9053	chrX	-3.32067301	7.27E-05
141751_at	FBgn0023544	CG4399	chrX	-3.03320695	5.16E-05
141759_at	FBgn0020383	CG9209	chrX	-7.85318451	9.76E-07
141774_at	FBgn0031020	CG12202	chrX	-29.0485948	2.80E-05
142170_at	FBgn0011837	CG4070	chrX	-4.64834713	8.94E-07
142203_at	FBgn0027792	CG14788	chrX	-4.17320563	6.11E-06
142338_at	FBgn0004368	CG6899	chrX	-75.9655428	5.36E-07
142777_at	FBgn0004655	CG3707	chrX	-10.3999603	4.74E-05
142823_at	FBgn0020547	CG3000	chrX	-27.3904645	1.78E-05
142824_at	FBgn0022768	CG2984	chrX	-3.3494881	0.00058077
142871_s_at	FBgn0030734	CG9911	chrX	-5.19617535	0.00020357
142977_at	FBgn0030086	CG7033	chrX	-4.21094962	9.98E-06
143083_at	FBgn0000163	CG5055	chrX	-5.04484213	1.79E-05
143092_at	FBgn0000259	CG15224	chrX	-9.81028258	7.23E-07
143133_at	FBgn0000499	CG18361	chrX	-7.04322649	5.99E-09
143135_at	FBgn0000524	CG3929	chrX	-14.9630689	2.68E-05
143161_at	FBgn0000709	CG1484	chrX	-13.6673725	1.11E-06
143175_at	FBgn0001092	CG8893	chrX	-5.88567397	8.18E-07
143302_at	FBgn0003079	CG2845	chrX	-5.80554959	1.73E-05
143373_at	FBgn0003659	CG33070	chrX	-11.1950905	0.00013737
143735_at	FBgn0013987	CG3086	chrX	-4.58358372	4.49E-07
143963_at	FBgn0022724	CG7128	chrX	-10.7135105	7.51E-05
143994_at	FBgn0023518	CG3848	chrX	-3.12646093	0.00030834
144094_at	FBgn0025800	CG2262	chrX	-67.077694	1.18E-06
144114_at	FBgn0026076	CG9038	chrX	-20.0366511	1.79E-12
144179_at	FBgn0027359	CG1728	chrX	-3.7118292	8.05E-05
144245_at	FBgn0028686	CG16916	chrX	-3.0115401	1.66E-05
144430_at	FBgn0029650	CG3939	chrX	-3.3742879	1.47E-05
144485_at	FBgn0029718	CG7038	chrX	-5.61004717	2.92E-07
144536_at	FBgn0029786	CG3171	chrX	-9.8180299	4.40E-05
144548_at	FBgn0029800	CG15929	chrX	-11.446745	1.13E-09
144604_at	FBgn0029884	CG32742	chrX	-11.261254	0.00099076
144653_at	FBgn0029959	CG12156	chrX	-5.68515369	3.68E-08
144709_at	FBgn0030056	CG11284	chrX	-4.13641949	0.00013387
144730_at	FBgn0030090	CG12664	chrX	-4.51631278	0.00014123
144803_at	FBgn0030205	CG17255	chrX	-6.65455692	4.17E-05
144831_at	FBgn0030239	CG17333	chrX	-53.150914	4.11E-06
144867_at	FBgn0030291	CG1738	chrX	-248.808851	7.48E-09
144883_at	FBgn0030311	CG11699	chrX	-3.76661139	0.00036299
144889_at	FBgn0030320	CG2247	chrX	-3.0183665	6.38E-05
144972_at	FBgn0030434	CG4400	chrX	-7.80086984	3.03E-08
145056_at	FBgn0030552	CG15871	chrX	-5.98475793	0.00085688
145071_at	FBgn0030572	CG14413	chrX	-9.2460997	0.00019109
145095_at	FBgn0030602	CG32594	chrX	-29.4969329	1.36E-05
145131_at	FBgn0030659	CG9215	chrX	-3.05796915	0.00066398
145132_at	FBgn0030660	CG8097	chrX	-12.8439827	1.55E-09
145155_at	FBgn0030694	CG15602	chrX	-5.63444807	1.84E-05
145251_at	FBgn0030855	CG5800	chrX	-4.15960268	6.45E-06
145261_at	FBgn0030867	CG32560	chrX	-83.1382359	5.11E-08

145276_at	FBgn0030894	CG7192	chrX	-3.35314674	4.22E-05
145379_at	FBgn0031036	CG14220	chrX	-18.4675195	3.27E-05
145395_at	FBgn0031069	CG12703	chrX	-4.66864325	9.51E-07
151363_at	FBgn0040890	CG14199	chrX	-4.49344322	2.10E-05
151370_at	FBgn0040899	CG17776	chrX	-3.64514468	2.62E-06
151390_at	FBgn0040921	CG32736	chrX	-10.1851659	9.80E-10
151397_at	FBgn0040928	CG15345	chrX	-16.0409694	5.89E-06
151504_i_at	GH10316.complete-hit	CG3620	chrX	-4.37296259	5.68E-05
151557_i_at	GH23604.3prime-hit	CG32737	chrX	-28.6585657	5.18E-07
151606_at	HL07962.3prime-hit	CG1841	chrX	-32.0638072	2.50E-05
151610_at	LD03548.3prime-hit	CG3026	chrX	-3.04533784	0.00012442
151660_at	LD21771.3prime-hit	CG3429	chrX	-5.24830102	0.00013059
151740_at	SD02216.3prime-hit	CG7033	chrX	-6.91579298	2.87E-09
151790_at	FBgn0031174	CG1486	chrX	-9.82946318	5.20E-08
151908_at	FBgn0030668	CG8128	chrX	-4.34639434	0.00011296
151972_at	FBgn0030679	CG8206	chrX	-5.73766042	3.71E-07
152009_at	FBgn0030692	CG8470	chrX	-3.44198321	5.90E-06
152014_at	FBgn0025633	CG13366	chrX	-30.8594971	2.19E-08
152024_at	FBgn0029818	CG3033	chrX	-14.1410524	0.00026314
152069_at	FBgn0005410	CG1903	chrX	-84.1336236	7.96E-07
152239_at	FBgn0028486	CG2967	chrX	-94.2581514	3.49E-07
152259_at	FBgn0030671	CG8134	chrX	-25.0353521	3.72E-05
152404_at	FBgn0030327	CG2448	chrX	-25.597968	1.02E-11
152433_at	FBgn0026144	CG1435	chrX	-12.5162416	0.00058252
152453_at	FBgn0023169	CG3051	chrX	-3.9425463	9.05E-06
152691_at	FBgn0030061	CG1785	chrX	-4.00288985	1.75E-06
152692_at	FBgn0027621	CG3400	chrX	-29.2701542	6.86E-13
152858_at	FBgn0003302	CG4336	chrX	-3.58534673	7.98E-05
152953_at	FBgn0030411	CG2540	chrX	-4.29726727	1.40E-05
152977_at	FBgn0029666	CG10803	chrX	-31.7460712	6.98E-06
153035_at	FBgn0030028	CG10965	chrX	-14.0331782	6.13E-09
153044_at	FBgn0015565	CG2913	chrX	-9.58891716	1.11E-08
153060_at	FBgn0023526	CG2865	chrX	-125.11865	4.06E-08
153081_at	FBgn0011754	CG1830	chrX	-4.93847041	4.30E-05
153153_at	FBgn0015926	CG6157	chrX	-3.19118553	3.12E-05
153167_at	FBgn0030241	CG11207	chrX	-4.29043844	1.88E-07
153190_at	FBgn0030753	CG4420	chrX	-4.14602286	5.43E-06
153222_at	FBgn0030274	CG1908	chrX	-65.1094679	9.62E-07
153230_at	FBgn0024251	CG32527	chrX	-46.4826683	1.02E-05
153242_at	FBgn0023515	CG14814	chrX	-6.12898303	1.25E-07
153253_at	FBgn0031003	CG7889	chrX	-9.30963091	0.0006801
153332_at	FBgn0029958	CG12151	chrX	-28.5407505	2.14E-05
153463_at	FBgn0030243	CG2186	chrX	-5.66564306	1.29E-06
153517_at	FBgn0029970	CG17256	chrX	-4.46213839	8.63E-06
153571_at	FBgn0026751	CG4208	chrX	-3.5914128	0.00094066
153592_at	FBgn0024332	CG4206	chrX	-5.50541107	9.49E-06
153647_at	FBgn0014133	CG1822	chrX	-3.67754729	4.41E-05
153675_at	FBgn0003964	CG4380	chrX	-90.8905591	6.18E-07
153695_at	FBgn0030878	CG6769	chrX	-6.10553392	3.43E-05
153702_at	FBgn0031046	CG12238	chrX	-16.461876	7.38E-08
153708_at	FBgn0029516	CG2995	chrX	-18.9127356	0.00012197

153751_at	FBgn0030003	CG2116	chrX	-5.24172132	3.53E-06
153784_at	FBgn0023130	CG3771	chrX	-3.98015562	0.00033143
153789_at	FBgn0029882	CG3226	chrX	-4.09169511	1.40E-06
153790_at	FBgn0029721	CG7010	chrX	-6.11392144	1.28E-05
153917_at	FBgn0023536	CG3156	chrX	-9.43593064	0.00094116
153942_at	FBgn0030710	CG8924	chrX	-4.98442652	2.47E-06
154041_at	FBgn0027521	CG3679	chrX	-8.55960493	6.30E-08
154060_at	FBgn0011606	CG8590	chrX	-4.15023919	1.77E-05
154096_at	FBgn0029737	CG6903	chrX	-3.4799539	7.15E-06
154099_at	FBgn0000257	CG12230	chrX	-26.1093028	3.23E-05
154137_at	FBgn0010315	CG9096	chrX	-3.59018154	3.54E-05
154188_at	FBgn0030817	CG4991	chrX	-10.432017	1.32E-05
154221_at	FBgn0011761	CG4193	chrX	-31.1837497	3.90E-11
154261_at	FBgn0030973	CG7332	chrX	-5.37043855	2.30E-06
154279_at	FBgn0029680	CG2934	chrX	-3.05584561	1.70E-05
154351_at	FBgn0020369	CG1489	chrX	-3.90184531	1.21E-07
154417_at	FBgn0030146	CG32697	chrX	-3.50706722	0.00019675
154424_at	FBgn0028469	CG8062	chrX	-3.5038064	0.00050928
154460_at	FBgn0030557	CG12047	chrX	-5.66351813	1.20E-07
154462_at	FBgn0030849	CG5675	chrX	-17.9995102	0.00011374
154489_at	FBgn0000376	CG2714	chrX	-4.08525289	0.00012508
154490_at	FBgn0031118	CG1412	chrX	-5.19958004	8.70E-06
154558_at	FBgn0026080	CG6121	chrX	-6.76610034	6.52E-08
154638_at	FBgn0031062	CG14230	chrX	-4.05244528	2.95E-05
154705_at	FBgn0031061	CG14232	chrX	-21.3615787	1.39E-06
154709_at	FBgn0030321	CG1703	chrX	-4.37184666	4.07E-06
154712_at	FBgn0030871	CG8142	chrX	-6.00454192	1.04E-07
154744_at	FBgn0026143	CG3658	chrX	-21.5292929	6.24E-05
154770_at	FBgn0030854	CG8289	chrX	-6.52832555	5.70E-07
154841_at	FBgn0030435	CG4645	chrX	-4.58590704	3.10E-07
154939_at	FBgn0010269	CG15793	chrX	-8.42382201	6.28E-09
155024_at	FBgn0031091	CG9576	chrX	-7.63494578	8.41E-05
155026_at	FBgn0030612	CG5599	chrX	-6.89258624	7.57E-06
155028_at	FBgn0023527	CG3071	chrX	-5.65747889	8.57E-07
155109_at	FBgn0025617	CG7359	chrX	-3.14204313	0.00034484
155132_at	FBgn0000520	CG2711	chrX	-15.1576915	4.01E-05
