# Supplementary material for: Unmasking Activation of the Zygotic Genome Using Chromosomal Deletions in the Drosophila Embryo
Source: PLoS Biol. 2007 Apr 24;5(5):e117. doi: 10.1371/journal.pbio.0050117 (PMC1854917; doi:10.1371/journal.pbio.0050117)
Supplement: Supplementary file 8 [file pbio.0050117.st008.pdf]

Early Zygotic Transcripts (1-2h)

affy_probe_set_id	fbgn for the probe se	CG id	Chr.Location	fold-changes	p-value
141541_at	FBgn0034501	CG13868	chr2R	106.2977486	2.74E-06
142451_at	FBgn0036914	NA	chr3L	3.751645659	0.009098677
142780_at	FBgn0035310	CG5704	chr3L	3.173375377	0.001788397
142969_at	FBgn0004629	CG8050	chr3R	7.122217293	0.002991341
143088_at	FBgn0000216	CG3096	chr3L	15.8065678	1.41E-06
143089_at	FBgn0000227	CG12205	chr2L	93.78592388	2.97E-07
143186_at	FBgn0001168	CG6494	chr3L	69.57520386	3.52E-06
143219_at	FBgn0001981	CG3758	chr2L	85.28228322	7.15E-08
143237_at	FBgn0002561	CG3839	chrX	69.592984	4.03E-07
143251_at	FBgn0002629	CG6099	chr3R	43.65313879	8.47E-07
143331_at	FBgn0003313	CG4922	chr2L	19.54556983	5.78E-05
143356_at	FBgn0003448	CG3956	chr2L	13.81441532	1.00E-05
143367_at	FBgn0003510	CG17957	chr3R	16.49327007	2.94E-07
143379_at	FBgn0003720	CG1378	chr3R	99.29148118	1.49E-07
143386_at	FBgn0003865	CG1502	chrX	5.482707671	2.50E-05
143420_at	FBgn0004053	CG1046	chr3R	7.482331504	1.43E-06
143421_at	FBgn0004054	CG1048	chr3R	9.24459959	0.000272835
143429_at	FBgn0004143	CG14426	chrX	15.09047701	1.36E-07
143431_at	FBgn0004170	CG3827	chrX	27.68849182	3.98E-07
143718_at	FBgn0013755	CG7960	chr3L	32.9721686	9.11E-10
144171_at	FBgn0027104	CG10393	chr2L	46.61801792	1.95E-07
144639_at	FBgn0029931	CG14427	chrX	18.01449184	2.58E-05
145222_at	FBgn0030807	CG13000	chrX	234.6998471	9.01E-09
145411_at	FBgn0031086	CG9571	chrX	14.57094688	3.25E-05
145586_at	FBgn0031346	CG7428	chr2L	30.1196329	1.02E-08
145621_at	FBgn0031393	CG15382	chr2L	100.4752474	2.16E-08
145830_at	FBgn0031718	CG14014	chr2L	39.37860458	8.70E-07

145831_at	FBgn0031719	CG18269	chr2L	295.0113366	5.79E-09
146224_at	FBgn0032335	CG14915	chr2L	5.760022294	0.00059541
146318_at	FBgn0032489	CG15480	chr2L	5.354847431	0.000589374
146320_at	FBgn0032491	CG16815	chr2L	6.820755598	2.69E-05
146647_at	FBgn0033030	CG1294	chr2R	3.570475987	0.004000385
147511_at	FBgn0034465	CG9854	chr2R	5.458761423	0.000183164
147549_at	FBgn0034514	CG13427	chr2R	105.1234345	3.44E-08
148164_at	FBgn0035525	CG11582	chr3L	31.00967527	6.08E-06
148191_at	FBgn0035563	CG13716	chr3L	19.03949893	1.47E-05
148194_at	FBgn0035569	CG15876	chr3L	76.50399653	2.86E-09
148195_at	FBgn0035570	CG13712	chr3L	54.74238015	1.04E-06
148197_at	FBgn0035572	CG13711	chr3L	147.5124221	1.57E-08
148227_at	FBgn0035618	CG10594	chr3L	73.88362941	1.17E-06
148702_at	FBgn0036349	CG14112	chr3L	27.5691682	7.46E-07
148998_s_at	FBgn0036791	CG4216/CG7271	chr3L	319.0642283	5.24E-09
149010_at	FBgn0036810	CG6885	chr3L	16.3711046	9.20E-07
149180_at	FBgn0037085	CG7204	chr3L	241.9907404	1.26E-08
150114_at	FBgn0038566	CG14317	chr3R	18.52652667	2.98E-06
150828_at	FBgn0039675	CG12048	chr3R	3.105986246	0.052350492
151178_at	FBgn0040701	NA	chr3L	63.40260495	1.42E-07
151179_at	FBgn0040702	CG13713	chr3L	87.4827874	1.62E-07
151283_s_at	FBgn0040808	CG12487/CG1346	chr3L	169.5327078	2.57E-08
151444_at	FBgn0040984	CG4440	chr2L	32.3237064	1.04E-07
151661_s_at	LD22004.3prime-hit	CG9704	chr3L	8.526249567	8.30E-05
151863_at	FBgn0003300	CG1849	chrX	12.47142531	0.000151215
151981_at	FBgn0003430	CG16738	chr2L	10.74255166	1.46E-05
152552_at	FBgn0039423	CG31085	chr3R	3.168483223	0.055632279
152877_at	FBgn0035315	CG8960	chr3L	160.6189159	1.04E-07
153088_at	FBgn0035770	CG8588	chr3L	5.017143784	0.011063532

153558_at	FBgn0026320	CG5185	chr3L	10.42424675	5.14E-05
154420_at	FBgn0005771	CG4491	chr2L	15.59224601	0.001747756
154983_at	FBgn0031621	CG15634	chr2L	78.09745992	9.36E-07
