# Supplementary material for: Unmasking Activation of the Zygotic Genome Using Chromosomal Deletions in the Drosophila Embryo
Source: PLoS Biol. 2007 Apr 24;5(5):e117. doi: 10.1371/journal.pbio.0050117 (PMC1854917; doi:10.1371/journal.pbio.0050117)
Supplement: Supplementary file 9 [file pbio.0050117.st009.pdf]

Table S9:Densities of CAGGTAG (and variants) occurrences within experimentally verified cis-regulatory modules (CRMs)

<table>
<tr><th># of CAGGATG/kb</th><th>CRM length (bp)</th><th># of CAGGATG</th><th>CRM name (RedFly)</th></tr>
<tr><td>7.42</td><td>539</td><td>4</td><td>dpp dl mel|Drosophila melanogaster|dpp|FBgn0000490|2L:2456345..2456884</td></tr>
<tr><td>5.56</td><td>540</td><td>3</td><td>Kr StH0.6HZ|Drosophila melanogaster|Kr|FBgn0001325|2R:20730653..20731193</td></tr>
<tr><td>5.43</td><td>368</td><td>2</td><td>tll D3|Drosophila melanogaster|tll|FBgn0003720|3R:26677662..26678030</td></tr>
<tr><td>5.1</td><td>392</td><td>2</td><td>sog broad lateral neurogenic ectoderm|Drosophila melanogaster|sog|FBgn0003463|X:15458634..15459026</td></tr>
<tr><td>4.75</td><td>631</td><td>3</td><td>sim mesectoderm|Drosophila melanogaster|sim|FBgn0004666|3R:8895835..8896466</td></tr>
<tr><td>4.02</td><td>497</td><td>2</td><td>vn neurogenic ectoderm|Drosophila melanogaster|vn|FBgn0003984|3L:5809617..5810114</td></tr>
<tr><td>3.54</td><td>1130</td><td>4</td><td>Kr StBg1.2HZ|Drosophila melanogaster|Kr|FBgn0001325|2R:20730653..20731783</td></tr>
<tr><td>3.45</td><td>1159</td><td>4</td><td>Kr CD1|Drosophila melanogaster|Kr|FBgn0001325|2R:20730218..20731377</td></tr>
<tr><td>3.4</td><td>589</td><td>2</td><td>hkb ventral element plus|Drosophila melanogaster|hkb|FBgn0001204|3R:173890..174479</td></tr>
<tr><td>3.22</td><td>622</td><td>2</td><td>slp1 5-3|Drosophila melanogaster|slp1|FBgn0003430|2L:3821715..3822337</td></tr>
<tr><td>3.21</td><td>624</td><td>2</td><td>zen dorsal ectoderm|Drosophila melanogaster|zen|FBgn0004053|3R:2580903..2581527</td></tr>
<tr><td>3.1</td><td>968</td><td>3</td><td>sna 0.9|Drosophila melanogaster|sna|FBgn0003448|2L:15474099..15475067</td></tr>
<tr><td>2.8</td><td>1073</td><td>3</td><td>btd Ss-Bg|Drosophila melanogaster|btd|FBgn0000233|X:9535642..9536715</td></tr>
<tr><td>2.75</td><td>726</td><td>2</td><td>zen 0.7|Drosophila melanogaster|zen|FBgn0004053|3R:2579865..2580591</td></tr>
<tr><td>2.75</td><td>2912</td><td>8</td><td>sna 2.8kb|Drosophila melanogaster|sna|FBgn0003448|2L:15474099..15477011</td></tr>
<tr><td>2.74</td><td>730</td><td>2</td><td>hb 0.7|Drosophila melanogaster|hb|FBgn0001180|3R:4519890..4520620</td></tr>
<tr><td>2.68</td><td>745</td><td>2</td><td>ftz zebra element|Drosophila melanogaster|ftz|FBgn0001077|3R:2689374..2690119</td></tr>
<tr><td>2.64</td><td>1513</td><td>4</td><td>zen 1.4|Drosophila melanogaster|zen|FBgn0004053|3R:2579865..2581378</td></tr>
<tr><td>2.63</td><td>760</td><td>2</td><td>ind moduleC+|Drosophila melanogaster|ind|FBgn0025776|3L:15005234..15005994</td></tr>
<tr><td>2.52</td><td>1983</td><td>5</td><td>dpp intron2|Drosophila melanogaster|dpp|FBgn0000490|2L:2455781..2457764</td></tr>
<tr><td>2.51</td><td>797</td><td>2</td><td>tld promoterfusion|Drosophila melanogaster|tld|FBgn0003719|3R:20574722..20575519</td></tr>
<tr><td>2.5</td><td>799</td><td>2</td><td>miR-309-6 8-miR enhancer|Drosophila melanogaster|miR-309-6|FBgn0067695|2R:15176486..15177285</td></tr>
<tr><td>2.28</td><td>876</td><td>2</td><td>h stripe1|Drosophila melanogaster|h|FBgn0001168|3L:8644871..8645747</td></tr>
<tr><td>2.12</td><td>1416</td><td>3</td><td>ind 1.4|Drosophila melanogaster|ind|FBgn0025776|3L:15004578..15005994</td></tr>
<tr><td>2.11</td><td>950</td><td>2</td><td>Kr H/I|Drosophila melanogaster|Kr|FBgn0001325|2R:20733665..20734615</td></tr>
<tr><td>2</td><td>1500</td><td>3</td><td>Kr H/J|Drosophila melanogaster|Kr|FBgn0001325|2R:20731858..20733358</td></tr>
<tr><td>1.95</td><td>2567</td><td>5</td><td>sli 2.6 RV|Drosophila melanogaster|sli|FBgn0003425|2R:11405651..11408218</td></tr>
<tr><td>1.95</td><td>2560</td><td>5</td><td>ato 5'F2.6|Drosophila melanogaster|ato|FBgn0010433|3R:4101301..4103861</td></tr>
<tr><td>1.89</td><td>1587</td><td>3</td><td>Kr Kr/F|Drosophila melanogaster|Kr|FBgn0001325|2R:20733979..20735566</td></tr>
<tr><td>1.85</td><td>1082</td><td>2</td><td>ind moduleBC|Drosophila melanogaster|ind|FBgn0025776|3L:15004912..15005994</td></tr>
<tr><td>1.82</td><td>1098</td><td>2</td><td>gt CE8001|Drosophila melanogaster|gt|FBgn0001150|X:2286739..2287837</td></tr>
<tr><td>1.81</td><td>2764</td><td>5</td><td>tll P2|Drosophila melanogaster|tll|FBgn0003720|3R:26675737..26678501</td></tr>
<tr><td>1.76</td><td>1707</td><td>3</td><td>Kr NcS1.7HZ|Drosophila melanogaster|Kr|FBgn0001325|2R:20731651..20733358</td></tr>
<tr><td>1.76</td><td>1706</td><td>3</td><td>vnd early embryonic enhancer|Drosophila melanogaster|vnd|FBgn0003986|X:448770..450476</td></tr>
<tr><td>1.74</td><td>1725</td><td>3</td><td>tll P3|Drosophila melanogaster|tll|FBgn0003720|3R:26676776..26678501</td></tr>
<tr><td>1.72</td><td>1745</td><td>3</td><td>h stripe 3+4 ET22|Drosophila melanogaster|h|FBgn0001168|3L:8637476..8639221</td></tr>
<tr><td>1.72</td><td>1162</td><td>2</td><td>CG32150 PE|Drosophila melanogaster|CG32150|FBgn0052150|3L:15811786..15812948</td></tr>
<tr><td>1.67</td><td>1196</td><td>2</td><td>Pgd -421.lacZ|Drosophila melanogaster|Pgd|FBgn0004654|X:2000354..2001550</td></tr>
<tr><td>1.65</td><td>1213</td><td>2</td><td>gt gt23|Drosophila melanogaster|gt|FBgn0001150|X:2294438..2295651</td></tr>
<tr><td>1.65</td><td>1212</td><td>2</td><td>nub CE8011|Drosophila melanogaster|nub|FBgn0002970|2L:12616132..12617344</td></tr>
<tr><td>1.65</td><td>1209</td><td>2</td><td>gt -3 construct|Drosophila melanogaster|gt|FBgn0001150|X:2286416..2287625</td></tr>
<tr><td>1.61</td><td>1861</td><td>3</td><td>oc otd early enhancer|Drosophila melanogaster|oc|FBgn0004102|X:8499138..8500999</td></tr>
<tr><td>1.6</td><td>1253</td><td>2</td><td>ftz ftzDE|Drosophila melanogaster|ftz|FBgn0001077|3R:2693514..2694767</td></tr>
<tr><td>1.49</td><td>3353</td><td>5</td><td>chn CG11798-PE|Drosophila melanogaster|chn|FBgn0015371|2R:10645741..10649094</td></tr>
<tr><td>1.45</td><td>1384</td><td>2</td><td>odd -5 construct|Drosophila melanogaster|odd|FBgn0002985|2L:3610419..3611803</td></tr>
<tr><td>1.44</td><td>2769</td><td>4</td><td>dpn dC|Drosophila melanogaster|dpn|FBgn0010109|2R:3743511..3746280</td></tr>
<tr><td>1.43</td><td>1401</td><td>2</td><td>prd O-E|Drosophila melanogaster|prd|FBgn0003145|2L:12080487..12081888</td></tr>
<tr><td>1.41</td><td>1421</td><td>2</td><td>hb HZ1.4|Drosophila melanogaster|hb|FBgn0001180|3R:4526521..4527942</td></tr>
<tr><td>1.41</td><td>1415</td><td>2</td><td>twi dl mel|Drosophila melanogaster|twi|FBgn0003900|2R:18552504..18553919</td></tr>
<tr><td>1.4</td><td>1432</td><td>2</td><td>ftz CE8024|Drosophila melanogaster|ftz|FBgn0001077|3R:2693264..2694696</td></tr>
<tr><td>1.4</td><td>1424</td><td>2</td><td>hb upstream enhancer|Drosophila melanogaster|hb|FBgn0001180|3R:4526521..4527945</td></tr>
<tr><td>1.34</td><td>1487</td><td>2</td><td>Brd 1.5|Drosophila melanogaster|Brd|FBgn0000216|3L:14936477..14937964</td></tr>
<tr><td>1.33</td><td>3001</td><td>4</td><td>h V-h|Drosophila melanogaster|h|FBgn0001168|3L:872601..875602</td></tr>
<tr><td>1.31</td><td>1523</td><td>2</td><td>cluster at 55C CE8016|Drosophila melanogaster|cluster at 55C||2R:13805197..13806720</td></tr>
<tr><td>1.27</td><td>1570</td><td>2</td><td>prd zebra enhancer|Drosophila melanogaster|prd|FBgn0003145|2L:12084451..12086021</td></tr>
<tr><td>1.23</td><td>1623</td><td>2</td><td>pdm2 +1 construct|Drosophila melanogaster|pdm2|FBgn0004394|2L:12678897..12680520</td></tr>
<tr><td>1.22</td><td>7381</td><td>9</td><td>Pgd -1172+6.2|Drosophila melanogaster|Pgd|FBgn0004654|X:2000354..2007735</td></tr>
<tr><td>1.21</td><td>1648</td><td>2</td><td>sli 1.5 BH|Drosophila melanogaster|sli|FBgn0003425|2R:11401369..11403017</td></tr>
<tr><td>1.18</td><td>5091</td><td>6</td><td>run 7stripe 5kb|Drosophila melanogaster|run|FBgn0003300|X:20500025..20505116</td></tr>
<tr><td>1.17</td><td>6849</td><td>8</td><td>sli 6.7 BR|Drosophila melanogaster|sli|FBgn0003425|2R:11401369..11408218</td></tr>
<tr><td>1.17</td><td>4286</td><td>5</td><td>aop A-lacZ|Drosophila melanogaster|aop|FBgn0000097|2L:2181550..2185836</td></tr>
<tr><td>1.17</td><td>1708</td><td>2</td><td>fkh -2 construct|Drosophila melanogaster|fkh|FBgn0000659|3R:24411718..24413426</td></tr>
<tr><td>1.17</td><td>1705</td><td>2</td><td>Ubx bx1|Drosophila melanogaster|Ubx|FBgn0003944|3R:12525924..12527629</td></tr>
<tr><td>1.15</td><td>1745</td><td>2</td><td>gt -10 construct|Drosophila melanogaster|gt|FBgn0001150|X:2293911..2295656</td></tr>
<tr><td>1.15</td><td>1745</td><td>2</td><td>abd-A iab-2(1.7)|Drosophila melanogaster|abd-A|FBgn0000014|3R:12636229..12637974</td></tr>
<tr><td>1.15</td><td>1743</td><td>2</td><td>Kr SN1.7KrZ|Drosophila melanogaster|Kr|FBgn0001325|2R:20733357..20735100</td></tr>
<tr><td>1.13</td><td>2646</td><td>3</td><td>run 7stripe reduced|Drosophila melanogaster|run|FBgn0003300|X:20502470..20505116</td></tr>
<tr><td>1.08</td><td>2770</td><td>3</td><td>Obp99a prom|Drosophila melanogaster|Obp99a|FBgn0039678|3R:25494672..25497442</td></tr>
<tr><td>1.06</td><td>1894</td><td>2</td><td>siz loner/CG32434-PE|Drosophila melanogaster|siz|FBgn0026179|3L:20999788..21001682</td></tr>
<tr><td>1.04</td><td>4790</td><td>5</td><td>sli 4.8 BV|Drosophila melanogaster|sli|FBgn0003425|2R:11405651..11410441</td></tr>
<tr><td>1.03</td><td>2900</td><td>3</td><td>sna dl mel|Drosophila melanogaster|sna|FBgn0003448|2L:15469704..15472604</td></tr>
<tr><td>1.01</td><td>1985</td><td>2</td><td>nub -2 construct|Drosophila melanogaster|nub|FBgn0002970|2L:12615791..12617776</td></tr>
<tr><td>1</td><td>3001</td><td>3</td><td>Obp19c prom|Drosophila melanogaster|Obp19c|FBgn0031111|X:20229403..20232404</td></tr>
<tr><td>0.93</td><td>7491</td><td>7</td><td>ato 5'F7.2|Drosophila melanogaster|ato|FBgn0010433|3R:4096370..4103861</td></tr>
<tr><td>0.9</td><td>5527</td><td>5</td><td>ato 5'F5.1|Drosophila melanogaster|ato|FBgn0010433|3R:4098334..4103861</td></tr>
<tr><td>0.89</td><td>6775</td><td>6</td><td>aop D-lacZ|Drosophila melanogaster|aop|FBgn0000097|2L:2172660..2179435</td></tr>
<tr><td>0.87</td><td>2305</td><td>2</td><td>aop C-lacZ|Drosophila melanogaster|aop|FBgn0000097|2L:2174335..2176640</td></tr>
<tr><td>0.87</td><td>10339</td><td>9</td><td>ato 5'F9.3|Drosophila melanogaster|ato|FBgn0010433|3R:4093522..4103861</td></tr>
<tr><td>0.86</td><td>6984</td><td>6</td><td>Scr 6.5KS|Drosophila melanogaster|Scr|FBgn0003339|3R:2683633..2690617</td></tr>
<tr><td>0.86</td><td>2329</td><td>2</td><td>rst F6d|Drosophila melanogaster|rst|FBgn0003285|X:2839593..2841922</td></tr>
<tr><td>0.84</td><td>2380</td><td>2</td><td>repo -4.3/-1.9|Drosophila melanogaster|repo|FBgn0011701|3R:14057605..14059985</td></tr>
<tr><td>0.83</td><td>2399</td><td>2</td><td>PFE PE|Drosophila melanogaster|PFE|FBgn0032661|2L:18009141..18011540</td></tr>
<tr><td>0.82</td><td>6067</td><td>5</td><td>sc DC|Drosophila melanogaster|sc|FBgn0004170|X:216690..222757</td></tr>
<tr><td>0.82</td><td>4874</td><td>4</td><td>gcm -4.9|Drosophila melanogaster|gcm|FBgn0014179|2L:9581741..9586615</td></tr>
<tr><td>0.8</td><td>2513</td><td>2</td><td>unpg U6|Drosophila melanogaster|unpg|FBgn0015561|2R:4711060..4713573</td></tr>
<tr><td>0.8</td><td>2503</td><td>2</td><td>Obp19a prom|Drosophila melanogaster|Obp19a|FBgn0031109|X:20223943..20226446</td></tr>
<tr><td>0.76</td><td>2640</td><td>2</td><td>slp2 -3 construct|Drosophila melanogaster|slp2|FBgn0004567|2L:3832697..3835337</td></tr>
<tr><td>0.75</td><td>6688</td><td>5</td><td>y D-1868|Drosophila melanogaster|y|FBgn0004034|X:211463..218151</td></tr>
<tr><td>0.75</td><td>2664</td><td>2</td><td>salm BAT|Drosophila melanogaster|salm|FBgn0004579|2L:11410694..11413358</td></tr>
<tr><td>0.74</td><td>5395</td><td>4</td><td>Scr 5.4BB|Drosophila melanogaster|Scr|FBgn0003339|3R:2660685..2666080</td></tr>
<tr><td>0.72</td><td>4183</td><td>3</td><td>Dll 179|Drosophila melanogaster|Dll|FBgn0000157|2R:20314822..20319005</td></tr>
<tr><td>0.72</td><td>4156</td><td>3</td><td>EcR AB|Drosophila melanogaster|EcR|FBgn0000546|2R:1686466..1690622</td></tr>
<tr><td>0.7</td><td>8588</td><td>6</td><td>Scr BSR|Drosophila melanogaster|Scr|FBgn0003339|3R:2667896..2676484</td></tr>
<tr><td>0.67</td><td>3002</td><td>2</td><td>Obp57a prom|Drosophila melanogaster|Obp57a|FBgn0043535|2R:16017031..16020033</td></tr>
<tr><td>0.67</td><td>3001</td><td>2</td><td>Obp83ef Obp83f prom|Drosophila melanogaster|Obp83ef|FBgn0046876|3R:1936728..1939729</td></tr>
<tr><td>0.67</td><td>3001</td><td>2</td><td>Obp56e prom|Drosophila melanogaster|Obp56e|FBgn0034471|2R:15224260..15227261</td></tr>
<tr><td>0.67</td><td>3001</td><td>2</td><td>Obp19b prom|Drosophila melanogaster|Obp19b|FBgn0031110|X:20224439..20227440</td></tr>
<tr><td>0.66</td><td>3051</td><td>2</td><td>Ser IV-3.0|Drosophila melanogaster|Ser|FBgn0004197|3R:23013610..23016661</td></tr>
<tr><td>0.66</td><td>3020</td><td>2</td><td>Obp56a prom|Drosophila melanogaster|Obp56a|FBgn0034468|2R:15209629..15212649</td></tr>
<tr><td>0.65</td><td>7692</td><td>5</td><td>y D-2873|Drosophila melanogaster|y|FBgn0004034|X:210459..218151</td></tr>
<tr><td>0.62</td><td>3244</td><td>2</td><td>drl drlT|Drosophila melanogaster|drl|FBgn0015380|2L:19189646..19192890</td></tr>
<tr><td>0.61</td><td>4912</td><td>3</td><td>y D-91|Drosophila melanogaster|y|FBgn0004034|X:213239..218151</td></tr>
<tr><td>0.59</td><td>3401</td><td>2</td><td>Ubx dorsal imaginal disc enhancer|Drosophila melanogaster|Ubx|FBgn0003944|3R:12595044..12598445</td></tr>
<tr><td>0.58</td><td>6878</td><td>4</td><td>run 6stripe +3GG|Drosophila melanogaster|run|FBgn0003300|X:20507938..20514816</td></tr>
<tr><td>0.58</td><td>5205</td><td>3</td><td>btd RV-2ndB|Drosophila melanogaster|btd|FBgn0000233|X:9534641..9539846</td></tr>
<tr><td>0.57</td><td>5233</td><td>3</td><td>mira PE|Drosophila melanogaster|mira|FBgn0021776|3R:15757262..15762495</td></tr>
<tr><td>0.56</td><td>5394</td><td>3</td><td>drl drlW|Drosophila melanogaster|drl|FBgn0015380|2L:19189646..19195040</td></tr>
<tr><td>0.56</td><td>5316</td><td>3</td><td>y D-495|Drosophila melanogaster|y|FBgn0004034|X:212835..218151</td></tr>
<tr><td>0.53</td><td>5616</td><td>3</td><td>gcm -29/-24|Drosophila melanogaster|gcm|FBgn0014179|2L:9605401..9611017</td></tr>
<tr><td>0.53</td><td>3809</td><td>2</td><td>Dll 207|Drosophila melanogaster|Dll|FBgn0000157|2R:20305645..20309454</td></tr>
<tr><td>0.5</td><td>7934</td><td>4</td><td>Ubx pair rule (zebra) like S1 enhancer|Drosophila melanogaster|Ubx|FBgn0003944|3R:12581426..12589360</td></tr>
<tr><td>0.48</td><td>6255</td><td>3</td><td>gsb 2.8S|Drosophila melanogaster|gsb|FBgn0001148|2R:20564953..20571208</td></tr>
<tr><td>0.45</td><td>6731</td><td>3</td><td>ato 3'F5.8|Drosophila melanogaster|ato|FBgn0010433|3R:4104814..4111545</td></tr>
<tr><td>0.45</td><td>6720</td><td>3</td><td>Tl 6.5RL/LR|Drosophila melanogaster|Tl|FBgn0003717|3R:22615402..22622122</td></tr>
<tr><td>0.45</td><td>6606</td><td>3</td><td>sc ANP|Drosophila melanogaster|sc|FBgn0004170|X:286525..293131</td></tr>
<tr><td>0.43</td><td>11579</td><td>5</td><td>EcR AG|Drosophila melanogaster|EcR|FBgn0000546|2R:1679043..1690622</td></tr>
<tr><td>0.42</td><td>4803</td><td>2</td><td>run 6stripe +1EE|Drosophila melanogaster|run|FBgn0003300|X:20506115..20510918</td></tr>
<tr><td>0.4</td><td>7421</td><td>3</td><td>scrt sA|Drosophila melanogaster|scrt|FBgn0004880|3L:3956021..3963442</td></tr>
<tr><td>0.4</td><td>4991</td><td>2</td><td>rst F6|Drosophila melanogaster|rst|FBgn0003285|X:2837067..2842058</td></tr>
<tr><td>0.39</td><td>5165</td><td>2</td><td>aop E-lacZ|Drosophila melanogaster|aop|FBgn0000097|2L:2167496..2172661</td></tr>
</table>

<table>
<tr><td>0.38</td><td>10420</td><td>4</td><td>Scr 10.0XX|Drosophila melanogaster|Scr|FBgn0003339|3R:2710985..2721405</td></tr>
<tr><td>0.38</td><td>10419</td><td>4</td><td>pb 10.6+HZR|Drosophila melanogaster|pb|FBgn0051481|3R:2549099..2559518</td></tr>
<tr><td>0.37</td><td>5392</td><td>2</td><td>scrt sC|Drosophila melanogaster|scrt|FBgn0004880|3L:3962922..3968314</td></tr>
<tr><td>0.37</td><td>5380</td><td>2</td><td>elav construct J|Drosophila melanogaster|elav|FBgn0000570|X:374760..380140</td></tr>
<tr><td>0.36</td><td>5509</td><td>2</td><td>Scr 5.6BB|Drosophila melanogaster|Scr|FBgn0003339|3R:2692761..2698270</td></tr>
<tr><td>0.34</td><td>8883</td><td>3</td><td>gcm -11.5/-20|Drosophila melanogaster|gcm|FBgn0014179|2L:9593723..9602606</td></tr>
<tr><td>0.34</td><td>5858</td><td>2</td><td>w B4hsp-W|Drosophila melanogaster|w|FBgn0003996|X:2646754..2652612</td></tr>
<tr><td>0.32</td><td>6280</td><td>2</td><td>EcR DG|Drosophila melanogaster|EcR|FBgn0000546|2R:1679043..1685323</td></tr>
<tr><td>0.3</td><td>6628</td><td>2</td><td>w Bmdel-W|Drosophila melanogaster|w|FBgn0003996|X:2646754..2653382</td></tr>
<tr><td>0.29</td><td>6906</td><td>2</td><td>Ubx abx6.8|Drosophila melanogaster|Ubx|FBgn0003944|3R:12507336..12514242</td></tr>
<tr><td>0.29</td><td>6790</td><td>2</td><td>Scr 7.0RR|Drosophila melanogaster|Scr|FBgn0003339|3R:2699430..2706220</td></tr>
<tr><td>0.28</td><td>7059</td><td>2</td><td>bi pomb29|Drosophila melanogaster|bi|FBgn0000179|X:4283977..4291036</td></tr>
<tr><td>0.26</td><td>7737</td><td>2</td><td>w HPst-W|Drosophila melanogaster|w|FBgn0003996|X:2646754..2654491</td></tr>
<tr><td>0.25</td><td>12002</td><td>3</td><td>dpp BS3.0|Drosophila melanogaster|dpp|FBgn0000490|2L:2475948..2487950</td></tr>
<tr><td>0.21</td><td>9656</td><td>2</td><td>Ser II-9.5|Drosophila melanogaster|Ser|FBgn0004197|3R:23024933..23034589</td></tr>
<tr><td>0.19</td><td>10773</td><td>2</td><td>salm BI|Drosophila melanogaster|salm|FBgn0004579|2L:11399922..11410695</td></tr>
<tr><td>0.19</td><td>10371</td><td>2</td><td>ac 101H10|Drosophila melanogaster|ac|FBgn0000022|X:228981..239352</td></tr>
<tr><td>0.18</td><td>11092</td><td>2</td><td>Pdp1 2nd intron enhancer|Drosophila melanogaster|Pdp1|FBgn0016694|3L:7809872..7820964</td></tr>
</table>
