# Supplementary material for: Correction: Unmasking Activation of the Zygotic Genome Using Chromosomal Deletions in the Drosophila Embryo
Source: PLoS Biol. 2007 Aug 14;5(8):e213. doi: 10.1371/journal.pbio.0050213 (PMC1945081; doi:10.1371/journal.pbio.0050213)
Supplement: Table S3 — (51 KB PDF) [file pbio.0050213.st001.pdf]

Table S3: Secondary Targets Non-Zygotic

affy_probe_set_id	fbgn for the probe set	CG id	Chr.Location	fold-changes	p-value
141445_at	FBgn0024753	CG32593	chrX	-4.59129406	0.000537159
141484_at	FBgn0035588	CG10672	chr3L	-4.178666376	2.21E-11
141648_at	FBgn0023000	CG6936	chr3L	-10.9363276	1.04E-09
141733_at	FBgn0036640	CG4118	chr3L	-3.401839771	8.19E-09
142200_at	FBgn0035115	CR32477	chr3L	-3.807074725	7.90E-09
142393_at	FBgn0039026	CG7029	chr3R	-3.696222738	0.000535828
142855_at	FBgn0031057	CG14224	chrX	-3.233573655	7.25E-06
143038_at	FBgn0037207	CG11100	chr3L	-14.62060191	4.52E-07
143173_at	FBgn0001085	CG17697	chr3L	-3.162060127	5.47E-07
143807_at	FBgn0015402	CG2899	chr3R	-6.678180627	4.86E-07
144186_at	FBgn0027534	CG11006	chr3L	-3.030929389	3.89E-07
144501_at	FBgn0029738	CG4068	chrX	-3.349753136	1.12E-08
144629_f_at	FBgn0029919	NA	chrX	-3.673714667	5.28E-06
144783_at	FBgn0030179	CG12094	chrX	-5.115868648	1.52E-07
145094_at	FBgn0030601	CG32594	chrX	-3.93858081	1.59E-07
147928_at	FBgn0035136	CG6905	chr3L	-3.334423139	4.41E-07
148687_at	FBgn0036330	CG11263	chr3L	-3.353645583	1.19E-06
149033_at	FBgn0036852	CG9739	chr3L	-3.734697017	1.55E-08
149085_at	FBgn0036927	CG7433	chr3L	-3.984199892	2.37E-12
149707_at	FBgn0037948	NA	chr3R	-3.15055305	2.18E-06
149718_at	FBgn0037965	CG31211	chr3R	-5.588849205	2.29E-05
150190_at	FBgn0038699	CG31212	chr3R	-3.426702944	5.16E-09
150501_at	FBgn0039181	CG31132	chr3R	-3.377848771	1.11E-06
150941_f_at	FBgn0039843	NA	chr3R	-3.145375647	1.45E-09
151706_s_at	LD41963.3prime-hit	CG33261	chr3L	-4.194916561	4.44E-10
152798_at	FBgn0036806	CG4120	chr3L	-23.24753064	9.45E-08
153488_at	FBgn0039884	CG31000	chr3R	-3.388570617	3.82E-10
153515_at	FBgn0002849	CG1897	chr3R	-3.13318658	0.000928445
153555_at	FBgn0037205	CG11133	chr3L	-5.530183296	3.38E-11
154659_at	FBgn0015610	CG4236	chr3R	-13.33771132	0
154861_at	FBgn0029094	CG9383	chr3L	-3.762996964	1.60E-10
141293_at	FBgn0037696	CG9362	chr3R	-4.506797576	1.19E-11
142538_at	FBgn0034341	CG17531	chr2R	-21.17718632	3.37E-08
143637_at	FBgn0011206	CG4760	chr3L	-6.14764769	3.52E-09
143889_at	FBgn0017430	CG5994	chr3L	-3.271100778	3.21E-11
148655_at	FBgn0036280	CG32104	chr3L	-3.181366079	5.54E-08
149136_at	FBgn0037009	CG5104	chr3L	-9.884402079	0
150041_at	FBgn0038467	CG3590	chr3R	-3.259106873	1.06E-11
151612_at	LD04071.3prime-hit	CG32149	chr3L	-3.076426227	3.13E-12
152410_at	FBgn0015573	CG1089	chr3R	-19.43757615	0
153182_at	FBgn0038362	CG17604	chr3R	-4.476052656	3.95E-06
153577_at	FBgn0036793	CG4174	chr3L	-12.02276526	6.13E-06
153603_at	FBgn0034360	CG10927	chr2R	-8.879233831	0.000133554
153873_at	FBgn0034813	CG9893	chr2R	-4.683112416	2.32E-10
154519_at	FBgn0037479	CG33546	chr3R	-11.62333776	2.07E-13
155045_at	FBgn0000480	CG33553	chr3R	-3.72799053	1.24E-13
141242_at	FBgn0034388	CG15092	chr2R	-4.730479759	7.42E-05
141678_at	FBgn0030318	CG1697	chrX	-28.39162482	8.01E-12
141706_at	FBgn0033482	CG1371	chr2R	-4.402856905	1.68E-06
142851_at	FBgn0031663	CG8891	chr2L	-3.121773544	4.89E-08
142885_at	FBgn0032799	CG10166	chr2L	-4.013872886	4.62E-08
142988_at	FBgn0032400	CG6770	chr2L	-16.71167983	1.21E-10
143956_at	FBgn0022343	CG3760	chr2R	-6.383200608	2.91E-07

144224_at	FBgn0028516	CG3994	chr2L	-8.368978389	3.63E-10
145022_at	FBgn0030507	CG11164	chrX	-9.413987649	1.07E-08
145146_at	FBgn0030677	CG8198	chrX	-4.950215035	0.000302315
145666_at	FBgn0031459	CG2862	chr2L	-19.81270963	2.23E-14
145759_at	FBgn0031603	CG15432	chr2L	-4.991955195	1.33E-06
145807_at	FBgn0031677	CG14036	chr2L	-18.23978953	8.07E-11
146025_at	FBgn0032027	CG33297	chr2L	-3.596068469	1.62E-06
146374_at	FBgn0032599	CG13277	chr2L	-9.866216734	9.47E-06
146402_at	FBgn0032634	CG6840	chr2L	-3.809897538	1.41E-09
146694_at	FBgn0033105	CG3450	chr2R	-3.82856316	4.04E-08
146878_at	FBgn0033406	CG8799	chr2R	-29.76048345	2.19E-09
146892_at	FBgn0033429	CG12929	chr2R	-5.027240273	2.49E-06
146978_s_at	FBgn0033576	CG30489/CG3	chr2R	-12.15593431	8.60E-08
147104_at	FBgn0033754	CG8816	chr2R	-3.252396957	6.19E-07
147470_at	FBgn0034399	CG15083	chr2R	-3.203866934	2.33E-07
147497_at	FBgn0034443	CG10460	chr2R	-4.544098397	1.56E-07
147810_at	FBgn0034914	CG5554	chr2R	-3.142933766	2.68E-06
151217_at	FBgn0040741	CR32886	chr2R	-893.6536457	6.29E-11
151418_at	FBgn0040954	CG13779	chr2L	-4.212285227	3.03E-07
151659_at	LD21711.3prime-hit	CG5580	chr2R	-12.40889334	1.34E-08
151702_r_at	LD38070.3prime-hit	CG7288	chrX	-5.747387442	5.01E-06
151753_r_at	SD07726.3prime-hit	CG33123	chr2L	-5.833760998	0.000165049
151849_at	FBgn0010288	CG4265	chr2L	-4.031751098	1.02E-05
151851_at	FBgn0027611	CG6206	chr2L	-3.188902913	3.05E-05
151944_at	FBgn0031872	CG9211	chr2L	-3.654260097	3.60E-05
152113_at	FBgn0011746	CG8084	chr2R	-4.564264582	3.42E-05
152301_at	FBgn0033477	CG12918	chr2R	-3.113631167	7.09E-05
152452_at	FBgn0020415	CG4475	chr2L	-34.75596787	6.00E-15
152919_at	FBgn0031228	CG11455	chr2L	-3.156004095	1.65E-07
153158_at	FBgn0031709	CG11024	chr2L	-3.238759405	3.32E-07
153185_at	FBgn0034299	CG5757	chr2R	-6.544573977	3.34E-10
153235_at	FBgn0023184	NA	chr2R	-3.000387911	0.00046829
153318_at	FBgn0027348	CG4501	chr2L	-4.528440156	0.000464257
153359_at	FBgn0030472	CG1633	chrX	-3.549153918	3.16E-07
153430_at	FBgn0033456	CG10536	chr2R	-6.083422065	5.14E-08
153518_at	FBgn0033686	CG12367	chr2R	-4.945400716	0.00069273
153762_at	FBgn0003963	CG2762	chr2L	-14.99850198	5.98E-05
154088_at	FBgn0033381	CG11784	chr2R	-3.089065978	8.82E-07
154260_at	FBgn0025336	CG4882	chr2R	-4.796743128	1.09E-05
154265_at	FBgn0033342	CG8258	chr2R	-3.271282964	7.66E-07
154291_at	FBgn0030550	CG32604	chrX	-3.001432961	4.36E-05
154548_at	FBgn0034098	CG15707	chr2R	-11.61655437	0.000292025
154745_at	FBgn0032752	CG10702	chr2L	-5.396466785	4.22E-09
154865_at	FBgn0033994	CG7544	chr2R	-4.650156873	0.000273737
141267_at	FBgn0031374	CG7337	chr2L	-3.56374114	0.000929064
141286_at	FBgn0035641	CG5568	chr3L	-142.8287011	7.89E-08
141641_at	FBgn0014141	CG3937	chr3R	-3.54891585	1.43E-05
141748_at	FBgn0035927	CG32030	chr3L	-14.31517164	0.000512711
142257_at	FBgn0027552	CG10863	chr3L	-5.357220846	1.54E-07
142537_at	FBgn0034340	CG17530	chr2R	-17.54307319	0.000664082
142612_at	FBgn0037615	CG11760	chr3R	-4.124700573	2.92E-07
142838_at	FBgn0000166	CG1034	chrU	-13.50843597	4.44E-06
142862_at	FBgn0032215	CG5385	chr2L	-3.118417864	2.55E-05
143268_at	FBgn0002778	CG3297	chr3L	-3.464398581	0.00027825
143307_at	FBgn0003114	CG9183	chr2R	-3.582706712	0.000586968
143333_at	FBgn0003326	CG17579	chr2R	-6.118143082	2.51E-07

143830_at	FBgn0015614	CG11217	chr2R	-6.931478152	0.000199827
144023_at	FBgn0024732	CG8357	chr2R	-19.14757091	6.43E-05
144026_at	FBgn0024841	CG1963	chr3R	-3.181388144	6.27E-05
144169_at	FBgn0027052	CG5203	chr2L	-3.392315262	3.58E-05
144183_at	FBgn0027506	CG6542	chr2R	-3.501415305	0.000363732
144335_at	FBgn0028967	CG8947	chr3L	-12.9299909	8.60E-11
145586_at	FBgn0031346	CG7428	chr2L	-3.071170407	6.87E-06
145612_at	FBgn0031381	CG7291	chr2L	-3.250942931	3.61E-07
146305_at	FBgn0032463	CG3762	chr2L	-3.027348011	3.95E-05
146644_at	FBgn0033026	CG30440	chr2R	-14.02438487	0.000101792
146963_at	FBgn0033548	CG7637	chr2R	-3.761242577	2.67E-07
147260_at	FBgn0034039	CG30080	chr2R	-3.839383524	2.94E-05
148690_at	FBgn0036335	CG11258	chr3L	-7.37020317	8.38E-08
150326_at	FBgn0038903	CG13418	chr3R	-3.544041603	2.49E-05
150561_at	FBgn0039291	CG13663	chr3R	-3.08793081	0.000118929
150748_at	FBgn0039544	CG12877	chr3R	-3.563414446	0.000185387
151445_at	FBgn0040985	CG6115	chr2L	-3.438202393	7.70E-05
151823_at	FBgn0034450	CG8201	chr2R	-4.073441697	2.11E-05
152174_at	FBgn0035195	CG9128	chr3L	-3.833632239	7.68E-06
152480_at	FBgn0034028	CG8186	chr2R	-5.021343269	1.58E-08
152504_at	FBgn0014869	CG1721	chr3R	-4.378765042	3.14E-07
152523_at	FBgn0032250	CG5198	chr2L	-3.072338917	4.08E-05
153187_at	FBgn0034365	CG5335	chr2R	-4.616240052	4.39E-06
153193_at	FBgn0037617	CG8145	chr3R	-3.088312325	1.16E-05
153307_at	FBgn0001226	CG4466	chr3L	-3.661928325	3.28E-06
153457_at	FBgn0037844	CG4570	chr3R	-3.868101169	5.49E-07
153469_at	FBgn0032193	CG5727	chr2L	-5.033547637	3.45E-06
153500_at	FBgn0037655	CG11984	chr3R	-4.991188993	4.20E-06
153522_at	FBgn0000384	CG17678	chr2h	-3.358057996	0.000202543
153524_at	FBgn0015788	CG8024	chr2R	-6.244145906	5.78E-09
153715_at	FBgn0036194	CG11652	chr3L	-5.344212251	4.48E-08
153802_at	FBgn0039132	CG5864	chr3R	-3.22372426	3.33E-05
153947_at	FBgn0002673	CG4965	chr2L	-6.062752554	0.000303336
154067_at	FBgn0038252	CG3509	chr3R	-17.30285528	4.55E-10
154116_at	FBgn0025803	CG17299	chr3R	-3.60465136	1.82E-06
154129_at	FBgn0011705	CG9552	chr2L	-3.999954566	0.000141989
154262_at	FBgn0004507	CG7254	chr2L	-7.300365647	5.36E-09
154312_at	FBgn0003015	CG10901	chr3R	-7.411459061	1.14E-05
154347_at	FBgn0036863	CG9670	chr3L	-4.512295796	0.000108443
154391_at	FBgn0033635	CG7777	chr2R	-20.43080718	0.000118677
154408_at	FBgn0028528	CG4482	chr2L	-5.074131294	7.90E-06
154516_at	FBgn0016070	CG5263	chr3L	-4.69612965	1.83E-06
154534_at	FBgn0038251	CG3508	chr3R	-4.248716096	2.59E-05
154559_at	FBgn0036689	CG7730	chr3L	-4.017298967	7.83E-05
154699_at	FBgn0001078	CG4059	chr3L	-4.469718922	0.000424835
154856_at	FBgn0033236	CG14764	chr2R	-4.279234737	1.14E-05
154885_at	FBgn0028743	CG5036	chr2R	-3.361356095	2.56E-06
154971_at	FBgn0031320	CG5126	chr2L	-4.119281479	5.07E-06
154972_at	FBgn0037664	CG8420	chr3R	-5.567138761	7.81E-08
155019_at	FBgn0038410	CG12785	chr3R	-3.836069186	4.57E-06
155134_at	FBgn0036333	CG11259	chr3L	-10.11745156	0.000336823
155142_at	FBgn0032262	CG7384	chr2L	-4.262569854	7.19E-05
141220_at	FBgn0039831	CG12054	chr3R	3.327230211	5.53E-08
141264_at	FBgn0003525	CG1395	chr3R	3.061975849	3.42E-07
141298_at	FBgn0035206	CG9186	chr3L	4.303263211	4.72E-12
141387_at	FBgn0035473	CG14981	chr3L	3.757776318	7.09E-11

141483_at	FBgn0038661	CG17836	chr3R	12.97909451	1.20E-11
141629_at	FBgn0015218	CG4035	chr3L	3.663410328	1.12E-11
141664_at	FBgn0015245	CG12101	chrX	3.520258281	2.54E-07
141691_at	FBgn0003495	CG6134	chr3R	5.014171685	1.28E-07
142265_at	FBgn0036712	CG16793	chr3L	15.28591112	6.98E-09
142266_at	FBgn0035836	CG7919	chr3L	10.16625441	1.30E-07
142492_at	FBgn0013953	CG7005	chr3R	11.51402543	4.44E-16
142503_at	FBgn0026429	CG10988	chrX	4.080854895	9.24E-09
142569_at	FBgn0003423	CG1417	chrX	6.885982556	1.48E-06
142580_at	FBgn0036453	CG5272	chr3L	4.121108374	3.68E-07
142610_at	FBgn0038828	CG17270	chr3R	6.832457146	5.40E-12
142661_at	FBgn0027516	CG18617	chr3R	3.014733183	4.94E-06
142799_at	FBgn0036008	CG3408	chr3L	11.86618813	1.19E-08
142971_at	FBgn0003638	CG3019	chrX	17.11312225	2.97E-09
143055_at	FBgn0000022	CG3796	chrX	31.20603628	1.12E-11
143197_at	FBgn0001230	CG5436	chr3R	3.603363968	0.000144511
143230_at	FBgn0002522	CG1264	chr3R	9.702252135	2.20E-06
143411_at	FBgn0003997	CG5123	chr3L	4.461444803	1.49E-06
143429_at	FBgn0004143	CG14426	chrX	23.68589658	0
143431_at	FBgn0004170	CG3827	chrX	32.25566221	0
143680_at	FBgn0011706	CG4319	chr3L	29.01013548	2.22E-16
143839_at	FBgn0015765	CG5475	chr3R	10.33285724	2.70E-08
143856_at	FBgn0015919	CG10605	chr3L	6.517072514	2.82E-06
144097_at	FBgn0025814	CG1742	chrX	3.761995754	3.51E-12
144115_at	FBgn0026084	CG4944	chrX	8.300013692	6.99E-10
144398_at	FBgn0029610	CG14050	chrX	3.526225391	0.000381276
144766_at	FBgn0030151	CG1354	chrX	6.292096361	1.78E-15
144911_at	FBgn0030363	CG15736	chrX	6.470506148	0
144927_s_at	FBgn0030380	NA	chrU	18.32834985	3.12E-11
145183_at	FBgn0030743	CG9921	chrX	4.657942658	8.19E-09
145309_at	FBgn0030934	CG6335	chrX	3.059692851	5.81E-09
145366_at	FBgn0031016	CG12199	chrX	4.455674812	1.17E-08
145376_at	FBgn0031032	CG14204	chrX	3.509333561	1.67E-09
145393_at	FBgn0031067	CG32528	chrX	4.915506767	6.63E-08
145466_at	FBgn0031165	CG32521	chrX	5.24124013	8.69E-06
146619_i_at	FBgn0032985	CG1742/CR12	chrX	5.379780714	2.83E-13
146620_s_at	FBgn0032985	CG1742/CR12	chrX	3.813973794	3.09E-14
147972_at	FBgn0035211	CG2211	chr3L	11.72923372	1.18E-08
148191_at	FBgn0035563	CG13716	chr3L	101.1263318	0
148412_at	FBgn0035907	CG6662	chr3L	24.21729907	1.23E-11
148468_at	FBgn0036003	CG14180	chr3L	10.24606668	7.30E-11
148567_at	FBgn0036150	CG6185	chr3L	3.789268561	1.55E-07
148591_at	FBgn0036188	CG7339	chr3L	4.006966952	9.20E-09
148694_at	FBgn0036341	CG11278	chr3L	3.355450048	9.04E-08
148775_at	FBgn0036461	CG10006	chr3L	3.532414865	0.000374089
148908_at	FBgn0036652	CG13032	chr3L	5.847769278	6.34E-06
148915_at	FBgn0036664	CG9710	chr3L	10.38736441	1.11E-15
149095_at	FBgn0036942	CG7328	chr3L	3.028745475	2.35E-07
149305_at	FBgn0037290	CG1124	chr3R	44.81972432	4.44E-16
149571_at	FBgn0037722	CG8319	chr3R	3.195428175	1.42E-07
149762_at	FBgn0038027	CG4421	chr3R	3.960268188	8.49E-08
149953_at	FBgn0038331	CG5073	chr3R	4.143223104	1.75E-08
150121_at	FBgn0038587	CG7998	chr3R	3.089787289	5.12E-07
150539_at	FBgn0039251	CG17462	chr3R	3.386799245	9.16E-08
150776_at	FBgn0039585	CG1894	chr3R	39.25184952	1.55E-11
150831_at	FBgn0039678	CG18111	chr3R	3.936584656	1.74E-10

150862_at	FBgn0039729	CG7899	chr3R	12.74794202	2.53E-08
151522_at	GH13437.3prime-hit	CG5889	chr3R	3.34915928	0.000297049
151569_at	GH28553.3prime-hit	CG11848	chr3R	14.47763686	8.56E-14
151635_s_at	LD12394.3prime-hit	CG6235	chr3R	3.747722133	2.17E-09
151664_at	LD25651.3prime-hit	CG2522	chrX	5.010215274	2.94E-08
151670_at	LD27553.3prime-hit	CG2678	chr3R	4.61977604	1.66E-10
151795_at	FBgn0035881	CG7176	chr3L	3.955608212	4.22E-08
151799_at	FBgn0026252	CG7935	chr3L	4.294001005	5.88E-12
151885_at	FBgn0027601	CG9009	chrX	4.104391409	0.000230819
152119_at	FBgn0027573	CG5518	chr3R	28.19822668	7.97E-09
152120_at	FBgn0038194	CG3050	chr3R	17.48122243	0
152336_at	FBgn0038563	CG7780	chr3R	6.472735648	1.45E-11
152481_at	FBgn0036671	CG9951	chr3L	3.005860059	1.07E-05
152492_at	FBgn0035937	CG5741	chr3L	4.69173307	5.53E-06
152753_at	FBgn0027103	CG6159	chr3R	3.036767945	3.11E-06
153031_at	FBgn0038037	CG11466	chr3R	3.151672842	8.98E-05
153126_at	FBgn0037156	CG11523	chr3L	3.494484854	3.45E-11
153166_at	FBgn0037843	CG4511	chr3R	3.786691585	7.61E-11
153169_at	FBgn0030740	CG9917	chrX	3.677562852	3.20E-08
153174_at	FBgn0038191	CG9925	chr3R	3.518848902	2.22E-07
153199_at	FBgn0000242	CG6500	chrX	24.5739192	8.58E-08
153396_at	FBgn0023023	CG1411	chr3R	7.057848927	5.53E-10
153402_at	FBgn0030345	CG1847	chrX	3.517959242	1.41E-08
153443_at	FBgn0038925	CG6022	chr3R	3.452625218	8.11E-10
153450_at	FBgn0030724	CG9212	chrX	3.219105645	8.66E-07
153523_at	FBgn0011826	CG9842	chrX	3.883311506	6.89E-07
153616_at	FBgn0039304	CG10425	chr3R	4.39138653	5.01E-09
153685_at	FBgn0035181	CG9205	chr3L	3.138341176	1.14E-09
153688_at	FBgn0038721	CG16718	chr3R	4.627961441	0.000455559
153694_at	FBgn0036650	CG3971	chr3L	4.047616056	7.76E-06
153765_at	FBgn0038141	CG9829	chr3R	3.21191261	7.57E-09
153769_at	FBgn0012034	CG9390	chr3L	4.953290085	2.95E-08
153777_at	FBgn0037513	CG3027	chr3R	5.244746034	7.30E-11
153782_at	FBgn0035978	CG4347	chr3L	4.564649302	2.05E-11
153941_at	FBgn0037724	CG9434	chr3R	4.763037864	2.92E-06
153959_at	FBgn0016034	CG11254	chr3L	4.824089509	5.97E-14
154175_at	FBgn0038463	CG3534	chr3R	3.252691188	1.74E-08
154191_at	FBgn0039335	CG5127	chr3R	3.104291362	2.07E-06
154245_at	FBgn0030551	CG11674	chrX	4.159223965	3.62E-11
154381_at	FBgn0036357	CG10724	chr3L	4.214355082	2.00E-11
154458_at	FBgn0026083	CG4857	chrX	3.27375146	1.51E-06
154465_at	FBgn0038268	CG3631	chr3R	3.337171929	2.49E-05
154496_at	FBgn0036332	CG11261	chr3L	154.0587298	2.22E-16
154521_at	FBgn0004598	CG18734	chrX	3.491224935	5.74E-05
154531_at	FBgn0036887	CG9231	chr3L	3.12083878	1.66E-09
154553_at	FBgn0038296	CG6752	chr3R	5.699476479	8.07E-09
154652_at	FBgn0015568	CG1031	chr3R	6.704478345	1.79E-11
154763_at	FBgn0036572	CG5165	chr3L	3.437617606	1.00E-07
154796_at	FBgn0037203	CG11128	chr3L	3.09908788	4.99E-06
154828_at	FBgn0039018	CG4771	chr3R	3.201423182	1.38E-07
154883_at	FBgn0035848	CG7979	chr3L	6.035203775	9.98E-10
154888_at	FBgn0029924	CG4586	chrX	10.68618505	1.33E-09
154989_at	FBgn0035151	CG17129	chr3L	5.091176669	1.90E-11
155055_at	FBgn0037353	CG31549	chr3R	23.79431452	0
155139_at	FBgn0036837	CG18135	chr3L	3.761627119	5.51E-07
155145_at	FBgn0029889	CG4094	chrX	6.943187881	9.66E-15

155161_at	FBgn0003512	CG17958	chr3R	3.869189756	4.47E-13
141216_at	FBgn0030457	CG12096	chrX	4.092680305	2.23E-09
141245_at	FBgn0014861	CG7538	chr3R	3.316754466	8.17E-12
141361_at	FBgn0039637	CG11880	chr3R	3.008030792	7.30E-08
141498_at	FBgn0024250	CG9653	chrX	3.09330454	1.51E-12
141522_at	FBgn0033311	CG8643	chr2R	6.391492918	7.43E-08
141707_at	FBgn0011570	CG17158	chr2L	28.96653072	0
141811_at	FBgn0035226	CG1009	chr3L	4.46441801	2.44E-15
142210_at	FBgn0022740	CG5005	chr2R	9.485350296	0
142306_at	FBgn0035152	CG3386	chr3L	3.911706579	2.42E-11
142372_at	FBgn0033971	CG10209	chr2R	4.027164065	3.25E-14
142510_at	FBgn0033847	CG6050	chr2R	3.114914073	6.86E-13
142634_at	FBgn0034087	CG8443	chr2R	3.50521446	1.04E-07
143105_at	FBgn0000346	CG1618	chrX	3.265042844	6.74E-06
143145_at	FBgn0000562	CG4051	chr2R	5.392699023	1.82E-08
143150_at	FBgn0000579	CG17654	chr2L	5.21318578	2.55E-15
143253_at	FBgn0002632	CG8354	chr3R	6.859702287	4.81E-08
143288_at	FBgn0003023	CG12743	chrX	4.154580476	6.37E-09
143742_at	FBgn0014010	CG3664	chr2L	7.274079908	0
143867_at	FBgn0016059	CG6446	chr2R	4.113036683	3.55E-12
144149_at	FBgn0026602	CG1851	chr2R	37.23516079	4.44E-15
144337_at	FBgn0029504	CG12690	chrX	3.143803666	6.64E-07
144476_at	FBgn0029706	CG3626	chrX	14.73074557	3.01E-11
144792_at	FBgn0030191	CG15306	chrX	6.601613729	8.24E-08
145400_f_at	FBgn0031073	CG11941	chrX	11.0553901	1.11E-15
145489_at	FBgn0031194	CG17598	chrX	13.45401744	5.23E-12
145592_at	FBgn0031352	CG7361	chr2L	5.569276822	0
145595_at	FBgn0031357	CG17642	chr2L	11.53996129	0
145637_at	FBgn0031418	CG3609	chr2L	3.879415015	9.99E-16
145847_at	FBgn0031741	CG11034	chr2L	3.539944007	5.88E-10
145955_at	FBgn0031912	CG5261	chr2L	3.082278382	3.87E-10
146001_at	FBgn0031974	CG12560	chr2L	8.699217089	1.64E-11
146648_at	FBgn0033031	CG8245	chr2R	3.216366659	0.000707051
146947_at	FBgn0033519	CG11825	chr2R	8.889856189	0
147059_s_at	FBgn0033689	NA	chr2R	3.487232072	4.75E-08
147302_at	FBgn0034113	CG8060	chr2R	5.479820347	8.40E-06
147501_at	FBgn0034452	CG11237	chr2R	4.023739733	8.51E-08
147513_at	FBgn0034467	CG15128	chr2R	5.971761001	1.42E-11
147514_at	FBgn0034468	CG11797	chr2R	5.917411032	6.66E-16
147516_at	FBgn0034470	CG11218	chr2R	9.739545782	3.01E-14
148284_at	FBgn0035706	CG10125	chr3L	3.272470496	2.81E-06
151138_at	FBgn0040654	CG33152	chr2R	3.199582598	5.51E-10
151192_at	FBgn0040715	CG15386	chr2L	4.861100969	8.67E-09
151308_at	FBgn0040835	CG32365	chr3L	5.934536265	9.10E-06
151501_at	GH08307.3prime-hit	CG12052	chr2R	5.063255826	7.03E-06
151649_at	LD18295.3prime-hit	CG32037	chr3L	4.524005148	7.11E-10
151655_at	LD19312.3prime-hit	CG8443	chr2R	3.55351709	5.76E-06
151763_at	FBgn0033484	CG2269	chr2R	3.955907515	3.88E-08
151924_at	FBgn0034408	CG12758	chr2R	3.578222716	2.30E-09
152194_at	FBgn0010240	CG17336	chrX	3.48516183	0.00067572
152222_at	FBgn0027558	CG4445	chr2R	3.001265597	6.90E-09
152231_at	FBgn0034930	CG5594	chr2R	3.040579204	5.58E-05
152370_at	FBgn0034724	CG3624	chr2R	5.204762437	1.24E-07
152520_at	FBgn0034094	CG3666	chr2R	6.648749543	8.54E-06
152829_at	FBgn0035456	CG32264	chr3L	4.717800992	1.61E-09
152948_at	FBgn0033309	CG8735	chr2R	3.901473872	5.21E-07

153233_at	FBgn0033875	CG6357	chr2R	6.842264879	2.62E-06
153270_at	FBgn0039496	CG31063	chr3R	4.863470026	1.11E-16
153300_at	FBgn0030048	CG12112	chrX	4.646766553	3.58E-12
153339_at	FBgn0039737	CG7920	chr3R	5.168324417	3.33E-16
153498_at	FBgn0027897	CG6773	chr3R	4.522866614	0
153631_at	FBgn0030834	CG8675	chrX	3.480208862	2.43E-09
153804_at	FBgn0039638	CG11881	chr3R	3.296271971	6.88E-12
154024_at	FBgn0015268	CG5330	chr2R	10.15763986	0
154070_at	FBgn0024489	CG7001	chrX	4.48855552	2.19E-14
154078_at	FBgn0014469	CG2060	chr2R	3.020054154	1.32E-10
154112_at	FBgn0038220	CG12207	chr3R	3.616733912	1.87E-12
154176_at	FBgn0034934	CG2827	chr2R	5.656625217	5.82E-13
154244_at	FBgn0001091	CG12055	chr2R	4.542606392	2.11E-15
154326_at	FBgn0032714	CG17322	chr2L	8.761643527	5.77E-15
154484_at	FBgn0031391	CG11723	chr2L	3.526366832	2.06E-06
154613_at	FBgn0031360	CG31937	chr2L	3.755413915	2.22E-16
155162_at	FBgn0034395	CG15081	chr2R	6.336257454	0
141308_at	FBgn0031224	CG11454	chr2L	3.413844671	2.25E-06
141489_at	FBgn0034599	CG9437	chr2R	3.571487484	2.01E-05
141491_at	FBgn0030845	CG8465	chrX	3.687825534	1.41E-08
141558_at	FBgn0023506	CG1474	chrX	4.372358556	5.11E-09
141588_at	FBgn0003742	CG10128	chr2R	3.914469017	2.57E-06
141607_at	FBgn0024988	CG14801	chrX	5.370686736	2.11E-10
142125_at	FBgn0001404	CG9659	chrX	3.277656608	0.000670113
142234_at	FBgn0004581	CG17611	chr2R	8.084562556	3.62E-09
142240_at	FBgn0032011	CG8049	chr2L	12.53207205	0.000221023
142444_at	FBgn0029980	CG10778	chrX	3.13029429	6.50E-07
142484_at	FBgn0011571	CG3606	chrX	17.94647784	5.53E-08
142524_at	FBgn0030330	CG1841	chrX	4.379940899	3.74E-07
142590_at	FBgn0030703	CG9066	chrX	3.023113664	3.45E-07
142687_at	FBgn0030087	CG7766	chrX	4.724270967	1.46E-06
142714_at	FBgn0032172	CG5850	chr2L	4.792244126	1.72E-06
142843_at	FBgn0034371	CG5473	chr2R	4.477364797	2.07E-06
142856_at	FBgn0031895	CG4497	chr2L	3.680339603	6.55E-08
142917_at	FBgn0025394	CG32810	chrX	3.526185816	9.19E-07
142930_at	FBgn0033584	CG7737	chr2R	3.007286179	2.68E-05
143037_at	FBgn0034015	CG8174	chr2R	3.694452904	1.64E-05
143091_at	FBgn0000233	CG12653	chrX	3.19352006	5.25E-05
143210_at	FBgn0001330	CG3228	chrX	4.281057286	3.44E-06
143502_at	FBgn0004647	CG3936	chrX	3.123366213	0.000103363
143556_at	FBgn0005617	CG10385	chr2L	3.737589705	4.48E-07
143685_at	FBgn0011742	CG9901	chrX	9.244017386	3.76E-06
143693_at	FBgn0011818	CG9884	chr2L	6.857489216	2.49E-08
143696_at	FBgn0011824	CG4038	chr2R	3.455913007	5.29E-06
144031_at	FBgn0024920	CG3181	chr2L	3.574694193	2.36E-08
144080_at	FBgn0025639	CG13363	chrX	4.511388579	1.73E-06
144157_s_at	FBgn0026758	CG18009	chrX	3.228182657	1.13E-06
144162_at	FBgn0026871	CG14781	chrX	4.943075817	5.59E-08
144202_at	FBgn0028397	CG9214	chrX	4.35616992	2.13E-06
144217_at	FBgn0028485	CG4272	chr2L	4.507365238	1.06E-08
144459_at	FBgn0029686	CG2941	chrX	4.956205148	3.65E-07
144547_at	FBgn0029798	CG4078	chrX	3.230549366	2.51E-06
144584_at	FBgn0029852	CG3576	chrX	3.681967534	8.32E-08
144596_at	FBgn0029869	CG3861	chrX	5.932261818	8.02E-10
144625_at	FBgn0029915	CG14434	chrX	5.480506229	1.04E-08
144626_at	FBgn0029916	CG32737	chrX	3.750599436	5.84E-08

144641_at	FBgn0029933	CG33070	chrX	11.18781396	8.69E-10
144645_at	FBgn0029944	CG2079	chrX	16.01825074	0.000164308
144706_at	FBgn0030046	CG33181	chrX	5.090780025	2.37E-06
144716_at	FBgn0030067	CG10648	chrX	14.01548613	3.35E-09
144726_at	FBgn0030081	CG7246	chrX	5.592070533	1.38E-07
144729_at	FBgn0030089	CG9113	chrX	3.075650734	1.22E-06
144764_at	FBgn0030147	CG32697	chrX	15.53575337	3.47E-06
144850_at	FBgn0030268	CG1453	chrX	3.683036324	1.34E-06
144938_at	FBgn0030391	CG1900	chrX	3.086307096	2.96E-05
144950_at	FBgn0030405	CG32654	chrX	8.040657134	1.64E-06
144993_at	FBgn0030460	CG2453	chrX	3.932060901	7.63E-06
145020_at	FBgn0030502	CG12175	chrX	5.988345941	0.000183571
145098_at	FBgn0030608	CG9057	chrX	5.707832828	1.32E-06
145152_at	FBgn0030687	CG17209	chrX	15.43414026	0.000118144
145326_s_at	FBgn0030956	CG6961/CG18	chrX	4.324900892	4.37E-09
145330_at	FBgn0030964	CG7103	chrX	18.83255127	2.30E-07
145397_at	FBgn0031071	CG12701	chrX	17.5449964	4.67E-05
145473_at	FBgn0031173	CG1696	chrX	4.346243711	6.66E-08
145582_at	FBgn0031339	CG5481	chr2L	4.489489915	2.19E-06
145583_at	FBgn0031340	CG5481	chr2L	4.149073372	7.01E-06
145621_at	FBgn0031393	CG15382	chr2L	6.760853137	1.20E-11
145685_at	FBgn0031491	CG17223	chr2L	3.307017036	3.93E-07
145846_at	FBgn0031736	CG11030	chr2L	3.207737521	2.47E-05
145888_at	FBgn0031798	CG9491	chr2L	5.27996411	5.46E-08
145889_at	FBgn0031799	CG9493	chr2L	5.874696433	2.23E-08
145910_at	FBgn0031832	CG9596	chr2L	3.290982458	3.74E-06
146035_at	FBgn0032044	CG9233	chr2L	6.478446569	5.45E-08
146041_at	FBgn0032056	CG18042	chr2L	3.059736198	2.70E-05
146113_at	FBgn0032168	CG13126	chr2L	3.102751682	3.25E-06
146198_at	FBgn0032305	CG6700	chr2L	3.698850572	1.01E-06
146212_at	FBgn0032320	CG33129	chr2L	5.572830612	2.18E-05
146253_at	FBgn0032378	CG14939	chr2L	4.114912554	2.36E-06
146417_at	FBgn0032656	CG5674	chr2L	3.131009935	1.23E-06
146446_at	FBgn0032702	CG10376	chr2L	3.779937551	8.84E-07
146565_at	FBgn0032907	CG9272	chr2L	4.63372946	5.24E-06
146576_at	FBgn0032928	CG9242	chr2L	4.182987397	1.15E-08
146613_at	FBgn0032979	CG1832	chr2L	7.778080275	3.49E-12
146649_at	FBgn0033032	CG1298	chr2R	3.248203516	4.01E-08
146698_at	FBgn0033109	CG9446	chr2R	5.85506647	3.10E-09
146733_at	FBgn0033163	CG11166	chr2R	3.084638083	1.31E-05
146764_at	FBgn0033217	CG18812	chr2R	5.074193194	9.96E-06
146778_at	FBgn0033239	CG14764	chr2R	3.369449115	5.88E-06
146787_at	FBgn0033258	CG8712	chr2R	3.323580506	0.000344107
146882_at	FBgn0033414	CG11804	chr2R	5.971541712	1.89E-06
147107_at	FBgn0033757	CG8811	chr2R	3.969619706	8.64E-08
147110_at	FBgn0033762	CG8632	chr2R	14.12495841	0.000213271
147154_at	FBgn0033841	CG5912	chr2R	3.959970824	0.00018877
147195_at	FBgn0033926	CG12505	chr2R	6.200508302	0.000222338
147254_at	FBgn0034025	CG8182	chr2R	8.208015346	1.40E-06
147282_at	FBgn0034078	NA	chr2R	16.14477993	0.000126804
147364_at	FBgn0034222	CG14478	chr2R	5.655576984	2.08E-09
147382_at	FBgn0034252	CG4943	chr2R	3.431122046	2.69E-06
147419_at	FBgn0034313	CG5726	chr2R	6.500731727	2.82E-07
147452_at	FBgn0034370	CG30122	chr2R	11.2925187	0.000801316
147593_at	FBgn0034573	CG3295	chr2R	3.4553625	2.06E-05
147607_at	FBgn0034603	CG9480	chr2R	4.242334314	3.55E-06

148646_at	FBgn0036270	CG17332	chr2L	3.414320338	2.31E-08
151361_at	FBgn0040888	CG7481	chrX	4.481583324	9.36E-06
151387_at	FBgn0040918	CG3576	chrX	3.506620978	0.000125334
151442_at	FBgn0040981	CG4482	chr2L	4.899643659	1.27E-08
151498_at	GH07910.3prime-hit	CG32782	chrX	3.407763829	0.000106629
151708_s_at	LD43003.3prime-hit	CG1745	chrX	5.319333257	1.35E-07
151920_at	FBgn0032223	CG5034	chr2L	5.135073521	4.14E-07
152340_at	FBgn0004837	CG3497	chr2L	4.232711179	4.76E-06
152361_at	FBgn0030555	CG1839	chrX	5.617172561	0.000418247
152396_at	FBgn0031995	CG8475	chr2L	3.244069017	2.42E-05
152474_at	FBgn0034722	CG10955	chr2R	7.040426974	8.08E-10
152478_at	FBgn0035087	CG2765	chr2R	3.344005139	6.96E-05
152583_at	FBgn0019650	CG11186	chr4	5.619011226	2.26E-07
152715_at	FBgn0033188	CG1600	chr2R	3.717167452	1.60E-05
152884_at	FBgn0033812	CG4663	chr2R	3.620930316	9.31E-06
152922_at	FBgn0021748	CG6775	chrX	4.775091351	0.000148723
153013_at	FBgn0028916	CG33090	chr2L	3.874960658	1.78E-05
153026_at	FBgn0033383	CG8057	chr2R	6.178217587	2.23E-08
153130_at	FBgn0030744	CG9992	chrX	4.712663276	4.23E-07
153269_at	FBgn0030474	CG15747	chrX	4.506861583	5.12E-06
153459_at	FBgn0026869	CG1981	chr4	4.532094143	1.59E-06
153484_at	FBgn0030838	CG5445	chrX	3.669465352	4.69E-08
153511_at	FBgn0031972	CG7221	chr2L	6.218403049	1.17E-07
153665_at	FBgn0017558	NA	chr2R	3.964785676	8.90E-09
153669_at	FBgn0031871	CG10158	chr2L	4.015973977	3.60E-08
153740_at	FBgn0030735	CG3632	chrX	3.013395875	1.77E-07
153812_at	FBgn0010292	CG1430	chrX	4.336957331	1.41E-09
153897_at	FBgn0000183	CG6605	chr2L	7.476207766	5.93E-08
153908_at	FBgn0024984	CG3457	chrX	3.933464829	9.19E-09
153955_at	FBgn0025381	CG14782	chrX	3.745730787	8.02E-08
153979_at	FBgn0033402	CG2078	chr2R	3.724977214	9.89E-06
154001_at	FBgn0030607	CG5560	chrX	6.228162711	0.000526731
154035_at	FBgn0033845	CG17064	chr2R	4.344935293	8.93E-08
154069_at	FBgn0031090	CG9575	chrX	3.893402714	5.51E-08
154113_at	FBgn0031947	CG7154	chr2L	3.182037164	1.83E-07
154155_at	FBgn0033082	CG3273	chr2R	4.193502648	4.34E-08
154253_at	FBgn0003031	CG5119	chr2R	4.693408417	2.38E-07
154297_at	FBgn0032467	CG9934	chr2L	3.183935522	1.10E-07
154305_at	FBgn0034962	CG3167	chr2R	3.740740744	9.53E-09
154311_at	FBgn0034432	CG7461	chr2R	6.56408051	4.96E-10
154368_at	FBgn0030135	CG32701	chrX	3.828662737	8.95E-07
154522_at	FBgn0031018	CG12200	chrX	429.3072434	4.10E-09
154586_at	FBgn0027842	CG12891	chr2R	3.290587127	7.12E-08
154592_at	FBgn0030809	CG9086	chrX	3.4961354	1.10E-06
154761_at	FBgn0032956	CG1512	chr2L	3.884849989	2.02E-05
154842_at	FBgn0034569	CG3221	chr2R	3.844995188	8.20E-08
154846_at	FBgn0034657	CG17952	chr2R	3.894553141	3.79E-08
154931_at	FBgn0033581	CG12391	chr2R	3.959952679	1.22E-07
154973_at	FBgn0033185	CG1603	chr2R	3.898018481	3.66E-09
154988_at	FBgn0023216	CG2864	chrX	23.27658445	7.67E-05
141392_at	FBgn0038881	CG16791	chr3R	3.090957194	4.90E-05
141627_at	FBgn0037141	CG7143	chr3L	3.860058311	0.000120304
141660_at	FBgn0036844	CG14080	chr3L	15.54472947	7.31E-08
141750_at	FBgn0039072	CG6768	chr3R	3.587339219	0.000271029
142045_at	LD36125.3prime-hit	NA	chr2L	4.533777997	0.000110682
142630_at	FBgn0036400	CG32133	chr3L	26.16802063	1.41E-08

142834_at	FBgn0024958	CG4900	chr3R	7.297048504	2.56E-08
143004_at	FBgn0039174	CG33106	chr3R	3.080761055	0.000225611
143444_at	FBgn0004359	CG5507	chr3R	4.0248895	1.02E-05
143458_at	FBgn0004395	CG4620	chr3R	3.115548837	0.000502965
143635_at	FBgn0010808	CG1715	chr3R	4.620996188	5.11E-08
143845_at	FBgn0015797	CG6601	chr2L	4.789704945	2.15E-05
143885_at	FBgn0017397	CG10293	chr3R	3.612899729	2.38E-07
144102_at	FBgn0025832	CG8648	chr2R	3.107866216	0.000226568
144113_at	FBgn0026064	CG6715	chr3R	9.811857291	2.87E-08
145584_at	FBgn0031341	CG5481	chr2L	3.14354989	4.13E-05
147102_at	FBgn0033750	CG13151	chr2R	4.417696709	1.06E-06
147309_at	FBgn0034126	CG4398	chr2R	3.091737269	0.000535185
147384_at	FBgn0034254	CG10683	chr2R	5.081266986	1.77E-06
147553_at	FBgn0034520	CG13424	chr2R	12.62654622	4.70E-06
147679_at	FBgn0034733	CG4752	chr2R	6.315127275	8.73E-06
147836_at	FBgn0034969	NA	chr2R	3.667554257	0.000262393
148125_at	FBgn0035465	CG14975	chr3L	3.995238978	1.47E-06
148630_at	FBgn0036245	CG5661	chr3L	3.906598383	0.000554836
148726_at	FBgn0036382	CG13737	chr3L	143.9872466	1.29E-12
148929_at	FBgn0036693	CG33522	chr3L	5.255317415	1.04E-05
149221_at	FBgn0037150	CG7133	chr3L	3.512467415	0.000886048
149311_at	FBgn0037297	CG1116	chr3R	3.181115373	6.69E-05
149755_at	FBgn0038020	CG10091	chr3R	62.37820478	6.49E-12
149759_at	FBgn0038024	CG12242	chr3R	5.113045882	5.72E-06
149867_at	FBgn0038193	CG12537	chr3R	7.118043473	3.54E-07
150069_at	FBgn0038504	CG5407	chr3R	5.098611172	7.60E-06
150095_at	FBgn0038540	CG14321	chr3R	3.190504311	9.25E-06
150346_at	FBgn0038934	CG5732	chr3R	5.073674341	1.83E-05
150844_at	FBgn0039695	CG12068	chr3R	4.218615991	2.34E-06
151023_at	FBgn0040536	CG12418	chr3R	5.461177323	5.24E-05
151554_at	GH23001.3prime-hit	NA	chr3R	92.83649149	5.39E-11
151575_at	GM03596.3prime-hit	NA	chr3L	8.69750315	4.47E-08
151576_s_at	GM04312.3prime-hit	CG17964	chr4	3.324435648	5.30E-06
151632_i_at	LD12042.3prime-hit	CG3307	chr3R	15.68067544	5.81E-11
151633_r_at	LD12042.3prime-hit	CG3307	chr3R	3.623302841	3.91E-05
151680_at	LD29477.3prime-hit	CG31152	chr3R	3.352439823	4.58E-06
152090_at	FBgn0032394	CG6746	chr2L	4.303765433	1.21E-05
152326_at	FBgn0037822	CG14683	chr3R	3.356372481	1.80E-07
152374_at	FBgn0037552	CG7800	chr3R	3.258847892	0.000322146
152470_at	FBgn0032793	CG10189	chr2L	3.184110972	1.62E-05
152700_at	FBgn0033123	CG30159	chr2R	4.584974871	7.86E-07
152714_at	FBgn0037975	CG3397	chr3R	4.811105639	1.86E-06
152900_at	FBgn0025454	CG8453	chr2R	75.07134506	5.86E-10
152940_at	FBgn0031516	CG9663	chr2L	4.090948677	2.00E-05
153015_i_at	FBgn0037269	CG31534	chr3R	5.680979335	4.07E-06
153082_at	FBgn0035542	CG11347	chr3L	18.57430052	5.17E-09
153109_at	FBgn0034521	CG13431	chr2R	3.427784566	1.33E-05
153313_at	FBgn0014011	CG8556	chr3L	11.60665924	3.99E-10
153392_at	FBgn0032148	CG18854	chr2L	97.54423491	3.00E-15
153400_at	FBgn0037279	CG1129	chr3R	3.373051081	1.61E-05
153557_at	FBgn0036354	CG10191	chr3L	4.076723262	1.25E-08
153756_at	FBgn0038992	CG31156	chr3R	4.240798166	4.11E-06
153911_at	FBgn0003459	CG3836	chr3L	5.321276724	0.000500335
153930_at	FBgn0034354	CG5224	chr2R	9.644781604	3.50E-08
153952_at	FBgn0036386	CG8833	chr3L	3.416854537	5.73E-05
153980_at	FBgn0026150	CG6291	chr2L	3.781154725	3.79E-09

154125_at	FBgn0036151	CG7590	chr3L	5.848986256	3.30E-05
154396_at	FBgn0020766	CG13348	chr2R	3.661167514	4.12E-06
154619_at	FBgn0001185	CG4694	chr2L	3.031556193	2.52E-06
154650_at	FBgn0036098	CG32067	chr3L	6.798930506	1.59E-07
154914_at	FBgn0031961	CG7102	chr2L	4.802989352	2.35E-06
154962_at	FBgn0034922	CG5602	chr2R	4.088583076	3.25E-05
